# Waveband specific transcriptional control of select genetic pathways in vertebrate skin (*Xiphophorus maculatus*)

**DOI:** 10.1186/s12864-018-4735-5

**Published:** 2018-05-10

**Authors:** Ronald B. Walter, Mikki Boswell, Jordan Chang, William T. Boswell, Yuan Lu, Kaela Navarro, Sean M. Walter, Dylan J. Walter, Raquel Salinas, Markita Savage

**Affiliations:** The Xiphophorus Genetic Stock Center, Molecular Biosciences Research Group, Department of Chemistry and Biochemistry, Texas State University, 419 Centennial Hall, 601 University Drive, San Marcos, TX 78666 USA

**Keywords:** Southern Platyfish, Light source, Wavelength, RNA-Seq, Differential gene expression, Skin, Transcriptional regulation

## Abstract

**Background:**

Evolution occurred exclusively under the full spectrum of sunlight. Conscription of narrow regions of the solar spectrum by specific photoreceptors suggests a common strategy for regulation of genetic pathways. Fluorescent light (FL) does not possess the complexity of the solar spectrum and has only been in service for about 60 years. If vertebrates evolved specific genetic responses regulated by light wavelengths representing the entire solar spectrum, there may be genetic consequences to reducing the spectral complexity of light.

**Results:**

We utilized RNA-Seq to assess changes in the transcriptional profiles of *Xiphophorus maculatus* skin after exposure to FL (“cool white”), or narrow wavelength regions of light between 350 and 600 nm (i.e., 50 nm or 10 nm regions, herein termed “wavebands”). Exposure to each 50 nm waveband identified sets of genes representing discrete pathways that showed waveband specific transcriptional modulation. For example, 350–400 or 450–500 nm waveband exposures resulted in opposite regulation of gene sets marking necrosis and apoptosis (i.e., 350–400 nm; necrosis suppression, apoptosis activation, while 450–500 nm; apoptosis suppression, necrosis activation).

Further investigation of specific transcriptional modulation employing successive 10 nm waveband exposures between 500 and 550 nm showed; (a) greater numbers of genes may be transcriptionally modulated after 10 nm exposures, than observed for 50 nm or FL exposures, (b) the 10 nm wavebands induced gene sets showing greater functional specificity than 50 nm or FL exposures, and (c) the genetic effects of FL are primarily due to 30 nm between 500 and 530 nm.

Interestingly, many genetic pathways exhibited completely opposite transcriptional effects after different waveband exposures. For example, the epidermal growth factor (*EGF*) pathway exhibits transcriptional suppression after FL exposure, becomes highly active after 450–500 nm waveband exposure, and again, exhibits strong transcriptional suppression after exposure to the 520–530 nm waveband.

**Conclusions:**

Collectively, these results suggest one may manipulate transcription of specific genetic pathways in skin by exposure of the intact animal to specific wavebands of light. In addition, we identify genes transcriptionally modulated in a predictable manner by specific waveband exposures. Such genes, and their regulatory elements, may represent valuable tools for genetic engineering and gene therapy protocols.

**Electronic supplementary material:**

The online version of this article (10.1186/s12864-018-4735-5) contains supplementary material, which is available to authorized users.

## Background

The perception of light by specialized retinal cells and the mechanism by which this leads to an organismal response are topics that have been well studied [1–3]. The genetics and evolution of photoreceptors have also been subjects of strong interest [4, 5], and recent genome level analyses have forwarded new insights into the array of potential photoreceptors in both terrestrial and aquatic vertebrates [6, 7].

Groundbreaking studies by many investigators over the past few decades have given us a reasonable understanding of light reception in the eye, and transduction of the light energy into a neural impulse. In the human eye, light signals are received from an estimated ≈130 million rods and cones. Collectively, these light signals are processed in the brain where the light information is transduced to the organs inciting appropriate genetic responses that have been selected for over evolutionary history [8, 9]. An inherent aspect of the molecular complexity of photoreception, from a genetic perspective, is the necessity for an appropriate genetic response to light to be based on the collective nature of the light normally received in any particular niche during the evolution of vertebrate species.

Fishes have long-served as valuable vertebrate models to study light reception, and it has been well-established that fishes possess non-visual photosensitive receptors in the pineal gland, brain and skin [9–11]. Indeed, fish organs dissected and exposed to light outside the animal show light responsive expression in gene sets involved with circadian cycling [12], and further, that re-entrainment of circadian clock gene expression can be induced in a wide array of zebrafish organs (i.e., brain, eye, fin, gill, gut, heart, liver, muscle, pineal, pituitary, skin, testis) after removal from the organism [6]. These findings, and others, suggest non-visual photoreception may be broadly expressed and active in many organ types.

**Fig. 1:**
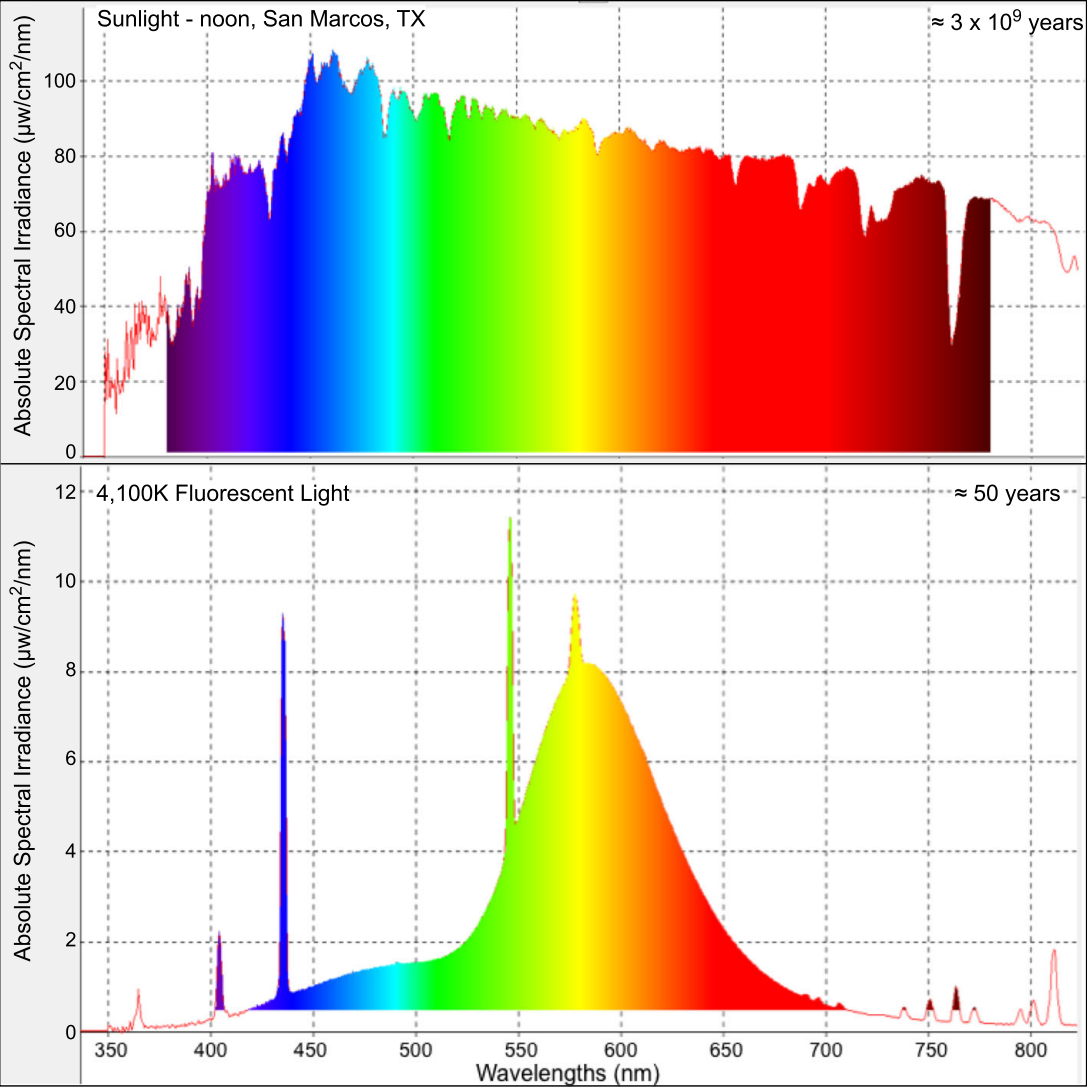
Complex spectra for sunlight (top) measured at noon in San Marcos, TX, and 4100 K FL (bottom). Middle (green peak) is 550 nm and increments are 50 nm

Evolution occurred over 3 billion years exclusively under the full spectrum of sunlight (Fig. 1). Thus, all spectral wavebands were represented during vertebrate evolution; however, due to specific niche physical pressures, organisms may have adaptively paired select genetic responses with different spectral regions (wavebands) and/or light intensities. In contrast to the solar spectrum, fluorescent light (FL) has only been in service for about 60 years and these light sources do not fully represent the solar spectrum (Fig. 1). If vertebrates evolved specific genetic responses that utilize select light wavebands within the solar spectrum, there may be genetic consequences to substantially reducing the spectral complexity of light.

In the wild, as cold-blooded vertebrates, fishes utilize light for warmth, predation, predator avoidance, coordination of diurnal metabolic activity, and as cues for monthly or yearly breeding cycles. Functional genomic analyses have shown existence of new and varied candidate photoreceptor genes in zebrafish [6] involving 42 new opsin genes that may produce 10 known visual and 32 non-visual opsins. These new genes hallmark four new opsin classes that seem to have arisen in zebrafish, possibly proceeding the teleost specific genome duplication [6, 13, 14]. The teleost specific genome duplication allowed each fish group to independently retain and alter new sets of visual and non-visual photoreceptor genes and thus, to receive and process light in new ways dependent on adaptation to their specific niche. However, the varied molecular genetic responses that may occur among fishes, after the intact organism is exposed to specific narrow wavelength regions (i.e., wavebands) of light has not been extensively investigated.

**Fig. 2:**
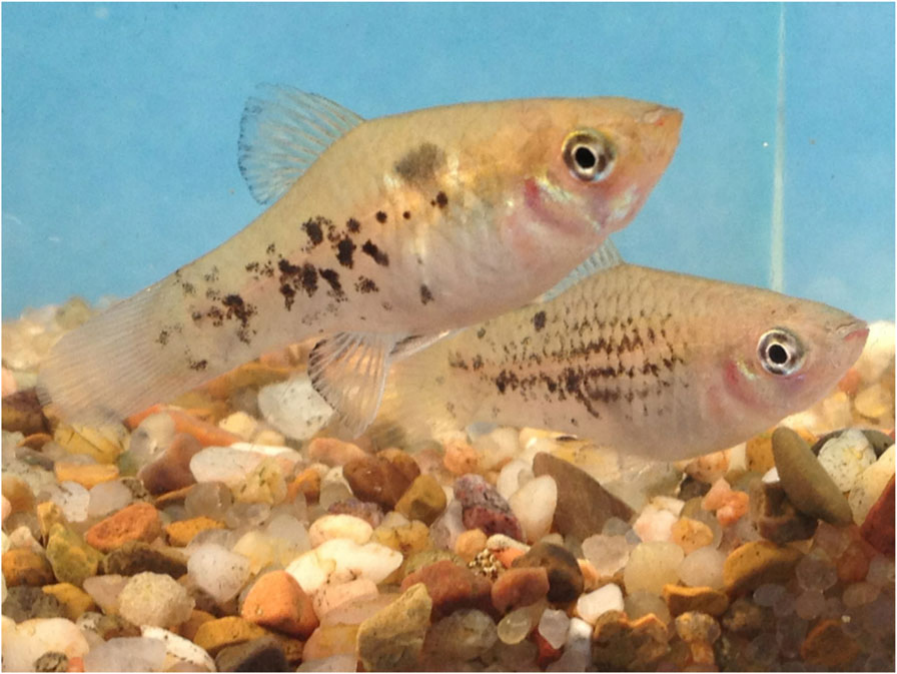
Example of *Xiphophorus maculatus* Jp 163 B male (bottom) and female (top). Male siblings in their 105th generation of brother-sister line breeding were utilized for the experiment detailed herein

To begin to address the question of light waveband induced genetic response, we have recently employed tractable fish models such as the live-bearing tropical fish, *Xiphophorus* (Fig. 2), to perform experiments detailing modulation of gene expression patterns in skin and other organs after exposure of the intact animal to various types of common fluorescent light sources (311 nm UVB, 4100 K or “cool white” FL, and 10,000 K or “sunlight” FL) [15–19], and to specific light wavebands between 300 and 600 nm [20].

Our recent studies establish exposure of *Xiphophorus* to commonly used FL leads to light source specific genetic signatures in the skin, and suggest each different light source may be expected to induce a different “homeogenetic” state in skin. Additionally, previous results of 50 nm waveband exposures, between 300 and 600 nm, have shown that each 50 nm waveband incites both unique, and a collective common set of shared, genetic responses. However, interestingly, two select 50 nm wavebands (350–400 nm, and 500–550 nm) exhibited more robust transcriptional effects than other wavebands tested in the 300–600 nm region [19].

Herein we present genetic responses of *X. maculatus* skin to FL exposure compared to exposure to 50 and 10 nm wavebands of light. We show that exposure of the intact animal to select 50 nm or 10 nm wavebands of light serves to induce waveband specific effects on the transcription of gene sets representing common genetic pathways. These novel results establish waveband specific transcriptional regulation of genetic pathways, and suggest experimental exposure to different light wavebands may be utilized to specifically up- or down-regulate gene expression within the same pathway. Should these novel waveband specific genetic effects be translatable to other vertebrate or mammalian systems, these results suggest select light waveband exposures may be employed as a facile regulator of genetic homeostasis to enhance experimental or therapeutic outcomes. We hypothesize that waveband specific genetic effects are deeply embedded in the vertebrate genetic repertoire and that once understood they may become useful tools for genetic engineering and gene therapy.

## Results

### Differential genetics of exposure to 4100 K (“cool white”) FL light

**Fig. 3:**
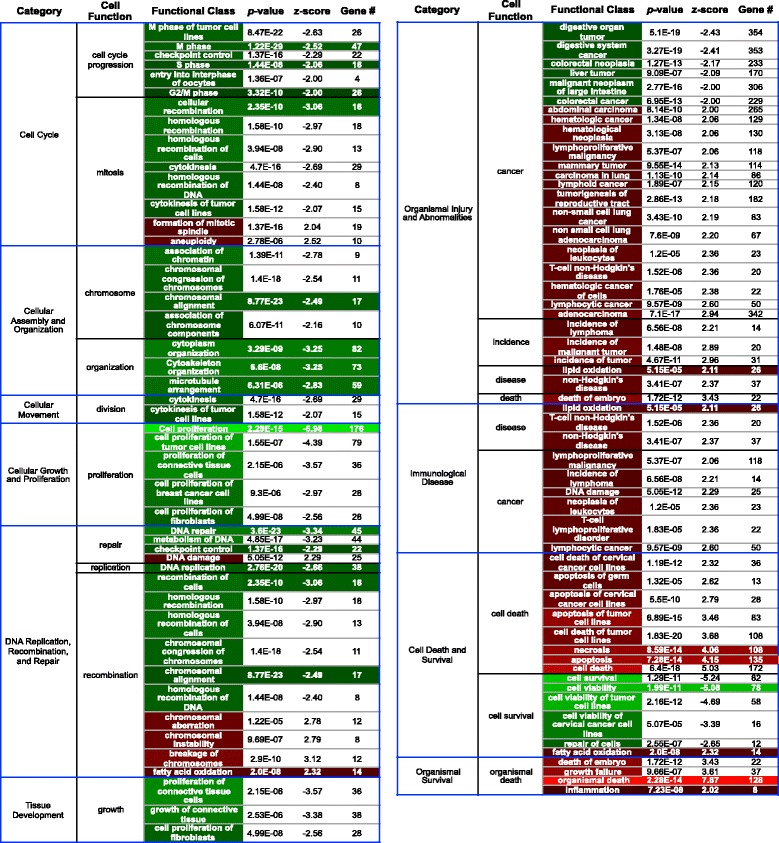
*X. maculatus* exposed to 35 kJ/m^2^ FL differentially modulated 413 genes which were analyzed using IPA (Qiagen). 391 genes (95% of the overall response) were able to be categorized into 74 statistically significant functional classes (z-score ± 2; 5 gene minimum); the remaining 5% of the genes modulated either failed to fall into a statistically significant functional class or could not be properly annotated and mapped by IPA. The 74 functional classes represent 10 primary categories; cell cycle; cellular assembly/organization; cellular movement; cellular growth/proliferation; DNA replication, recombination/repair; organismal survival; tissue development; organismal injury/abnormality; immunological disease; and cell death and survival. The 19 functional classes shared between FL and the 50 or 10 nm wavebands are shown by color highlighting the gene number. Compensatory effects due to the combined exposure by multiple wavebands is assumed to be responsible for the remaining 55 functional classes observed following FL exposure. For complete gene lists of each functional class see Additional file 2: Table S2a

Robust changes in the transcriptional state of *Xiphophorus* skin after exposure to various light sources have been reported [15–20]. For example, *X. maculatus* exposed to 35 kJ/m^2^ FL differentially modulates 413 genes that were analyzed using the Ingenuity Pathway Analysis (IPA; Qiagen, Redwood City, CA.) software suite. Of these 413 genes, 391 genes (95% of the overall response) were able to be categorized into 74 statistically significant functional classes (z-score ≥ |2|; 5 gene minimum; Fig. 3); the remaining 5% of the dataset either failed to fall into a statistically significant functional class or could not be properly annotated and mapped by IPA. The 74 functional classes represent 10 primary categories; cell cycle; cellular assembly and organization; cellular movement; cellular growth and proliferation; DNA replication, recombination and repair; organismal survival; tissue development; organismal injury and abnormality; immunological disease; and cell death and survival. The largest predicted functional effects, based on modulated transcriptional profiles following FL exposure, in male *X. maculatus* skin are suppression of cell growth/cell proliferation and an increase in organismal injury and abnormalities (Fig. 3). Due to the large effect FL exposure has on DNA replication, recombination and repair, coupled with cell cycle progression and cellular growth, it was not unexpected to see functional classes related to the onset of cancer where FL modulated genes are involved in several overlapping pathways, all related to cell proliferation. Suppression of cellular growth and proliferation has previously been suggested [19], and the results reported here represent 176 unique genes or approximately 43% of the total differentially modulated genes in the FL dataset. Supporting the suppression of cell growth and proliferation is the suppression of cell cycle progression (64 unique genes, 15% of the total dataset). While this encompasses the largest predicted response to FL, the overall transcriptional response to FL exposure is very complex and made up of multiple components that we suspected may directly reflect the spectral peaks emitted by FL. Surprisingly, although the spectral distribution is outside of the UVB and UVA range, genes associated with cell death are significantly represented (172 genes, z-score 5.03, *p*-value 6.4E-18) and indicate a potential suppression of cell viability and an increase in cell death through apoptosis and necrosis.

### Exposure to varied 50 nm wavebands leads to unique genetic responses

**Fig. 4:**
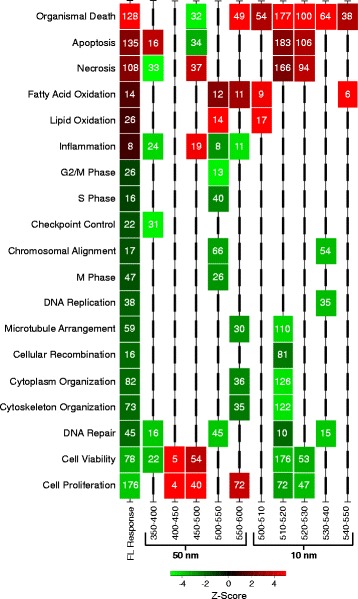
Functional classes (z-score ≥ |2|) were compared through the wavebands using Qiagen’s IPA software and plotted if they were shared with FL in any waveband from 350 to 600 nm. Heat map color represents IPA classified z-score (red is up and green is down) and the number inside each box represents the number of genes contributing to the functional effect. For complete gene lists of each functional class see Additional file 4: Table S4a–k

To attempt to assign wavelengths, and possibly FL spectral peaks represented in discrete wavebands, to the observed transcriptional effects predicting altered cellular functional pathways, we exposed male *X. maculatus* fish to 50 nm wavebands spanning the FL spectrum (350–600 nm). Gene sets involved with 19 of the functional classes transcriptionally modulated by FL were also found modulated after exposure of the animals to select 50 nm wavebands (Figs. 3 and 4). The 19 functional classes are shared in FL, and at least one 50 nm waveband, are highlighted in Fig. 3 (colored columns at right) and shown at the far left of Fig. 4. In both Figs. 3 and 4 the numbers of genes assigned to each functional class, ranging from 8 to 176, are shown in the appropriate colored box, where the color represents the direction (i.e., red, up-regulated and green, down-regulated) and magnitude (i.e., fold change or z-score color bars) of the response. Based on these results, compensatory effects due to the combined exposure inherent to the complex 4100 K FL emission spectrum are assumed to be responsible for modulation of the remaining 55 specific functional classes observed following FL exposure, but not incited by 50 nm waveband exposures.

We have previously reported transcriptional effects of 50 nm waveband exposure in male *Xiphophorus* skin unexpectedly showed two wavebands (350–400 nm and 500–550 nm; log_2_FC, fdr ≤ 0.05) that were able to incite significantly higher differential gene expression compared to any of the other 50 nm wavebands between 300 and 600 nm [20]. In these studies, we showed that exposure to either 350–400 or 500–550 nm wavebands led to modulation of circadian gene activity and altered the activity of TP53 gene targets, but via very different regulatory mechanisms; *ATM* at 350–400 and *ATR* at 500–550 nm [20]. Additionally, exposure to only the 500–550 nm waveband induced genes associated with cellular stress as a major response, while this gene set was not present in the genes showing differential transcription after exposure to the 350–400 nm waveband.

Further analysis of the 50 nm waveband exposure results led to identification of transcriptional responses that show opposite directional modulation of genes associated with major cellular pathways, such as necrosis and apoptosis. For example, *X. maculatus* skin exposed to 350–400 nm light (Fig. 4 and Additional file 1: Table S1) exhibit down modulation of transcription in 33 genes that collectively would be expected to produce a decrease in necrosis; however, exposure of the same fish (>100 generations inbred) to the same dose of FL, or the 450–500 nm waveband, leads to substantial up-regulation of 108 (FL) or 37 (450–500 nm) genes, involved with necrosis, and predicted to produce an overall increase in necrosis. Similarly, 450–500 nm exposure transcriptionally down-modulates 34 genes that predict an overall decrease in apoptosis (Fig. 4), but exposure to FL, or the 350–400 nm waveband, leads to increased transcription of apoptosis associated genes (Fig. 4; 135 for FL and 16 up-modulated for 350–400 nm, respectively). This opposite effect on transcription of necrosis and apoptosis associated genes after exposure to FL, or select 50 nm wavebands of light, may not occur in exactly the same gene targets (Additional file 1: Table S1), but does involve genes associated with necrosis and apoptosis function based on their similarity with human genes (e.g., assigned HUGO id’s) and functional clustering by IPA software. In any case, the results shown in Fig. 4 serve as examples of waveband selective transcriptional modulation among multiple specific gene targets within multigenetic functional pathways. Other examples of waveband selective transcriptional modulation within functional pathways, and/or oppositely modulated transcriptional effects among the 50 nm exposures are presented in Additional file 1: Table S1, both for FL and each 50 nm waveband.

### Select waveband exposures show increased specificity of gene expression patterns

To better understand the 50 nm waveband results regarding differential transcription of select pathways, we also exposed fish to 10 nm wavebands in the most gene responsive 50 nm waveband (i.e., 500–550 nm) [20]. Table 1 shows transcriptionally modulated gene numbers for 4100 K FL, the 50 nm wavebands between 300 and 600 nm, and for 10 nm wavebands within the 500–550 nm region. Interestingly, the overall numbers of genes modulated for each exposure is not matched to waveband width or spectral complexity, but each appears to have unique properties unto itself. For example, the 10 nm waveband between 510 and 520 shows more genes transcriptionally modulated (763) than any other exposure, even more than exposure to the comparatively complex 4100 K FL spectra (413). In contrast, the 540–550 nm waveband shows very few transcriptionally modulated genes (162 genes), compared to the other 10 nm waveband exposures.

**Table 1:** Numbers of genes transcriptionally up-modulated, down-modulated and total after exposure to FL or each of the various wavebands

Exposure	Up	Down	Total
4100 K FL	52	361	413
350–400 nm	59	58	117
550–550 nm	177	168	345
500–510 nm	82	141	223
510–520 nm	233	530	763
520–530 nm	169	241	410
530–540 nm	90	189	279
540–550 nm	45	116	161

In Fig. 4, the 19 functional pathways exhibiting the largest degree of transcriptional modulation after exposure to 4100 K FL, that are shared in at least one differentially regulated functional gene class after the 50 nm or 10 nm waveband exposures, is presented. The functional class box color in Fig. 4 represents the degree of up- (red) or down-regulation (green) based on the z-score (inset color bar). These functional classes of light waveband responsive genes represent the most affected pathways for 4100 K FL, 50, and 10 nm exposures, as determined by the numbers of genes modulated. For each functional class of genes represented, the numbers of differential transcriptionally modulated genes are shown inside functional class boxes. The FL dose was 35 kJ/m^2^, while does of all waveband exposures were ≈18 kJ/m^2^. As shown, some of the gene sets exhibiting predicted transcriptional effects in a functional class that are incited by FL exposure are observed to segregate into specific 50 nm exposures. For example, the 500–550 nm waveband incited the most robust transcriptional response, modulating 8 of the 19 functional classes initially observed in the 4100 K FL exposure and representing 224 (65%) of the total 345 genes modulated among the 19 classes. Transcriptional modulation of these eight classes of genes after 500–550 nm exposure largely mirrored the direction (i.e. up- or down-modulation) and gene numbers modulated after 4100 K FL exposure, with a few exceptions (e.g., Chromosomal alignment/Movement, S phase showing higher numbers of genes effected, while M and G2/M phases showed lower numbers of effected genes). This suggests the large-scale suppression of cell cycle associated genes previously reported for FL exposure [19] is principally due to the 500–550 nm waveband, a minor component of the 4100 K FL spectrum (Fig. 1).

Aside from the 500–550 nm waveband, the other 50 nm waveband exposures each exhibited only a few shared functional class transcriptional effects observed for FL exposure (e.g., 400–450-2, 450–500 – 4, and both 350–400 and 550–600 nm shared modulation in 5 of the 19 functional classes). Thus, many of the 4100 K FL functional class responses were segregated into separate 50 nm wavebands. However, in many cases the waveband specific transcriptional effects were opposite in direction, when compared to the corresponding effected group of genes after FL exposure. For example, the 450–500 nm waveband exhibited exclusive increased transcription of genes associated with inflammation (19 genes), cell viability (54 genes) and cell proliferation (40 genes). Concurrently, the 450–500 nm waveband exposed skin exhibited decreased transcription in genes associated with organismal death (32 genes), and apoptosis (34 genes), but interestingly, up-regulated genes associated with necrosis. With the exception of necrosis and inflammation, the 450–500 nm waveband served to transcriptionally up-modulate functional class gene sets in the opposite direction from 4100 K FL exposure.

There appear many classes of genes among the 50 nm waveband exposures that exhibit waveband specific transcriptional response, or show, waveband specific and opposite transcriptional response, compared to exposure by the complex FL light source (e.g., note alternating green and red boxes as one moves from left FL to the right within the 50 nm bands of Fig. 4).

To further investigate waveband specific transcriptional responses, we exposed fish to successive 10 nm wavebands within the most responsive 50 nm waveband (i.e., 500–550 nm; Fig. 4, right and Additional file 2: Table S2 g–k). Here we make three summative observations; (1) there are much greater numbers of genes affected in many of the 10 nm functional classes than in the corresponding 50 nm or FL functional class. For example, “organismal death” has 22 genes modulated after 4100 K FL, while the 510–520 nm exposure shows 177 modulated genes in this class. In the “microtubule arrangement” class, 4100 K FL exposure led to transcriptional modulation of 59 genes, whereas the 510–520 nm exposure shows 110. (2) There appears a higher degree of functional specificity segregating into specific 10 nm exposures than observed in the 50 nm exposures. For example, the 500–510 nm exposure only up-regulates lipid metabolism functions, 510–520 nm seems to primarily down-regulate cell division and correspondingly up-regulate organismal death, 520–530 nm is central to cell death and necrosis pathways, 530–540 nm primarily affects genes involved in DNA replication, repair and chromosome structure which are likely overlapping functions of the same genes, whereas 540–550 nm exposure is rather innocuous, only up-regulating four genes involved with fatty acid biosynthesis. (3) The greatest effects of FL or 500–550 nm exposures in skin are sequestered into only 3 of the 10 nm exposures (i.e., 30 nm between 500 and 530 nm). There is a split in functional responses between the 500–510 nm and 510–520 nm. Although these exposures are only 10 nm apart, the transcriptional responses at 500–510 nm (lipid metabolism) and 510–520 nm (cell reorganization and cell death) are expected to have very little overlap in function and result in very different effects in the skin.

**Fig. 5:**
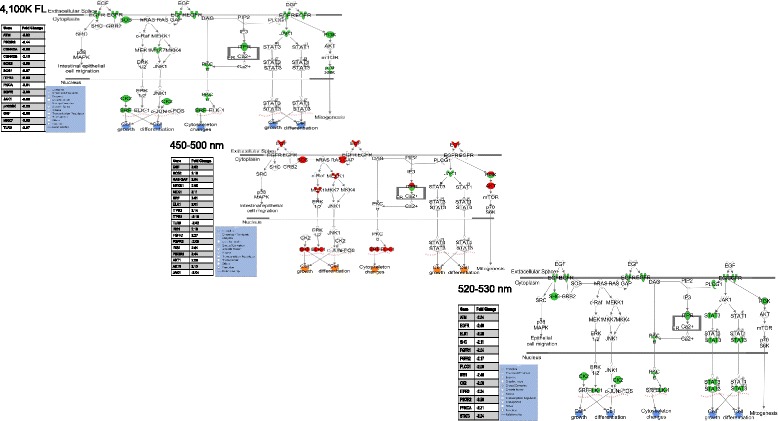
Waveband specific differential transcriptional regulation of the epidermal growth factor (*EGF*) pathway. The EGF pathway is suppressed when fish are exposed to 4100 K FL or 520–530 nm waveband light (top and bottom). In these two cases, the transmembrane EGF receptor is down-modulated as are many downstream gene targets of the pathway. However, when the fish are exposed to 450–500 nm waveband light (center) EGF transcription is up-modulated. The EGFR is present and predicted to be ligand bound leading to up-regulated transcription of most downstream targets. Each molecule differentially up modulated is represented in red and down represented in green. Downstream effects are predicted to be activated (orange) or suppressed (blue) based on IPA z-score. Complexes consist of multiple genes represented by a single symbol in the figure (4100 K: PI3K-TLR9, PIK3R6, ATM; SOS-SOS1, SOS2; ITPR-ITPR2; CK2-CSNK2A, CSNK2B. 450–500 nm: SOS-SOS2; ITPR-ITPR2, ITPR3; PI3K-TLR9, IRS1, FGFR1, FGFR2, IRS2, PIK3R3; AKT-AKT1, AKT3. 520–530 nm: ITPR-ITPR3, PI3K-ATM, FGFR1, FGFR2, IRS1, PIK3R2; PKCα: PRKCA)

Waveband dependent differentially regulated transcriptional gene clusters were further analyzed for effects on specific genetic and/or biochemical pathways. For example, Fig. 5 shows the epidermal growth factor (*EGF*) pathway may be experimentally manipulated by varied waveband exposures. The *EGF* response shows suppression in 4100 K FL exposed skin (green colored gene targets, Fig. 5), then switches to exhibit transcriptional activation after 450–500 nm exposure (red colored gene targets, Fig. 4), then once again exhibits transcriptional suppression upon exposure to 510–520 nm light. As shown, gene target responses within the *EGF* pathway transcriptionally suppressed by 4100 K FL exposure (Fig. 5, top) involve, *JAK1*, *PLCG1*, and *PI3K* regulators. However, after organismal exposure to 450–500 nm light, this same pathway is highly transcriptionally up-regulated (Fig. 5, middle) with *SOS*, *RAS*, *PI3K* regulators all showing enhanced transcription. Exposure to 520–530 nm waveband light once again exhibits suppression of *EGF* pathway through reduced transcription of *EGFR*, *PLCG1*, *SHC*, *PI3K*, *PKC*, *STAT1*, *STAT3*, *CK2* and *LK1* (Fig. 5, bottom).

**Fig. 6:**
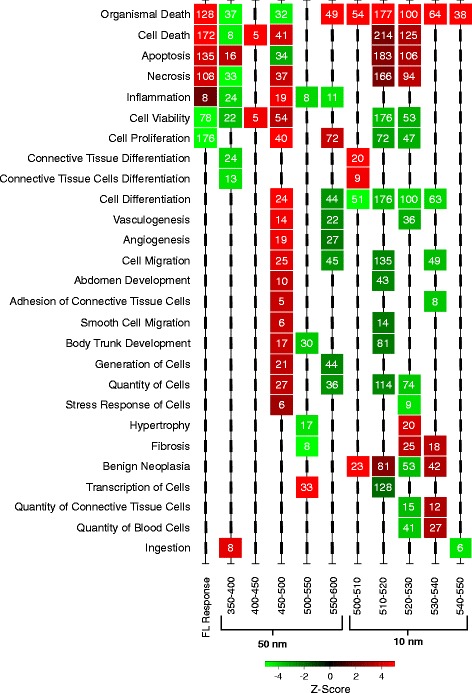
A compilation of oppositely modulated functional classes (z-score ≥ |2|; minimum of five genes) were compared through the wavebands using IPA software and plotted. Heat map color represents IPA classified z-score (red is up and green is down) and the number inside each box represents the number of genes contributing to the functional effect. Functional classes had to be oppositely modulated in at least one waveband or FL exposed samples to be plotted. For complete gene lists of each functional class see Additional file 5: Table S5a–k

The *EGF* situation shown in Fig. 5, wherein different waveband exposures lead to opposite transcriptional regulation of discrete pathways is not uncommon in *X. maculatus* skin. In Fig. 6, we show 25 functional classes based upon modulated transcription of gene sets that clearly exhibit opposite transcriptional responses between any two of the FL, 50 nm or 10 nm waveband exposures (Fig. 6, note color change from red to green in boxes as one moves from left to right). Within the functional classes, that each exhibit waveband specific regulation, are many specific biochemical and/or genetic pathways that may be manipulated by specific waveband exposures of the intact animal. Additional file 1: Table S1 presents all functional classes that are either uniquely regulated with specific waveband exposures, or are oppositely regulated by any two of the 50 nm or 10 nm waveband exposures, along with numbers of genes transcriptionally modulated in each class.

### Determination of genetic effects of exposure to waveband combinations

**Fig. 7:**
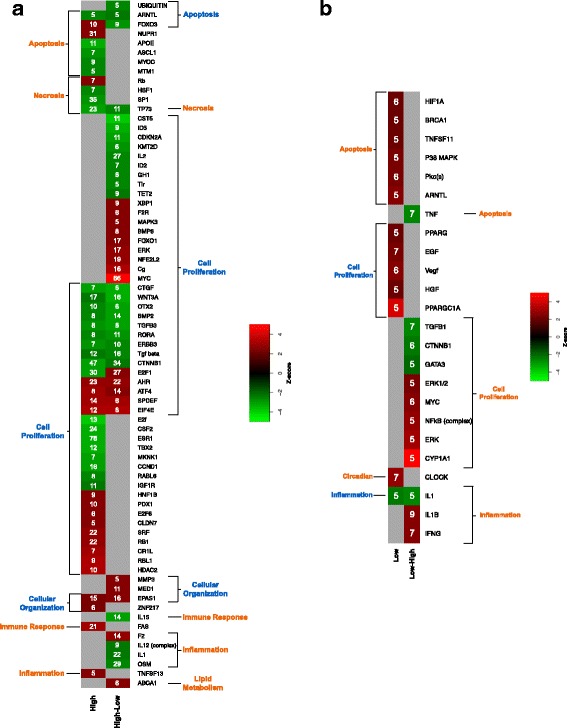
Panel (**a**) Results from *X. maculatus* skin after exposure to 510–520 nm (High) of light, or 510–520 nm of light immediately followed by exposure to 350–360 nm of light. When exposed first to 510 nm of light and then 350 nm of light (High-Low) the effect observed mimics the solely exposed 510–520 nm waveband results where 18 upstream regulators are shared by the two exposure types. Dominate functions effected by either exposure regimen effect apoptosis, necrosis, cell proliferation, cellular organization, immune and inflammation response and lipid metabolism. Of all pathways modulated, only apoptosis is oppositely modulated; up (orange) following 510–520 nm exposure and down (blue) following the High-Low exposure. The numbers of genes showing modulated transcription in each functional pathway are indicated by the number inside of each red (up-modulated) and green (down-modulated) box. For complete gene lists of each upstream regulator see Additional file 6: Table S6a–d. Panel (**b**) *X. maculatus* exposed to 350–360 nm (Low) of light compared to *X. maculatus* exposed to 350–360 nm of light immediately followed by exposure to 510–520 nm (Low-High) of light. When exposed first to 350 nm of light and then 510 nm of light, the observed effects are quite different from the inverse “High-Low” exposure. In this case many fewer functional classes are effected, consistent with results from single exposure of 350–360 nm vs, 510–520 nm exposure. However, of the four upstream classes effected all are oppositely regulated by the Low-High exposure, compared to the 350–360 nm exposure except apoptosis. Apoptosis is up regulated by either 350–360 nm or the Low-High exposure regimen. Not only are the main functional groups oppositely modulated in the Low-High exposure, when compared to the 350–360 nm waveband, but also when compared to the High-Low exposure (510 followed by 350 nm, left). This suggests that although the light effect is largely muted, it is dominated by a unique set of gene regulators that oppositely effect the downstream cellular and metabolic functions. For complete gene lists of each upstream regulator see Additional file 6: Table S6a–d

As it is observed that wavebands (i.e. 50 and 10 nm) are capable of shifting the gene expression profiles of *Xiphophorus* skin, we wished to determine if one waveband exposure may offset and/or further modulate the genetic effect of a different waveband, or if combining two waveband exposures may result in a completely new genetic effect, due to combined effects of both wavebands. As an initial test, we selected two waveband regions, 350–360 nm and 510–520 nm that produce two different genetic response peaks [19], and are positioned 150 nm from each other in the visible spectrum. We exposed fish to either; each waveband singly (i.e. 350–360 nm or 510–520 nm), and also to dual exposures by one waveband that was immediately followed by exposure to the second waveband (e.g., 350–360 nm and immediately followed by exposure to 510–520 nm). In Fig. 7 panel a, we show results of *X. maculatus* exposed to 510–520 nm of light singly, compared to *X. maculatus* exposed to 510–520 nm followed immediately by exposure to 350–360 nm light. When first exposed to 510–520 nm and then followed by the 350–360 nm waveband, a dominant effect of the longer waveband (i.e., 510–520 nm) is observed. The dual exposed skin shows modulation of gene expression in some of the same and many different genes, but overall reflects a similar overall functional class response as the singly exposed 510–520 nm fish skin. Of the functional categories effected, by modulated regulators and gene sets, only apoptosis is predicted to be oppositely modulated (up-orange, following 510–520 nm single exposure, and down-blue following the dual exposure).

In contrast, when the order of light exposure is reversed (Fig. 7 panel b), wherein 350–360 nm exposure is immediately followed by 510–520 nm exposure, only four upstream regulators are shared with the singly exposed 510–520 nm fish skin. These four upstream regulators are all modulated in the same direction, while the combined transcriptional effects with the other regulators modulated in the 350–360 nm/510–520 nm dual exposure are predicted to result in a directional change in functional response.

As shown in Fig. 7 panel b, there is only one common regulator of the dual exposed skin that mirrors the 350–360 nm singly exposed skin, apoptosis. All other principal functional categories appear to be oppositely modulated (e.g., cell proliferation and inflammation), compared to single 350–360 nm exposure. Thus, not only is the dual exposed skin exhibiting predicted opposite functional modulation compared to the 350–360 nm single waveband exposure, but also when compared to the dual exposed 510–520 nm followed by 350–360 nm skin (Fig. 7 panel a). Based on these results it appears the longer waveband (510–520 nm) exerts a dominant effect on the capacity of the lower wavelength (350–360 nm) to modulate transcription in *Xiphophorus* skin.

### Validation of RNA-Seq determined transcriptional responses after waveband exposures

To confirm RNA-Seq determined gene expression changes for FL and waveband light exposures, we employed an independent platform, the NanoString nCounter®, for direct assessment of gene expression. The nCounter® platform works as a simultaneous gene expression detection system for many transcripts [21–24]. The technology employs oligonucleotide capture and reporter sequences complementary to each target gene [21]. We have developed and utilized [15, 19, 20] a *Xiphophorus* 200 gene target light responsive NanoString panel and used this to determine transcriptional modulation after the various light exposures presented herein. Additional file 3: Table S3 presents the *Xiphophorus* gene targets and capture probes employed in these analyses (also see, http://www.xiphophorus.txstate.edu/NanoString). For gene expression analyses, this panel also contains 10 housekeeper genes that have been extensively tested and shown to exhibit unmodulated expression upon light exposures and within various tumor types. These 10 housekeepers represent three statistical categories (high, medium, low gene expression) and are utilized for normalization.

**Fig. 8:**
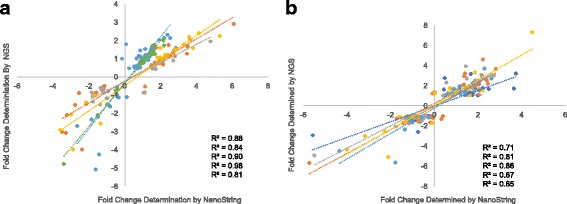
To confirm RNA-Seq determined fold changes, NanoString nCounter Analysis was performed. For complete target and probe information used for confirmation see Additional file 3: Table S3. Panel (**a**) Log_2_(Fold Change) values for each 50 nm waveband exposure were determined by each independent technology and plotted. Analysis of the 350–400 nm region (50 transcripts) is in orange, the 400–450 nm region (22 transcripts) is in gray, the 450–500 nm region (19 transcripts) is in yellow, the 500–550 nm region (59 transcripts) is in light blue and the 550–600 nm region (26 transcripts) is in green. R^2^ values were calculated for each correlation and determined to be 0.88, 0.84, 0.90, 0.98 and 0.81 respectively for each of the 50 nm region. In addition, analysis of the correlation indicated that 97% of the transcripts tested across all wavebands were confirmed in direction. Panel (**b**) To confirm RNA-Seq determined fold changes, NanoString nCounter Analysis was performed. The plot shows log_2_(Fold Change) values determined for each 10 nm waveband exposure by each independent technology. Analysis of the 500–510 nm region (30 transcripts) is in dark blue, the 510–520 nm region (37 transcripts) is in orange, the 520–530 nm region (24 transcripts) is in gray, the 530–540 nm region (32 transcripts) is in yellow and the 540–550 nm region (23 transcripts) is in light blue. R^2^ values were calculated for each correlation and determined to be 0.71, 0.81, 0.86, 0.87 and 0.85, respectively for the 10 nm region. Analysis of the correlations indicate 94% of the transcripts tested across all wavebands confirmed in the direction of modulated expression

In Fig. 8 we present plots indicating log_2_(Fold Change) values determined by each independent technology, RNA-Seq or NanoString. In Fig. 8, panel a, each 50 nm waveband exposure is plotted independently. Analysis of the 350–400 nm region (50 transcripts) is in orange, the 400–450 nm region (22 transcripts) is in gray, the 450–500 nm region (19 transcripts) is in yellow, the 500–550 nm region (59 transcripts) is in light blue and the 550–600 nm region (26 transcripts) is in green. R^2^ values were calculated for each correlation and determined to be 0.84, 0.88, 0.90, 0.81 and 0.98, respectively for each of the 50 nm regions. In addition, for the 50 nm waveband data, analysis of the correlation indicated that 97% of the transcripts tested across all wavebands were confirmed in direction.

In Fig. 8, panel b we show log_2_(Fold Change) values determined by RNA-Seq or NanoString for each 10 nm waveband exposure plotted independently. Analysis of the 500–510 nm region (30 transcripts) is in dark blue, the 510–520 nm region (37 transcripts) is in orange, the 520–530 nm region (24 transcripts) is in gray, the 530–540 nm region (32 transcripts) is in yellow and the 540–550 nm region (23 transcripts) is in light blue. R^2^ values were calculated for each correlation and determined to be 0.71, 0.81, 0.86, 0.87 and 0.85 respectively for the 10 nm region. For the 10 nm exposures, analysis of the correlation indicated that 94% of the transcripts tested across all wavebands were confirmed in direction. Validation of the 4100 K FL exposure samples by NanoString analysis, and by quantitative real-time PCR have been previously reported [15, 19, 20].

## Discussion

Evolution occurred under full spectrum sunlight. Unlike artificial light, the solar spectrum produces intense irradiance across all wavebands in the visible spectrum from 400 to 700 nm (Fig. 1). Competition for resources under the solar spectrum led to development of the eye and other photoreceptive mechanisms that make efficient use of light as cues for biological responses. It is likely the same selective pressures that led to complex structures, such as the eye, were concurrently adapted to make genetic use of all wavelengths or wavebands within the solar spectrum. The perception of light and the mechanism by which it leads to signal transduction, in the sense of neural excitation, are topics that have been well-studied at the biophysical, cell biological, and physiological levels. In addition, the evolution of photoreceptors has been a recent subject of strong interest [3–12]. Light reception in the eye and transduction of the photon energy into a neural impulse has been established to involve specific photoreceptor cells, rods and cones. These cells are associated with members of a very large family of proteins, termed opsins, that are fine-tuned to receive different wavelengths in the color spectrum. Cones are clustered into long wavelength, L cones (maxima at ≈560 nm, waveband range 400–675 nm), medium wavelength, M cones (maxima ≈539 nm, waveband range 400–650 nm) and short wavelength, S cones (maxima ≈420 nm, waveband range 325 to 524 nm). The three classes of vertebrate cones differ in their absorption spectra because of differences in the amino acid sequence of the associated opsins. Therefore, it is well-established that absorption of specific wavelengths of light is utilized by animals to incite activation of a signal cascades that translate the light signal into a neural signal and a behavioral response [4, 5]. In the brain, light signals received from hundreds of millions of rods or cones in each eye are processed, and signals for appropriate actions sent to the vertebrate organs. However, it is also recognized that among vertebrates, non-visual mechanisms exist for photoreception, and with the exception of known role of melanopsins in circadian regulation, these are less well studied. Non-visual photoreception may be considered a master regulator of fundamental organismal biology that exerts control on both behavioral and physiological functions (e.g., circadian rhythms, cell division cycles, DNA repair, replication, etc.). Recent genomic comparative data has uncovered the presence of many more photopigments and photoreceptor molecules in vertebrates than previously thought to exist [5–7]. Conservation of these newly discovered sets of photoreception proteins among vertebrate genomes underscores non-visual photoreception as an understudied, but potentially significant, mechanism for organismal adaptation and survival.

Results presented herein depart from the well-represented fields of light perception that involve phototransduction, photobiology and photochemistry. Instead, our approach employed RNA-Seq to ask only about the genetic transcriptional response to light. In the detailed experiments, we utilized highly inbred (<105 generations) live-bearing fish of the genus *Xiphophorus* (http://www.xiphophorus.txstate.edu/) to assess transcriptional changes in skin after FL or specific waveband exposures. During our experimental treatments, fish were maintained individually in 125 ml beakers containing 100 ml of water. The water utilized was changed at each experimental stage (i.e., pre-incubation, treatment chamber, post-exposure incubation) from the same large carboy for all fish in a particular experiment. Importantly, all control, sham and light exposed fish were carefully maintained in the exactly the same manner. Extrinsic experimental parameters that may have arisen during the overnight or post-exposure incubation periods were controlled by identically treated unexposed control, and sham treated, fish replicates. The sham treated fish replicates received identical treatment as the light exposed replicates, but with the light sources turned “off”. Thus, parameters other than light exposure that may change the genetic profile would not show up in our analyses of differentially expressed genes, since they would also have been present in the unexposed control, or sham treated fish, to which all light treated fish were compared.

The 26 known species of *Xiphophorus* are tropical, ranging from northern Mexico, and south along the Sierra Madre uplift, into Belize and Honduras [25]. These fish are known for a variety of expressed pigment patterns and these patterns are thought to play a role in complex courting behavior necessary for live-bearing animals to select mates [26]. We employed a simple experimental protocol involving; (a) exposure to the same dose of fluorescent light (4100 K, 35 kJ/m^2^), or selected wavebands of light (50 nm or 10 nm wavebands, ≈18 kJ/m^2^), (b) post-exposure incubation in the dark (6 h) to allow time for genetic remodeling, and (c) RNA-Seq transcriptional profiling to assess the genetic response incited by each selected light exposure. It is worth noting that FL lights have only been employed for ≈60 years (https://energy.gov/articles/history-light-bulb) and the spectral complexity they produce is dissimilar to the solar spectrum (Fig. 1). Also, in regard to the narrow waveband exposures, the exposures performed in the detailed experiments may be the first time in history that a vertebrate animal has been subjected to, exclusively, a 50 nm or 10 nm waveband of light.

Thus, observations made in these studies imply that waveband specific genetic circuitry, whereby specific wavebands incite predictable genetic response, is embedded in the vertebrate genome. That is, specific wavebands may have been conscripted over evolutionary time for specific utility in genetic regulation. A recent paper detailing the comparative genetic responses in the skin, brain and liver, after FL exposure of three divergent fish model systems; *Xiphophorus*, medaka (*Oryzias latipes*) and zebrafish (*Danio rerio*) [26] supports this concept.

Given the observed post-FL modulation of transcription among gene sets involved with many varied functional classes, we attempted to de-convolute the complex FL transcriptional effects by exposure to specific 50 nm wavebands between 350 and 600 nm. Here, we were able to define a small portion of the genome embedded response to each waveband, since again, no fish would have seen a 50 nm or 10 nm waveband of light until this experiment. The response of 50 nm waveband exposures on gene expression in *Xiphophorus* skin established two 50 nm wavebands (350–400 nm and 500–550 nm) that produced significantly higher differential gene transcriptional modulation than the other wavebands [20]. However, in-depth analysis led to the surprising observations that each specific 50 nm waveband was able to selectively modulate transcription of specific gene sets and/or pathways. Indeed, exposure of the animal to 50 nm wavebands that are very close to each other (e.g., 350–400 and 400–450 nm, Fig. 4) or separated by 100 nm (350–400 and 450–500 nm, Fig. 6), resulted in suppression, or activation, of genes involved in the same functional classes (Figs. 4 and 6, also see Additional file 1: Table S1). These novel findings support the concept that exposure to select wavebands may be utilized to alter expression of specific genetic pathways and thereby affect the downstream genetic response of the animal to experimental protocols. Further, as noted above, the concept stemming from these studies is that one may alter the homeogenetic transcriptional state of skin and push the genetic profile toward a new position by exposure of the intact animal to specific light wavebands. Once these effects are well-documented, and better understood, it appears likely waveband specific gene expression of select pathways may become predictable, and useful as a tool in research investigations or gene or pathway specific targeted therapies.

Exposure of fish to 10 nm wavebands within the 500–550 nm region indicate the numbers of genes modulated for each light exposure is not matched to the width of the waveband, but instead each waveband has its own unique induction response that must be empirically characterized (Table 1). This may be due to slightly different total doses for each waveband (see Materials and Methods), but we feel this is unlikely since the total energy delivered did not vary appreciably among the waveband exposures between 350 and 600 nm (i.e., 350–400 nm [18.6 kJ/m^2^], 400–450 [21.6 kJ/m^2^], 450–500 [21.2 kJ/m^2^], 500–550 [17.4 kJ/m^2^] and 550–600 [13.8 kJ/m^2^]). Further, the genetic differences between wavebands does not seem to track with differences in dose for either 50 nm or 10 nm waveband results. Thus, if specific genetic transcriptional signaling pathways have evolved to be responsive to specific wavelength regions (i.e., wavebands) of light, as suggested from the data in Figs. 4, 5 and 6, then narrower 10 nm waveband exposures delivered a stronger stimulus to the reception apparatus than broader 50 nm wavebands or the complex FL source. This may partially explain the observed general trend in higher numbers of genes transcriptionally modulated by the narrower wavebands (i.e., 10 nm) compared to the 50 nm wavebands within shared functional classes (Fig. 4 and Additional file 1: Table S1). However, not all functional classes showed this enhanced gene representation as waveband exposure narrowed, and many exhibited completely opposite transcriptional modulation of responsive genes (Fig. 6, Additional file 1: Table S1) as exemplified by the *EGF* pathway (Fig. 5).

The 10 nm waveband results suggest the greatest functional effects of complex FL (or 500–550 nm) exposure in skin are sequestered to 30 nm between 500 and 530 nm with a major split in functional effects between 500 and 510 nm (lipid metabolism) and 510–520 nm (organismal death) (Fig. 4). Additionally, the novel observations that specific pathways, such as *EGF*, may be selectively up- or down-modulated by exposure to varied wavebands of light is consistent with the differential expression observed for transcriptional expression patterns identified after complex FL exposures, where each different FL light type elicits a unique light source specific genetic response signature, in addition to shared transcriptional responses (data not shown). Given the waveband specific effects on transcriptional profiles in fish skin presented herein, and the fact that FL spectra do not reflect the complexity of sunlight (Fig. 1), an obvious question becomes: Does reduction in external light complexity have genetic consequences associated with it? Herein we only present the transcriptional effects of exposure to varied light wavebands. The degree and type(s) of effect(s) alternative lighting may, or may not, have on organismal biology is a subject for future research.

However, the innovative concept stemming from these results is that one may be able to manipulate the homeostatic genetic state of an organ, or tissue, by exposure of the intact animal to specific light wavebands. Ongoing studies using medaka (*Oryzias latipes*) and zebrafish (*Danio rerio*), to be reported elsewhere, have established that waveband specific transcriptional effects are not skin specific, but also extend to the internal organs (i.e., brain, liver). However, the mechanism(s) that have evolved that allow transduction of specific waveband signals through the organism to produce molecular genetic responses within internal organs are currently unknown.

Evolution under the solar spectrum may have segregated select genetic responses to different spectral regions (wavebands) and/or intensities. Based on gene family analyses (not shown), the observed light-driven regulatory circuits are ancient and largely conserved in the genomic circuitry of vertebrates, perhaps including mammals [26]. However, each biomedical model uniquely evolved over many millennia to their specific habitat, and therefore, would be expected to have their gene expression patterns fine-tuned to the light type and the light cycle of their environmental niche. The evolutionary fine-tuning of light-driven transcriptional regulation under a complex solar spectrum may involve both agonist and antagonistic pathway effects, that only in combination produce the proper adaptive response by the organism, as it synchronizes its behavior to the environmental conditions. If this is tenable, it suggests moving organisms into brightly lit FL environments, as exists in many animal facilities, may not provide the full antagonistic/agonistic signals needed for genetic calibration of the homeogenetic state. Likewise, the advent of less complex artificial lighting and concurrent extension of day length, may hallmark similar aberrant shifts in genetic homeostasis in higher mammals, including humans. Despite the very rapid culturally driven changes in light conditions, the molecular genetic response of animals to changing light types has not been studied in detail. Given the results presented herein, a prudent path for future studies may be to assess the breadth of waveband specific genetic changes among a representative set of vertebrates including mammals.

## Conclusions

Changes in the transcriptional profiles of *Xiphophorus maculatus* skin after exposure to successive 50 nm wavebands of light between 350 and 600 nm allowed identification of waveband specific transcriptional modulation of gene sets representing discrete functional pathways within *Xiphophorus* skin.

Further investigation of waveband specific transcriptional modulation employing successive 10 nm waveband exposures within the genetically responsive 500–550 nm region showed; (a) greater numbers of genes may be transcriptionally modulated after 10 nm exposures, than observed for either 50 nm or FL exposures. (b) the 10 nm wavebands incited functional specificity, and (c) the principal genetic effects of FL are due to 30 nm between 500 and 530 nm.

A large set of genetic pathways were identified exhibiting opposite transcriptional effects after different select waveband exposures.

Collectively, these results suggest one may manipulate transcription of specific genetic pathways in skin by exposure of the intact animal to select wavebands of light. We identify gene targets that may be transcriptionally modulated in a predictable manner by specific waveband exposures. Such genes, and their regulatory elements, may represent valuable tools for genetic engineering and gene therapy protocols.

## Methods

### Fish utilized and light exposures

All studies were approved by the Texas State University Institutional Animal Care and Use Review Board (IACUC protocol #2015107711). Fish used in this study were maintained and utilized in accordance with the applicable OLAW guidelines governing animal experimentation in the USA, and international legislation regulations governing animal experimentation.

*Xiphophorus* utilized in these studies were bred and maintained in the *Xiphophorus* Genetic Stock Center (http://www.xiphophorus.txstate.edu/). Fish were sacrificed by over-anesthetization with MS222 (0.06%). Fish utilized were mature male *X. maculatus* Jp 163 B, 10 to 11 months of age, from the 105th generation of sibling inbreeding. Skin from four individual fish taken straight out of the dark cycle without any experimental treatment or handling, were utilized for RNA isolation and Illumina sequencing (controls). For each light exposure three fish were individually exposed, and RNA from the skin of these exposed fish subjected to Illumina sequencing and utilized for RNA-Seq or NanoString nCounter® analysis. Prior to all light exposures, each fish was placed into an individual 125 mL flask filled with 100 mL of filtered water from their home aquaria and kept in the dark 14 h. Fish from these individual flasks were randomly chosen for light exposure or to remain unexposed and serve as controls. Sham animals were treated as those exposed to light except the light source remained off during the sham treatment.

FL Light exposures were carried out essentially as previously detailed [19]. Briefly, FL exposure occurred in a specially designed wooden box (77 cm in length, 41 cm in height, and 36 cm in depth), with a hinged wooden lid capable of sealing the interior of the box from external light. On the bottom of each of the two sides (41 cm × 36 cm) were 15.5 cm diameter high-speed fans that maintained interior temperatures of the closed box at less than 24 °C. For FL exposure, single fish were placed into UV transparent (UVT) plastic cuvettes (9 cm × 7.5 cm × 1.5 cm) in about 95 mL water and the cuvettes were suspended in a rack centered between and about 10 cm from the bank of four FL bulbs (each side) inside the exposure chamber. FL sources were “cool white” Philips F 20 T12/CW 20 W, Alto (i.e., 4100 K FL) fluorescent lamps provided doses of 35 kJ/m^2^, that equated to an exposure time of about 40 min.

For waveband exposure of *X. maculatus* to 50 nm [350–400 18.6 kJ/m^2^), 400–450 (21.6 kJ/m^2^), 450–500 (21.2 kJ/m^2^), 500–550 (17.4 kJ/m^2^), and 550–600 nm (13.8 kJ/m^2^)] or 10 nm wavebands of light [500–510 (18.5 kJ/m^2^), 510–520 (17.9 kJ/m^2^), 520–530 (17.5 kJ/m^2^), 530–540 (16.9 kJ/m^2^), and 540–550 (16.4 kJ/m^2^), we utilized a TLS-300X Series Tunable Light Source (Newport Corporation, Irvine, CA, USA) containing an Ushio 300 W Xenon Short Arc Lamp Model 6258. Exposures were as detailed previously [20]. Briefly, light emitted from the source was passed through a Cornerstone 130 Monochromator (Newport Corporation, Irvine, CA, USA) to define specific wavelengths. The bulb was burned in 15 min prior to all exposure treatments. The specific wavelengths were divided by two fiber optic light cables, allowing the fish to be exposed on both sides simultaneously to the defined wavelengths of light. Spectral distributions were made to determine the power output of each light source at specific wavelengths using a Newport 1918-R power meter (Newport Corporation, Irvine, CA, USA). The spectral distribution of the xenon light source was measured at full spectrum (0 nm) using an Ocean Optics STS 350–800 nm Microspectrometer (Ocean Optics Inc., Dundedin, FL, USA) and OceanView software v1.5 (http://oceanoptics.com/product/oceanview/). The microspectrometer was calibrated to a known standard using Ocean Optics Halogen Calibrated Light Source HL-3P-CAL (Ocean Optics Inc., Dundedin, FL, USA). To cover each wavelength in each 50 nm region, the monochromator was set to scan and repeat (i.e. loop) using Asoftech Automation (http://www.asoftech.com/) through the wavelengths of each region (1 nm/sec for 50 s) for the duration of the light exposure.

Each fish was then placed in a 4 cm length × 1 cm wide × 4.5 cm height quartz cuvette filled with 14 mL of filtered aquaria water. The cuvette was then centered between the two fiber optic light cables and covered by a cardboard box to eliminate ambient light. After exposure, the fish was removed from the cuvette, rinsed with filtered aquaria water, placed back into a 125 mL flask filled with 100 mL of filtered aquaria water, and in the dark for 6 h to allow for gene expression prior to sacrifice and tissue dissection.

At dissection, fish were anesthetized in an ice bath and upon loss of gill movement were sacrificed by cranial resection. Skin was dissected directly into TRI reagent (Sigma Inc., St Louis, MO, USA) and flash frozen in an ethanol dry ice bath. Remaining tissues were placed in individual 1.5 mL microcentrifuge tubes filled with 300 μL RNA*later* (Life Technologies, Grand Island, NY, USA).

### RNA isolation and sequencing

RNA isolation was performed following the Qiagen RNeasy RNA isolation protocol (Qiagen, Valencia, CA, USA). Skin samples harvested from fish were first homogenized using a hand-held homogenizer in a 1.5 mL microcentrifuge tube while the sample remained frozen in TRI Reagent (Sigma Inc., St Louis, MO, USA). After homogenization, 300 μL of fresh 4 °C TRI Reagent was added to the samples followed by room temperature incubation for 5 min. Chloroform extraction was performed by adding 120 μL chloroform and shaken for 15 s. Samples were centrifuged (16,100 rcf for 5 min at 4 °C) for phase partition. The aqueous layer was transferred to a new 1.5 mL microcentrifuge tube and a second chloroform extraction performed (300 μL TRI Reagent, 60 μL chloroform). After extraction, nucleic acids in the aqueous phase were precipitated with 500 μL 70% EtOH in diethylpyrocarbonate (DEPC) treated water. The sample was then transferred to a Qiagen RNeasy mini spin column and on-column DNase treatment was performed for 15 min at 25 °C. RNA samples were then washed and eluted in 100 μL RNase free water. RNA concentration was measured with a Qubit 2.0 fluorometer (Life Technologies, Grand Island, NY, USA). To further assess the RNA quality, a RNA integrity (RIN) score was determined using an Agilent 2100 Bioanalyzer (Agilent Technologies, Santa Clara, CA, USA). All samples processed for RNA sequencing had a RIN score above 8.

All RNA-seq experiments were performed, independently, on at least, two individual fish. Organs utilized for RNA-seq were from independent animals representing two exposure replicates for each light type. Isolated RNA samples were sent to Beckman Coulter Genomics (Beckman Coulter, Inc., Atlanta, GA) for Illumina High-throughput Sequencing using the Illumina TruSeq mRNA Library Prep Kit on the HiSeq 2000 platform (Illumina, Inc., San Diego, CA, USA). RNA was sequenced (125 bp, paired-end [PE] reads) and the raw reads were trimmed and filtered using a custom Perl script and adapter sequences were removed from the sequencing reads [27]. The reads were truncated based on similarity to library adaptor sequences using custom Perl scripts [27]. Then, low-scoring sections of each read were removed, preserving the longest remaining fragment as previously described [15, 19, 20]. FastQC was then used to assess the quality of the filtered reads to identify any potential deficiencies within the data for each sample (http://www.bioinformatics.babraham.ac.uk/projects/fastqc/).

### Differentially Expressed Gene (DEG) analysis

Genes exhibiting modulated transcriptional expression due to light type or waveband exposures were identified as detailed previously [16–20]. Briefly, the trimmed and filtered reads are mapped to the *Xiphophorus* cDNA reference sequences (Ensembl) [28] using GSNAP [29]. Mapped reads are quantified as raw counts in each file by xEpress. Differentially expressed genes were analyzed using the R-Bioconductor package edgeR [30]. For a gene to be a Differentially Expressed Gene (DEG), it has to alter at least two fold with a False Discovery Rate (FDR) adjusted *p*-value less than 0.05 (Log_2_FC ≥ 1 or Log_2_FC ≤ − 1, FDR < 0.05). Two experimental replicates were utilized for DEG analyses.

Extrinsic experimental parameters that may have arisen during the overnight or post-exposure incubation periods were controlled by identically treated unexposed control, and sham treated, fish replicates. The sham treated fish replicates received identical treatment as the light exposed replicates, but with the light sources turned “off”. Analyses of differentially expressed genes were compared between unexposed control and sham treated fish, to determine those genes altered due to the experimental protocol or handling. Genes showing differential expression in the sham samples relative to the unexposed controls (never more than 5% of the total differentially expressed genes), were removed from comparisons between the light/waveband exposed and unexposed control fish samples prior to functional analyses.

### Functional analysis of differentially expressed genes

Bioinformatics analysis of exposure data was performed using Ingenuity Pathway Analysis (IPA; Qiagen, Redwood City, CA.) for clustering and assessing the biological function of differentially expressed genes, as well as, for inferring up-stream regulators aiding further mechanistic interpretation. *Xiphophorus* Ensemble IDs of DEGs were converted to human homolog gene IDs using Ensemble Biomart. Herein, the term “pathways” is short for canonical pathways as assigned by IPA based on input DEG data. IPA assigns DEGs to pathways if the analysis results in a DEG that has previously been identified within well-established signaling or metabolic pathways based on published literature. Pathway analysis is performed by testing the over-representation of genes belonging to a certain pathway in the input DEGs. Pathways with an enrichment -log_10_(*p-*value) score > 1.3 (*p*-value < 0.05) were kept for further analysis. To predict overall pathway activation or repression, a z-score is calculated by comparing observed differential gene expression and expected gene expression directions. z-score ≥ 2 is used to determine a pathway that is activated, and a z-score ≤ − 2 is used to determine a pathway that is repressed.

### NanoString validation of RNA-Seq

To validate these data, we employed the NanoString nCounter® platform. The nCounter® platform works as a simultaneous gene expression detection system for hundreds of transcripts [21–24]. The technology employs oligonucleotide capture and reporter sequences complementary to each selected light responsive gene, determined from our previous studies, in order to validate modulated genes in the FL, 50 and 10 nm waveband exposures [21]. A custom 200 *Xiphophorus* gene target NanoString panel was utilized to assess the informative gene targets shown in Fig. 8 for each exposure. Fish mRNA is hybridized in solution to gene-specific ‘capture’ and ‘reporter’ probes to form ternary complexes. After hybridization, biotin-labeled capture probes anchor ternary complexes to a slide. Reporter probes may contain 4^7^ (16,384) different fluorescent barcode combinations. Scanning barcoded, immobilized, and electrophoresis extended complexes decodes each hybrid complex to directly count mRNAs without nucleic acid amplification. The gene targets analyzed in this work by NanoString are presented in Additional file 3: Table S3. The raw read counts generated from different nCounter slots were first normalized to built-in positive controls to remove effect of hybridization efficiency differences. Subsequently, the normalized read counts were scaled to the geometric mean of housekeeping genes to remove effect of different amount of RNA loaded into different slots and background noise removed from read counts. Differential expression was assessed by calculating gene expression fold change between light exposed samples to controls.

## Additional files

Additional file 1: **Table S1.** Functional classes determined by IPA were compared for each 50 and 10 nm waveband (z-score ≥ |2|, 5 gene minimum). Unique classes are those that only appear in that waveband and are not shared with any other waveband. Opposite classes are functional classes that were predicted to be up-modulated in one waveband and down modulated in another waveband. A list of all Functional classes predicted by IPA can be found in Additional file 2: Table S2a–k. This table compares and reports both within 50 and 10 nm regions and between 50 and 10 nm regions. (PDF 21 kb)

Additional file 2: **Table S2a–k.** A list of all differentially modulated genes used by IPA enrichment software to predict the direction of change for each functional class represented in Additional file 1: Table S1. Table a is FL, tables b–e are the 50 nm wavebands and tables g–k are the 10 nm wavebands. (ZIP 701 kb)

Additional file 3: **Table S3.** A complete list of all NanoString targets and probe sequences used to verify the RNA-Seq data for each waveband exposure. (ZIP 242 kb)

Additional file 4: **Table S4a–k.** A list of all differentially modulated genes used by IPA enrichment software to predict the direction of change for each functional class represented in Fig. 4. Table a is FL, tables b–e are the 50 nm wavebands and tables g–k are the 10 nm wavebands. (ZIP 262 kb)

Additional file 5: **Table S5a–k.** A list of all differentially modulated genes used by IPA enrichment software to predict the direction of change for each functional class represented in Fig. 6. Table a is FL, tables b–e are the 50 nm wavebands and tables g–k are the 10 nm wavebands. (ZIP 77 kb)

Additional file 6: **Table S6a–d.** A list of all differentially modulated genes used by IPA enrichment software to predict the direction of change for each upstream regulator represented in Fig. 7. Table a is the list for the low waveband exposure, table b is for the low-high exposure, table c is the high exposure and table d is the high-low exposure. (PDF 28 kb)

## Abbreviations

DNA: Deoxyribonucleic acid
EGF: Epidermal growth factor
FC: Fold change
Fdr: False discovery rate
FL: 4100 K fluorescent light, commercially termed “cool-white” fluorescent light
HUGO id: Human gene nomenclature
IPA: Ingenuity Pathway Analysis (IPA; Qiagen, Redwood City, CA.)
kJ/m^2^: kilojoules per square meter
Nm: nanometer
RNA: Ribonucleic acid
RNA-Seq: A technique that uses high-throughput next generation sequencing to reveal the presence and quantity of RNA in a biological sample at a given moment in time
UVA or UVB: Ultraviolet light in the A (315–400 nm) or B (280–315 nm) regions.
Waveband: A 50 nm region of the solar spectrum between 350 and 600 nm (i.e., 350–400 nm, 400–450 nm, etc.), or a 10 nm region of the solar spectrum between 500 and 550 nm (i.e., 500–510 nm, 510–520 nm, etc.)
