# Supplementary material for: Waveband specific transcriptional control of select genetic pathways in vertebrate skin (Xiphophorus maculatus)
Source: BMC Genomics. 2018 May 10;19:355. doi: 10.1186/s12864-018-4735-5 (PMC5946439; doi:10.1186/s12864-018-4735-5)
Supplement: Supplementary file 1 — Table S1. Functional classes determined by IPA were compared for each 50 and 10 nm waveband (z-score ≥ |2|, 5 gene minimum). Unique classes are those that only appear in that waveband and are not shared with any other waveband. Opposite classes are functional classes that were predicted to be up-modulated in one waveband and down modulated in another waveband. A list of all Functional classes predicted by IPA can be found in Additional file 2: Table S2a–k. This table compares and reports both within 50 and 10 nm regions and between 50 and 10 nm regions. (PDF 21 kb) [file 12864_2018_4735_MOESM1_ESM.pdf]

Waveband (nm)	Total	Unique	Opposite	List of opposite (gene number)	Shared with FL
FL	74 (391)	48	6	Organismal death (128), cell death (172), necrosis (108), apoptosis (135), cell viability (78), cell proliferation (176)	
350-400	24 (64)	10 (32)	9	Cell death (8), Organismal death (37), apoptosis (16), necrosis (33), inflammation (24), connective tissue differentiation (24), connective tissue cells differentiation (13), ingestion (8), cell viability (22)	6
400-450	3 (5)	0	2	Cell Death (5), cell viability (5)	2
450-500	136 (72)	105 (68)	18	Cell Death (41), quantity of cells (27), cell migration (25), generation of cells (21), cell differentiation (24), vasculogenesis (14), angiogenesis (19), organismal death (32), body trunk development (17), stress response of cells (6), smooth cell migration (6), adhesion of connective tissue cells (5), apoptosis (34), necrosis (37), inflammation (19), abdomen development (10), cell proliferation (40), cell viability (54), – promotion of cell viability	6
500-550	28 (197)	16 (106)	5	Body trunk development (30), transcription of cells (33), hypertrophy (17), fibrosis (8), inflammation (8)	8
550-600	41 (165)	5 (21)	9	Quantity of cells (36), cell migration (45), generation of cells (44), cell differentiation (44), vasculogenesis (22), angiogenesis (27), inflammation (11), cell proliferation (72), organismal death (49) – suppression of cell viability	7
500-510	35 (184)	14 (108)	5	Benign neoplasia (23), organismal death (54), connective tissue cells differentiation (9), connective tissue differentiation (20), differentiation of cells (51)	3
510-520	134 (669)	74 (223)	14	Benign neoplasia (81), organismal death (177), cell death (214), apoptosis (183), necrosis (166), transcription of cells (128), smooth cell migration (14), abdomen development (43), cell migration (135), body trunk development (81), quantity of cells (114), cell proliferation (72), cell viability (176), cell differentiation (176)	10
520-530	102 (349)	40 (92)	15	Quantity of connective tissue cells (15), benign neoplasia (53), quantity of blood cells (41), quantity of cells (74), cell viability (53), cell proliferation (47), stress response of cells (9), cell differentiation (100), vasculogenesis (36), hypertrophy (20), fibrosis (25), necrosis (94), apoptosis (106), cell death (125), organismal death (100)	5
530-540	57 (235)	14 (33)	8	Quantity of connective tissue cells (12), benign neoplasia (42), quantity of blood cells (27), adhesion of connective tissue cells (8), cell migration (49), cell differentiation (63), fibrosis (18), organismal death (64)	4
540-550	11 (141)	3 (16)	2	Ingestion (6), organismal death (38),	2
