# Supplementary material for: Waveband specific transcriptional control of select genetic pathways in vertebrate skin (Xiphophorus maculatus)
Source: BMC Genomics. 2018 May 10;19:355. doi: 10.1186/s12864-018-4735-5 (PMC5946439; doi:10.1186/s12864-018-4735-5)
Supplement: Supplementary file 2 — Table S2a–k. A list of all differentially modulated genes used by IPA enrichment software to predict the direction of change for each functional class represented in Additional file 1: Table S1. Table a is FL, tables b–e are the 50 nm wavebands and tables g–k are the 10 nm wavebands. (ZIP 701 kb) [file 12864_2018_4735_MOESM2_ESM.zip › TableS2k_540-550nm.pdf]

Functional Class	p-Value	Activation	# Genes	Genes					
ingestion	5.98E-03	-2.34	6	ATM	GHR	MSTN	PER1	SLC14A1	SLC14A2
size of body	7.70E-05	-2.61	21	AEBP1	ATM	ATRN	CDON	COL10A1	COL2A1
concentration of lipid	2.24E-03	-2.53	17	ACSBG1	AEBP1	ALOX12B	ALOXE3	ANXA5	AQP12A/A(
quantity of adipose tissue	3.01E-03	-2.26	8	AEBP1	ATM	DPT	GHR	LPL	MSTN
production of cells	5.69E-03	-2.00	5	ATM	CSF1R	GATA3	MSTN	RBL1	
congenital anomaly of musculoskeletal system	7.82E-06	2.05	21	ADAMTS1(	ADAMTS2	ATR	BMPR1B	CDON	CDT1
development of connective tissue	1.94E-03	2.13	10	BMPR1B	COL11A1	COL2A1	CSF1R	CYP1A1	CYP1A2
cell movement of embryonic cell lines	1.36E-03	2.19	5	COL18A1	CTSE	CXCL12	PLXNB1	POSTN	
hearing loss	1.60E-03	3.19	7	COL1A1	COL2A1	CSF1R	OCM	OTOF	SLC17A8
fatty acid oxidation	1.68E-06	2.19	6	GHR	IGFBP5	LPL	MSTN	RBL1	SIK3
organismal death	2.65E-03	2.65	38	AEBP1	AGRN	ALOX12B	ALOXE3	ATM	ATR

COL5A2 CSF1R ENTPD5 GHR IGFBP5 MMP13 MSTN PER1 POSTN RBL1 SIK3 SLC14A1 SLC14A2
COL18A1 CXCL12 CYP1A1 CYP1A2 EEF1A1 ENTPD5 GHR LPL MSTN SIK3 TTR
RBL1 SIK3

COL10A1 COL11A1 COL1A1 COL1A2 COL2A1 COL5A1 COL5A2 COL6A1 COL9A3 GHR LPL MMP13 MSTN
JDP2 MMP13 PER1 SLC14A1

TNC

CDC45 CDON COL10A1 COL11A1 COL1A1 COL2A1 COL5A1 COL5A2 COL7A1 CSF1R CXCL12 CYP1A1 CYP1A2

STC2 XIRP1

PIEZO2 POLE

DOT1L GATA3 GHR GPHN IGFBP5 LPL MCM10 MCM2 MNX1 MSTN POSTN RBL1 RPL24

SALL3	SEMA5A	SIK3	SLC14A1	SUZ12	TRRAP
