# Supplementary material for: Waveband specific transcriptional control of select genetic pathways in vertebrate skin (Xiphophorus maculatus)
Source: BMC Genomics. 2018 May 10;19:355. doi: 10.1186/s12864-018-4735-5 (PMC5946439; doi:10.1186/s12864-018-4735-5)
Supplement: Supplementary file 2 — Table S2a–k. A list of all differentially modulated genes used by IPA enrichment software to predict the direction of change for each functional class represented in Additional file 1: Table S1. Table a is FL, tables b–e are the 50 nm wavebands and tables g–k are the 10 nm wavebands. (ZIP 701 kb) [file 12864_2018_4735_MOESM2_ESM.zip › TableS2j_530-540nm.pdf]

Functional Class	p-Value	Activation	# Genes	Genes															
development of gap junctior	1.49E-03	-3.59	10	ADGRL2	AGRN	CHRNB2	EPHB3	GPM6A	PANX3	SLITRK3	SRPX2	THBS2	TNC						
developmental process of s	3.23E-03	-3.40	9	ADGRL2	AGRN	CHRNB2	EPHB3	GPM6A	SLITRK3	SRPX2	THBS2	TNC							
cell movement	6.90E-04	-3.37	54	ADAM8	ADORA1	ALOX15B	ANPEP	ANXA5	AQP3	ATM	C6	CAPN1	CELSR2	COL11A1	COL18A1	COL1A1	COL2A1	COL7A1	CSF1R
size of body	1.36E-05	-3.33	31	AEBP1	ATM	ATRN	CDON	CERK	COL10A1	COL11A2	COL2A1	COL5A2	COL5A3	CSF1R	ENTPD5	F3	FAT4	GFRA2	HMOX1
association of plasma mem	1.80E-03	-3.18	11	ADGRL2	AGRN	CHRNB2	CSF1R	EPHB3	GPM6A	PANX3	SLITRK3	SRPX2	THBS2	TNC					
differentiation of cells	1.18E-05	-2.80	63	ADAM8	ADORA1	AGRN	ALAS2	ALOX15B	ALOXE3	ANPEP	AQP3	ATM	BRD8	CA2	CDON	CHRNB2	CNTFR	COL11A2	COL18A1
obesity	6.21E-04	-2.66	17	ADORA1	AEBP1	ATRN	C1QTNF1	CA1	CA13	CA2	CA3	COL5A3	COX6A2	HSD11B2	MSTN	NR4A1	SCN4A	SIK3	TNMD
adhesion of fibroblasts	1.86E-05	-2.63	6	COL2A1	COL7A1	POSTN	THBS2	TNC	TNMD										
dna repair	1.47E-08	-2.59	15	ADGRL2	AGRN	ATM	CHRNB2	DNMT3B	EPHB3	GPM6A	HMOX1	MMS22L	PANX3	SLITRK3	SRPX2	THBS2	TNC	TONSL	
adhesion of connective tiss	1.18E-03	-2.57	8	COL1A1	COL2A1	COL7A1	POSTN	TGFBI	THBS2	TNC	TNMD								
migration of cells	8.74E-04	-2.48	49	ADAM8	ADORA1	ALOX15B	ANPEP	ANXA5	AQP3	ATM	C6	CELSR2	COL11A1	COL18A1	COL1A1	COL7A1	CSF1R	CTSE	CYP1A1
invasion of tumor cell lines	8.16E-03	-2.39	19	ADAM8	CA3	COL11A1	COL18A1	DNMT3B	ENTPD5	EPHB4	GRB7	HMOX1	LAMB3	MST1R	PLCD1	PLXNB1	POSTN	SATB2	SUZ12
chromosomal alignment/mc	3.03E-05	-2.37	54	ADAM8	ADORA1	ALOX15B	ANPEP	ANXA5	AQP3	ATM	C6	CAPN1	CELSR2	COL11A1	COL18A1	COL1A1	COL2A1	COL7A1	CSF1R
injury of mice	8.89E-04	-2.34	6	C6	CAPN8	COL1A1	F3	MGAT5B	POSTN										
DNA replication	6.33E-03	-2.32	35	ADORA1	AGRN	ANXA5	AQP3	ATM	ATR	CA2	CERK	CNTFR	COL18A1	COL1A1	CSF1R	EPHB3	EPHB4	FA2H	GATA1
branching of endothelial cel	2.22E-03	-2.23	5	COL18A1	EPHB3	EPHB4	SRPX2	TNC											
branching of epithelial tissu	4.93E-03	-2.23	6	COL18A1	EPHB3	EPHB4	SEMA5A	SRPX2	TNC										
length of body region	6.96E-06	-2.20	8	COL1A2	COL2A1	CYP1A1	CYP1A2	EPHB3	HSD11B2	POSTN	WASF1								
movement of vascular endc	3.08E-03	-2.18	8	ANPEP	COL18A1	GATA1	HSPA5	NR4A1	TGFBI	THBS2	WNK1								
formation of muscle	6.27E-03	-2.11	13	AEBP1	ANXA5	CDON	COL11A1	COL25A1	COL6A3	HSP90B1	MNX1	MSTN	MYOZ1	NEURL2	PLXNB1	WT1			
morphogenesis of endothel	1.86E-04	-2.07	5	ANPEP	COL18A1	COL1A1	EPHB4	TNMD											
morphogenesis of epithelial	4.37E-03	-2.07	7	ANPEP	CA2	COL18A1	COL1A1	EPHB4	SEMA5A	TNMD									
morphogenesis of cardiova:	7.78E-03	-2.07	9	ANPEP	COL11A1	COL18A1	COL1A1	COL2A1	COL5A1	EPHB4	FAT4	TNMD							
adhesion of embryonic cells	4.81E-04	-2.00	5	COL2A1	EPHB3	PLXNB1	POSTN	TNMD											
Infarction	3.96E-03	2.04	12	ADAM8	ADORA1	ANXA5	C6	CSF1R	CYP1A2	F3	FSTL3	HMOX1	POSTN	PTX3	TUBB1				
congenital anomaly of mout	5.93E-03	2.07	7	CDON	COL2A1	DNMT3B	EPHB3	LAMB3	MSTN	SATB2									
perinatal death	9.24E-04	2.10	20	AEBP1	AGRN	ALOX12B	ALOXE3	ATM	CDON	CNTFR	COL2A1	COL5A2	CYP1A2	F3	FAT4	HMOX1	MBTD1	MNX1	MSTN
Bleeding	1.79E-03	2.11	15	ADORA1	CAPN1	CDON	CHRNB2	COL18A1	COL1A1	COL2A1	DNMT3B	F3	GATA1	PTX3	SCN4A	THBS2	TUBB1	WT1	
abdominal cancer	1.13E-09	2.11	194	AATK	ABCC5	ABI3BP	ADAM8	ADAMTS1C	ADAMTS2	ADAMTSL1	ADGRL2	ADORA1	AEBP1	AGRN	ALAS2	ALOX15B	ANK1	ANO7	ANPEP
quantity of connective tissu	1.19E-04	2.17	12	ATM	CAPN1	COL1A1	DNMT3B	FSTL3	GATA1	HSD11B2	PER1	PER2	POSTN	SIK3	WT1				
benign neoplasia	7.25E-10	2.17	42	AEBP1	AGRN	ANXA5	ATM	ATR	ATRN	CA1	CA2	COL10A1	COL11A1	COL11A2	COL15A1	COL16A1	COL18A1	COL1A1	COL1A2
epithelial cancer	2.35E-10	2.18	197	AATK	ABCC5	ABI3BP	ADAM8	ADAMTS1C	ADAMTS2	ADAMTSL1	ADGRL2	ADORA1	AEBP1	AGRN	ALAS2	ALOX12B	ALOX15B	ANK1	ANO7
mammary tumor	2.28E-06	2.21	52	ABCC5	ADAMTS1C	ADAMTS2	ADGRL2	AGRN	ALOX12B	AQP12A/A(	ATM	ATR	COL1A1	COL1A2	COL5A3	CSF1R	CYP1A1	CYP1A2	DENND4B
congenital anomaly of musc	5.86E-04	2.26	24	ADAMTS1C	ADAMTS2	ATR	CDON	COL10A1	COL11A1	COL11A2	COL1A1	COL1A2	COL2A1	COL5A1	COL5A2	COL6A3	DNMT3B	EPHB3	FAT4
quantity of blood cells	7.88E-03	2.32	27	ADAM8	ANPEP	ATM	C6	CERK	COL10A1	CSF1R	CTSE	CYP1A1	F3	GATA1	HBB	HBZ	HMOX1	HPX	HSP90B1
neoplasia of epithelial tissu	3.10E-10	2.32	198	AATK	ABCC5	ABI3BP	ADAM8	ADAMTS1C	ADAMTS2	ADAMTSL1	ADGRL2	ADORA1	AEBP1	AGRN	ALAS2	ALOX12B	ALOX15B	ANK1	ANO7
tumorigenesis of tissue	1.48E-10	2.32	201	AATK	ABCC5	ABI3BP	ADAM8	ADAMTS1C	ADAMTS2	ADAMTSL1	ADGRL2	ADORA1	AEBP1	AGRN	ALAS2	ALOX12B	ALOX15B	ANK1	ANO7
fibrosis	1.39E-03	2.34	18	ADORA1	COL1A1	COL1A2	CSF1R	CYP1A2	F3	FSTL3	HBB	HMOX1	HPX	LRRC15	MMP13	MSTN	POSTN	PTX3	SLC4A1
fibrosis of heart	3.17E-03	2.37	8	ADORA1	F3	FSTL3	HMOX1	POSTN	PTX3	SLC4A1	THBS2								
weight gain	1.85E-03	2.48	12	C1QTNF1	CA1	CA13	CA2	CA3	CYP1A1	CYP1A2	MFN1	MSTN	NR4A1	PER2	SLC4A1				
abdominal neoplasm	1.93E-09	2.51	195	AATK	ABCC5	ABI3BP	ADAM8	ADAMTS1C	ADAMTS2	ADAMTSL1	ADGRL2	ADORA1	AEBP1	AGRN	ALAS2	ALOX15B	ANK1	ANO7	ANPEP
loss of neurons	5.26E-03	2.54	7	ANXA5	ATM	CHRNB2	CNTFR	COL25A1	GFRA2	TTR									
invasion of tumor	6.98E-03	2.54	9	COL7A1	F3	HMOX1	HSPA5	LRRC15	MST1R	POSTN	SATB2	WT1							
vascularization of body regi	7.98E-03	2.54	6	ANPEP	C6	COL18A1	F3	THBS2	WT1										
accumulation of myeloid ce	9.02E-03	2.67	7	C6	COL18A1	F3	HMOX1	NR4A1	POSTN	PTX3									
digestive organ tumor	2.34E-10	2.94	178	AATK	ABCC5	ABI3BP	ADAMTS1C	ADAMTS2	ADAMTSL1	ADGRL2	ADORA1	AEBP1	AGRN	ALAS2	ALOX15B	ANK1	ANO7	ANPEP	AQP12A/A(
hematologic cancer	3.36E-04	2.96	50	ADGRL2	ADORA1	ALOX12B	ANK1	ANXA5	ATM	ATR	ATRN	CELSR2	CNTFR	COL16A1	COL1A1	COL1A2	COL27A1	COL2A1	COL5A2
liver tumor	5.84E-08	2.98	114	AATK	ABCC5	ABI3BP	ADAMTS1C	ADAMTS2	ADAMTSL1	AEBP1	AGRN	ANK1	ANO7	ANPEP	AQP12A/A(	ARNTL2	ASB9	ATAD2	ATM
digestive system cancer	2.02E-10	3.00	177	AATK	ABCC5	ABI3BP	ADAMTS1C	ADAMTS2	ADAMTSL1	ADGRL2	ADORA1	AEBP1	AGRN	ALAS2	ALOX15B	ANK1	ANO7	ANPEP	AQP12A/A(
vascularization	1.71E-03	3.17	10	ANPEP	C6	COL18A1	EPHB3	F3	MMP13	MST1R	PLCD1	THBS2	WT1						
Lymphoid Cancer and Tum	1.84E-04	3.19	49	ADGRL2	ADORA1	ANK1	ATM	ATR	CELSR2	COL16A1	COL18A1	COL1A1	COL1A2	COL27A1	COL2A1	COL5A2	COL6A3	COL7A1	CSF1R
hematological neoplasia	4.20E-04	3.19	51	ADGRL2	ADORA1	ALOX12B	ANK1	ANXA5	ATM	ATR	ATRN	CELSR2	CNTFR	COL16A1	COL1A1	COL1A2	COL27A1	COL2A1	COL5A2
lymphohematopoietic cance	6.61E-05	3.19	53	ADGRL2	ADORA1	ALOX12B	ANK1	ANXA5	ATM	ATR	ATRN	CELSR2	CNTFR	COL16A1	COL18A1	COL1A1	COL1A2	COL27A1	COL2A1
lymphoid cancer	1.42E-04	3.19	48	ADGRL2	ADORA1	ANK1	ATM	ATR	CELSR2	COL16A1	COL18A1	COL1A1	COL1A2	COL27A1	COL2A1	COL5A2	COL6A3	COL7A1	CSF1R
hearing loss	1.91E-03	3.20	9	COL11A2	COL1A1	COL2A1	CSF1R	OCM	OTOF	SLC52A3	TNC	TSPEAR							
organismal death	4.37E-05	3.35	64	ADORA1	AEBP1	AGRN	ALAS2	ALOX12B	ALOXE3	ATM	ATR	CAPN1	CDC45	CDON	CERK	CHRNB2	CNTFR	COL10A1	COL11A1
lymphohematopoietic neopl	8.26E-05	3.40	54	ADGRL2	ADORA1	ALOX12B	ANK1	ANXA5	ATM	ATR	ATRN	CELSR2	CNTFR	COL16A1	COL18A1	COL1A1	COL1A2	COL27A1	COL2A1

CTSE	CYP1A1	CYP1A2	EPHB3	EPHB4	F3	GAPDH	GATA1	GPM6A	GRB7	HMOX1	HSP90B1	HSPA5	IGSF8	LAMB1	LAMB3	LRRC15	MCM2	MMP13	MNX1	MST1R
MMP13	MSTN	MYOZ1	NEURL2	PER1	PER2	PLCD1	POSTN	RHCG	SIK3	SLC14A1	SLC14A2	SLC4A1	TGFBI	WASF1						
COL25A1	COL2A1	CSF1R	CTSE	CYB5D2	CYTL1	DNMT3B	DOT1L	EPHB3	EPHB4	EXTL1	FSTL3	GAPDH	GATA1	GPM6A	HMOX1	HPX	HSP90B1	HSPA5	JARID2	JDP2
WT1																				
CYP1A2	EPHB3	EPHB4	F3	GATA1	GPM6A	GRB7	HMOX1	HSP90B1	HSPA5	IGSF8	LAMB1	LAMB3	LRRC15	MCM2	MMP13	MNX1	MST1R	NR4A1	PER1	PLCD1
TGFBI	TNC	WBP1L																		
CTSE	CYP1A1	CYP1A2	EPHB3	EPHB4	F3	GAPDH	GATA1	GPM6A	GRB7	HMOX1	HSP90B1	HSPA5	IGSF8	LAMB1	LAMB3	LRRC15	MCM2	MMP13	MNX1	MST1R
GFRA2	HBB	HBZ	HMOX1	HSP90B1	HSPA5	JDP2	MEFV	METAP2	MMP13	MMS22L	MST1R	NR4A1	PLXNB1	POSTN	SLC25A23	THBS2	TONSL	WT1		
SALL3	SATB2	SIK3	WT1																	
ANXA5	AQP12A/A(	AQP3	ARNTL2	ASB9	ATAD2	ATM	ATR	ATRN	BRD8	C1QTNF1	C6	CA1	CA2	CAMSAP2	CAPN1	CAPN8	CCDC169	CDC45	CDON	CELSR2
COL21A1	COL22A1	COL25A1	COL27A1	COL2A1	COL4A6	COL5A1	COL5A2	COL5A3	COL6A3	COL7A1	COX6A2	CSF1R	DNMT3B	ENTPD5	EPHB3	FKBP10	GPM6A	HMOX1	HSD11B2	LAMB3
ANPEP	ANXA5	AQP12A/A(	ARNTL2	ASB9	ATAD2	ATM	ATR	ATRN	BRD8	C1QTNF1	C6	CA1	CA2	CA3	CAMSAP2	CAPN1	CAPN8	CCDC169	CDC45	CDON
DNAH7	DOT1L	FSTL3	GATA1	GRB7	HBB	HMOX1	HSP90B1	HSPA5	IGSF8	KLF11	LAMB1	LAMB3	LRRC15	MAMDC2	MARK4	MCM10	MCM2	METAP2	MGAT5B	MMP13
HSP90B1	HSPA5	MBTD1	MMP13	MSTN	PIEZO2	SATB2	TGFBI													
JARID2	MBTD1	MGAT5B	MMP13	NR4A1	PER2	PLCD1	SLC14A1	SLC4A1	TGFBI	THBS2										
ANPEP	ANXA5	AQP12A/A(	ARNTL2	ASB9	ATAD2	ATM	ATR	ATRN	BRD8	C1QTNF1	C6	CA1	CA2	CA3	CAMSAP2	CAPN1	CAPN8	CCDC169	CDC45	CDON
ANPEP	ANXA5	AQP12A/A(	ARNTL2	ASB9	ATAD2	ATM	ATR	ATRN	BRD8	C1QTNF1	C6	CA1	CA2	CA3	CAMSAP2	CAPN1	CAPN8	CCDC169	CDC45	CDON
THBS2	WT1																			
ANXA5	AQP12A/A(	AQP3	ARNTL2	ASB9	ATAD2	ATM	ATR	ATRN	BRD8	C1QTNF1	C6	CA1	CA2	CAMSAP2	CAPN1	CAPN8	CCDC169	CDC45	CDON	CELSR2
AQP3	ARNTL2	ASB9	ATAD2	ATM	ATR	ATRN	BRD8	C1QTNF1	C6	CA1	CA2	CAMSAP2	CAPN1	CAPN8	CDC45	CDON	CELSR2	CNTFR	COL10A1	COL11A1
COL6A3	COL7A1	CSF1R	CYP1A1	DNAH7	DNMT3B	EPHB3	EPHB4	F3	FAM161A	FAT4	GATA1	GPR1	HERC1	HMOX1	HSPA5	JARID2	MFN1	MST1R	MTMR11	PER2
ATR	ATRN	C1QTNF1	CA2	CAPN8	CDC45	CELSR2	COL10A1	COL11A1	COL11A2	COL16A1	COL18A1	COL1A1	COL1A2	COL21A1	COL22A1	COL27A1	COL2A1	COL4A6	COL5A1	COL5A2
AQP3	ARNTL2	ASB9	ATAD2	ATM	ATR	ATRN	BRD8	C1QTNF1	C6	CA1	CA2	CAMSAP2	CAPN1	CAPN8	CDC45	CDON	CELSR2	CNTFR	COL10A1	COL11A1
CYP1A1	DNAH7	DNMT3B	EPHB3	EPHB4	F3	FAM161A	FAT4	GATA1	GPR1	HERC1	HSP90B1	HSPA5	JARID2	LAMB3	MFN1	MST1R	MTMR11	PER2	PER3	RFPL4A/RF
COL6A3	COL7A1	CSF1R	CYP1A1	DNAH7	DNMT3B	EPHB3	EPHB4	F3	FAM161A	FAT4	GATA1	GPR1	HERC1	HMOX1	HSPA5	JARID2	MFN1	MST1R	MTMR11	PER2
COL5A2	COL6A3	COL7A1	CSF1R	CYP1A1	DNAH7	DNMT3B	EPHB3	EPHB4	F3	FAM161A	FAT4	GATA1	GPR1	HERC1	HMOX1	HSP90B1	HSPA5	JARID2	LAMB3	MFN1
CYP1A1	DNAH7	DNMT3B	EPHB3	EPHB4	F3	FAM161A	FAT4	GATA1	GPR1	HERC1	HSP90B1	HSPA5	JARID2	LAMB3	MFN1	MST1R	MTMR11	PER2	PER3	RFPL4A/RF
COL1A1	COL25A1	COL2A1	COL5A1	COL5A2	COL7A1	CSF1R	CYP1A1	CYP1A2	DNMT3B	DOT1L	EPHB3	F3	FAT4	GATA1	HBZ	HMOX1	HSD11B2	HSP90B1	HSPA5	KLF1
COL5A2	COL6A3	COL7A1	CSF1R	CYP1A1	DNAH7	DNMT3B	EPHB3	EPHB4	F3	FAM161A	FAT4	GATA1	GPR1	HERC1	HMOX1	HSP90B1	HSPA5	JARID2	LAMB3	MFN1

NR4A1	PER1	PLCD1	PLXNB1	POSTN	PTX3	SATB2	SEMA5A	SRPX2	SUZ12	TGFBI	THBS2	TNC	UNC5C	WASF1	WNK1	WT1				
KLF1	LAMB3	MFN1	MGAT5B	MMP13	MNX1	MST1R	MSTN	NR4A1	OCSTAMP	OGN	PANX3	PER3	PLCD1	POSTN	RORB	SALL3	SATB2	SEMA5A	SIK3	SRPX2
PLXNB1	POSTN	PTX3	SATB2	SEMA5A	SRPX2	SUZ12	TGFBI	THBS2	TNC	UNC5C	WASF1									
NR4A1	PER1	PLCD1	PLXNB1	POSTN	PTX3	SATB2	SEMA5A	SRPX2	SUZ12	TGFBI	THBS2	TNC	UNC5C	WASF1	WNK1	WT1				
CHRNB2	CNTFR	COL10A1	COL11A1	COL11A2	COL15A1	COL16A1	COL18A1	COL1A1	COL1A2	COL21A1	COL22A1	COL25A1	COL27A1	COL28A1	COL2A1	COL4A6	COL5A1	COL5A2	COL5A3	COL6A3
MST1R	NR4A1	RORB	THBS2	TNC																
CELSR2	CHRNB2	CNTFR	COL10A1	COL11A1	COL11A2	COL15A1	COL16A1	COL18A1	COL1A1	COL1A2	COL21A1	COL22A1	COL25A1	COL27A1	COL28A1	COL2A1	COL4A6	COL5A1	COL5A2	COL5A3
MRC1	MST1R	NR4A1	OCSTAMP	OTOF	PER3	PHOSPHO	POSTN	TGFBI	TNC	TTR	TUBB1	WT1	YBX2	ZAN						
CELSR2	CHRNB2	CNTFR	COL10A1	COL11A1	COL11A2	COL15A1	COL16A1	COL18A1	COL1A1	COL1A2	COL21A1	COL22A1	COL25A1	COL27A1	COL28A1	COL2A1	COL4A6	COL5A1	COL5A2	COL5A3
CELSR2	CHRNB2	CNTFR	COL10A1	COL11A1	COL11A2	COL15A1	COL16A1	COL18A1	COL1A1	COL1A2	COL21A1	COL22A1	COL25A1	COL27A1	COL28A1	COL2A1	COL4A6	COL5A1	COL5A2	COL5A3
CHRNB2	CNTFR	COL10A1	COL11A1	COL11A2	COL15A1	COL16A1	COL18A1	COL1A1	COL1A2	COL21A1	COL22A1	COL25A1	COL27A1	COL28A1	COL2A1	COL4A6	COL5A1	COL5A2	COL5A3	COL6A3
COL11A2	COL15A1	COL16A1	COL18A1	COL1A1	COL1A2	COL21A1	COL22A1	COL25A1	COL27A1	COL2A1	COL4A6	COL5A1	COL5A2	COL5A3	COL6A3	COL7A1	CPXM2	CSF1R	CTSE	CYP1A1
PER3	RFPL4A/RF	RORB	SALL3	SEMA5A	SRPX	SUZ12	SVEP1	TGFBI	THBS2	TUBB1	WT1	ZAN								
COL6A3	COL7A1	CPXM2	CSF1R	CYTL1	DNAH7	DOT1L	ENTPD5	F3	FAM150B	FAT4	FBRSL1	FKBP10	FMO2	FSTL3	GPM6A	HBB	HERC1	HMCN1	HMOX1	HPX
COL11A2	COL15A1	COL16A1	COL18A1	COL1A1	COL1A2	COL21A1	COL22A1	COL25A1	COL27A1	COL2A1	COL4A6	COL5A1	COL5A2	COL5A3	COL6A3	COL7A1	CPXM2	CSF1R	CTSE	CYP1A1
RORB	RPS20	SALL3	SEMA5A	SRPX	SUZ12	SVEP1	TGFBI	THBS2	TUBB1	WT1	ZAN									
PER3	RFPL4A/RF	RORB	RPS20	SALL3	SEMA5A	SRPX	SUZ12	SVEP1	TGFBI	THBS2	TUBB1	WT1	ZAN							
MST1R	MTMR11	PER2	PER3	RFPL4A/RF	RORB	SALL3	SEMA5A	SRPX	SUZ12	SVEP1	TGFBI	THBS2	TUBB1	WT1	ZAN					
RORB	SALL3	SEMA5A	SRPX	SUZ12	SVEP1	TGFBI	THBS2	TUBB1	WT1	ZAN										
MBTD1	MCM10	MCM2	MCM3AP	METAP2	MFN1	MNX1	MRC1	MST1R	MSTN	PER2	PLCD1	POSTN	PTX3	RPL24	SALL3	SATB2	SEMA5A	SIK3	SLC14A1	SLC4A1
MST1R	MTMR11	PER2	PER3	RFPL4A/RF	RORB	RPS20	SALL3	SEMA5A	SRPX	SUZ12	SVEP1	TGFBI	THBS2	TUBB1	WT1	ZAN				

SUZ12	THBS2	TMBIM1	TNC	WT1																
COL7A1	CPXM2	CSF1R	CTSE	CYP1A1	CYP1A2	CYTL1	DENND4B	DNAH7	DNMT3B	DOT1L	ENTPD5	EPHB4	F3	FA2H	FAM150B	FAM161A	FAM46A	FAT4	FBRSL1	FKBP10
COL6A3	COL7A1	COX6A2	CPXM2	CSF1R	CTSE	CYP1A1	CYP1A2	CYTL1	DENND4B	DNAH7	DNMT3B	DOT1L	ENTPD5	EPHB4	F3	FA2H	FAM150B	FAM161A	FAM46A	FAT4
COL6A3	COL7A1	COX6A2	CPXM2	CSF1R	CTSE	CYP1A1	CYP1A2	CYTL1	DENND4B	DNAH7	DNMT3B	DOT1L	ENTPD5	EPHB3	EPHB4	F3	FA2H	FAM150B	FAM161A	FAM46A
COL6A3	COL7A1	COX6A2	CPXM2	CSF1R	CTSE	CYP1A1	CYP1A2	CYTL1	DENND4B	DNAH7	DNMT3B	DOT1L	ENTPD5	EPHB3	EPHB4	F3	FA2H	FAM150B	FAM161A	FAM46A
COL7A1	CPXM2	CSF1R	CTSE	CYP1A1	CYP1A2	CYTL1	DENND4B	DNAH7	DNMT3B	DOT1L	ENTPD5	EPHB3	EPHB4	F3	FA2H	FAM150B	FAM161A	FAM46A	FAT4	FBRSL1
CYP1A2	CYTL1	DENND4B	DNAH7	DNMT3B	DOT1L	ENTPD5	EPHB3	EPHB4	F3	FA2H	FAM150B	FAM46A	FAT4	FBRSL1	FKBP10	FMO2	FNDC1	FSTL3	GFRA2	GPM6A
HSD11B2	HSPA5	IGSF10	IGSF8	JARID2	JDP2	KIAA0556	KIAA1109	LAMB1	LAMB3	LIMCH1	MARK4	MCM3AP	MEGF6	MGAT5B	MMP13	MMS22L	MST1R	MTMR11	MYO7B	NR4A1
CYP1A2	CYTL1	DENND4B	DNAH7	DNMT3B	DOT1L	ENTPD5	EPHB4	F3	FA2H	FAM150B	FAM46A	FAT4	FBRSL1	FKBP10	FMO2	FNDC1	FSTL3	GFRA2	GPM6A	HBB
SUZ12	TGFBI	THBS2	TRRAP	WASF1	WT1															

FMO2	FMO3	FMO4	FNDC1	FSTL3	GAPDH	GFRA2	GPM6A	GRB7	HBB	HBD	HBE1	HERC1	HMCN1	HMOX1	HPX	HSD11B2	HSP90B1	HSPA12B	HSPA5	IGSF10
FBRSL1	FKBP10	FMO2	FMO3	FMO4	FNDC1	FSTL3	GFRA2	GPM6A	GRB7	HBB	HBD	HBE1	HERC1	HMCN1	HMOX1	HPX	HSD11B2	HSP90B1	HSPA12B	HSPA5
FAT4	FBRSL1	FKBP10	FMO2	FMO3	FMO4	FNDC1	FSTL3	GFRA2	GPM6A	GRB7	HBB	HBD	HBE1	HERC1	HMCN1	HMOX1	HPX	HSD11B2	HSP90B1	HSPA12B
FAT4	FBRSL1	FKBP10	FMO2	FMO3	FMO4	FNDC1	FSTL3	GATA1	GFRA2	GPM6A	GPR1	GRB7	HBB	HBD	HBE1	HERC1	HMCN1	HMOX1	HPX	HSD11B2
FKBP10	FMO2	FMO3	FMO4	FNDC1	FSTL3	GAPDH	GFRA2	GPM6A	GRB7	HBB	HBD	HBE1	HERC1	HMCN1	HMOX1	HPX	HSD11B2	HSP90B1	HSPA12B	HSPA5
HBB	HERC1	HMCN1	HMOX1	HPX	HSD11B2	HSP90B1	HSPA12B	HSPA5	IGSF10	IGSF8	JARID2	JDP2	KIAA0556	KIAA1109	KLF1	KLF11	LAMB1	LAMB3	LAMB4	LIMCH1
NUP205	OBSL1	OCSTAMP	OTOF	P3H3	PANX3	PER1	PER3	PLXNB1	POSTN	PTPRB	PTX3	RAP1GAP2	RFPL1/RFF	SALL3	SHTN1	SLC25A41	SLC4A1	SLC4A3	SLITRK3	SRPX
HERC1	HMCN1	HMOX1	HPX	HSD11B2	HSP90B1	HSPA12B	HSPA5	IGSF10	IGSF8	JARID2	JDP2	KIAA0556	KIAA1109	KLF1	KLF11	LAMB1	LAMB3	LAMB4	LIMCH1	LRRC15

IGSF8	JARID2	JDP2	KIAA0556	KIAA1109	KLF1	KLF11	LAMB1	LAMB3	LAMB4	LIMCH1	LRRC15	MARK4	MBTD1	MCM10	MCM2	MCM3AP	MEFV	MEGF6	METAP2	MFN1
IGSF10	IGSF8	JARID2	JDP2	KIAA0556	KIAA1109	KLF1	KLF11	LAMB1	LAMB3	LAMB4	LIMCH1	LRRC15	MAMDC2	MARK4	MBTD1	MCM10	MCM2	MCM3AP	MEFV	MEGF6
HSPA5	IGSF10	IGSF8	JARID2	JDP2	KIAA0556	KIAA1109	KLF1	KLF11	LAMB1	LAMB3	LAMB4	LIMCH1	LRRC15	MAMDC2	MARK4	MBTD1	MCM10	MCM2	MCM3AP	MEFV
HSP90B1	HSPA12B	HSPA5	IGSF10	IGSF8	JARID2	JDP2	KIAA0556	KIAA1109	KLF1	KLF11	LAMB1	LAMB3	LAMB4	LIMCH1	LRRC15	MAMDC2	MARK4	MBTD1	MCM10	MCM2
IGSF10	IGSF8	JARID2	JDP2	KIAA0556	KIAA1109	KLF1	KLF11	LAMB1	LAMB3	LAMB4	LIMCH1	LRRC15	MARK4	MBTD1	MCM10	MCM2	MCM3AP	MEFV	MEGF6	METAP2
LRRC15	MARK4	MBTD1	MCM10	MCM3AP	MEFV	MEGF6	MFN1	MGAT5B	MLKL	MMP13	MMS22L	MST1R	MSTN	MTMR11	MYO7B	NACC2	NDUFB7	NR1D2	NR4A1	NUP205
SRPX2	SVEP1	TGFBI	THBS2	TMC2	TMEM63B	TONSL	TRIML2	TRRAP	TTC28	UNC5C	WNK1	WT1	ZAN							
MARK4	MBTD1	MCM10	MCM3AP	MEFV	MEGF6	MFN1	MGAT5B	MLKL	MMP13	MMS22L	MST1R	MSTN	MTMR11	MYO7B	NACC2	NDUFB7	NR1D2	NR4A1	NUP205	OBSL1

MGAT5B	MLKL	MMP13	MMS22L	MST1R	MSTN	MTMR11	MYO7B	NACC2	NDUFB7	NR1D2	NR4A1	NUP205	OBSL1	OCSTAMP	OLFM3	OLFML3	OTOF	P3H3	PANX3	PCNX
METAP2	MFN1	MGAT5B	MLKL	MMP13	MMS22L	MRC1	MST1R	MSTN	MTMR11	MYO7B	NACC2	NDUFB7	NR1D2	NR4A1	NUP205	OBSL1	OCSTAMP	OLFM3	OTOF	P3H3
MEGF6	METAP2	MFN1	MGAT5B	MLKL	MMP13	MMS22L	MRC1	MST1R	MSTN	MTMR11	MYO7B	NACC2	NDUFB7	NR1D2	NR4A1	NUP205	OBSL1	OCSTAMP	OLFM3	OTOF
MCM3AP	MEFV	MEGF6	METAP2	MFN1	MGAT5B	MLKL	MMP13	MMS22L	MRC1	MST1R	MSTN	MTMR11	MYO7B	NACC2	NDUFB7	NR1D2	NR4A1	NUP205	OBSL1	OCSTAMP
MFN1	MGAT5B	MLKL	MMP13	MMS22L	MST1R	MSTN	MTMR11	MYO7B	NACC2	NDUFB7	NR1D2	NR4A1	NUP205	OBSL1	OCSTAMP	OLFM3	OLFML3	OTOF	P3H3	PANX3
OBSL1	OCSTAMP	OLFML3	OTOF	P3H3	PANX3	PCNX	PER1	PER2	PER3	PIEZO2	PLCD1	PLXNB1	POSTN	PRSS35	PRSS36	PTPRB	PTX3	RAP1GAP2	RFPL1/RFF	RHBG
OCSTAMP	OLFML3	OTOF	P3H3	PANX3	PCNX	PER1	PER2	PER3	PIEZO2	PLCD1	PLXNB1	POSTN	PRSS35	PRSS36	PTPRB	PTX3	RAP1GAP2	RFPL1/RFF	RHBG	RHCG

PER1	PER2	PER3	PIEZO2	PLCD1	PLXNB1	POSTN	PRSS35	PRSS36	PTPRB	PTX3	RAP1GAP2	RFPL1/RFF	RHBG	RHCG	RORB	SALL3	SATB2	SCN4A	SEMA5A	SHTN1
PANX3	PCNX	PER1	PER2	PER3	PHOSPHO	PIEZO2	PLCD1	PLXNB1	POSTN	PRSS35	PRSS36	PTPRB	PTX3	RAP1GAP2	RFPL1/RFF	RHBG	RHCG	RORB	SALL3	SATB2
P3H3	PANX3	PCNX	PER1	PER2	PER3	PHOSPHO	PIEZO2	PLCD1	PLXNB1	POSTN	PRSS35	PRSS36	PTPRB	PTX3	RAP1GAP2	RFPL1/RFF	RHBG	RHCG	RORB	SALL3
OLFM3	OTOF	P3H3	PANX3	PCNX	PER1	PER2	PER3	PHOSPHO	PIEZO2	PLCD1	PLXNB1	POSTN	PRSS35	PRSS36	PTPRB	PTX3	RAP1GAP2	RFPL1/RFF	RFPL4A/RF	RHBG
PCNX	PER1	PER2	PER3	PIEZO2	PLCD1	PLXNB1	POSTN	PRSS35	PRSS36	PTPRB	PTX3	RAP1GAP2	RFPL1/RFF	RHBG	RHCG	RORB	SALL3	SATB2	SCN4A	SEMA5A
RHCG	RORB	SALL3	SCN4A	SEMA5A	SHTN1	SIK3	SLC14A1	SLC14A2	SLC22A7	SLC25A41	SLC4A1	SLC4A3	SLC52A3	SLITRK3	SRPX	SRPX2	SVEP1	TGFBI	THBS2	TMC2
RORB	SALL3	SCN4A	SEMA5A	SHTN1	SIK3	SLC14A1	SLC14A2	SLC22A7	SLC25A41	SLC4A1	SLC4A3	SLC52A3	SLITRK3	SRPX	SRPX2	SVEP1	TGFBI	THBS2	TMC2	TMEM63B

SIK3	SLC14A1	SLC14A2	SLC22A7	SLC25A41	SLC4A1	SLC4A3	SLC52A3	SLITRK3	SRPX	SRPX2	SUZ12	SVEP1	TGFBI	THBS2	TMC2	TMEM63B	TNC	TNMD	TONSL	TRIML2
SCN4A	SEMA5A	SHTN1	SIK3	SLC14A1	SLC14A2	SLC22A7	SLC25A41	SLC4A1	SLC4A3	SLC52A3	SLITRK3	SRPX	SRPX2	SUZ12	SVEP1	TGFBI	THBS2	TMC2	TMEM63B	TNC
SATB2	SCN4A	SEMA5A	SHTN1	SIK3	SLC14A1	SLC14A2	SLC22A7	SLC25A41	SLC4A1	SLC4A3	SLC52A3	SLITRK3	SRPX	SRPX2	SUZ12	SVEP1	TGFBI	THBS2	TMC2	TMEM63B
RHCG	RORB	SALL3	SATB2	SCN4A	SEMA5A	SHTN1	SIK3	SLC14A1	SLC14A2	SLC22A7	SLC25A41	SLC4A1	SLC4A3	SLC52A3	SLITRK3	SRPX	SRPX2	SUZ12	SVEP1	TGFBI
SHTN1	SIK3	SLC14A1	SLC14A2	SLC22A7	SLC25A41	SLC4A1	SLC4A3	SLC52A3	SLITRK3	SRPX	SRPX2	SUZ12	SVEP1	TGFBI	THBS2	TMC2	TMEM63B	TNC	TNMD	TONSL
TMEM63B	TNC	TNMD	TONSL	TRIML2	TRRAP	TSPEAR	TTC28	TTR	TUBB1	UNC5C	WDR90	WNK1	WT1	ZAN						
TNC	TNMD	TONSL	TRIML2	TRRAP	TSPEAR	TTC28	TTR	TUBB1	UNC5C	WDR90	WNK1	WT1	ZAN							

TRRAP	TSPEAR	TTC28	TTR	TUBB1	UNC5C	WDR90	WNK1	WT1	ZAN						
TNMD	TONSL	TRIML2	TRRAP	TSPEAR	TTC28	TTR	TUBB1	UNC5C	WDR90	WNK1	WT1	ZAN			
TNC	TNMD	TONSL	TRIML2	TRRAP	TSPEAR	TTC28	TTR	TUBB1	UNC5C	WDR90	WNK1	WT1	ZAN		
THBS2	TMC2	TMEM63B	TNC	TNMD	TONSL	TRIML2	TRRAP	TSPEAR	TTC28	TTR	TUBB1	UNC5C	WDR90	WNK1	WT1	ZAN
TRIML2	TRRAP	TSPEAR	TTC28	TTR	TUBB1	UNC5C	WDR90	WNK1	WT1	ZAN					
