# Supplementary material for: Waveband specific transcriptional control of select genetic pathways in vertebrate skin (Xiphophorus maculatus)
Source: BMC Genomics. 2018 May 10;19:355. doi: 10.1186/s12864-018-4735-5 (PMC5946439; doi:10.1186/s12864-018-4735-5)
Supplement: Supplementary file 2 — Table S2a–k. A list of all differentially modulated genes used by IPA enrichment software to predict the direction of change for each functional class represented in Additional file 1: Table S1. Table a is FL, tables b–e are the 50 nm wavebands and tables g–k are the 10 nm wavebands. (ZIP 701 kb) [file 12864_2018_4735_MOESM2_ESM.zip › TableS2i_520-530nm.pdf]

Functional Class	p-Value	Activation	# Genes	Genes
cell viability of cervical canc	6.09E-04	-3.747	13	ACER2,ATR,CERK,EGFR,FA2H,FANCA,HBB,MMS22L,PIK3R2,PPFIA2,PPFIA4,PRKDC,TONSL
differentiation of muscle ce	1.29E-03	-3.556	10	ARHGAP26,CTSB,DDR1,FBXO32,IRS1,JDP2,MSTN,MTOR,NMRK2,SKI
cell viability of tumor cell lin	3.52E-04	-3.458	34	ACER2,ANGPT2,AQP3,ATF3,ATR,BHLHE40,CA2,CERK,CUL9,E2F1,EGFR,EPHB4,FA2H,FANCA,FASN,FOSL1,HBB,HMGA1,HMOX1,HSP90B1,HSPA5
microtubule dynamics	6.90E-06	-3.444	53	AATK,AGRN,ANGPT2,AREG,ARHGAP32,ARHGEF9,ATF3,CACNA1A,CAV3,CELSR2,CEP192,CHRNB2,CLASP1,CNTN4,CSF1R,CTSV,CUL7,CUL9,DDF
organization of cytoskeleto	5.19E-05	-3.306	57	AATK,AGRN,ANGPT2,AREG,ARHGAP32,ARHGEF9,ATF3,CACNA1A,CAV3,CELSR2,CEP192,CHRNB2,CLASP1,CNTN4,CSF1R,CTSV,CUL7,CUL9,DDF
formation of cellular protrus	1.96E-04	-3.244	39	AGRN,AREG,ARHGAP32,ARHGEF9,ATF3,CACNA1A,CELSR2,CHRNB2,CNTN4,CUL7,DDR1,DKK3,EEF1A1,EGFR,EPHB3,FASN,HERC1,LRP4,MAGI2,
organization of cytoplasm	1.18E-04	-3.137	60	AATK,ABCA1,AGRN,ANGPT2,AREG,ARHGAP32,ARHGEF9,ATF3,CACNA1A,CAV3,CELSR2,CEP192,CHRNB2,CLASP1,CNTN4,CSF1R,CTSV,CUL7,CL
development of body axis	4.75E-05	-3.116	46	AATK,ARNTL,ATRN,CACNA1A,CHRNB2,COL11A1,CRISPLD1,CSF1R,CTSD,CYP26A1,DDR1,DPT,E2F1,ECE1,EGFR,EPHB3,F3,FAT3,GLI2,HSP90B1,I
endoplasmic reticulum stre	5.13E-04	-2.982	7	CTSB,CTSD,DNAJB9,HMOX1,HSP90B1,HSPA5,MTOR
endoplasmic reticulum stre	6.29E-05	-2.98	11	ATF3,CTSB,CTSD,DNAJB9,HMOX1,HSP90B1,HSPA1A/HSPA1B,HSPA5,HSPA6,MTOR,WFS1
cell survival	4.97E-05	-2.919	57	ACER2,AGRN,ANGPT2,ANXA5,AQP3,AREG,ATF3,ATR,BHLHE40,CA2,CERK,CISH,CLOCK,COL17A1,CSF1R,CTSB,CUL9,E2F1,EGFR,EPHB3,EPHB4,
development of head	1.67E-05	-2.869	45	AATK,ARNTL,ATRN,CACNA1A,CHRNB2,COL11A1,CRISPLD1,CSF1R,CTSD,CYP26A1,DDR1,DPT,E2F1,ECE1,EGFR,EPHB3,F3,FAT3,GLI2,IL6ST,INSI
neuritogenesis	4.11E-04	-2.805	27	AGRN,AREG,ARHGAP32,ATF3,CACNA1A,CELSR2,CHRNB2,CNTN4,CUL7,DDR1,EGFR,EPHB3,HERC1,LRP4,MAGI2,METRN,MNX1,MTOR,PAK6,PLX
interphase	1.33E-03	-2.766	28	ATF3,ATR,BHLHE40,CREG1,CSF1R,DOT1L,E2F1,E2F2,EGFR,FASN,GLI2,HMGA1,HMOX1,ILKAP,IRS1,MCM10,MMS22L,MTOR,POLE,PRKDC,PTCH1
behavior	1.82E-04	-2.741	42	APBA1,ARHGEF9,ARNTL,CACNA1A,CAPN1,CHRNB2,CLOCK,CRTC1,CRY1,CTSB,CTSV,E2F1,EPHB3,FA2H,HBB,HMGCR,HMOX1,HSD11B2,HSPA1/
stress response of cells	1.30E-03	-2.709	9	ANGPT2,CTSB,CTSD,DNAJB9,HMOX1,HSP90B1,HSPA5,MTOR,NR3C2
mitosis	8.67E-04	-2.704	23	ADGRL2,AREG,ATR,CEP192,CLASP1,CSF1R,CUL7,CUL9,DOT1L,E2F1,E2F2,EGFR,IRS1,JARID2,MTOR,PBRM1,PRKCB,PTCH1,PTX3,THBS1,TIMP2,
formation of filopodia	1.04E-04	-2.64	12	AGRN,ARHGEF9,EEF1A1,MKL1,MTSS1,NEO1,PAK6,RELN,RHOU,SRGAP2,TNC,TNFRSF1A
morphogenesis of neurites	4.17E-04	-2.618	21	AGRN,AREG,ARHGAP32,ATF3,CACNA1A,CELSR2,CHRNB2,CNTN4,CUL7,EGFR,EPHB3,HERC1,LRP4,MAGI2,MNX1,MTOR,PLXNB1,RELN,SRGAP2,T
quantity of endocrine cells	5.78E-04	-2.584	9	ANGPT2,ARNTL,COL5A3,CSF1R,E2F1,E2F2,FSTL3,MNX1,WFS1
differentiation of adipocytes	2.72E-04	-2.58	15	ALOXE3,ARNTL,ATF3,CREB5,E2F1,HMOX1,INSIG1,IRS1,JDP2,LRP6,MKL1,MSTN,NR3C2,SIK3,ZNF385A
formation of brain	7.59E-05	-2.474	26	AATK,ATRN,CACNA1A,CHRNB2,CSF1R,CYP26A1,E2F1,EGFR,EPHB3,F3,GLI2,IL6ST,LRP6,MTOR,PRKDC,PTCH1,PTPRS,RELN,RORB,SEMA5A,SEF
obesity	1.47E-05	-2.458	26	ANGPT2,ARNTL,ATF3,ATRN,CA1,CA13,CA2,CA3,CLOCK,COL5A3,FASN,HMGA1,HMGCR,HSD11B2,HSPA1A/HSPA1B,IL6ST,IRS1,LRP6,MMP19,MST
differentiation of cells	7.69E-10	-2.401	100	ADAM8,ADAMTS20,AGRN,ALOX15B,ALOXE3,ANGPT2,AQP3,AREG,ARHGAP26,ARHGAP32,ARNTL,ATF3,BHLHE40,CA2,CACNA1A,CAV3,CD109,CH
senescence of cells	5.89E-04	-2.381	14	ATR,BHLHE40,CBX7,CUL7,DNMT3B,DOT1L,E2F1,EGFR,FANCA,FASN,HSPA1A/HSPA1B,MTOR,PBRM1,WT1
proliferation of cells	7.12E-08	-2.365	137	AATK,ABCC5,ACER2,AGRN,ALOX15B,ANGPT2,AREG,ARHGAP32,ARNTL,ARNTL2,ATAD2,ATF3,ATR,BHLHE40,CA3,CACNA1A,CAPN1,CAV3,CBX7,(
size of body	2.38E-05	-2.305	40	ABCA1,APBA1,AREG,ARNTL,ATRN,CACNA1A,CBX7,CERK,CHTF18,COL5A3,CSF1R,CTSD,CUL9,E2F1,E2F2,EGFR,F3,GLI2,GPX2,HMGA1,HMOX1,IL
quantity of connective tissu	1.28E-06	-2.285	34	ABCA1,ANGPT2,ARNTL,CAPN1,COL5A3,CREB5,CTSV,DNMT3B,DPT,E2F1,EGFR,FASN,FSTL3,GLI2,HBB,HMOX1,HSD11B2,HSPA5,IL6ST,INSIG1,IRS
quantity of cells	1.76E-06	-2.274	74	ABCA1,ADAM8,AGRN,ALOX15B,ANGPT2,ARNTL,ARNTL2,ATF3,BHLHE40,C4A/C4B,C6,CACNA1A,CAPN1,CERK,CHRNB2,CISH,COL5A3,CSF1R,CTS
quantity of blood cells	7.85E-04	-2.255	41	ABCA1,ADAM8,ARNTL,ATF3,BHLHE40,C4A/C4B,C6,CACNA1A,CERK,CISH,CSF1R,CTSD,CTSV,CYP27A1,DDR1,DKK3,E2F1,E2F2,F3,FANCA,HBB,HI
cell proliferation	1.98E-05	-2.24	47	ADGRL2,ALOX15B,AREG,ARHGAP32,ARNTL,ARNTL2,ATF3,ATR,CBX7,CEP192,CLASP1,CSF1R,CUL7,CUL9,DNMT3B,DOT1L,E2F1,E2F2,EGFR,FAN
branching of epithelial tissu	5.47E-04	-2.217	9	ANGPT2,EPHB3,EPHB4,GLI2,PTPRJ,SEMA5A,TIMP2,TNC,WNT9B
development of central ner	7.52E-05	-2.192	31	AATK,ATRN,CACNA1A,CHRNB2,CSF1R,CYP26A1,E2F1,EGFR,EPHB3,F3,GLI2,IL6ST,JARID2,LRP6,MNX1,MTOR,NPTX1,PRKDC,PTCH1,PTPRJ,PTPI
development of epithelial ti	1.56E-03	-2.19	23	ANGPT2,CA2,CSF1R,CTSB,ECE1,EGFR,EPHB4,F3,GLI2,HMOX1,JAG2,MTOR,ODC1,PRKDC,PTPRJ,SEMA5A,SLC14A1,SMOC2,THBS1,TIMP2,WNK1
volume of tumor	8.09E-04	-2.172	5	E2F1,EGFR,F3,PRKCB,TIMP2
morphogenesis of cardiova	6.31E-04	-2.172	14	ANGPT2,COL11A1,CYP26A1,DSP,EPHB4,LRP6,MTOR,ODC1,PBRM1,PTCH1,PTPRJ,THBS1,TIMP2,ZMIZ1
entry into S phase	1.56E-03	-2.171	9	BHLHE40,E2F1,E2F2,HMGA1,HMOX1,ILKAP,IRS1,MCM10,MTOR
benign neoplasia	1.91E-09	-2.144	53	AGRN,ANGPT2,ANXA5,ATR,ATRN,C4A/C4B,CA1,CA2,CACNA1A,CBX7,COL11A1,COL16A1,COL17A1,COL19A1,COL21A1,COL24A1,COL27A1,COL4/
quantity of subcutaneous fa	7.55E-05	-2.134	7	ARNTL,COL5A3,CREB5,DPT,INSIG1,SIK3,STEAP4
weight loss	4.61E-04	-2.112	14	ALOXE3,AREG,ARNTL,ATF3,CLOCK,COL19A1,COL5A3,CRY1,CSF1R,EGFR,HMOX1,HSP90B1,HSPA1A/HSPA1B,ITPR3
neuroepithelial tumor	8.59E-07	-2.109	42	ADAM8,ANGPT2,ATR,C6,CD109,COL11A1,COL5A3,COL6A5,CSF1R,CYP51A1,DENND4B,DKK3,DQX1,E2F1,EGFR,FA2H,HERC1,HKDC1,HMCN1,HSF
expression of protein	1.11E-03	-2.106	17	ANGPT2,CAV3,EEF1A1,EGFR,FOSL1,GRB7,HSPA1A/HSPA1B,HSPA5,IRS1,MSTN,MTOR,RPL24,SKI,THBS1,TP53BP1,WT1,YBX2
quantity of islet cells	3.18E-04	-2.091	7	ARNTL,COL5A3,E2F1,E2F2,FSTL3,MNX1,WFS1
growth of connective tissue	1.57E-05	-2.082	33	AREG,ATF3,ATR,CBX7,CD109,CSF1R,CTSB,CTSD,CTSV,DKK3,E2F1,EGFR,FBRS,FOSL1,GLI2,HMGA1,HMOX1,HSPA1A/HSPA1B,IL6ST,IRS1,KMT2(
proliferation of connective t	4.22E-05	-2.076	30	AREG,ATF3,ATR,CBX7,CD109,CSF1R,CTSB,CTSD,CTSV,DKK3,E2F1,EGFR,FBRS,FOSL1,GLI2,HMOX1,HSPA1A/HSPA1B,IL6ST,IRS1,KMT2C,MLLT6
synthesis of DNA	1.93E-05	-2.075	24	ALOX15B,ANGPT2,AREG,ATF3,ATR,CSF1R,E2F1,EGFR,GLI2,HSPA1A/HSPA1B,IL6ST,IRS1,MCM3AP,MMS22L,MTOR,NCOA1,ODC1,POLE,PRKCB,S
proliferation of fibroblasts	6.98E-04	-2.074	18	ATF3,CBX7,CSF1R,CTSB,CTSD,DKK3,E2F1,EGFR,FBRS,FOSL1,HMOX1,IL6ST,KMT2C,MSTN,PRKDC,SKI,TNFRSF1A,ZMIZ1
quantity of secretory structu	2.77E-04	-2.063	11	ALOX15B,ANGPT2,ARNTL,COL5A3,CSF1R,E2F1,E2F2,EGFR,FSTL3,MNX1,WFS1
quantity of connective tissu	1.18E-04	-2.056	15	ANGPT2,ARNTL,CAPN1,CTSV,DNMT3B,EGFR,FSTL3,GLI2,HSD11B2,IL6ST,IRS1,JAG2,PER1,SIK3,WT1
cell viability	9.75E-05	-2.018	53	ACER2,AGRN,ANGPT2,ANXA5,AQP3,AREG,ATF3,ATR,BHLHE40,CA2,CERK,CISH,CLOCK,COL17A1,CSF1R,CTSB,CUL9,E2F1,EGFR,EPHB3,EPHB4,
vasculogenesis	2.24E-05	-2.012	36	ANGPT2,ARNTL,ATF3,C6,CSF1R,CTSB,CUL7,CYP51A1,DDR1,E2F1,ECE1,EGFR,EPHB3,EPHB4,F3,HMOX1,IL6ST,IRS1,LUZP1,MTOR,NCOA1,ODC1,
viability	1.43E-03	-2	7	CTSD,CYP26A1,E2F1,EGFR,HBE1,PRKDC,TP53BP1
failure of kidney	3.04E-04	-2	13	ATF3,C4A/C4B,HMGCR,HMOX1,LGMN,MAGI2,MTOR,NR3C2,PRKCB,THBS1,TNFRSF1A,WNT9B,WT1
apoptosis	6.93E-08	2	106	AATK,ABCA1,ADAM8,ADAMTS20,ADAMTSL4,ADGRL2,AGRN,ALOX15B,ANGPT2,ANXA5,AQP3,AREG,ATAD2,ATF3,ATR,BHLHE40,C6,CA3,CACNA1/
cell death	2.00E-07	2.004	125	AATK,ABCA1,ABCC5,ACER2,ADAM8,ADAMTS20,ADAMTSL4,ADGRL2,AGRN,ALOX15B,ANGPT2,ANXA5,AQP3,AREG,ARNTL,ATAD2,ATF3,ATR,BHLI
islet cell tumor	7.52E-05	2.021	6	CSF1R,CTSB,CTSV,CUL9,MTOR,THBS1
differentiation of bone cells	7.09E-04	2.034	20	ADAM8,AREG,CA2,CSF1R,EGFR,EPHB4,FASN,FOSL1,FSTL3,GLI2,IL6ST,IRS1,JDP2,LRP4,MTOR,OCSTAMP,PTCH1,RORB,SKI,TNC
hypertrophy of heart cells	2.39E-04	2.038	11	ANGPT2,ATF3,CA2,CAV3,CSRP3,CTSB,CTSD,FBXO32,FSTL3,MTOR,PRKCB
proliferation of fibroblast ce	2.81E-06	2.044	26	ANGPT2,AREG,ARHGAP32,CNOT6L,CSF1R,CTSD,CUL7,E2F1,E2F2,EGFR,GRB7,HMGA1,HMOX1,HSPA1A/HSPA1B,IRS1,MLLT6,MSTN,ODC1,RHOU
thoracic neoplasm	1.87E-03	2.047	43	ABCC5,ADAM8,AGRN,AREG,ATF3,ATR,CBX7,CERS5,COL11A1,CSF1R,CTSD,CUL9,DDR1,DLEC1,E2F1,EEF1A1,EGFR,EPHB4,F3,FASN,FAT1,HMOX
invasion of malignant tumo	8.33E-04	2.066	10	CTSB,CTSL,EGFR,F3,FASN,HMOX1,HSPA5,PAK6,TIMP2,WT1
polyp	9.70E-04	2.067	6	EGFR,MSTN,PIK3R2,PRKDC,PTCH1,THBS1
abnormal ratio tissue	2.68E-04	2.067	7	EGFR,GLI2,MSTN,PIK3R2,PRKDC,PTCH1,THBS1
head and neck cancer	4.69E-04	2.085	44	ADAM8,ADAMTS10,ALOX15B,ANGPT2,ATR,C6,CBX7,CD109,COL11A1,COL5A3,COL6A5,CSF1R,CYP51A1,DENND4B,DKK3,DPT,DQX1,E2F1,EGFR,E
lymphohematopoietic canc	5.61E-09	2.093	86	ABCA1,ADGRL2,ALOX12B,ANXA5,APBA1,ARHGAP26,ATR,ATRN,BAG2,C4A/C4B,CACNA1A,CARMIL3,CBX7,CCDC173,CD109,CELSR2,CEP192,CNT
left ventricular dysfunction	4.66E-04	2.118	8	ABCA1,ANGPT2,CAV3,DSP,EGFR,FSTL3,HMGA1,MTOR
myocardial dysfunction	4.90E-04	2.118	9	ABCA1,ANGPT2,ANXA5,CAV3,DSP,EGFR,FSTL3,HMGA1,MTOR
necrosis	2.73E-05	2.12	94	AATK,ACER2,ADAM8,ADGRL2,AGRN,ANGPT2,AREG,ATAD2,ATF3,ATR,BHLHE40,CA3,CACNA1A,CAPN1,CAV3,CDC45,CERK,CERS5,CISH,CLASP1
muscular hypertrophy	8.95E-04	2.121	12	ANGPT2,ATF3,CA2,CAV3,CSRP3,ECE1,FBXO32,FSTL3,IL6ST,MSTN,MTOR,PRKCB
midline defect	4.72E-04	2.133	15	CYP26A1,CYP51A1,DNMT3B,ECE1,EGFR,EPHB3,INSIG1,JAG2,LRP6,MSTN,PTCH1,PTPRS,SKI,WFIKKN2,WNT9B
lymphoid cancer	1.29E-07	2.195	76	ABCA1,ADGRL2,ARHGAP26,ATR,BAG2,C4A/C4B,CACNA1A,CARMIL3,CCDC173,CD109,CELSR2,CEP192,CNTN4,COL16A1,COL17A1,COL24A1,COL
Lymphoid Cancer and Tum	2.93E-07	2.195	77	ABCA1,ADGRL2,ARHGAP26,ATR,BAG2,C4A/C4B,CACNA1A,CARMIL3,CCDC173,CD109,CELSR2,CEP192,CNTN4,COL16A1,COL17A1,COL24A1,COL
endocrine gland tumor	4.23E-08	2.217	73	ADAM8,ADAMTS10,ADAMTSL4,AGRN,ALOX15B,ANGPT2,ANO9,ANXA5,AQP12A/AQP12B,C4A/C4B,CA13,CACNA1A,CBX7,CELSR2,CHRNB2,CHTF1
accumulation of cells	1.75E-03	2.287	17	ANGPT2,C4A/C4B,C6,DDR1,DOT1L,E2F1,F3,FASN,FOSL1,HMOX1,IL6ST,LRP6,MCM10,PTX3,THBS1,TIMP2,TNFRSF1A
lymphohematopoietic neop	1.27E-08	2.29	87	ABCA1,ADGRL2,ALOX12B,ANXA5,APBA1,ARHGAP26,ATR,ATRN,BAG2,C4A/C4B,CACNA1A,CARMIL3,CBX7,CCDC173,CD109,CELSR2,CEP192,CNT
apoptosis of tumor cell line	3.80E-04	2.291	48	AATK,ADGRL2,ANGPT2,AREG,ATAD2,ATF3,ATR,BHLHE40,CAPN1,CLASP1,CSF1R,CTSB,CTSD,CUL9,CYFIP2,DHCR24,DKK3,E2F1,E2F2,EGFR,EPI
hypertrophy of cells	4.41E-04	2.32	16	ANGPT2,ATF3,CA2,CAV3,CSRP3,CTSB,CTSD,ECE1,FBXO32,FSTL3,IL6ST,MAGI2,MMP19,MSTN,MTOR,PRKCB
hypertrophy of tissue	9.66E-04	2.355	14	ANGPT2,ATF3,CA2,CAV3,CSRP3,ECE1,FBXO32,FSTL3,HSD11B2,IL6ST,MMP19,MSTN,MTOR,PRKCB
hematologic cancer	6.68E-09	2.36	85	ABCA1,ADGRL2,ALOX12B,ANXA5,APBA1,ARHGAP26,ATR,ATRN,BAG2,C4A/C4B,CACNA1A,CARMIL3,CBX7,CCDC173,CD109,CELSR2,CEP192,CNT
malignant solid tumor	8.02E-13	2.36	329	AACS,AATK,ABCA1,ABCC5,ABHD2,ABI3BP,ACER2,ACSBG1,ADAM8,ADAMTS10,ADAMTS20,ADAMTSL1,ADAMTSL4,ADGRG2,ADGRL2,AEBP2,AGR
hypertrophy of cardiac mus	2.51E-04	2.401	11	ANGPT2,ATF3,CA2,CAV3,CSRP3,ECE1,FBXO32,FSTL3,IL6ST,MTOR,PRKCB
proliferation of tumor cells	5.84E-05	2.417	25	AREG,ATF3,CSF1R,CTSB,CTSV,DOCK4,EEF1A1,EGFR,F3,FASN,FOSL1,HMGCR,HMOX1,HSPA1A/HSPA1B,HSPA5,IRS1,MTOR,NCOA1,ODC1,PTCH
craniofacial abnormality	1.01E-04	2.452	20	ATR,CYP26A1,CYP51A1,DNMT3B,ECE1,EGFR,EPHB3,GLI2,INSIG1,JAG2,LRP6,MSTN,NALCN,POLE,PTCH1,PTPRJ,PTPRS,SKI,WFIKKN2,WNT9B
congenital malformation of	1.09E-03	2.455	17	ANGPT2,C4A/C4B,DBP,E2F1,EGFR,EPHB3,GLI2,GPX2,HMGCR,HMOX1,LTBP4,MAGI2,MTOR,NCOA1,TIMP2,WNT9B,WT1
neuroendocrine tumor	1.40E-04	2.461	17	COL11A1,CSF1R,CTSB,CTSV,CUL9,E2F1,EGFR,FKBP10,HMGA1,HMOX1,HSD11B2,HSP90B1,MTOR,ODC1,POLE,RELN,THBS1
catabolism of protein	7.14E-04	2.481	27	ADAM8,ADAMTS20,ARNTL,CAPN1,CTSB,CTSD,CTSL,CTSV,CYFIP2,ECE1,EGFR,FBXO32,HSP90B1,HSPA1A/HSPA1B,HSPA5,LGMN,LTBP4,MMP19,
neonatal death	8.32E-04	2.489	21	ALOX12B,ALOXE3,APBA1,CREB5,CUL7,ECE1,F3,GLI2,IL6ST,JAG2,LRP4,LRP6,MAGI2,MNX1,NALCN,PHF21A,PTPRS,SALL3,SIK3,WNT9B,WT1
hematological neoplasia	1.62E-08	2.554	86	ABCA1,ADGRL2,ALOX12B,ANXA5,APBA1,ARHGAP26,ATR,ATRN,BAG2,C4A/C4B,CACNA1A,CARMIL3,CBX7,CCDC173,CD109,CELSR2,CEP192,CNT
head and neck neoplasia	6.70E-09	2.716	97	ADAM8,ADAMTS10,ADAMTSL4,AGRN,ALOX15B,ANGPT2,ANO9,ANXA5,AQP12A/AQP12B,ATR,C4A/C4B,C6,CA13,CACNA1A,CBX7,CD109,CELSR2,(
cell death of tumor cell line	1.05E-04	2.731	60	AATK,ACER2,ADGRL2,ANGPT2,AREG,ATAD2,ATF3,ATR,BHLHE40,CACNA1A,CAPN1,CERK,CERS5,CLASP1,CSF1R,CTSB,CTSD,CUL7,CUL9,CYFIP
invasion of tumor	4.61E-05	2.753	16	AREG,COL7A1,CTSB,CTSL,CTSV,DSP,EGFR,F3,FASN,HMOX1,HSPA5,LRRC15,PAK6,THBS1,TIMP2,WT1
cancer	1.70E-12	2.767	331	AACS,AATK,ABCA1,ABCC5,ABHD2,ABI3BP,ACER2,ACSBG1,ADAM8,ADAMTS10,ADAMTS20,ADAMTSL1,ADAMTSL4,ADGRG2,ADGRL2,AEBP2,AGR
synthesis of ceramide	1.43E-03	2.964	6	ALOX12B,ALOXE3,CERK,CERS5,FASN,PRKDC
tumorigenesis of malignant	1.67E-03	2.978	14	CTSL,CUL9,DNMT3B,E2F1,E2F2,EGFR,GLI2,GPX2,JARID2,ODC1,PRKDC,PTCH1,TAOK1,THBS1
organismal death	2.22E-08	3.018	100	ABCA1,AGRN,ALOX12B,ALOXE3,ANGPT2,APBA1,AREG,ARNTL,ATF3,ATR,C4A/C4B,CACNA1A,CAPN1,CDC45,CERK,CHRNB2,CHTF18,COL11A1,C(
metabolism of membrane li	1.52E-06	3.185	25	ABCA1,AKR1D1,ALOX12B,ALOXE3,APOF,CERK,CERS5,CSF1R,CYP27A1,CYP51A1,DHCR24,EBP,EEF1A1,FASN,FOSL1,HMGCR,HMOX1,INSIG1,LS
fibrosis	1.04E-03	3.208	25	ANGPT2 ATF3 CAV3 CISH CSF1R CSRP3 CTSB DSP EGFR F3 FSTL3 HBB HMOX1
perinatal death	1.76E-05	3.418	31	ABCA1,AGRN,ALOX12B,ALOXE3,APBA1,CREB5,CUL7,CYP26A1,E2F1,E2F2,ECE1,EGFR,F3,GLI2,HMOX1,IL6ST,JAG2,LRP4,LRP6,MAGI2,MKL1,MN>
hypertrophy	2.47E-05	3.5	20	ABCA1 ANGPT2 ATF3 CA2 CAV3 CSRP3 CTSB CTSD ECE1 EGFR FBXO32 FSTL3 HMGA1

OX1,HSP90B1,HSPA5,INSIG1,JAG2,MMS22L,MTOR,PIK3R2,PPFIA2,PPFIA4,PRKDC,SVIL,TIMP2,TNFRSF1A,TONSL,TP53BP1
CTSV,CUL7,CUL9,DDR1,DKK3,DOCK4,DSP,EEF1A1,EGFR,EPHB3,FASN,HERC1,LRP4,MAGI2,METRN,MKL1,MNX1,MTOR,MTSS1,NEO1,PAK6,PLXNB1,POC1B,RELN,RHOU,SALL3,SEMA5A,SEPT4,SGK2,SRGAP2,STXBP5,TAOK1,THBS1,TNC,TNFRSF1A,T
CTSV,CUL7,CUL9,DDR1,DKK3,DOCK4,DSP,EEF1A1,EGFR,EPHB3,F3,FASN,FAT1,HERC1,LRP4,MAGI2,METRN,MKL1,MNX1,MTOR,MTSS1,NEO1,PAK6,PLXNB1,POC1B,PRKDC,RELN,RHOU,SALL3,SEMA5A,SEPT4,SGK2,SLC3A2,SRGAP2,STXBP5,TAOK1,
HERC1,LRP4,MAGI2,METRN,MKL1,MNX1,MTOR,MTSS1,NEO1,PAK6,PLXNB1,POC1B,RELN,RHOU,SEPT4,SRGAP2,STXBP5,THBS1,TNC,TNFRSF1A,TNIK,UNC119B,WISP2
CSF1R,CTSV,CUL7,CUL9,DDR1,DKK3,DOCK4,DSP,EEF1A1,EGFR,EPHB3,F3,FASN,FAT1,HERC1,LRP4,MAGI2,METRN,MKL1,MNX1,MTOR,MTSS1,MUL1,NEO1,PAK6,PLXNB1,POC1B,PRKDC,PTX3,RELN,RHOU,SALL3,SEMA5A,SEPT4,SGK2,SLC3A2,SRGA
FAT3,GLI2,HSP90B1,IL6ST,INSIG1,JAG2,LMO7,LRP4,LRP6,MTOR,PRKCB,PRKDC,PTCH1,PTPRS,RELN,RORB,SAG,SEMA5A,SEPT4,SKI,SLC4A10,THBS1,TIMP2,TNC,TNFRSF1A,TNIK,WFIKKN2,WNT9B,WT1

EGFR,EPHB3,EPHB4,FA2H,FANCA,FASN,FAT1,FBXO32,FOSL1,HBB,HMGA1,HMOX1,HSP90B1,HSPA1A/HSPA1B,HSPA5,IL6ST,INSIG1,IRS1,JAG2,JDP2,MEFV,MMS22L,MTOR,ODC1,PIK3R2,PLXNB1,PPFIA2,PPFIA4,PRKCB,PRKDC,PTCH1,SLX4,SVIL,THE
FAT3,GLI2,IL6ST,INSIG1,JAG2,LMO7,LRP4,LRP6,MTOR,PRKCB,PRKDC,PTCH1,PTPRS,RELN,RORB,SAG,SEMA5A,SEPT4,SKI,SLC4A10,THBS1,TIMP2,TNC,TNFRSF1A,TNIK,WFIKKN2,WNT9B,WT1
NX1,MTOR,PAK6,PLXNB1,RELN,SEPT4,SRGAP2,STXBP5,TNC,TNIK,WISP2
POLE,PRKDC,PTCH1,RHOU,SLX4,TAOK1,TIMP2,TONSL,TP53BP1,WT1
X1,HSD11B2,HSPA1A/HSPA1B,ITPR3,KCNT1,MKL1,MSTN,MTOR,NCOA1,NPTX1,NR3C2,PAK6,PER1,PER3,PHF21A,PRKCB,RELN,SALL3,SCN8A,SIK3,SLC17A8,TNC,TNFRSF1A,TNIK,WFS1,ZDHHC8

,PTX3,THBS1,TIMP2,TNC,TRRAP

XNB1,RELN,SRGAP2,TNC,TNIK

N,RORB,SEMA5A,SEPT4,SKI,SLC4A10,THBS1,TNC,TNIK
S1,LRP6,MMP19,MSTN,NCOA1,PRKCB,SIK3,TIMP2,TNFRSF1A,WT1
A1A,CAV3,CD109,CHRNB2,CISH,CNTN4,COL24A1,CREB5,CREG1,CSF1R,CSRP3,CTSB,CTSV,CYB5D2,CYP26A1,CYP27A1,DDR1,DNMT3B,DOCK4,DOT1L,DSP,E2F1,E2F2,EBP,EGFR,EPHB3,EPHB4,FASN,FBXO32,FLCN,FOSL1,FSTL3,GLI2,HMGA1,HMO

,CAPN1,CAV3,CBX7,CD109,CDC45,CERK,CHRNB2,CISH,CLOCK,CNOT6L,CREG1,CRTC1,CRY1,CSF1R,CTSB,CTSD,CTSL,CTSV,CUL7,DDR1,DHCR24,DKK3,DLEC1,DNMT3B,DOCK4,DOT1L,DPT,DSP,E2F1,E2F2,EEF1A1,EGFR,EPHB3,EPHB4,F3,FA2H,FA
X2,HMGA1,HMOX1,IL6ST,INSIG1,IRS1,ITPR3,LGMN,LMO7,MKL1,MSTN,PER1,PTCH1,PTPRJ,PTPRS,RHCG,SIK3,SLC14A1,SLC14A2,TNFRSF1A,TP53BP1,ZNF385A
PA5,IL6ST,INSIG1,IRS1,JAG2,JARID2,LGMN,MSTN,MTOR,PER1,PRKCB,PTPRS,SIK3,SLC14A1,STEAP4,WT1,ZNF385A
,COL5A3,CSF1R,CTSB,CTSD,CTSV,CUL7,CYP27A1,DDR1,DKK3,DNMT3B,DOT1L,DSP,E2F1,E2F2,EGFR,F3,FANCA,FSTL3,GLI2,HBB,HMGA1,HMOX1,HSD11B2,HSP90B1,IL6ST,IRS1,JAG2,JARID2,LGMN,LRP4,LRP6,MKL1,MMP19,MNX1,MSTN,MTOR,NEC
F2,F3,FANCA,HBB,HMOX1,HSP90B1,IL6ST,JAG2,JARID2,LGMN,LRP6,MKL1,MMP19,NR3C2,PRKCB,PRKDC,PTPRJ,SLC14A1,STEAP4,THBS1,TIMP2,TNFRSF1A,TP53BP1,ZNF385A
E2F1,E2F2,EGFR,FANCA,FASN,FOSL1,HMOX1,HSPA1A/HSPA1B,IRS1,JARID2,JDP2,LRP6,MCM2,MLLT6,MMS22L,MTOR,PBRM1,PER1,PER3,PIK3R2,PRKCB,PTCH1,PTPRB,PTX3,RHOU,SKI,THBS1,TIMP2,TNC,TRRAP,WT1

C,PTCH1,PTPRJ,PTPRS,RELN,RORB,RPL24,SEMA5A,SEPT4,SKI,SLC4A10,THBS1,TNC,TNIK
,THBS1,TIMP2,WNK1,WNT9B,WT1

4A1,COL27A1,COL4A6,COL5A3,COL7A1,CSF1R,CTSB,CUL9,DNMT3B,DPT,E2F1,E2F2,EGFR,EPHB3,FKBP10,GLI2,HMGA1,HMGCR,HMOX1,HSD11B2,IL6ST,JAG2,KMT2C,MTOR,NR3C2,ODC1,OLFM1,PBRM1,PRKCB,PTCH1,RELN,RORB,SLC5A5,THBS1,T

1,HKDC1,HMCN1,HSPA5,KIAA1109,KMT2C,MTOR,NCOA1,NPTX1,NR3C2,OTUD4,PBRM1,PRKCB,PTX3,RELN,SEMA5A,SEPT4,SHC4,SKI,TAOK1,THBS1,TNC,TNFRSF1A,TRRAP,VPS13B,WT1

C,MLLT6,MSTN,PIK3R2,PLXNB1,PRKDC,PTCH1,SKI,SLC3A2,TIMP2,TNFRSF1A,WT1,ZMIZ1
T,IRS1,KMT2C,MLLT6,MSTN,PLXNB1,PRKDC,PTCH1,SKI,SLC3A2,TIMP2,TNFRSF1A,ZMIZ1
ODC1,POLE,PRKCB,SMOC2,TIMP2,TNC,TNFRSF1A,WT1

EGFR,EPHB3,EPHB4,FA2H,FANCA,FASN,FAT1,FBXO32,FOSL1,HBB,HMGA1,HMOX1,HSP90B1,HSPA1A/HSPA1B,HSPA5,IL6ST,INSIG1,JAG2,MMS22L,MTOR,PIK3R2,PLXNB1,PPFIA2,PPFIA4,PRKCB,PRKDC,PTCH1,SLX4,SVIL,THBS1,TIMP2,TNFRSF1A,TO
MTOR,NCOA1,ODC1,PLXNB1,PRKDC,PTPRB,PTPRJ,PTX3,ROBO4,SEMA5A,SMOC2,THBS1,TIMP2,TNC,WNK1,WT1,ZMIZ1

E40,C6,CA3,CACNA1A,CAPN1,CAV3,CDC45,CHTF18,CLASP1,COL5A3,CSF1R,CTSB,CTSD,CTSL,CTSV,CUL7,CUL9,CYFIP2,DDAH2,DDR1,DHCR24,DKK3,DNMT3B,DOT1L,DSP,E2F1,E2F2,ECE1,EEF1A1,EGFR,EPHB4,F3,FANCA,FASN,FBXO32,FLCN,FOSL
TAD2,ATF3,ATR,BHLHE40,C4A/C4B,C6,CA3,CACNA1A,CAPN1,CAV3,CBX7,CDC45,CERK,CERS5,CHTF18,CISH,CLASP1,CLOCK,COL5A3,CSF1R,CTSB,CTSD,CTSL,CTSV,CUL7,CUL9,CYFIP2,DDAH2,DDR1,DHCR24,DKK3,DNMT3B,DOT1L,DSP,E2F1,E2F2

J,SKI,SLC3A2,SUZ12,THBS1,TNC,WNK1,WT1
F3,FASN,FAT1,HMOX1,HSP90B1,ITPR3,KMT2C,LMO7,MCM2,MMP19,MTOR,NCOA1,ODC1,PBRM1,PLXNB1,POLE,PRKCB,PRKDC,SCUBE2,TAOK1,THBS1,TNC,TP53BP1,WNK1,WT1

T,DQX1,E2F1,EGFR,EPHB4,FASN,GLI2,HERC1,HKDC1,HMCN1,HSP90B1,KIAA1109,KMT2C,MTOR,MTSS1,NCOA1,NPTX1,NR3C2,OTUD4,PBRM1,POLE,PRKDC,PTCH1,PTPRS,THBS1,TNC,TP53BP1,TRRAP,VPS13B
CELSR2,CEP192,CNTN4,COL16A1,COL17A1,COL24A1,COL27A1,COL6A6,COL7A1,CRTC1,CSF1R,CTSL,CUL7,CUL9,CYP51A1,DDR1,DNMT3B,DSP,E2F1,E2F2,EEF1A1,EGFR,EPHB3,EPHB4,F3,FASN,FAT1,HERC1,HMGA1,HMGCR,HMOX1,HSP90B1,HSPA

CERS5,CISH,CLASP1,CLOCK,COL5A3,CSF1R,CTSB,CTSD,CTSV,CUL7,CUL9,CYFIP2,DHCR24,DKK3,DNMT3B,DSP,E2F1,E2F2,EEF1A1,EGFR,EPHB4,EYS,F3,FANCA,FASN,FBXO32,FOSL1,FSTL3,GLI2,GPX2,HMGA1,HMOX1,HSD11B2,HSP90B1,HSPA1A/I

L17A1,COL24A1,COL27A1,COL6A6,COL7A1,CRTC1,CSF1R,CUL7,CUL9,CYP51A1,DDR1,DNMT3B,DSP,E2F1,E2F2,EEF1A1,EGFR,EPHB3,EPHB4,F3,FAT1,HERC1,HMGA1,HMGCR,HSP90B1,HSPA5,ILKAP,JARID2,KMT2C,LRBA,LRP4,LRP6,MAGI2,MKL1,MS
L17A1,COL24A1,COL27A1,COL6A6,COL7A1,CRTC1,CSF1R,CUL7,CUL9,CYP51A1,DDR1,DNMT3B,DSP,E2F1,E2F2,EEF1A1,EGFR,EPHB3,EPHB4,F3,FAT1,HERC1,HMGA1,HMGCR,HSP90B1,HSPA1A/HSPA1B,HSPA5,ILKAP,JARID2,KMT2C,LRBA,LRP4,LRF
SR2,CHRNB2,CHTF18,CISH,CNTN4,COL11A1,COL4A6,CPN2,CSF1R,CTSB,CTSV,CUL9,DNMT3B,DPP6,E2F1,EGFR,EPHB3,EPHB4,FA2H,FBRSL1,FKBP10,FOSL1,HMCN1,HMGA1,HMOX1,HSD11B2,HSP90B1,HSPA1A/HSPA1B,JAG2,KIF26A,KMT2C,LRBA,

CELSR2,CEP192,CNTN4,COL16A1,COL17A1,COL24A1,COL27A1,COL6A6,COL7A1,CRTC1,CSF1R,CTSL,CUL7,CUL9,CYP51A1,DDR1,DNMT3B,DSP,E2F1,E2F2,EEF1A1,EGFR,EPHB3,EPHB4,F3,FASN,FAT1,HERC1,HMGA1,HMGCR,HMOX1,HSP90B1,HSPA
E2F1,E2F2,EGFR,EPHB4,FANCA,FASN,FBXO32,GPX2,HMGA1,HMOX1,HSPA1A/HSPA1B,HSPA5,IL6ST,ILKAP,ITPR3,MCM10,MSTN,MTOR,NACC2,NR3C2,ODC1,PER1,PRKCB,PRKDC,SEPT4,SMOX,THBS1,TNFRSF1A,TNS2,TP53BP1,WT1

CELSR2,CEP192,CNTN4,COL16A1,COL17A1,COL24A1,COL27A1,COL6A6,COL7A1,CRTC1,CSF1R,CTSL,CUL7,CUL9,CYP51A1,DDR1,DNMT3B,DSP,E2F1,E2F2,EEF1A1,EGFR,EPHB3,EPHB4,F3,FASN,FAT1,HERC1,HMGA1,HMGCR,HMOX1,HSPA5,ILKAP,J
ADGRL2,AEBP2,AGRN,AKR1D1,ALOX12B,ALOX15B,ALOXE3,ANGPT2,ANXA5,APBA1,APOF,AQP12A/AQP12B,AQP3,AREG,ARHGAP26,ARHGAP32,ARHGEF9,ARNTL,ARNTL2,ARVCF,ATAD2,ATF3,ATR,ATRN,AUTS2,BAG2,BHLHE40,C4A/C4B,C6,CA1,CA2

1,SKI,SLC3A2,THBS1,TIMP2,WT1

LGMN,LTBP4,MMP19,MSTN,MTOR,MUL1,ODC1,PRSS36,SLC14A2,TIMP2,WFS1,WIZ

CELSR2,CEP192,CNTN4,COL16A1,COL17A1,COL24A1,COL27A1,COL6A6,COL7A1,CRTC1,CSF1R,CTSL,CUL7,CUL9,CYP51A1,DDR1,DNMT3B,DSP,E2F1,E2F2,EEF1A1,EGFR,EPHB3,EPHB4,F3,FASN,FAT1,HERC1,HMGA1,HMGCR,HMOX1,HSPA1A/HSPA1
BX7,CD109,CELSR2,CHRNB2,CHTF18,CISH,CNTN4,COL11A1,COL4A6,COL5A3,COL6A5,CPN2,CSF1R,CTSB,CUL9,CYP51A1,DENND4B,DKK3,DNMT3B,DPP6,DPT,DQX1,E2F1,E2F2,EGFR,EPHB3,EPHB4,FA2H,FASN,FBRSL1,FKBP10,FOSL1,GLI2,HERC1,
SD,CUL7,CUL9,CYFIP2,DHCR24,DKK3,E2F1,E2F2,EGFR,EPHB4,EYS,FANCA,FASN,FBXO32,GPX2,HMGA1,HMOX1,HSPA1A/HSPA1B,HSPA5,IL6ST,ILKAP,ITPR3,LRP6,MCM10,MEFV,MMS22L,MSTN,MTOR,NACC2,NR3C2,ODC1,PER1,PRKCB,PRKDC,SEP

ADGRL2,AEBP2,AGRN,AKR1D1,ALOX12B,ALOX15B,ALOXE3,ANGPT2,ANXA5,APBA1,APOF,AQP12A/AQP12B,AQP3,AREG,ARHGAP26,ARHGAP32,ARHGEF9,ARNTL,ARNTL2,ARVCF,ATAD2,ATF3,ATR,ATRN,AUTS2,BAG2,BHLHE40,C4A/C4B,C6,CA1,CA2

OL19A1,COL7A1,CREB5,CSF1R,CSRP3,CTSB,CTSD,CTSV,CUL7,CUL9,CYP26A1,CYP51A1,DDR1,DNAJB9,DNMT3B,DOT1L,DSP,E2F1,E2F2,ECE1,EGFR,EPHB3,F3,FASN,FAT1,FLCN,FOSL1,GLI2,GPX2,HM
CR,HMOX1,INSIG1,LSS,MTOR,NSDHL,PRKCB,PRKDC,PTX3,SQLE

IL6ST	LGMN	LRRC15	MSTN	MTSS1	NR3C2	PRKCB	PTX3	THBS1	TIMP2	TNFRSF1A WT1

P6,MAGI2,MKL1,MNX1,MSTN,NALCN,PHF21A,PTPRS,SALL3,SIK3,SKI,WNT9B,WT1

HMGCR	HMOX1	IL6ST	MKL1	MTOR	NR3C2	PRKCB

GAP2,STXBP5,TAOK1,THBS1,TNC,TNFRSF1A,TNIK,UNC119B,WISP2
SEPT4,SGK2,SLC3A2,SRGAP2,STXBP5,TAOK1,THBS1,TNC,TNFRSF1A,TNIK,UNC119B,WISP2

OU,SALL3,SEMA5A,SEPT4,SGK2,SLC3A2,SRGAP2,STXBP5,TAOK1,THBS1,TNC,TNFRSF1A,TNIK,UNC119B,WISP2

2,PPFIA4,PRKCB,PRKDC,PTCH1,SLX4,SVIL,THBS1,TIMP2,TNFRSF1A,TONSL,TP53BP1,WT1

FBXO32,FLCN,FOSL1,FSTL3,GLI2,HMGA1,HMOX1,HSP90B1,HSPA1A/HSPA1B,HSPA5,IL6ST,INSIG1,IRS1,JAG2,JARID2,JDP2,LRP4,LRP6,LTBP4,MAGI2,METRN,MKL1,MMP19,MNX1,MSTN,MTOR,NCOA1,NMRK2,NR3C2,OCSTAMP,ODC1,PER3,PIK3R2,PF

E2F2,EEF1A1,EGFR,EPHB3,EPHB4,F3,FA2H,FANCA,FASN,FBRS,FLCN,FOSL1,FSTL3,GLI2,GRB7,HMGA1,HMGCR,HMOX1,HSD11B2,HSP90B1,HSPA1A/HSPA1B,HSPA5,IL6ST,ILKAP,INSIG1,IRS1,ITPR3,JAG2,JARID2,JDP2,KIF26A,KLF11,KMT2C,KRT23,L

P4,LRP6,MKL1,MMP19,MNX1,MSTN,MTOR,NEO1,NR3C2,PER1,PRKCB,PRKDC,PTPRJ,PTPRS,RELN,SIK3,SKI,SLC14A1,SLC17A8,STEAP4,THBS1,TIMP2,TNC,TNFRSF1A,TP53BP1,WFS1,WT1,YBX2,ZNF385A

1,PRKCB,PTCH1,RELN,RORB,SLC5A5,THBS1,TNC,TNFRSF1A,UGP2,WISP2

C,PTCH1,SLX4,SVIL,THBS1,TIMP2,TNFRSF1A,TONSL,TP53BP1,WT1

FR,EPHB4,F3,FANCA,FASN,FBXO32,FLCN,FOSL1,FSTL3,GLI2,GPX2,HMGA1,HMGCR,HMOX1,HSD11B2,HSP90B1,HSPA1A/HSPA1B,HSPA5,IL6ST,ILKAP,IRS1,ITPR3,JAG2,KLF11,LGMN,LRP6,MCM10,MCM2,MEFV,MKL1,MNX1,MSTN,MTOR,NACC2,NCOA
DHCR24,DKK3,DNMT3B,DOT1L,DSP,E2F1,E2F2,ECE1,EEF1A1,EGFR,EPHB4,EYS,F3,FANCA,FASN,FBXO32,FLCN,FOSL1,FSTL3,GLI2,GPX2,GRB7,HBB,HMGA1,HMGCR,HMOX1,HSD11B2,HSP90B1,HSPA1A/HSPA1B,HSPA5,IL6ST,ILKAP,IRS1,ITPR3,JAG

HERC1,HMGA1,HMGCR,HMOX1,HSP90B1,HSPA5,ILKAP,JARID2,KMT2C,LRBA,LRP4,LRP6,MAGI2,MKL1,MSMO1,MTOR,NALCN,ODC1,PBRM1,PDIA6,PER3,PLEKHG3,POLE,PRKCB,PRKDC,RELN,RFPL4A/RFPL4AL1,RHOU,RORB,SALL3,SEMA5A,SGK2,SK

2,HMGA1,HMOX1,HSD11B2,HSP90B1,HSPA1A/HSPA1B,HSPA5,IL6ST,ILKAP,IRS1,ITPR3,LGMN,LRP6,MCM10,MCM2,MEFV,MKL1,MMS22L,MSTN,MTOR,NACC2,NCOA1,NEO1,NPTX1,NR3C2,ODC1,PAK6,PER1,PIK3R2,PLXNB1,PRKCB,PRKDC,PTCH1,SEF

ARID2,KMT2C,LRBA,LRP4,LRP6,MAGI2,MKL1,MSMO1,MTOR,NALCN,ODC1,PBRM1,PDIA6,PER3,PLEKHG3,POLE,PRKCB,PRKDC,RELN,RFPL4A/RFPL4AL1,RHOU,RORB,SALL3,SEMA5A,SGK2,SKI,STEAP4,STXBP5,SUZ12,SVEP1,TAOK1,THBS1,TP53BP1
B,HSPA5,ILKAP,JARID2,KMT2C,LRBA,LRP4,LRP6,MAGI2,MKL1,MSMO1,MTOR,NALCN,ODC1,PBRM1,PDIA6,PER3,PLEKHG3,POLE,PRKCB,PRKDC,RELN,RFPL4A/RFPL4AL1,RHOU,RORB,SALL3,SEMA5A,SGK2,SKI,STEAP4,STXBP5,SUZ12,SVEP1,TAOK
1,HSPA1A/HSPA1B,JAG2,KIF26A,KMT2C,LRBA,LTBP4,MKL1,MTOR,NR3C2,ODC1,OTOF,OTUD4,PCNX,PER1,PER3,POLE,PRSS36,PTCH1,RELN,RFPL1/RFPL3,RFPL4A/RFPL4AL1,RHBG,SALL3,SCN8A,SGK2,SRGAP2,SVEP1,THBS1,TNFRSF1A,TNRC18,T

HERC1,HMGA1,HMGCR,HMOX1,HSP90B1,HSPA1A/HSPA1B,HSPA5,ILKAP,JARID2,KMT2C,LRBA,LRP4,LRP6,MAGI2,MKL1,MSMO1,MTOR,NALCN,ODC1,PBRM1,PDIA6,PER3,PLEKHG3,POLE,PRKCB,PRKDC,RELN,RFPL4A/RFPL4AL1,RHOU,RORB,SALL3,
TNFRSF1A,TNS2,TP53BP1,WT1

HERC1,HMGA1,HMGCR,HMOX1,HSPA5,ILKAP,JARID2,KMT2C,LRBA,LRP4,LRP6,MAGI2,MKL1,MSMO1,MTOR,NALCN,ODC1,PBRM1,PDIA6,PER3,PLEKHG3,POLE,PRKCB,PRKDC,RELN,RFPL4A/RFPL4AL1,RHOU,RORB,SALL3,SEMA5A,SGK2,SKI,STEAP4
N,AUTS2,BAG2,BHLHE40,C4A/C4B,C6,CA1,CA2,CA3,CACNA1A,CAPN1,CARMIL3,CASKIN1,CBX7,CCDC169,CCDC173,CD109,CDC45,CELSR2,CEP192,CERS5,CHAC1,CHRNB2,CHTF18,CISH,CLASP1,CLIC2,CLOCK,CMBL,CNTN4,COL11A1,COL16A1,COL

HERC1,HMGA1,HMGCR,HMOX1,HSPA1A/HSPA1B,HSPA5,ILKAP,JARID2,KMT2C,LRBA,LRP4,LRP6,MAGI2,MKL1,MSMO1,MTOR,NALCN,ODC1,PBRM1,PDIA6,PER3,PLEKHG3,POLE,PRKCB,PRKDC,RELN,RFPL4A/RFPL4AL1,RHOU,RORB,SALL3,SEMA5A,S
2H,FASN,FBRSL1,FKBP10,FOSL1,GLI2,HERC1,HKDC1,HMCN1,HMGA1,HMOX1,HSD11B2,HSP90B1,HSPA1A/HSPA1B,JAG2,KIAA1109,KIF26A,KMT2C,LRBA,LTBP4,MKL1,MTOR,MTSS1,NCOA1,NPTX1,NR3C2,OTOF,OTUD4,PBRM1,PCNX,PER1,PER3,POL
IACC2,NR3C2,ODC1,PER1,PRKCB,PRKDC,SEPT4,SLC3A2,SLC5A5,SMOX,SVIL,THBS1,TNFRSF1A,TNS2,TP53BP1,WT1

N,AUTS2,BAG2,BHLHE40,C4A/C4B,C6,CA1,CA2,CA3,CACNA1A,CAPN1,CARMIL3,CASKIN1,CBX7,CCDC169,CCDC173,CD109,CDC45,CELSR2,CEP192,CERS5,CHAC1,CHRNB2,CHTF18,CISH,CLASP1,CLIC2,CLOCK,CMBL,CNTN4,COL11A1,COL16A1,COL

GCR,HMOX1,HSD11B2,HSP90B1,HSPA5,IL6ST,INSIG1,ITPR3,JAG2,KIF26A,KMT2C,LIAS,LMO7,LRP4,LRP6,MAGI2,MCM10,MCM2,MCM3AP,MKL1,MNX1,MSTN,MTOR,MTSS1,NALCN,NCOA1,NR3C2,PHF21A,

MRK2,NR3C2,OCSTAMP,ODC1,PER3,PIK3R2,PRKCB,PRKDC,PTCH1,PTPRJ,RELN,RLTPR,RORB,SALL3,SEMA5A,SHC4,SIK3,SKI,SLC3A2,SNX19,SRGAP2,SUZ12,THBS1,TIMP2,TMBIM1,TNC,TNFRSF1A,WFIKKN2,WISP2,WT1,ZNF385A

G2,JARID2,JDP2,KIF26A,KLF11,KMT2C,KRT23,LRP6,LTBP4,MAGI2,MCM10,MCM2,MKL1,MLLT6,MMP19,MNX1,MSTN,MTOR,MTSS1,MUL1,NACC2,NCOA1,NEO1,NPTX1,NR3C2,ODC1,PAK6,PBRM1,PER1,PIK3R2,PLXNB1,POC1B,PRKCB,PRKDC,PTCH1,PT

2,MEFV,MKL1,MNX1,MSTN,MTOR,NACC2,NCOA1,NPTX1,NR3C2,ODC1,PAK6,PER1,PIK3R2,PRKCB,PRKDC,PTCH1,SEPT4,SHC4,SKI,SLX4,SMOX,SUZ12,TAOK1,THBS1,TIMP2,TNC,TNFRSF1A,TNS2,TP53BP1,WFS1,WNK3,WT1,YBX2,ZNF385A

A/HSPA1B,HSPA5,IL6ST,ILKAP,IRS1,ITPR3,JAG2,KLF11,LGMN,LRP6,MCM10,MCM2,MEFV,MKL1,MMS22L,MNX1,MSTN,MTOR,NACC2,NCOA1,NEO1,NPTX1,NR3C2,ODC1,OLFM1,PAK6,PER1,PIK3R2,PLXNB1,PRKCB,PRKDC,PTCH1,SEPT4,SHC4,SKI,SLC

FPL4AL1,RHOU,RORB,SALL3,SEMA5A,SGK2,SKI,STEAP4,STXBP5,SUZ12,SVEP1,TAOK1,THBS1,TIMP2,TNIK,TP53BP1,WT1,ZMIZ1

ER1,PIK3R2,PLXNB1,PRKCB,PRKDC,PTCH1,SEPT4,SKI,SLC3A2,SLC5A5,SMOX,SVIL,THBS1,TIMP2,TNC,TNFRSF1A,TNS2,TP53BP1,WFS1,WNK3,WT1

STXBP5,SUZ12,SVEP1,TAOK1,THBS1,TP53BP1,WT1,ZMIZ1

GK2,SKI,STEAP4,STXBP5,SUZ12,SVEP1,TAOK1,THBS1,TP53BP1,WT1,ZMIZ1

K2,SRGAP2,SVEP1,THBS1,TNFRSF1A,TNRC18,TONSL,WDR90,YBX2

C,RELN,RFPL4A/RFPL4AL1,RHOU,RORB,SALL3,SEMA5A,SGK2,SKI,STEAP4,STXBP5,SUZ12,SVEP1,TAOK1,THBS1,TIMP2,TNIK,TP53BP1,WT1,ZMIZ1

RHOU,RORB,SALL3,SEMA5A,SGK2,SKI,STEAP4,STXBP5,SUZ12,SVEP1,TAOK1,THBS1,TIMP2,TNIK,TP53BP1,WT1,ZMIZ1

2,CLOCK,CMBL,CNTN4,COL11A1,COL16A1,COL17A1,COL19A1,COL21A1,COL24A1,COL27A1,COL28A1,COL4A6,COL5A3,COL6A5,COL6A6,COL7A1,CPAMD8,CPN2,CREB5,CRISPLD1,CRTC1,CRY1,CSF1R,CSRP3,CTSB,CTSD,CTSL,CTSV,CTTNBP2,CUL7

FPL4A/RFPL4AL1,RHOU,RORB,SALL3,SEMA5A,SGK2,SKI,STEAP4,STXBP5,SUZ12,SVEP1,TAOK1,THBS1,TIMP2,TNIK,TP53BP1,WT1,ZMIZ1

C2,OTOF,OTUD4,PBRM1,PCNX,PER1,PER3,POLE,PRKDC,PRSS36,PTCH1,PTPRS,RELN,RFPL1/RFPL3,RFPL4A/RFPL4AL1,RHBG,SALL3,SCN8A,SGK2,SRGAP2,SVEP1,THBS1,TNC,TNFRSF1A,TNRC18,TONSL,TP53BP1,TRRAP,VPS13B,WDR90,YBX2

2,CLOCK,CMBL,CNTN4,COL11A1,COL16A1,COL17A1,COL19A1,COL21A1,COL24A1,COL27A1,COL28A1,COL4A6,COL5A3,COL6A5,COL6A6,COL7A1,CPAMD8,CPN2,CREB5,CRISPLD1,CRTC1,CRY1,CSF1R,CSRP3,CTSB,CTSD,CTSL,CTSV,CTTNBP2,CUL7

,PRKDC,PTCH1,PTPRJ,PTPRS,PTX3,RPL24,SALL3,SEMA5A,SIK3,SKI,SLC14A1,SLC3A2,SLC4A10,SLX4,STXBP5,SUZ12,THBS1,TIMP2,TNFRSF1A,TP53BP1,TRRAP,WNT9B,WT1,ZMIZ1,ZNF385A

FIKKN2,WISP2,WT1,ZNF385A

K3R2,PLXNB1,POC1B,PRKCB,PRKDC,PTCH1,PTPRJ,PTPRS,PTX3,RELN,RHOU,RLTPR,SEMA5A,SEPT4,SGK2,SHC4,SKI,SLC3A2,SLX4,SMOX,SRGAP2,SUZ12,THBS1,TIMP2,TNC,TNFRSF1A,TNIK,TNS2,TP53BP1,TRRAP,WFIKKN2,WISP2,WNK1,WT1,YPE

P1,WFS1,WNK3,WT1,YBX2,ZNF385A

31,PRKCB,PRKDC,PTCH1,SEPT4,SHC4,SKI,SLC3A2,SLC5A5,SLX4,SMOX,SUZ12,SVIL,TAOK1,THBS1,TIMP2,TNC,TNFRSF1A,TNS2,TP53BP1,WFS1,WNK3,WT1,YBX2,ZNF385A

CSRP3,CTSB,CTSD,CTSL,CTSV,CTTNBP2,CUL7,CUL9,CYFIP2,CYP26A1,CYP27A1,CYP51A1,DDR1,DENND4B,DHCR24,DHRS1,DHX34,DIP2B,DKK3,DLEC1,DNAJB9,DNMT3B,DOCK4,DOT1L,DPP6,DPT,DQX1,DSP,E2F1,E2F2,EBP,ECE1,EEF1A1,EGFR,EM

NSL,TP53BP1,TRRAP,VPS13B,WDR90,YBX2

CSRP3,CTSB,CTSD,CTSL,CTSV,CTTNBP2,CUL7,CUL9,CYFIP2,CYP26A1,CYP27A1,CYP51A1,DDR1,DENND4B,DHCR24,DHRS1,DHX34,DIP2B,DKK3,DLEC1,DNAJB9,DNMT3B,DOCK4,DOT1L,DPP6,DPT,DQX1,DSP,E2F1,E2F2,EBP,ECE1,EEF1A1,EGFR,EM

53BP1,TRRAP,WFIKKN2,WISP2,WNK1,WT1,YPEL3,ZMIZ1

1,DSP,E2F1,E2F2,EBP,ECE1,EEF1A1,EGFR,EMC9,EMID1,EML6,EPHB3,EPHB4,EYS,F3,FA2H,FAM198B,FANCI,FASN,FAT1,FAT3,FBRSL1,FBXO32,FKBP10,FLCN,FNDC7,FOCAD,FOSL1,FSTL3,GARNL3,GLI2,GLTSCR1,GPX2,GRB7,GREB1L,HBB,HBD,HBE

1,DSP,E2F1,E2F2,EBP,ECE1,EEF1A1,EGFR,EMC9,EMID1,EML6,EPHB3,EPHB4,EYS,F3,FA2H,FAM198B,FANCI,FASN,FAT1,FAT3,FBRSL1,FBXO32,FKBP10,FLCN,FNDC7,FOCAD,FOSL1,FSTL3,GARNL3,GLI2,GLTSCR1,GPX2,GRB7,GREB1L,HBB,HBD,HBE

2,GLTSCR1,GPX2,GRB7,GREB1L,HBB,HBD,HBE1,HBG1,HBG2,HERC1,HKDC1,HMCN1,HMGA1,HMGCR,HMOX1,HPCAL1,HSD11B2,HSP90B1,HSPA1A

2,GLTSCR1,GPX2,GRB7,GREB1L,HBB,HBD,HBE1,HBG1,HBG2,HERC1,HKDC1,HMCN1,HMGA1,HMGCR,HMOX1,HPCAL1,HSD11B2,HSP90B1,HSPA1A
