# Supplementary material for: Waveband specific transcriptional control of select genetic pathways in vertebrate skin (Xiphophorus maculatus)
Source: BMC Genomics. 2018 May 10;19:355. doi: 10.1186/s12864-018-4735-5 (PMC5946439; doi:10.1186/s12864-018-4735-5)
Supplement: Supplementary file 2 — Table S2a–k. A list of all differentially modulated genes used by IPA enrichment software to predict the direction of change for each functional class represented in Additional file 1: Table S1. Table a is FL, tables b–e are the 50 nm wavebands and tables g–k are the 10 nm wavebands. (ZIP 701 kb) [file 12864_2018_4735_MOESM2_ESM.zip › TableS2h_510-520nm.pdf]

Functional Class	p-Value	Activation	# Genes	Genes
formation of cellular protrusions	7.73E-14	-5.734	92	ADGRF5 ADGRG6 AGRN ALS2 AQP1 ARHGAP32 ARHGEF9 ATF3 AVIL BCAS3 CALD1 CC2D2A CELSR2 CEP250 CHRNB2 CLSTN1 CNTNAP1 CNTNAP2 COL25A1 CUL7 DAB2IP DAG1 DDR1 DKK3 DOCK7 EGFR EIF2AK4 ELK1 EPHA3 EPHB3 EPHB6 ESPN FASN FGFR1 FGFR2 GATA3 HERC1 IFT172 ITGA1 ITGA6 KIF5B LAMA1 LAMB1
size of body	9.22E-05	-5.093	62	ABCA1 ACTC1 ADGRF5 ADGRG6 AKAP6 AQP1 ARHGAP35 ARL6IP5 ATM ATRN BRCA2 CACNA2D2 CBX7 CERK CHTF18 CNTNAP2 COL5A2 COL5A3 CSF1R CTSD CUL9 CYP24A1 DNMT3A E2F1 EGFR F3 FAT4 FGFR2 GLI2 GNAL HEYL HMOX1 HSPG2 IGFBP4 INSIG1 IRS1 ITPR3 LAMC1 MBNL1 MMP14 MSTN MYOD1 NCAM1
organization of cytoskeleton	1.12E-12	-4.406	122	AATK ABR ADGRF5 ADGRG6 AGRN ALS2 ANGPT2 APBB1IP AQP1 ARHGAP32 ARHGAP4 ARHGEF9 ATF3 AVIL BCAS3 CALD1 CAMSAP2 CBL CC2D2A CELSR2 CEP192 CEP250 CHRNB2 CLASP1 CLSTN1 CNTNAP1 CNTNAP2 COL25A1 CSF1R CTSV CUL7 CUL9 DAB2IP DAG1 DDR1 DKK3 DOCK4 DOCK7 DSP EGFR EIF2AK4 ELK1 EPHA3
quantity of neurons	7.36E-04	-4.401	32	ABCA1 AGRN ALS2 ANGPT2 ATM CACNA2D2 CHRNB2 CLSTN1 CNTFR CNTN5 CNTNAP2 CTSD E2F1 EGFR EIF2AK4 FGFR1 FGFR2 GLI2 HEYL HSPB8 JAG2 LRP4 NCAM1 NRG2 PTPRF PTPRS RBL1 RELN SLC17A8 SLC1A3 UNC13A WNT7B
organization of cytoplasm	3.84E-11	-4.283	126	AATK ABCA1 ABR ADGRF5 ADGRG6 AGRN ALS2 ANGPT2 APBB1IP AQP1 ARHGAP32 ARHGAP4 ARHGEF9 ATF3 AVIL BCAS3 BNIP3 CALD1 CAMSAP2 CBL CC2D2A CELSR2 CEP192 CEP250 CHRNB2 CLASP1 CLSTN1 CNTNAP1 CNTNAP2 COL25A1 CSF1R CTSV CUL7 CUL9 DAB2IP DAG1 DDR1 DKK3 DOCK4 DOCK7 DSP EGFR EIF2AK4
development of neurons	1.26E-09	-4.158	73	ADGRG6 ADGRL2 AGRN ALS2 ARHGAP32 ATF3 AVIL CACNA2D2 CALD1 CELSR2 CEP250 CHRNB2 CLSTN1 CNTNAP1 CNTNAP2 COL25A1 CUL7 DAB2IP DDR1 DOCK7 ECE1 EGFR EIF2AK4 ELK1 EPHA3 EPHB3 FGFR1 FGFR2 GLI2 GLI3 HERC1 IGSF9B ITGA1 LAMA1 LAMB1 LAMC1 LINGO1 LRP1 LRP4 MAGI2 MINK1 MNX1 MYO6
microtubule arrangement	2.54E-13	-4.105	110	AATK ADGRF5 ADGRG6 AGRN ALS2 ANGPT2 AQP1 ARHGAP32 ARHGAP4 ARHGEF9 ATF3 AVIL BCAS3 CALD1 CAMSAP2 CBL CC2D2A CELSR2 CEP192 CEP250 CHRNB2 CLASP1 CLSTN1 CNTNAP1 CNTNAP2 COL25A1 CSF1R CTSV CUL7 CUL9 DAB2IP DAG1 DDR1 DKK3 DOCK4 DOCK7 DSP EGFR EIF2AK4 ELK1 EPHA3 EPHB3 EPHB6
formation of plasma membrane projections	1.56E-10	-3.994	62	ADGRG6 AGRN ALS2 ARHGAP32 ATF3 AVIL CALD1 CELSR2 CHRNB2 CLSTN1 CNTNAP1 CNTNAP2 COL25A1 CUL7 DAB2IP DDR1 DOCK7 EGFR EIF2AK4 ELK1 EPHB3 FGFR1 FGFR2 HERC1 ITGA1 KIF5B LAMA1 LAMB1 LAMC1 LINGO1 LRP1 LRP4 MAGI2 MINK1 MNX1 MYO6 NCAM1 PAK6 PIP5K1B PLXNB1 PLXNB2 PPP1R9B PRKCA
formation of filopodia	1.29E-05	-3.944	19	AGRN ARHGEF9 BCAS3 DAG1 ESPN ITGA6 MTSS1 NEO1 PAK6 PLCG2 PPP1R9B PVR RELN RHOU SRGAP1 SRGAP2 SRGAP3 TENM1 TNC
assembly of intercellular junctions	8.77E-04	-3.942	22	ADGRL2 AGRN ALS2 CACNA2D2 CHRNB2 CLSTN1 DSP EPHA3 EPHB3 FGFR2 FLCN IGSF9B LAMC1 LRP4 MYO6 MYOD1 NCAM1 PARD3B PRKCA SLITRK3 TNC UNC13A
neuritogenesis	1.58E-10	-3.87	61	ADGRG6 AGRN ALS2 ARHGAP32 ATF3 AVIL CALD1 CELSR2 CHRNB2 CLSTN1 CNTNAP1 CNTNAP2 COL25A1 CUL7 DAB2IP DDR1 DOCK7 EGFR EIF2AK4 ELK1 EPHB3 FGFR1 FGFR2 HERC1 ITGA1 LAMA1 LAMB1 LAMC1 LINGO1 LRP1 LRP4 MAGI2 MINK1 MNX1 MYO6 NCAM1 PAK6 PIP5K1B PLXNB1 PLXNB2 PPP1R9B PRKCA PTPRF
developmental process of synapse	8.14E-04	-3.77	19	ADGRL2 AGRN ALS2 CACNA2D2 CHRNB2 CLSTN1 EPHA3 EPHB3 FGFR2 IGSF9B LAMC1 LRP4 MYO6 MYOD1 NCAM1 PRKCA SLITRK3 TNC UNC13A
formation of plasma membrane	5.90E-04	-3.729	23	ADGRL2 AGRN ALS2 CACNA2D2 CHRNB2 CLSTN1 CSF1R DOCK4 EPHA3 EPHB3 FGFR2 IGSF9B ITGA6 LAMC1 LRP4 MYO6 MYOD1 NCAM1 PARD3B PRKCA SLITRK3 TNC UNC13A
differentiation of cells	3.95E-13	-3.612	176	ADAMTS20 ADGRF5 AGRN ALOX15B ALOXE3 ANGPT2 AQP3 ARHGAP26 ARHGAP32 ARL11 ATF3 ATM BHLHE40 BNIP3 BRCA2 BRD8 CA2 CALD1 CBL CD109 CHRNB2 CITED1 CITED2 CLSTN1 CNTFR CNTNAP2 COL24A1 COL25A1 CREB5 CREG1 CSF1R CTSB CTSE CTSV CYB5D2 CYP26B1 CYTL1 DAB2IP DAG1 DDIT4 DDR1 DNMT3A DNMT3B
cell viability	2.63E-11	-3.61	176	ADAMTS20 ADGRF5 AGRN ALOX15B ALOXE3 ANGPT2 AQP3 ARHGAP26 ARHGAP32 ARL11 ATF3 ATM BHLHE40 BNIP3 BRCA2 BRD8 CA2 CALD1 CBL CD109 CHRNB2 CITED1 CITED2 CLSTN1 CNTFR CNTNAP2 COL24A1 COL25A1 CREB5 CREG1 CSF1R CTSB CTSE CTSV CYB5D2 CYP26B1 CYTL1 DAB2IP DAG1 DDIT4 DDR1 DNMT3A DNMT3B
morphogenesis of neurons	1.14E-08	-3.58	45	AGRN ALS2 ARHGAP32 ATF3 CALD1 CELSR2 CHRNB2 CLSTN1 CNTNAP1 CNTNAP2 COL25A1 CUL7 DAB2IP DOCK7 EGFR ELK1 EPHB3 FGFR1 HERC1 ITGA1 LAMA1 LAMB1 LAMC1 LINGO1 LRP4 MAGI2 MINK1 MNX1 NCAM1 PLXNB1 PTPRF RAPH1 RELN SDK2 SEMA3E SEMA3F SLC1A3 SLIT1 SRGAP2 SV2A TLR7 TNC TNIK
morphogenesis of neurites	1.95E-08	-3.58	44	AGRN ALS2 ARHGAP32 ATF3 CALD1 CELSR2 CHRNB2 CLSTN1 CNTNAP1 CNTNAP2 COL25A1 CUL7 DAB2IP DOCK7 EGFR ELK1 EPHB3 FGFR1 HERC1 ITGA1 LAMA1 LAMB1 LAMC1 LINGO1 LRP4 MAGI2 MINK1 MNX1 NCAM1 PLXNB1 PTPRF RAPH1 RELN SDK2 SEMA3E SEMA3F SLIT1 SRGAP2 SV2A TLR7 TNC TNIK WNT7B
morphogenesis of embryonic tissue	8.99E-05	-3.449	16	CITED1 CITED2 DCHS1 EGFR FAT4 GLI2 GLI3 HEYL ITGA8 JAG2 LRP4 MMP14 PTCH1 WNT7B WT1 ZFPM2
morphogenesis of embryo	3.00E-04	-3.449	22	CITED1 CITED2 CYP26B1 DCHS1 ECE1 EGFR FAT4 FGFR1 GLI2 GLI3 HEYL ITGA8 JAG2 LRP4 MMP14 PTCH1 SALL3 SIK3 SKI WNT7B WT1 ZFPM2
size of embryo	3.69E-04	-3.432	29	ARHGAP35 BRCA2 CITED2 CUL7 DAG1 DNMT3B DOT1L DSP ECE1 EGFR EPHA3 EXT1 F3 FGFR1 FGFR2 GLI2 HSPG2 KIF5B LIAS LIG1 MMP14 POLG PTCH1 RBL1 SFRP5 SUZ12 TSG101 XBP1 ZMIZ1
sprouting	3.48E-04	-3.373	31	AGRN ANGPT2 ARHGAP32 ATF3 CALD1 CHRNB2 CLSTN1 CNTNAP2 COL25A1 DAB2IP ELK1 EPHB3 EPHB4 FGFR1 HSPG2 IGFBP4 MAGI2 MMP14 NCAM1 PTPRF RAPH1 RELN SDK2 SEMA3E SEMA3F SEMA5A SLIT1 SV2A TLR7 TNC ZMIZ1
shape change of neurons	7.08E-04	-3.284	24	AGRN ARHGAP32 ATF3 CALD1 CHRNB2 CLSTN1 CNTNAP2 COL25A1 DAB2IP ELK1 EPHB3 GLI2 MAGI2 MNX1 NCAM1 PTPRF RAPH1 RELN SDK2 SEMA3E SEMA3F SLIT1 SV2A TLR7
morphogenesis of metanephric bud	5.02E-04	-3.236	9	CITED1 DCHS1 FAT4 GLI3 ITGA8 LRP4 MMP14 PTCH1 WT1
development of metanephric bud	1.04E-04	-3.236	11	CITED1 DCHS1 FAT4 FGFR1 FGFR2 GLI3 ITGA8 LRP4 MMP14 PTCH1 WT1
development of metanephros	1.09E-04	-3.236	12	CITED1 DCHS1 FAT4 FGFR1 FGFR2 GLI3 ITGA8 LRP4 MMP14 PTCH1 WNT7B WT1
differentiation of neuroblastoma cell lines	8.63E-04	-3.2	7	ARHGAP32 ATM EGFR NCAM1 NME1 SRGAP1 SRGAP2
differentiation of muscle	9.48E-05	-3.173	27	ANGPT2 ARHGAP26 EPHB4 EZH2 FGFR2 GLI3 HEYL HMOX1 ITGA6 JDP2 LAMC1 LDLR LGALS1 MBNL1 MSTN MYOD1 NCAM1 NMRK2 ODC1 OGN PLCB1 SEMA4C SKI SOCS1 SPAG9 WNT7B XBP1
differentiation of muscle cells	6.37E-05	-3.121	26	ANGPT2 EPHB4 EZH2 FGFR2 GLI3 HEYL HMOX1 ITGA6 JDP2 LAMC1 LDLR LGALS1 MBNL1 MSTN MYOD1 NCAM1 NMRK2 ODC1 OGN PLCB1 SEMA4C SKI SOCS1 SPAG9 WNT7B XBP1
development of genitourinary system	5.90E-05	-3.117	65	ABCA1 ADAMTS2 ADGRG2 ATM BRCA2 CBL CDH6 CEP131 CHTF18 CITED1 CITED2 CREM CSF1R CUL7 CYP26B1 DCHS1 DDR1 DNMT3A EGFR ERMP1 EZH2 FANCA FANCL FAT4 FGFR1 FGFR2 FOSL1 FSTL3 GADD45G GATA3 GLI2 GLI3 HEYL IRS1 ITGA1 ITGA6 ITGA8 JAG2 LAMC1 LRP4 LZTS2 MMP14 MSTN
size of animal	3.92E-04	-3.043	17	COL1A2 DDR1 DNMT3A DNMT3B EGFR HSPG2 IRS1 LAMC1 MNX1 MYOD1 PHC1 PHEX RBL1 RPL24 SKI STC1 TSG101
axonogenesis	1.94E-04	-3.043	21	ADGRG6 AGRN ALS2 ARHGAP32 ATF3 CHRNB2 COL25A1 DOCK7 EPHB3 FGFR2 LAMC1 LRP1 LRP4 NCAM1 PLXNB1 PLXNB2 PRKCA RELN SEMA3E SLIT1 SLITRK3
cell proliferation	2.18E-04	-2.986	72	ADARB1 ADGRL2 ALOX15B ARHGAP32 ARNTL2 ATF3 ATM ATR BRCA2 CALD1 CBL CBX7 CEP192 CEP250 CITED2 CLASP1 COL1A1 CSF1R CUL7 CUL9 DDIT4 DNMT3B DOT1L E2F1 EGFR ELK1 EZH2 FANCA FASN FGFR1 FGFR2 FIGNL1 FOSL1 GADD45G GATA3 HMOX1 IRS1 JARID2 JDP2 LIG1 MCM2 MLLT6 MMS22L
differentiation of nervous system	9.43E-05	-2.962	40	AGRN ARHGAP32 ATM CNTFR CYB5D2 DAG1 DDIT4 DNMT3B DOCK4 DOCK7 EGFR EXT1 FGFR1 FGFR2 GATA3 GLI2 GLI3 HEYL LAMC1 LINGO1 LRP4 MNX1 MSTN MYO6 NCAM1 NCOA1 PLXNB2 PPM1L PRKCA PTCH1 RELN RNH1 S1PR4 SALL3 SLC1A3 SPAG9 TDO2 TLR2 TNC ZNF536
differentiation of muscle cell lines	3.29E-04	-2.958	16	ARHGAP26 CTSB DDR1 EGLN3 FBXO32 FGFR2 IRS1 JDP2 KIF5B MSTN MYOD1 NMRK2 PLCB1 SCUBE3 SKI SOCS1
selection of cells	5.58E-04	-2.951	9	E2F1 ELK1 GATA3 GLI3 JAG2 LGALS1 MINK1 RELN TNC
cell movement	2.68E-08	-2.834	151	ABCA1 ABR ACO2 ACTC1 ADARB1 ADGRF5 ADGRG3 ALOX15B ANGPT2 ANXA5 AQP1 AQP3 ARHGAP35 ARHGAP4 ASAP2 ATF3 ATM BCAS3 BHLHE40 BRCA2 CAPN1 CBL CELSR2 CITED2 CNTNAP2 COL11A1 COL17A1 COL1A1 COL4A2 COL7A1 CSF1R CTSB CTSE CTSL CTSV CYP26B1 DAG1 DDIT4 DDR1 DKK3 DOCK10 DOCK4 DPT
differentiation of myoblasts	4.32E-04	-2.831	13	ANGPT2 GLI3 HMOX1 ITGA6 LGALS1 MBNL1 MSTN MYOD1 NCAM1 NMRK2 ODC1 PLCB1 SOCS1
development of exocrine gland	1.84E-04	-2.709	21	BRCA2 CBL CSF1R DDR1 EGFR EZH2 FGFR1 FGFR2 GATA3 GLI2 GLI3 HEYL IRS1 ITGA6 MMP14 NCOA1 NME1 PTCH1 PTPRF SOCS1 THRSP
gliomatosis	5.37E-06	-2.709	63	ANGPT2 AOX1 ARHGAP35 ATM ATR BRCA2 CBL CD109 CNTNAP2 COL11A1 COL14A1 COL1A1 COL1A2 COL4A2 COL5A3 COL6A5 CSF1R CSMD3 DENND4B DKK3 DMXL2 E2F1 EGFR ELK1 EPHA3 EPHB6 EZH2 FER1L6 FGFR1 FGFR2 GLI3 HERC1 HMCN1 HSPG2 KIAA1109 LAMB1 LDLR MMP14 NCAM1 NCOA1 NLRC5 OTUD4 PBRM1
formation of kidney	5.49E-05	-2.674	26	CDH6 CITED1 CITED2 CSF1R DCHS1 FAT4 FGFR1 FGFR2 GATA3 GLI2 GLI3 IRS1 ITGA1 ITGA8 LAMC1 LRP4 LZTS2 MMP14 MSTN ODC1 PTCH1 SIK3 SOCS1 SREBF1 WNT7B WT1
development of urinary tract	1.15E-05	-2.674	28	CDH6 CITED1 CITED2 CSF1R DCHS1 FAT4 FGFR1 FGFR2 GATA3 GLI2 GLI3 IRS1 ITGA1 ITGA8 LAMC1 LRP4 LZTS2 MMP14 MSTN ODC1 PTCH1 PTPRF PTPRS SIK3 SOCS1 SREBF1 WNT7B WT1
migration of cells	1.54E-07	-2.586	135	ABCA1 ABR ADARB1 ADGRF5 ADGRG3 ALOX15B ANGPT2 ANXA5 AQP1 AQP3 ARHGAP35 ARHGAP4 ASAP2 ATF3 ATM BCAS3 BHLHE40 BRCA2 CBL CELSR2 CITED2 CNTNAP2 COL11A1 COL17A1 COL1A1 COL4A2 COL7A1 CSF1R CTSB CTSE CTSL CTSV CYP26B1 DAG1 DDIT4 DDR1 DOCK10 DOCK4 DPT EGFR EGLN3 ELK1 EPHB3
growth of organism	4.23E-06	-2.566	66	ABCA1 ADARB1 ALOX15B AQP1 ATM BRCA2 CACNA2D2 CITED1 CITED2 COL4A2 CTSB CTSD CUL7 DAG1 DNAJB9 E2F1 EGFR EHHADH EIF2AK4 EZH2 F3 FGFR1 FGFR2 FOSL1 GATA3 GLI2 GLI3 HMOX1 HSPG2 IDO1 IGFBP4 IRS1 ITGA1 ITGA6 ITPR3 KIF26A KIF5B LAMA1 LGALS1 LIAS LIG1 LRP4 LZTS2
differentiation of neurons	3.12E-04	-2.543	33	AGRN ARHGAP32 ATM CNTFR CYB5D2 DDIT4 DNMT3B DOCK4 EGFR FGFR1 FGFR2 GATA3 GLI2 GLI3 HEYL LAMC1 LRP4 MNX1 MYO6 NCAM1 NCOA1 PLXNB2 PPM1L PRKCA PTCH1 S1PR4 SALL3 SLC1A3 SPAG9 TDO2 TLR2 TNC ZNF536
quantity of cells	1.12E-04	-2.519	114	ABCA1 ADGRF5 AGRN ALOX15B ALS2 ANGPT2 APOA4 AQP1 ARNTL2 ATF3 ATM BHLHE40 BRCA2 CACNA2D2 CAPN1 CBL CERK CHRNB2 CITED2 CLSTN1 CNTFR CNTN5 CNTNAP2 COL1A1 COL5A3 CREM CSF1R CTSB CTSD CTSE CTSV CUL7 CYP26B1 DDR1 DKK3 DNMT3B DOT1L DSP E2F1 EDA EGFR EIF2AK4 F3
development of abdomen	4.03E-05	-2.515	43	ATF3 ATM BRCA2 CDH6 CITED1 CITED2 CSF1R DCHS1 ERMP1 FANCA FANCL FAT4 FGFR1 FGFR2 GADD45G GATA3 GLI2 GLI3 HP IRS1 ITGA1 ITGA8 JARID2 LAMC1 LIG1 LRP4 LZTS2 MBTD1 MMP14 MNX1 MSTN ODC1 PRKDC PTCH1 RFX3 SIK3 SLC9A4 SOCS1 SREBF1 WNT7B WT1 XBP1 YBX2
proliferation of neuronal progenitor cells	1.63E-04	-2.48	10	ATM E2F1 EGFR FGFR1 LAMA1 NCAM1 PLXNB2 S1PR4 SEMA4C WNT7B
invasion of cells	8.70E-06	-2.468	67	ANGPT2 ARHGAP4 ATF3 BHLHE40 CA3 CBL CITED1 COL11A1 COL7A1 CTSB CTSD CTSL CTSV DDR1 DKK3 DNMT3B EGFR ELK1 EPHB4 EPHB6 EZH2 F3 FAT1 FGFR1 FGFR2 FOSL1 GATA3 GLI2 GNAL GRB7 HIC1 HMOX1 ITGA1 ITGA6 LAMA1 LAMC1 LGALS1 LRP1 LTB4R2 MIA MINK1 MMP14 NCOA1
fusion of cells	8.61E-05	-2.45	15	ANXA5 ARHGAP26 CTSB LAMA1 LDLR LGALS1 MMP14 MSTN MYOD1 NEO1 NMRK2 PLCB1 SH3PXD2A SLC3A2 TANC1
cardiogenesis	5.53E-05	-2.446	39	ACTC1 ANKRD1 ATM CITED2 COL11A1 COL5A1 CYP51A1 DCHS1 DSP ECE1 EGFR EPHA3 EPHB4 FAT4 FGFR1 FGFR2 FHOD3 GATA3 GLI2 GLI3 HEYL HMOX1 HSPB8 HSPG2 IFT172 MSTN PBRM1 PHC1 PLCB1 PRKCA PRKDC PTCH1 S1PR4 SOX18 TCAP WT1 XBP1 ZFPM2 ZMIZ1
development of body trunk	1.52E-05	-2.389	81	ABCA1 ACTC1 ADAMTS2 ADGRF5 ANKRD1 ATF3 ATM BRCA2 CBL CDH6 CITED1 CITED2 COL11A1 COL5A1 CSF1R CYP51A1 DCHS1 DSP ECE1 EGFR EPHA3 EPHB3 EPHB4 ERMP1 FANCA FANCL FAT4 FGFR1 FGFR2 FHOD3 GADD45G GATA3 GLI2 GLI3 HEYL HMOX1 HP HSPB8 HSPG2 IFT172 IRS1 ITGA1 ITGA6
cellular recombination	8.55E-09	-2.39	81	ABCA1 ACTC1 ADAMTS2 ADGRF5 ANKRD1 ATF3 ATM BRCA2 CBL CDH6 CITED1 CITED2 COL11A1 COL5A1 CSF1R CYP51A1 DCHS1 DSP ECE1 EGFR EPHA3 EPHB3 EPHB4 ERMP1 FANCA FANCL FAT4 FGFR1 FGFR2 FHOD3 GADD45G GATA3 GLI2 GLI3 HEYL HMOX1 HP HSPB8 HSPG2 IFT172 IRS1 ITGA1 ITGA6
proliferation of connective tissue cells	1.11E-06	-2.369	52	AQP1 ATF3 ATM ATR BRCA2 CBL CBX7 CD109 CITED2 COL14A1 COL1A1 CSF1R CTSB CTSD CTSV DAB2IP DKK3 E2F1 EGFR EZH2 FBRS FGFR1 FGFR2 FIGNL1 FOSL1 GATA3 GLI2 GLI3 HMOX1 IDO1 IGFBP4 IRS1 ITGA1 LGALS1 LIG1 MLLT6 MMP14 MSTN MYOD1 PLXNB1 PRKCA PRKDC PTCH1
growth of connective tissue	7.12E-07	-2.312	56	AQP1 ATF3 ATM ATR BRCA2 CBL CBX7 CD109 CITED2 COL14A1 COL1A1 CSF1R CTSB CTSD CTSV DAB2IP DKK3 E2F1 EGFR ELK1 EZH2 FBRS FGFR1 FGFR2 FIGNL1 FOSL1 GATA3 GFI1B GLI2 GLI3 HMOX1 IDO1 IGFBP4 IRS1 ITGA1 LGALS1 LIG1 MLLT6 MMP14 MSTN MYOD1 PIK3R2 PLXNB1
proliferation of cells	2.62E-10	-2.287	246	AATK ABCC5 ACER2 ADAMTS2 ADARB1 AGRN AKAP6 ALOX15B ALS2 ANGPT2 AQP1 ARHGAP32 ARL11 ARL6IP5 ARNTL2 ATAD2 ATF3 ATM ATR AVPR2 BHLHE40 BNIP3 BRCA2 C8orf4 CA3 CACNA2D2 CALB2 CALD1 CAPN1 CBL CBX7 CD109 CD33 CDC45 CDK20 CEP131 CERK CHRNB2 CITED1 CITED2 CLSTN1 CNOT6L CNTFR
phosphorylation of protein	1.66E-04	-2.261	54	AATK AGRN AKAP6 ATM ATR CBL CCDC88C CD109 CD33 COL5A3 CSF1R DAB2IP DDR1 EGFR EIF2AK4 EPHB3 EPHB4 EPHB6 EZH2 FGFR1 FGFR2 FLCN FNDC1 IRS1 ITGA6 LAMA1 LGALS1 LRP4 MARK4 MINK1 MYOD1 NME1 NME2 ODC1 PDK2 PKMYT1 PLXNB2 PPP1R9B PRKCA PRKDC PTPRF PTPRS RELN
migration of smooth cells	1.06E-03	-2.239	14	ANGPT2 ATF3 CBL CTSV DDR1 F3 FGFR1 HMOX1 IGFBP4 LDLR NEO1 PTPRF TLR2 WISP2
colony formation of fibroblast cell lines	1.14E-03	-2.195	10	ATR CBL CSF1R EGFR FGFR2 IRS1 MLLT6 PRKCA PTPRF SLC3A2
neuroepithelial tumor	1.77E-06	-2.184	65	ANGPT2 AOX1 ARHGAP35 ATM ATR BRCA2 CBL CD109 CNTNAP2 COL11A1 COL14A1 COL1A1 COL1A2 COL4A2 COL5A3 COL6A5 CSF1R CSMD3 CYP51A1 DENND4B DKK3 DMXL2 E2F1 EGFR ELK1 EPHA3 EPHB6 EZH2 FER1L6 FGFR1 FGFR2 GLI3 HERC1 HMCN1 HSPG2 KIAA1109 LAMB1 LDLR MMP14 NCAM1 NCOA1 NLRC5 OTUD4
oxidation of amino acids	3.16E-04	-2.172	5	IDO1 LPO MPO TDO2 TYR
double-stranded DNA break repair	9.53E-04	-2.166	12	ATM BRCA2 EGFR EZH2 LIG1 PBRM1 POLA1 PRKDC RIF1 SMARCC2 TP53BP1 TRRAP
transcription of DNA	1.04E-05	-2.163	106	AEBP2 AGRN ANKRD1 ARHGAP35 ARNTL2 ATAD2 ATF3 AUTS2 BCAS3 BHLHE40 BRCA2 BRD8 CBL CBX7 CELSR2 CIART CITED1 CITED2 COL1A1 COL4A2 CREB5 CREG1 CREM CYTL1 DAB2IP DBP DKK3 DNMT3A DNMT3B E2F1 EGFR ELK1 EZH2 FGFR1 FGFR2 FLCN FOSL1 FSTL3 GATA3 GFI1B GLI2 GLI3 HEYL
differentiation of embryonic cells	5.65E-04	-2.132	20	CBL CITED2 CYTL1 DOT1L EPHB4 EXT1 EZH2 FGFR1 FGFR2 FLCN GFI1B GLI3 HEYL MNX1 MTF2 MYOD1 RBL1 SKI SUZ12 TET1
mass of adipose tissue	8.46E-04	-2.13	17	ABCA1 APOA4 ATF3 E2F1 FSTL3 IGFBP4 INSIG1 IRS1 LDLR LPIN1 LRP1 MMP19 MSTN RBL1 SIK3 SREBF1 ZNF521
formation of mammary gland	2.78E-04	-2.109	19	BRCA2 CBL CSF1R DDR1 EGFR EZH2 FGFR2 GATA3 GLI2 GLI3 HEYL IRS1 MMP14 NCOA1 NME1 PTCH1 PTPRF SOCS1 THRSP
formation of gland	1.50E-04	-2.109	22	BRCA2 CBL CITED2 CSF1R DDR1 EDA EGFR EZH2 FGFR2 GATA3 GLI2 GLI3 HEYL IRS1 MMP14 NCOA1 NME1 PTCH1 PTPRF SOCS1 THRSP WT1
formation of muscle	5.85E-04	-2.085	31	ACTC1 ANKRD1 ANXA5 CACNA2D2 COL11A1 COL25A1 DDR1 FGFR1 FGFR2 FHOD3 HSPG2 ITGA8 LAMA1 MBNL1 MMP14 MNX1 MSTN MYOD1 NEO1 PLCB1 PLXNB1 SCN8A SLIT3 SVIL TANC1 TCAP TNNI2 WNT7B WT1 ZFAND5 ZFPM2
cell viability of muscle cells	1.11E-03	-2.078	8	ANGPT2 CSF1R FBXO32 HMOX1 HSPB8 LRP1 MYOD1 TLR2
proliferation of tumor cell lines	1.28E-03	-2.07	96	ALOX15B ANGPT2 AQP1 ARL11 ATAD2 ATF3 ATM ATR BNIP3 BRCA2 CA3 CBL CBX7 CITED1 COL14A1 COL1A1 COL4A2 CREG1 CREM CTSB CTSD CUL7 DKK3 DLEC1 DNMT3B E2F1 EGFR ELK1 EPHB6 EZH2 F3 FANCA FASN FGFR1 FGFR2 FOSL1 FSTL3 GADD45G GLI2 GNA15 GRB7 HEYL HIC1
limb development	3.39E-04	-2.067	21	COL1A2 CYP26B1 E2F1 ECE1 FGFR1 FGFR2 GLI2 GLI3 IFT172 ITGA6 LRP4 MMP14 MSTN PHC1 PTCH1 SALL3 SIK3 SKI SLC39A1 STC1 ZNF521
DNA repair	5.13E-04	-2.058	10	ATM BRCA2 EGFR EZH2 PBRM1 PRKDC RIF1 TLR2 TP53BP1 TRRAP
transcription of cells	3.77E-10	-2.044	128	AEBP2 AGRN ALOX12B ANKRD1 ARHGAP35 ARNTL2 ATAD2 ATF3 ATM AUTS2 AVPR2 BCAS3 BHLHE40 BRCA2 BRD8 C1QTNF1 CBL CBX7 CELSR2 CIART CITED1 CITED2 COL1A1 COL4A2 CREB5 CREG1 CREM CYP24A1 CYP26B1 CYTL1 DAB2IP DBP DKK3 DNMT3A DNMT3B DOT1L E2F1 EGFR EIF2AK4 ELK1 EZH2 FGFR1 FGFR2
transcription	5.82E-05	-2.044	128	AEBP2 AGRN ALOX12B ANKRD1 ARHGAP35 ARNTL2 ATAD2 ATF3 ATM AUTS2 AVPR2 BCAS3 BHLHE40 BRCA2 BRD8 C1QTNF1 CBL CBX7 CELSR2 CIART CITED1 CITED2 COL1A1 COL4A2 CREB5 CREG1 CREM CYP24A1 CYP26B1 CYTL1 DAB2IP DBP DKK3 DNMT3A DNMT3B DOT1L E2F1 EGFR EIF2AK4 ELK1 EZH2 FGFR1 FGFR2
adhesion of tumor cell lines	5.55E-04	-2.014	24	ALOX15B CDH24 DDR1 DKK3 DSP E2F1 EGFR FGFR2 IRS1 ITGA6 MINK1 MMP14 MMP19 MYO6 NCAM1 NECTIN2 NME1 NSD2 PLA2G10 PRKCA RHOU SEMA3F TLR2 TNC
generation of reactive oxygen species	9.22E-04	-2.004	18	ABCA1 ANGPT2 BNIP3 DDIT4 EGFR HMOX1 HP LTB4R2 MMP14 MSTN POLG PRKCA SLC25A38 SREBF1 STC1 TLR2 TLR7 XBP1
size of exocrine gland	2.16E-04	-2	6	CBX7 E2F1 EDA EGFR MMP14 XBP1
progression of tumor	3.09E-04	-2	16	COL11A1 COL1A1 COL1A2 EGFR EZH2 F3 FASN FGFR2 GATA3 IGFBP4 ITGA6 SEMA3E ST14 TLR2 TNC WNT7B
cognitive impairment	4.85E-04	-2	27	AUTS2 CA1 CA13 CA2 CA3 CC2D2A CUL7 DIP2B DNMT3A DPP6 FGFR1 FGFR2 GATAD2B GMPPA IQSEC2 MPO NLGN4X SATB2 SCN2B SCN8A SHROOM4 SKI SLC1A3 SMS SYNJ1 TNIK TTR
differentiation of epidermal cells	1.16E-03	2	15	ALOX15B AQP3 CD109 CTSE CTSV DSP EGFR FGFR2 GLI2 NME2 PTCH1 RBL1 SH3PXD2A ST14 TSG101
skin abnormality	2.50E-07	2	27	ADAMTS2 ALOX12B ALOXE3 COL11A1 COL17A1 COL1A1 COL1A2 COL5A1 COL5A2 COL7A1 DOCK6 DSP EDA EGFR EVC2 HMOX1 ITGA6 LDLR LTBP4 MMP14 OCA2 PRKCA ST14 TNXB TYR WT1 ZFPM2
multiple congenital anomalies	7.98E-06	2.015	38	ARHGAP35 ATM C5orf42 CBL CC2D2A COL11A1 COL1A1 COL9A3 CYP51A1 DCHS1 DNMT3B DOCK6 EGFR EPHB3 EVC2 EZH2 FAT4 FGFR1 FGFR2 GLI3 HSPG2 INSIG1 JAG2 LTBP4 MSTN PHC1 PIEZO2 PLCB1 SATB2 SCN2B SCN8A SEMA3E SKI SLC1A3 SMS TNNI2 VPS13B WT1
T-cell leukemia	3.13E-07	2.067	18	ATM ATR DNMT3A EZH2 GATA3 JARID2 LINGO1 POLA1 POLE RHOU SOCS1 SUZ12 TAX1BP1 TCF7 TET1 TLR7 WT1 ZMIZ1
lymphocytic leukemia	1.88E-07	2.067	51	ADGRL2 ARL6IP5 ATM ATR BRCA2 CDH10 CNTNAP1 CNTNAP2 COL17A1 COL1A1 COL1A2 COL27A1 COL7A1 CSF1R CUL9 DDR1 DNAH7 DNMT3A EPHB3 EZH2 F3 FANCL FAT4 FGFR1 GADD45G GATA3 IFI27 IL21R JARID2 LINGO1 LRP4 MAGI2 NALCN NOSTRIN PLCG2 POLA1 POLE PTPRD RHOU RIF1 SOCS1 SPAG9 SUZ12
Ovarian Cancer and Tumors	4.33E-06	2.091	61	ARHGAP35 ATM ATR BRCA2 CALB2 CDK20 CEP152 CLASP1 COL15A1 COL28A1 COL4A6 CREB5 CSF1R CUL9 CYFIP2 CYP24A1 DDR1 DEPDC5 DIP2B DNAH7 DNMT3A E2F1 EGFR EPHA3 EPHB6 ERAP2 EZH2 F3 FAT4 FGFR1 GATA3 GRB7 HIC1 HP IDO1 IFI27 IGFBP4 JAG2 KCNJ12 LIG1 LPO MMP14 NCAM1
tumorigenesis of gonad	9.69E-06	2.091	63	ARHGAP35 ATM ATR BRCA2 CALB2 CDK20 CEP152 CLASP1 COL15A1 COL28A1 COL4A6 CREB5 CSF1R CUL9 CYFIP2 CYP24A1 DDR1 DEPDC5 DIP2B DNAH7 DNMT3A E2F1 EGFR EPHA3 EPHB6 ERAP2 EZH2 F3 FAT4 FGFR1 GATA3 GRB7 HIC1 HP IDO1 IFI27 IGFBP4 JAG2 KCNJ12 LIG1 LPO LTB4R2 MMP14
gonadal tumor	1.57E-05	2.091	63	ARHGAP35 ATM ATR BRCA2 CALB2 CDK20 CEP152 CLASP1 COL15A1 COL28A1 COL4A6 CREB5 CSF1R CUL9 CYFIP2 CYP24A1 DDR1 DEPDC5 DIP2B DNAH7 DNMT3A E2F1 EGFR EPHA3 EPHB6 ERAP2 EZH2 F3 FAT4 FGFR1 GATA3 GRB7 HIC1 HP IDO1 IFI27 IGFBP4 JAG2 KCNJ12 LIG1 LPO LTB4R2 MMP14
female genital neoplasm	1.64E-13	2.091	254	ABI3BP ABR ACTC1 ADAMTS2 ADAMTSL1 ADAMTSL4 ADARB1 ADGRF1 ADGRF2 ADGRF4 ADGRF5 ADGRG2 AGAP6 (inc AKAP6 AMPD1 ANGPT2 ANKRD44 ANKRD50 ANXA5 APBB1IP AQP1 AQP12A/A(ARHGAP35 ARNTL2 ATAD2 ATM ATP2A3 ATR ATRN AVPR2 BPIFC BRCA2 BRD8 C5orf42 CA1 CA2 CALB2 CAMSAP2 CARMIL3 CBL CBX7 CC2D2A CCDC169
prostatic disease	1.07E-05	2.109	74	ABCA1 ACTC1 ADAMTS18 ADAMTS2 ADAMTS20 AGAP6 (inc ALOX15B ANAPC1 ANKRD1 BHLHE40 BRCA2 CA2 CALD1 CD33 CLSTN1 COL5A3 COL6A6 COL7A1 CYP24A1 CYP51A1 DAB2IP DDIT4 DENND4B DHX34 DMXL2 E2F1 EGFR EZH2 FASN FAT1 FCGBP FGFR1 FGFR2 GATA3 GLB1L2 GLI3 HMOX1 IDO1 IFI44L KIAA1109 LGALS1 LRBA LRP1
orofacial cleft	2.51E-04	2.203	12	CYP51A1 DNMT3B EGFR EPHB3 FGFR1 FGFR2 HSPG2 INSIG1 JAG2 MSTN PHC1 SATB2
skin tumor	6.66E-23	2.242	330	ABAT ABR ACSBG1 ACTC1 ADAMTS10 ADAMTS17 ADAMTS18 ADAMTS2 ADAMTS20 ADAMTSL4 ADGRF1 ADGRF2 ADGRF3 ADGRF4 ADGRF5 ADGRL2 AGRN AKAP6 AKR1D1 ALOX12B ALOX15B AMPD1 ANAPC1 ANXA5 AOX1 APBB1IP AQP1 ARHGAP26 ARL11 ARNTL2 ATAD2 ATM ATP2A3 ATR AUTS2 BAHCC1 BEGAIN BEST3 BPIFC BRCA2 BRD8 C8orf4 CA1
formation of skin	9.13E-06	2.249	34	ADAMTS2 ALOX15B AQP3 CD109 COL17A1 COL1A1 COL1A2 COL5A1 COL5A2 COL7A1 CSF1R CTSE CTSL CTSV CYP26B1 DSP E2F1 EDA EGFR EXT1 FGFR2 GLI2 IFT172 ITGA1 ITGA6 LRP4 NME2 PTCH1 RBL1 SH3PXD2A SOCS1 SOX18 ST14 TSG101
necrosis	3.33E-06	2.291	166	AATK ACER2 ACO2 ACTC1 ADARB1 ADGRL2 AGRN ALS2 ANGPT2 ANKRD1 ARHGAP4 ARL11 ATAD2 ATF3 ATM ATR BCAS2 BHLHE40 BNIP3 BRCA2 C8orf4 CA3 CACNA2D2 CAPN1 CBL CCDC86 CD33 CDC45 CDH6 CERK CERS5 CERS6 CITED1 CLASP1 CNTFR COL1A1 COL25A1 COL4A2 COL5A3 CREM CSF1R CTSB CTSD
micrognathia	1.34E-04	2.294	9	CYP26B1 CYP51A1 ECE1 FGFR1 MMP14 ORC1 PTPRF PTPRS SATB2
tumorigenesis of genital organ	8.75E-18	2.316	289	ABCA1 ABI3BP ABR ACTC1 ADAMTS18 ADAMTS2 ADAMTS20 ADAMTSL1 ADAMTSL4 ADGRF1 ADGRF2 ADGRF4 ADGRF5 ADGRG2 AGAP6 (inc AKAP6 AMPD1 ANAPC1 ANGPT2 ANKRD1 ANKRD44 ANKRD50 ANXA5 APBB1IP AQP1 AQP12A/A(ARHGAP35 ARNTL2 ATAD2 ATM ATP2A3 ATR ATRN AVPR2 BHLHE40 BPIFC BRCA2 BRD8 C5orf42 CA1 CA2 CALB2 CALD1
genital tumor	1.42E-16	2.316	292	ABCA1 ABI3BP ABR ACTC1 ADAMTS18 ADAMTS2 ADAMTS20 ADAMTSL1 ADAMTSL4 ADARB1 ADGRF1 ADGRF2 ADGRF4 ADGRF5 ADGRG2 AGAP6 (inc AKAP6 AMPD1 ANAPC1 ANGPT2 ANKRD1 ANKRD44 ANKRD50 ANXA5 APBB1IP AQP1 AQP12A/A(ARHGAP35 ARNTL2 ATAD2 ATM ATP2A3 ATR ATRN AVPR2 BHLHE40 BPIFC BRCA2 BRD8 C5orf42 CA1 CA2 CALB2
thoracic cancer	1.27E-04	2.334	75	ABCC5 ADGRF1 ADGRF4 AGRN ANAPC1 ARHGAP35 ARL11 ATF3 ATM ATR BRCA2 CACNA2D1 CACNA2D2 CBL CBX7 CDH10 CNTN5 CNTNAP2 COL11A1 COL22A1 CSF1R CSMD3 CTSD CYP24A1 DDR1 DLEC1 DMXL2 DNAJB12 DNMT3A E2F1 EGFR EPHA3 EPHB4 EPHB6 EZH2 F3 FASN FAT1 FCGBP FGFR1 FGFR2 GATA3 GLB1L2
cell death	1.58E-07	2.376	214	AATK ABCA1 ABCC5 ACER2 ACO2 ACTC1 ADAMTS20 ADAMTSL4 ADARB1 ADGRL2 AGRN ALOX15B ALS2 ANGPT2 ANKRD1 ANXA5 AQP1 AQP3 ARHGAP35 ARHGAP4 ARL11 ARL6IP5 ATAD2 ATF3 ATM ATR BCAS2 BHLHE40 BNIP3 BRCA2 C8orf4 CA3 CACNA2D2 CAPN1 CBL CBX7 CCDC86 CD33 CDC45 CDH6 CERK CERS5 CERS6
congenital anomaly of digestive system	2.65E-04	2.384	21	ARHGAP35 CITED2 CYP51A1 DNMT3B EDA EGFR EPHB3 FGFR1 FGFR2 GLI2 GMPPA HSPG2 INSIG1 JAG2 LTBP4 MMP14 MSTN PHC1 RBL1 RFX3 SATB2
angiogenesis of tumor	3.19E-04	2.391	13	ATM CBL CTSB EGFR F3 FGFR2 ITGA1 LGALS1 LRP1 MMP14 PAK6 WNT7B WT1
tumorigenesis of reproductive tract	5.21E-14	2.4	248	ABI3BP ABR ACTC1 ADAMTS2 ADAMTSL1 ADAMTSL4 ADGRF1 ADGRF2 ADGRF4 ADGRF5 ADGRG2 AGAP6 (inc AKAP6 AMPD1 ANGPT2 ANKRD44 ANKRD50 ANXA5 APBB1IP AQP1 AQP12A/A(ARHGAP35 ARNTL2 ATAD2 ATM ATP2A3 ATR ATRN AVPR2 BPIFC BRCA2 BRD8 C5orf42 CA1 CA2 CALB2 CAMSAP2 CARMIL3 CBX7 CC2D2A CCDC169 CCDC18 CD109
pelvic tumor	6.24E-21	2.408	324	AATK ABCA1 ABI3BP ABR ACTC1 ADAMTS17 ADAMTS18 ADAMTS2 ADAMTS20 ADAMTSL1 ADAMTSL4 ADGRF1 ADGRF2 ADGRF4 ADGRF5 ADGRG2 AGAP1 AGAP6 (inc AKAP6 ALOX15B AMPD1 ANAPC1 ANGPT2 ANKRD1 ANKRD44 ANKRD50 ANXA5 APBB1IP AQP1 AQP12A/A(ARHGAP32 ARHGAP35 ARNTL2 ATAD2 ATF3 ATM ATP2A3 ATR ATRN AVPR2 BCAS3 BHLHE40 BICRA
congenital anomaly of mouth	3.73E-04	2.423	16	CYP51A1 DNMT3B EDA EGFR EPHB3 FGFR1 FGFR2 GLI2 HSPG2 INSIG1 JAG2 MMP14 MSTN PHC1 RBL1 SATB2
congenital malformation of genitourinary system	3.18E-04	2.497	28	ANGPT2 AVPR2 C5orf42 CALD1 CC2D2A DBP E2F1 EGFR EPHB3 EVC2 FGFR2 GADD45G GLI2 GLI3 HMOX1 ITGA8 LAMC1 LTBP4 LZTS2 MAGI2 MMP14 NCOA1 PLCD4 SLC1A3 SLIT3 SOCS1 WT1 ZFPM2
benign neoplasia	4.94E-09	2.511	81	ACTC1 ADARB1 AGRN ANGPT2 ANXA5 ARHGAP35 ATM ATR ATRN BRCA2 CA1 CA2 CALB2 CBL CBX7 COL11A1 COL14A1 COL15A1 COL16A1 COL17A1 COL1A1 COL1A2 COL21A1 COL22A1 COL24A1 COL25A1 COL27A1 COL4A2 COL4A6 COL5A1 COL5A2 COL5A3 COL7A1 COL8A1 COL9A3 CSF1R CTSB CUL9 DNMT3A DNMT3B DPT E2F1 EGFR
congenital malformation of face	3.12E-06	2.541	19	C5orf42 CYP51A1 DNMT3B EGFR EPHB3 FGFR1 FGFR2 GLI2 HSPG2 IFT172 INSIG1 JAG2 MSTN NALCN PHC1 POLE PTCH1 SATB2 ZSWIM6
apoptosis	5.15E-09	2.627	183	AATK ABCA1 ACO2 ACTC1 ADAMTS20 ADAMTSL4 ADGRL2 AGRN ALOX15B ALS2 ANGPT2 ANKRD1 ANXA5 AQP1 AQP3 ARHGAP35 ARHGAP4 ARL11 ARL6IP5 ATAD2 ATF3 ATM ATR BCAS2 BHLHE40 BNIP3 BRCA2 C8orf4 CA3 CACNA2D2 CAPN1 CBL CCDC86 CD33 CDC45 CERS6 CHTF18 CITED1 CITED2 CLASP1 CNTFR COL1A1 COL25A1
dysplasia	7.23E-07	2.709	25	ADAMTS10 ADAMTS2 COL11A1 COL1A1 COL1A2 COL5A1 COL5A2 COL9A3 E2F1 EGFR EXT1 FGFR1 FGFR2 GATA3 GLI3 HSPG2 IFT172 ITGA8 LAMA1 LAMC1 PRKDC RBL1 TLR7 TNXB ZSWIM6
lymphoproliferative disorder	4.64E-08	2.709	72	ADGRL2 ARL6IP5 ATM ATR BRCA2 CCDC86 CDH10 CELSR2 CNTNAP1 CNTNAP2 COL17A1 COL1A1 COL1A2 COL27A1 COL4A2 COL7A1 CSF1R CUL9 DDR1 DNAH7 DNMT3A DNMT3B DSP E2F1 EGFR EPHA3 EPHB3 EZH2 F3 FANCL FAT4 FGFR1 GADD45G GATA3 IFI27 IL21R JARID2 LINGO1 LRP4 MAGI2 MCM5 NALCN NOSTRIN
congenital anomaly of digit	4.01E-04	2.741	13	CYP26B1 CYP51A1 FGFR1 FGFR2 GLI3 IFT172 ITGA6 JAG2 LRP4 MSTN PTCH1 SKI SMS
hyperactive behavior	6.86E-04	2.873	14	ABR CBL CHRNB2 CNTNAP2 CREM DKK3 FGFR1 GNAL LDLR LRP1 PER1 PLCB1 PTPRF SLC17A8
congenital anomalies of kidney and urinary tract	5.72E-04	2.941	21	ANGPT2 AVPR2 C5orf42 CALD1 CC2D2A DBP EGFR EPHB3 EVC2 FGFR2 GLI3 HMOX1 ITGA8 LAMC1 LZTS2 MAGI2 MMP14 PLCD4 SLC1A3 SLIT3 SOCS1
head and neck cancer	2.19E-06	2.96	83	ACTC1 ADAMTS10 ADAMTS2 ALOX15B ANGPT2 AOX1 ARHGAP35 ATM ATR BRCA2 CBL CBX7 CD109 COL11A1 COL14A1 COL15A1 COL1A1 COL5A3 COL6A5 CPXM2 CSF1R CSMD3 CYP26B1 CYP51A1 DENND4B DKK3 DNMT3A DPT E2F1 EGFR ELK1 EPHA3 EPHB4 EPHB6 EZH2 FASN FCGBP FER1L6 FGFR1 FGFR2 GATA3 GLI2 GLI3
pancreatic tumor	5.55E-09	2.988	100	ABI3BP ADAMTS10 ADAMTS2 ADAMTSL1 AMPD1 ARHGAP32 ARHGAP4 ATM BNIP3 BRCA2 BRD8 C5orf42 CALD1 CASKIN1 CBL CDH9 CNTN5 CNTNAP2 COL1A1 COL1A2 COL22A1 COL6A5 COL7A1 CSF1R CSMD3 CTSB CTSD CTSV CUL7 CUL9 DCHS1 DENND4B DIP2B DIP2C DNAH7 DOCK10 DOCK4 DSCAML1 EGFR EHHADH F3 FAM114A1 FASN
endocrine gland tumor	3.30E-08	3.062	119	ADAMTS10 ADAMTS18 ADAMTSL4 ADARB1 ADGRF3 AGAP6 (inc AGRN ALOX15B ANAPC1 ANGPT2 ANO9 ANXA5 APBB1IP AQP12A/A(ARHGAP4 ATM BAHCC1 BEGAIN BRD8 CA13 CBX7 CCDC18 CELSR2 CEP131 CHRNB2 CHTF18 CNTNAP2 COL11A1 COL4A6 COL9A3 CPN2 CSF1R CSMD3 CTSB CTSV CUL9 CYP24A1 DCHS1 DMXL2 DNMT3B DPP6 E2F1 EGFR
skin lesion	1.83E-23	3.145	334	ABAT ABR ACSBG1 ACTC1 ADAMTS10 ADAMTS17 ADAMTS18 ADAMTS2 ADAMTS20 ADAMTSL4 ADGRF1 ADGRF2 ADGRF3 ADGRF4 ADGRF5 ADGRL2 AGRN AKAP6 AKR1D1 ALOX12B ALOX15B AMPD1 ANAPC1 ANXA5 AOX1 APBB1IP AQP1 ARHGAP26 ARL11 ARNTL2 ATAD2 ATM ATP2A3 ATR AUTS2 BAHCC1 BEGAIN BEST3 BPIFC BRCA2 BRD8 C8orf4 CA1
midline defect	5.53E-05	3.212	25	ARHGAP35 BRCA2 CITED2 CYP51A1 DNMT3B ECE1 EGFR EPHB3 FGFR1 FGFR2 HSPG2 IFT172 INSIG1 JAG2 LAMC1 MSTN PHC1 PLXNB2 PTCH1 PTPRF PTPRS RGMA SATB2 SKI ZFPM2
hematological neoplasia	1.57E-09	3.227	145	ABAT ABCA1 ADAMTS17 ADGRL2 AGAP6 (inc AKAP6 ALOX12B ANXA5 ARHGAP26 ARHGAP35 ARL6IP5 ATM ATR ATRN BAG2 BRCA2 CALD1 CARMIL3 CBL CBX7 CCDC86 CD109 CD33 CDH10 CELSR2 CEP192 CHRNA9 CNTFR CNTNAP1 CNTNAP2 COL16A1 COL17A1 COL1A1 COL1A2 COL24A1 COL27A1 COL4A2 COL5A2 COL6A6 COL7A1 CSF1R CTSL CUL7
head and neck neoplasia	1.76E-11	3.385	170	ACTC1 ADAMTS10 ADAMTS18 ADAMTS2 ADAMTSL4 ADARB1 ADGRF3 AGAP6 (inc AGRN ALOX15B ANAPC1 ANGPT2 ANO9 ANXA5 AOX1 APBB1IP AQP12A/A(ARHGAP35 ARHGAP4 ATM ATR BAHCC1 BEGAIN BRCA2 BRD8 CA13 CBL CBX7 CCDC18 CD109 CELSR2 CEP131 CHRNB2 CHTF18 CNTNAP2 COL11A1 COL14A1 COL15A1 COL1A1 COL4A6 COL5A3 COL6A5 COL9A3
lymphoid cancer	1.72E-08	3.456	128	ABAT ABCA1 ADAMTS17 ADGRL2 AGAP6 (inc AKAP6 ARHGAP26 ARHGAP35 ARL6IP5 ATM ATR BAG2 BRCA2 CARMIL3 CBL CCDC86 CD109 CD33 CDH10 CELSR2 CEP192 CHRNA9 CNTNAP1 CNTNAP2 COL16A1 COL17A1 COL1A1 COL1A2 COL24A1 COL27A1 COL4A2 COL5A2 COL6A6 COL7A1 CSF1R CUL7 CUL9 CYP51A1 DDR1 DNAH7 DNMT3A DNMT3B DSP
hematologic cancer	2.54E-10	3.457	144	ABAT ABCA1 ADAMTS17 ADGRL2 AGAP6 (inc AKAP6 ALOX12B ANXA5 ARHGAP26 ARHGAP35 ARL6IP5 ATM ATR ATRN BAG2 BRCA2 CALD1 CARMIL3 CBL CBX7 CCDC86 CD109 CD33 CDH10 CELSR2 CEP192 CHRNA9 CNTFR CNTNAP1 CNTNAP2 COL16A1 COL17A1 COL1A1 COL1A2 COL24A1 COL27A1 COL4A2 COL5A2 COL6A6 COL7A1 CSF1R CTSL CUL7
seizure disorder	4.96E-05	3.562	39	ABAT ABCC5 ADARB1 ARHGEF9 ATF3 BHLHE40 CA1 CA13 CA2 CA3 CACNA2D1 CACNA2D2 CHRNB2 CITED2 CNTNAP2 CREM CTSB CTSD DBP DEPDC5 DOCK7 E2F1 GADD45G GLI3 HMOX1 LDLR LINGO1 MCM6 NCAM1 PLCB1 POLG PPP1R9B SCN2B SCN8A SEMA3F SLC17A8 SLC1A3 SV2A SYNJ1
Lymphoid Cancer and Tumors	5.04E-08	3.65	130	ABAT ABCA1 ADAMTS17 ADGRL2 AGAP6 (inc AKAP6 ARHGAP26 ARHGAP35 ARL6IP5 ATM ATR BAG2 BRCA2 CARMIL3 CBL CCDC86 CD109 CD33 CDH10 CELSR2 CEP192 CHRNA9 CNTNAP1 CNTNAP2 COL16A1 COL17A1 COL1A1 COL1A2 COL24A1 COL27A1 COL4A2 COL5A2 COL6A6 COL7A1 CSF1R CUL7 CUL9 CYP51A1 DDR1 DNAH7 DNMT3A DNMT3B DSP
craniofacial abnormality	8.12E-09	3.665	40	ARHGAP35 ATR BRCA2 C5orf42 CBL CEP152 CITED2 CYP26B1 CYP51A1 DCHS1 DNMT3B ECE1 EGFR EPHB3 FAT4 FGFR1 FGFR2 GLI2 GLI3 HSPG2 IFT172 INSIG1 JAG2 LAMC1 MMP14 MSTN NALCN ORC1 PHC1 PLXNB2 POLE PTCH1 PTPRF PTPRS RGMA SATB2 SKI SMS WDR62 ZSWIM6
cell death of tumor cell lines	2.23E-04	3.685	99	AATK ACER2 ACO2 ADGRL2 ALS2 ANGPT2 ANKRD1 ARHGAP4 ARL11 ATAD2 ATF3 ATM ATR BCAS2 BHLHE40 BNIP3 BRCA2 CACNA2D2 CAPN1 CERK CERS5 CERS6 CLASP1 COL1A1 COL4A2 CREM CSF1R CTSB CTSD CUL7 CUL9 CYFIP2 DDIT4 DKK3 DNMT3A E2F1 EGFR EGLN3 EIF2AK4 ELK1 EPHB4 EPHB6 EZH2
congenital malformation of skeleton	5.38E-07	3.691	41	ARHGAP35 ATR BRCA2 C5orf42 CBL CEP152 CITED2 CYP26B1 CYP51A1 DCHS1 DNMT3B ECE1 EGFR EPHB3 FAT4 FGFR1 FGFR2 GLI2 GLI3 HSPG2 IFT172 INSIG1 JAG2 LAMC1 MBTD1 MMP14 MSTN NALCN ORC1 PHC1 PLXNB2 POLE PTCH1 PTPRF PTPRS RGMA SATB2 SKI SMS WDR62 ZSWIM6
apoptosis of tumor cell lines	4.17E-04	3.898	80	AATK ACO2 ADGRL2 ALS2 ANGPT2 ANKRD1 ARHGAP4 ARL11 ATAD2 ATF3 ATM ATR BCAS2 BHLHE40 BNIP3 BRCA2 CACNA2D2 CAPN1 CERS6 CLASP1 COL1A1 COL4A2 CREM CSF1R CTSB CTSD CUL9 CYFIP2 DDIT4 DKK3 E2F1 EGFR EIF2AK4 EPHB4 EPHB6 EZH2 FANCA FASN FBXO32 FGFR1 FGFR2 GADD45G GFI1B
congenital malformation of brain	1.94E-04	3.919	26	ARHGAP35 ATR BRCA2 CACNA2D2 CCDC88C CEP152 CITED2 CNTNAP2 FGFR1 GLI2 HSPG2 IFT172 LAMA1 LAMB1 LAMC1 ORC1 PHC1 PIK3R2 PLXNB2 PTCH1 PTPRF PTPRS RELN RGMA SKI WDR62
neonatal death	4.86E-05	3.941	37	ABR AKAP6 ALOX12B ALOXE3 ATM CITED1 CNTFR COL5A2 CREB5 CUL7 CYP26B1 ECE1 EHHADH F3 FAT4 FGFR2 GLI2 JAG2 LAMC1 LRP4 MAGI2 MBTD1 MMP14 MNX1 MYOD1 NALCN PHF21A POU2F2 PTPRS RBL1 SALL3 SIK3 ST14 SV2A SYNJ1 UNC13A WT1
congenital anomaly of musculoskeletal system	3.54E-07	3.982	63	ADAMTS10 ADAMTS2 ALS2 ARHGAP35 ATR BRCA2 C5orf42 CALD1 CBL CEP152 CITED2 COL11A1 COL1A1 COL1A2 COL4A2 COL5A1 COL5A2 COL9A3 CYP26B1 CYP51A1 DAG1 DCHS1 DNMT3B ECE1 EGFR EPHB3 EXT1 FAT4 FGFR1 FGFR2 GLI2 GLI3 HSPG2 IFT172 INSIG1 ITGA6 JAG2 LAMC1 LRP4 MBNL1 MBTD1 MMP14 MSTN
dysgenesis	7.29E-05	4.557	43	ATM BRCA2 CACNA2D2 CBL CITED2 CTSD CYP26B1 CYP51A1 E2F1 ECE1 EGFR FGFR1 FGFR2 GLI2 HMOX1 ITGA6 ITGA8 LAMA1 LAMC1 LDLR LIG1 LRP4 MBTD1 MMP14 MYOD1 NCOA1 ORC1 PBRM1 PHC1 PLCG2 PRKDC PTPRF PTPRS RELN SATB2 SKI SLIT3 TP53BP1 TYR WNT7B WT1 XBP1 ZFPM2
Hypoplasia	1.27E-04	4.622	40	ATM BRCA2 CACNA2D2 CBL CTSD CYP26B1 CYP51A1 E2F1 ECE1 EGFR FGFR1 FGFR2 GLI2 ITGA6 ITGA8 LAMA1 LAMC1 LDLR LIG1 LRP4 MBTD1 MMP14 MYOD1 NCOA1 ORC1 PBRM1 PHC1 PLCG2 PRKDC PTPRF PTPRS RELN SATB2 SKI SLIT3 TP53BP1 WNT7B WT1 XBP1 ZFPM2
Growth Failure	1.24E-04	5.216	46	ABCA1 ADGRG6 AQP1 ATM BRCA2 CACNA2D2 CHRNB2 CITED1 CSF1R CTSB CTSV CUL7 DCHS1 DDR1 DNMT3A DOT1L EGFR EHHADH EIF2AK4 EZH2 F3 FGFR1 FGFR2 HMOX1 IRS1 ITGA6 ITPR3 KIF26A LAMA1 LIG1 LRP1 MMP14 ORC1 PHC1 POLG PTPRS RBL1 RFX3 SFRP5 SOCS1 SOX13 SV2A SYNJ1
perinatal death	9.65E-10	5.403	60	ABCA1 ABR AGRN AKAP6 ALOX12B ALOXE3 ARHGAP35 ATM BAHCC1 CITED1 CITED2 CNTFR COL5A2 CREB5 CUL7 CYP26B1 DCHS1 E2F1 ECE1 EGFR EHHADH EPHA3 EVC2 F3 FAT4 FGFR2 GLI2 HMOX1 HSPG2 ITGA6 JAG2 LAMC1 LRP4 MAGI2 MBTD1 MMP14 MNX1 MSTN MTF2 MYOD1 NALCN PHC1 PHF21A
organismal death	4.72E-11	6.4	177	ABCA1 ABR ACTC1 ADARB1 ADGRF5 ADGRG6 AGRN AKAP6 ALOX12B ALOXE3 ANGPT2 AQP1 ARHGAP35 ATF3 ATM ATR BAHCC1 BRCA2 CACNA2D2 CAPN1 CBL CCDC86 CDC45 CERK CHRNB2 CHTF18 CITED1 CITED2 CNTFR CNTNAP1 COL11A1 COL1A1 COL25A1 COL4A2 COL5A1 COL5A2 COL7A1 CREB5 CREM CSF1R CTSB CTSD CTSV

LAMC1 LINGO1 LRP1 LRP4 MAGI2 MARK4 MINK1 MNX1 MTSS1 MYO6 NCAM1 NEO1 NME1 PAK6 PIP5K1B PLCG2 PLXNB1 PLXNB2 PPP1R9B PRKCA PTK7 PTPDC1 PTPRF PVR RAPH1 RELN RFX3 RHOU SDK2 SEMA3E SEMA3F 4-Sep SLC9A5 SLIT1 SLIT3 SLITRK3 SRGAP1 SRGAP2 SRGAP3 SV2A TEKT3 TENM1 TLR7 TNC TNIK UNC119B WISP2 WNT7B ZSWIM6
NRG2 PER1 PHC1 PLCB1 PLCG2 PPP1R9B PTCH1 PTPRS RBL1 RFX3 RHCG SEMA3F SIK3 SOCS1 ST14 SV2A TET1 TP53BP1 UNC79
EPHB3 EPHB6 ESPN F3 FASN FAT1 FGFR1 FGFR2 GATA3 HERC1 IFT172 ITGA1 ITGA6 ITGA8 KIF5B LAMA1 LAMB1 LAMC1 LDLR LIG1 LINGO1 LPIN1 LRP1 LRP4 LTB4R MAGI2 MARK4 MAST1 MIA MINK1 MNX1 MTSS1 MYO6 NCAM1 NECTIN2 NEO1 NME1 PAK6 PIP5K1B PLCG2 PLXNB1 PLXNB2 PPP1R9B PRKCA PRKDC PTK7 PTPDC1 PTPRF PVR RAPH1

ELK1 EPHA3 EPHB3 EPHB6 ESPN F3 FASN FAT1 FGFR1 FGFR2 GATA3 HERC1 IFT172 ITGA1 ITGA6 ITGA8 KIF5B LAMA1 LAMB1 LAMC1 LDLR LIG1 LINGO1 LPIN1 LRP1 LRP4 LTB4R MAGI2 MARK4 MAST1 MIA MINK1 MNX1 MTSS1 MYO6 NCAM1 NECTIN2 NEO1 NME1 PAK6 PIP5K1B PLCG2 PLXNB1 PLXNB2 PPP1R9B PRKCA PRKDC PTK7 PTPDC1 PTPRF
MYOD1 NCAM1 PAK6 PIP5K1B PLXNB1 PLXNB2 PPP1R9B PRKCA PTPRF RAPH1 RELN SDK2 SEMA3E SEMA3F 4-Sep SLC1A3 SLC9A5 SLIT1 SLIT3 SLITRK3 SRGAP2 SV2A TLR7 TNC TNIK UNC13A WISP2 WNT7B XBP1 ZSWIM6
ESPN FASN FGFR1 FGFR2 GATA3 HERC1 IFT172 ITGA1 ITGA6 ITGA8 KIF5B LAMA1 LAMB1 LAMC1 LDLR LINGO1 LRP1 LRP4 LTB4R MAGI2 MARK4 MIA MINK1 MNX1 MTSS1 MYO6 NCAM1 NEO1 NME1 PAK6 PIP5K1B PLCG2 PLXNB1 PLXNB2 PPP1R9B PRKCA PTK7 PTPDC1 PTPRF PVR RAPH1 RELN RFX3 RHOU SALL3 SDK2 SEMA3E SEMA3F SEMA5A 4-Sep
PTPRF RAPH1 RELN SDK2 SEMA3E SEMA3F 4-Sep SLC9A5 SLIT1 SLIT3 SLITRK3 SRGAP2 SV2A TLR7 TNC TNIK WISP2 WNT7B ZSWIM6

RAPH1 RELN SDK2 SEMA3E SEMA3F 4-Sep SLC9A5 SLIT1 SLIT3 SLITRK3 SRGAP2 SV2A TLR7 TNC TNIK WISP2 WNT7B ZSWIM6

DOCK4 DOCK7 DOT1L DSP E2F1 EGFR EGLN3 EIF2AK4 ELK1 EPHB3 EPHB4 EVC2 EXT1 EZH2 FASN FBXO32 FGFR1 FGFR2 FIGNL1 FLCN FOSL1 FSTL3 GADD45G GATA3 GFI1B GLI2 GLI3 GNA15 GNAL HEYL HMOX1 HSPG2 IKZF4 IL21R INSIG1 IRS1 ITGA6 JAG2 JARID2 JDP2 KIF5B LAMC1 LDLR LGALS1 LINGO1 LPIN1 LRP1 LRP4 LTBP4 MAGI2
DOCK4 DOCK7 DOT1L DSP E2F1 EGFR EGLN3 EIF2AK4 ELK1 EPHB3 EPHB4 EVC2 EXT1 EZH2 FASN FBXO32 FGFR1 FGFR2 FIGNL1 FLCN FOSL1 FSTL3 GADD45G GATA3 GFI1B GLI2 GLI3 GNA15 GNAL HEYL HMOX1 HSPG2 IKZF4 IL21R INSIG1 IRS1 ITGA6 JAG2 JARID2 JDP2 KIF5B LAMC1 LDLR LGALS1 LINGO1 LPIN1 LRP1 LRP4 LTBP4 MAGI2
WNT7B ZSWIM6
ZSWIM6

NCOA1 NECTIN2 NME1 OCA2 ODC1 PBRM1 PTCH1 PTP4A2 PTPRF PTPRS 4-Sep SIK3 SOCS1 SPAG9 SREBF1 STRBP THRSP TTLL5 WNT7B WT1 YBX2 ZFPM2

MYOD1 NME1 PBRM1 PER1 PER3 PHC1 PIK3R2 PKMYT1 PLA2G10 POLA1 PPP1R9B PRKCA PTCH1 PTPRF PTX3 RBL1 RHOU SEMA3F SESN1 SKI SOCS1 TLR2 TNC TRRAP TSG101 TTC28 WT1 WWC1 XBP1

EGFR EGLN3 ELK1 EPHB3 EPHB4 EZH2 F3 FASN FAT1 FAT3 FGFR1 FGFR2 FLCN FOSL1 GATA3 GLI2 GLI3 GRB7 HIC1 HMOX1 HP IDO1 IGFBP4 IL21R IRS1 ITGA1 ITGA6 ITGA8 JAG2 KIF5B LAMA1 LAMB1 LAMC1 LDLR LGALS1 LRP1 LTB4R LTB4R2 MAGI2 MCM2 MCM3 MIA MINK1 MMP14 MMP19 MNX1 MPO MTSS1 MYO6 NCAM1

PLCB1 POLA1 PTK7 PTPRD PTX3 RAPH1 RELN SEMA5A 4-Sep SHC4 SIGLEC10 SKI SLC17A9 SLC9A4 SYNJ1 TNC TRRAP VPS13B WT1 ZNF536

EPHB4 EZH2 F3 FASN FAT1 FAT3 FGFR1 FGFR2 FLCN FOSL1 GATA3 GLI2 GLI3 GRB7 HIC1 HMOX1 HP IDO1 IGFBP4 IL21R IRS1 ITGA1 ITGA6 ITGA8 LAMA1 LAMB1 LAMC1 LDLR LGALS1 LRP1 LTB4R LTB4R2 MAGI2 MCM2 MCM3 MIA MINK1 MMP14 MMP19 MNX1 MPO NCAM1 NEO1 NME1 NME2 NSD2 OSGIN1 PAK6 PER1 PIK3R2
MCM10 NCAM1 NCOA1 NRG2 PLXNB2 PTCH1 PTP4A2 RBL1 S1PR4 SEMA4C SIK3 SLC1A3 SOCS1 STC1 SYNJ1 TDO2 TLR2 TNC TP53BP1 TRRAP TSG101 UNC79 WNT7B

FANCA FANCL FGFR1 FGFR2 FSTL3 GADD45G GATA3 GLI2 HEYL HMOX1 HSPB8 HSPG2 IDO1 IL21R IRS1 ITGA1 ITGA6 JAG2 JARID2 LAMC1 LDLR LGALS1 LIG1 LRP4 LTB4R MBTD1 MMP14 MMP19 MNX1 MPO MSTN MYOD1 NCAM1 NEO1 NLRC5 NRG2 PER1 PHC1 PLA2G10 PLCG2 POU2F2 PRKDC PTPRF PTPRS RBL1 RELN RHO SIGLEC1 SIGLEC10 SIGLEC8

NME1 NME2 NSD2 ODC1 PAK6 PIK3R2 PIP5K1B PLXNB1 PRKCA PTP4A2 RNH1 SATB2 SCN8A SCUBE3 SEMA3F SOCS1 ST14 SUZ12 TLR2 TNC WBP1L WISP2 WNK1 WNT7B

ITGA8 JARID2 LAMC1 LIG1 LRP4 LZTS2 MBTD1 MMP14 MNX1 MSTN ODC1 PBRM1 PHC1 PLCB1 PRKCA PRKDC PTCH1 PTPRS RBL1 RFX3 RLTPR S1PR4 SIK3 SLC23A1 SLC9A4 SOCS1 SOX18 SREBF1 TCAP TCF7 TLR2 TYR WNT7B WT1 XBP1 YBX2 ZFPM2 ZMIZ1
ITGA8 JARID2 LAMC1 LIG1 LRP4 LZTS2 MBTD1 MMP14 MNX1 MSTN ODC1 PBRM1 PHC1 PLCB1 PRKCA PRKDC PTCH1 PTPRS RBL1 RFX3 RLTPR S1PR4 SIK3 SLC23A1 SLC9A4 SOCS1 SOX18 SREBF1 TCAP TCF7 TLR2 TYR WNT7B WT1 XBP1 YBX2 ZFPM2 ZMIZ1
PTPRF RBL1 SFRP5 SKI SLC3A2 SOCS1 STC1 TSG101 ZMIZ1
PRKCA PRKDC PTCH1 PTPRF RBL1 SFRP5 SKI SLC3A2 SOCS1 STC1 TSG101 WT1 ZMIZ1
COL14A1 COL1A1 COL1A2 COL4A2 COL8A1 CREG1 CREM CSF1R CSMD3 CTSB CTSD CTSL CTSV CUL7 CYP24A1 DAB2IP DCHS1 DDIT4 DDR1 DKK3 DLEC1 DNMT3B DOCK4 DOCK7 DOT1L DPT DSP E2F1 EGFR EGLN3 EIF2AK4 ELK1 EPHB3 EPHB4 EPHB6 EXT1 EZH2 F3 FANCA FANCL FASN FAT4 FBRS FGFR1 FGFR2 FIGNL1 FLCN FOSL1 FSTL3 GABARAPL
RGMA RHO SIK3 SLC3A2 SOCS1 TLR7 TNIK WNK1 WNK3 WNK4 XBP1

PBRM1 PLCB1 POLA1 PTK7 PTPRD PTX3 RAPH1 RBL1 RELN SEMA5A 4-Sep SHC4 SIGLEC10 SKI SLC17A9 SLC9A4 SYNJ1 TNC TRRAP VPS13B WT1 ZNF536

HIC1 IFI27 IFT172 IKZF4 ITGA6 JARID2 JDP2 KLF11 LPIN1 MED13 MLLT6 MMS19 MNX1 MSTN MTF2 MYO6 MYOD1 NACC2 NCOA1 NEO1 NLRC5 NME2 NOSTRIN NR1D2 NSD2 PAK6 PBRM1 PER1 PER3 PHF21A PIK3R2 PLCB1 POU2F2 PRKDC PTCH1 RAD54L2 RBL1 RFX3 RORB SATB2 SCAF8 SKI SMARCC2 SOX18 SPIN1 SREBF1 SUZ12 TCF7 TENM1 TET1

HMOX1 HSPB8 IDO1 IGFBP4 ILKAP IRS1 ITGA1 ITGA6 LGALS1 LIG1 LRP1 MCM2 MIA MMP14 MMP19 MSTN MYOD1 NACC2 NCAM1 NCOA1 NEO1 NME1 NME2 NRG2 NSD2 ODC1 OSGIN1 PHC1 PLCD4 PLCG2 PLXNB1 PPP1R9B PRKCA PTCH1 PTPRF PTPRR RBL1 SKI SLC3A2 SLIT3 SMOX SOCS1 SREBF1 SUZ12 TLR2 TNIK TNXB TRRAP TSG101 TYR

FLCN FOSL1 FSTL3 GADD45G GATA3 GFI1B GLI2 GLI3 HEYL HIC1 IFI27 IFT172 IKZF4 ITGA6 JAG2 JARID2 JDP2 KLF11 LDLR LGALS1 LPIN1 LRP1 MAGI2 MED13 MLLT6 MMS19 MNX1 MSTN MTF2 MYO6 MYOD1 NACC2 NCAM1 NCOA1 NEO1 NLRC5 NME1 NME2 NOSTRIN NR1D2 NSD2 PAK6 PBRM1 PER1 PER3 PHF21A PIK3R2 PLCB1 POLA1 POU2F2
FLCN FOSL1 FSTL3 GADD45G GATA3 GFI1B GLI2 GLI3 HEYL HIC1 IFI27 IFT172 IKZF4 ITGA6 JAG2 JARID2 JDP2 KLF11 LDLR LGALS1 LPIN1 LRP1 MAGI2 MED13 MLLT6 MMS19 MNX1 MSTN MTF2 MYO6 MYOD1 NACC2 NCAM1 NCOA1 NEO1 NLRC5 NME1 NME2 NOSTRIN NR1D2 NSD2 PAK6 PBRM1 PER1 PER3 PHF21A PIK3R2 PLCB1 POLA1 POU2F2

SVEP1 TAX1BP1 TCF7 TET1 TLR2 TLR7 WT1 ZMIZ1
OCA2 OTUD4 PBRM1 PEAR1 PIP5K1B POLE PRKDC SCUBE3 SEMA4C SIGLEC12 SIPA1L2 ST14 STC1 TPM4 TSG101 TTR WISP2 ZFPM2
NCAM1 OCA2 OTUD4 PBRM1 PEAR1 PIP5K1B POLE PRKDC SCUBE3 SEMA4C SIGLEC12 SIPA1L2 ST14 STC1 TPM4 TSG101 TTR UPP1 WISP2 ZFPM2
NCAM1 OCA2 OTUD4 PBRM1 PEAR1 PIP5K1B POLE PRKDC SCUBE3 SEMA4C SIGLEC12 SIPA1L2 ST14 STC1 TPM4 TSG101 TTR UPP1
CCDC18 CD109 CDH10 CDK20 CEP131 CEP152 CHRNA9 CHRNB2 CLASP1 CLNK CNTN5 CNTNAP2 COL14A1 COL15A1 COL17A1 COL28A1 COL4A2 COL4A6 COL5A1 CPAMD8 CREB5 CREM CSF1R CSMD3 CTSB CTSL CTSV CUL9 CYFIP2 CYP24A1 DCHS1 DDR1 DEPDC5 DHRS1 DIP2B DLEC1 DMXL2 DNAH7 DNMT3A DOCK10 DOCK4 DOT1L DPP6 DPT DSP E2F1 EFHB EGFR EPHA3 EPHB6
LRP4 LZTS2 MAST1 MCM2 MCM5 MXRA5 NCAM1 NCOA1 NME1 ODC1 OTUD4 PAK6 PER1 PLXNA2 PLXNB1 PTK7 RBL1 RHCG SIGLEC1 SIGLEC12 SLC7A6 SMS SOCS1 SRGAP1 SVEP1 THRAP3 TLR2 TRRAP TTR WDR62 WT1

CA3 CACNA2D1 CACNA2D2 CALB2 CARMIL3 CASKIN1 CBL CC2D2A CCDC88C CD109 CD33 CDH10 CDH6 CDH9 CEP131 CEP192 CEP250 CERS6 CHD6 CHMP4C CHRNA9 CHRNB2 CLSTN1 CNTFR CNTN5 CNTNAP1 CNTNAP2 COL11A1 COL14A1 COL15A1 COL17A1 COL1A1 COL1A2 COL21A1 COL22A1 COL24A1 COL25A1 COL27A1 COL28A1 COL4A2 COL5A1 COL5A2 COL5A3 COL6A5 COL6A6 COL7A1 COL8A1 CPAMD8 CPN2 CPXM2

CTSV CUL7 CUL9 CYFIP2 DAB2IP DAG1 DDIT4 DKK3 DNMT3A DNMT3B DSP E2F1 EGFR EGLN3 EIF2AK4 ELK1 EPHB4 EPHB6 EPX EZH2 F3 FAM134B FANCA FANCL FASN FBXO32 FGFR1 FGFR2 FOSL1 FSTL3 GADD45G GATA3 GFI1B GLI2 GLI3 GNAL HEYL HIC1 HMOX1 HSPB8 IDO1 IGFBP4 IL21R ILKAP IRS1 ITGA1 ITGA6 ITPR3 LDLR LGALS1

CAMSAP2 CARMIL3 CBX7 CC2D2A CCDC169 CCDC18 CD109 CD33 CDH10 CDK20 CEP131 CEP152 CHRNA9 CHRNB2 CLASP1 CLNK CLSTN1 CNTN5 CNTNAP2 COL14A1 COL15A1 COL17A1 COL28A1 COL4A2 COL4A6 COL5A1 COL5A3 COL6A6 COL7A1 CPAMD8 CREB5 CREM CSF1R CSMD3 CTSB CTSL CTSV CUL9 CYFIP2 CYP24A1 CYP51A1 DAB2IP DCHS1 DDIT4 DDR1 DENND4B DEPDC5 DHRS1 DHX34 DIP2B
CALD1 CAMSAP2 CARMIL3 CBL CBX7 CC2D2A CCDC169 CCDC18 CD109 CD33 CDH10 CDK20 CEP131 CEP152 CHRNA9 CHRNB2 CLASP1 CLNK CLSTN1 CNTN5 CNTNAP2 COL14A1 COL15A1 COL17A1 COL28A1 COL4A6 COL5A1 COL5A3 COL6A6 COL7A1 CPAMD8 CREB5 CREM CSF1R CSMD3 CTSB CTSL CTSV CUL9 CYFIP2 CYP24A1 CYP51A1 DAB2IP DCHS1 DDIT4 DDR1 DENND4B DEPDC5 DHRS1
GLB1L3 ITPR3 LAMB4 LGALS1 LIG1 LRP1 LZTS2 MCM2 MMP14 MMP19 MPO MXRA5 NCAM1 NCOA1 NME1 ODC1 PBRM1 PLXNB1 POLE POU2F2 PRKDC SCUBE3 SLITRK3 SMARCC2 SPIN4 ST14 TP53BP1 TSG101 TTR WNK1 WT1 ZNF536
CHTF18 CITED1 CITED2 CLASP1 CLNK CNTFR COL1A1 COL25A1 COL4A2 COL5A3 CREM CSF1R CTSB CTSD CTSE CTSL CTSV CUL7 CUL9 CYFIP2 CYP26B1 DAB2IP DAG1 DDIT4 DDR1 DKK3 DNMT3A DNMT3B DOT1L DSP E2F1 ECE1 EDA EGFR EGLN3 EIF2AK4 ELK1 EPHB4 EPHB6 EPX EZH2 F3 FAM134B FANCA FANCL FASN FAT4 FBXO32 FGFR1 FGFR2

CDH10 CDK20 CEP131 CEP152 CHRNA9 CHRNB2 CLASP1 CLNK CNTN5 CNTNAP2 COL14A1 COL15A1 COL17A1 COL28A1 COL4A2 COL4A6 COL5A1 CPAMD8 CREB5 CREM CSF1R CSMD3 CTSB CTSL CTSV CUL9 CYFIP2 CYP24A1 DCHS1 DDR1 DEPDC5 DHRS1 DIP2B DLEC1 DMXL2 DNAH7 DNMT3A DOCK10 DOCK4 DOT1L DPP6 DPT DSP E2F1 EFHB EGFR EPHA3 EPHB6 EPX ERAP2
BPIFC BRCA2 BRD8 C5orf42 CA1 CA2 CALB2 CALD1 CAMSAP2 CARMIL3 CBX7 CC2D2A CCDC169 CCDC18 CD109 CD33 CDH10 CDK20 CEP131 CEP152 CEP192 CHRNA9 CHRNB2 CHTF18 CLASP1 CLNK CLSTN1 CNTN5 CNTNAP2 COL14A1 COL15A1 COL17A1 COL28A1 COL4A2 COL4A6 COL5A1 COL5A3 COL6A6 COL7A1 CPAMD8 CPXM2 CREB5 CREM CSF1R CSMD3 CTSB CTSE CTSL CTSV CYFIP2

EPHA3 EPHB3 EPHB6 EXT1 EZH2 FGFR1 FGFR2 FKBP10 GATA3 GLI2 HMOX1 HP IFI44L IGFBP4 ITGA6 JAG2 LGALS1 LIG1 MIA MMP14 MXRA5 NCAM1 ODC1 PBRM1 PLXNB2 PRKCA PTCH1 RELN RORB SLC24A3 SLIT3 TLR2 TNC TNNI2 TYR UGP2 WISP2 ZFPM2

COL4A2 COL5A3 CREM CSF1R CTSB CTSD CTSE CTSL CTSV CUL7 CUL9 CYFIP2 CYP26B1 DAB2IP DAG1 DDIT4 DDR1 DKK3 DNMT3A DNMT3B DOT1L DSP E2F1 ECE1 EDA EGFR EGLN3 EIF2AK4 ELK1 EPHB4 EPHB6 EZH2 F3 FAM134B FANCA FASN FAT4 FBXO32 FGFR1 FGFR2 FIGNL1 FLCN FNDC1 FOSL1 FSTL3 GADD45G GATA3 GFI1B GLI2 GLI3

NSD2 PLCG2 PLEKHG3 POLA1 POLE POU2F2 PPP6R2 PRKDC PTPRD RELN RHOU RIF1 SALL3 SEMA5A SIGLEC10 SLC9A5 SOCS1 SPAG9 SUZ12 SVEP1 TAX1BP1 TCF7 TET1 TLR2 TLR7 TP53BP1 VWA7 WT1 ZMIZ1

HERC1 HMCN1 HSPG2 ITGA6 ITGA8 KIAA1109 LDLR LGALS1 MMP14 MTSS1 NCAM1 NCOA1 NLRC5 NME1 OTUD4 PBRM1 PEAR1 PLCB1 POLA1 POLE PRKDC PTCH1 PTK7 PTPRD PTPRS RAPH1 RBL1 SIGLEC1 SIGLEC10 SLC17A9 SLC9A4 SOCS1 SPON1 TLR2 TNC TNXB TP53BP1 TRRAP TSG101 VPS13B
FAT1 FAT3 FGFR1 FGFR2 FIGNL1 FKBP10 GLB1L2 GLI3 HERC1 HMCN1 HP IDO1 KLF11 LAMA1 LAMB1 LGALS1 LRBA LRMP LRP1 LZTS2 MAGI2 MCM5 MEFV MMP14 NALCN NOSTRIN PBRM1 PFAS POLE PRKCA PRKDC PRR12 PTCH1 RAD54L2 RASGRF2 SCAF8 SEMA3E SEMA5A SGIP1 SIGLEC1 SIGLEC7 SIK3 SLC15A2 SLC17A9 SLITRK3 SMOX SOCS1 SPON1 SRGAP1 SVIL
ELK1 EPHB3 EPHB4 EZH2 FBRSL1 FCGBP FER1L6 FGFR1 FGFR2 FKBP10 FNDC1 FOSL1 GLI3 GNAL HMCN1 HMOX1 HP HYI IGSF9B JAG2 KCNJ12 KIF26A LAMC1 LRBA LTBP4 LZTS2 MAN1C1 MIA MMP14 NCAM1 NTN5 ODC1 OTOF OTUD4 PARD3B PCNX1 PER1 PER3 PHC1 PLXNA2 PLXNB2 POLE PRKCA PRSS36 PTCH1 PTPRF RAB6B RBL1 RELN RFPL1/RFF
CA3 CACNA2D1 CACNA2D2 CALB2 CARMIL3 CASKIN1 CBL CC2D2A CCDC88C CD109 CD33 CDH10 CDH6 CDH9 CEP131 CEP192 CEP250 CERS6 CHD6 CHMP4C CHRNA9 CHRNB2 CLSTN1 CNTFR CNTN5 CNTNAP1 CNTNAP2 COL11A1 COL14A1 COL15A1 COL17A1 COL1A1 COL1A2 COL21A1 COL22A1 COL24A1 COL25A1 COL27A1 COL28A1 COL4A2 COL5A1 COL5A2 COL5A3 COL6A5 COL6A6 COL7A1 COL8A1 CPAMD8 CPN2 CPXM2

CUL9 CYP51A1 DCHS1 DDR1 DNAH7 DNMT3A DNMT3B DSP E2F1 EGFR EPHA3 EPHB3 EPHB4 EZH2 F3 FAM208A FANCL FASN FAT1 FAT4 FCGBP FGFR1 FGFR2 GADD45G GATA3 GFI1B HERC1 HEYL HIC1 HMOX1 HSPG2 IFI27 IFI44L IGFBP4 IL21R ILKAP ITGA6 JARID2 KCNJ12 LGALS1 LINGO1 LRBA LRP4 MAGI2 MCM5 MCM6 MPO MYOD1 NALCN NCAM1
CPN2 CPXM2 CSF1R CSMD3 CTSB CUL9 CYP26B1 CYP51A1 DCHS1 DENND4B DKK3 DMXL2 DNMT3A DNMT3B DPP6 DPT E2F1 EGFR ELK1 EPHA3 EPHB3 EPHB4 EPHB6 EZH2 FASN FBRSL1 FCGBP FER1L6 FGFR1 FGFR2 FKBP10 FNDC1 FOSL1 GATA3 GLI2 GLI3 GNAL HERC1 HMCN1 HMOX1 HP HSPG2 HYI IGSF9B ITGA6 ITGA8 JAG2 KCNJ12 KIAA1109 KIF26A
E2F1 EGFR EPHA3 EPHB3 EPHB4 EZH2 F3 FAM208A FANCL FAT1 FAT4 FCGBP FGFR1 FGFR2 GADD45G GATA3 GFI1B HERC1 HEYL HIC1 HSPG2 IFI27 IFI44L IGFBP4 IL21R ILKAP ITGA6 JARID2 KCNJ12 LGALS1 LINGO1 LRBA LRP4 MAGI2 MCM5 MCM6 MPO MYOD1 NALCN NCAM1 NCEH1 NOSTRIN NSD2 ODC1 PBRM1 PDZD2 PER3 PLCG2 PLEKHG3 POLA1
CUL9 CYP51A1 DCHS1 DDR1 DNAH7 DNMT3A DNMT3B DSP E2F1 EGFR EPHA3 EPHB3 EPHB4 EZH2 F3 FAM208A FANCL FASN FAT1 FAT4 FCGBP FGFR1 FGFR2 GADD45G GATA3 GFI1B HERC1 HEYL HIC1 HMOX1 HSPG2 IFI27 IFI44L IGFBP4 IL21R ILKAP ITGA6 JARID2 KCNJ12 LGALS1 LINGO1 LRBA LRP4 MAGI2 MCM5 MCM6 MPO MYOD1 NALCN NCAM1

E2F1 EGFR EPHA3 EPHB3 EPHB4 EZH2 F3 FAM208A FANCL FAT1 FAT4 FCGBP FGFR1 FGFR2 GADD45G GATA3 GFI1B HERC1 HEYL HIC1 HSPG2 IFI27 IFI44L IGFBP4 IL21R ILKAP ITGA6 JARID2 KCNJ12 LGALS1 LINGO1 LRBA LRP4 LZTS2 MAGI2 MCM5 MCM6 MPO MYOD1 NALCN NCAM1 NCEH1 NOSTRIN NSD2 ODC1 PBRM1 PDE3A PDZD2 PER3 PLCG2

FANCA FANCL FASN FBXO32 FGFR1 FGFR2 GADD45G GFI1B GLI3 HIC1 HMOX1 HSPB8 IDO1 IGFBP4 ILKAP ITPR3 LGALS1 LIG1 LRP1 LTB4R2 LZTS2 MCM10 MEFV MIA MMP14 MMS22L MPHOSPH MSTN MYO6 NACC2 NCAM1 NME1 NSD2 ODC1 OSGIN1 PER1 PKMYT1 PLCG2 PRKCA PRKDC PTPRF PTPRR SEMA3F 4-Sep SLC3A2 SMOX SOCS1 SPIN1 SREBF1 SVIL

HIC1 HMOX1 HSPB8 IDO1 IGFBP4 ILKAP ITPR3 LGALS1 LIG1 LRP1 LTB4R2 MCM10 MIA MMP14 MSTN MYO6 NACC2 NCAM1 NME1 NSD2 ODC1 OSGIN1 PER1 PKMYT1 PLCG2 PRKCA PRKDC SEMA3F 4-Sep SMOX SOCS1 SREBF1 TLR2 TP53BP1 TSG101 WT1 XBP1

MYOD1 NALCN ORC1 PHC1 PIEZO2 PLXNB2 POLE PTCH1 PTPRF PTPRS RGMA SATB2 SKI SMS TCAP TNNI2 TNXB TPM4 WDR62 ZSWIM6

TET1 TP53BP1 XBP1
PLCG2 POU2F2 PTPRS RBL1 RFX3 SALL3 SATB2 SFRP5 SIK3 SKI SLC23A1 ST14 SV2A SYNJ1 UNC13A UNC79 WT1
CUL7 CUL9 CYP24A1 CYP26B1 CYP51A1 DAG1 DCHS1 DDR1 DNAJB9 DNMT3A DNMT3B DOT1L DSP E2F1 ECE1 EGFR EGLN3 EHHADH EIF2AK4 EPHA3 EPHB3 EPX EVC2 EXT1 EZH2 F3 FANCL FASN FAT1 FAT4 FGFR1 FGFR2 FLCN FOSL1 GATA3 GFI1B GLI2 GNAL HEYL HIC1 HMOX1 HP HPR HSD17B1 HSPB8 HSPG2 IDO1 IFT172 INSIG1 ITGA6

RELN RFX3 RHOU SALL3 SDK2 SEMA3E SEMA3F SEMA5A 4-Sep SHROOM4 SLC3A2 SLC9A5 SLIT1 SLIT3 SLITRK3 SRGAP1 SRGAP2 SRGAP3 SV2A TEKT3 TENM1 TLR7 TNC TNIK UNC119B WDR62 WISP2 WNT7B ZSWIM6

PTX3 PVR RAPH1 RELN RFX3 RHOU SALL3 SDK2 SEC61A1 SEMA3E SEMA3F SEMA5A 4-Sep SHROOM4 SLC3A2 SLC9A5 SLIT1 SLIT3 SLITRK3 SRGAP1 SRGAP2 SRGAP3 SV2A TEKT3 TENM1 TLR7 TNC TNIK UNC119B WDR62 WISP2 WNT7B ZSWIM6

SLC9A5 SLIT1 SLIT3 SLITRK3 SRGAP1 SRGAP2 SRGAP3 SV2A TEKT3 TENM1 TLR7 TNC TNIK UNC119B WISP2 WNT7B ZSWIM6

MBNL1 MINK1 MMP14 MMP19 MNX1 MSTN MTF2 MYO6 MYOD1 NCAM1 NCOA1 NME1 NME2 NMRK2 OCA2 ODC1 OGN PER3 PHC1 PIK3R2 PLCB1 PLCG2 PLXNB2 POU2F2 PPM1L PRKCA PRKDC PTCH1 PTPRF RAPH1 RBL1 RELN RLTPR RNH1 RORB S1PR4 SALL3 SATB2 SCUBE3 SDK2 SEMA3E SEMA3F SEMA4C SEMA5A SH3PXD2A SHC4 SIGLEC10 SIGLEC8 SIK3 SKI

MBNL1 MINK1 MMP14 MMP19 MNX1 MSTN MTF2 MYO6 MYOD1 NCAM1 NCOA1 NME1 NME2 NMRK2 OCA2 ODC1 OGN PER3 PHC1 PIK3R2 PLCB1 PLCG2 PLXNB2 POU2F2 PPM1L PRKCA PRKDC PTCH1 PTPRF RAPH1 RBL1 RELN RLTPR RNH1 RORB S1PR4 SALL3 SATB2 SCUBE3 SDK2 SEMA3E SEMA3F SEMA4C SEMA5A SH3PXD2A SHC4 SIGLEC10 SIGLEC8 SIK3 SKI

NEO1 NME1 NME2 NSD2 OSGIN1 PAK6 PER1 PIK3R2 PLA2G10 PLCG2 PLXNA2 PLXNB1 PLXNB2 PRKCA PTP4A2 PTPRF PTPRR PTX3 PVR RELN RHO RHOU RNH1 S1PR4 SATB2 SCN2B SEMA3E SEMA3F SEMA4C SEMA5A 4-Sep SFRP5 SH3PXD2A SHC4 SIGLEC8 SIGLEC9 SKI SLC1A3 SLC3A2 SLIT1 SLIT3 SOCS1 SPAG9 SREBF1 SRGAP1 STC1 SUZ12 TAX1BP1 TEKT3 TLR2

PLA2G10 PLCG2 PLXNA2 PLXNB1 PLXNB2 PRKCA PTP4A2 PTPRF PTX3 PVR RELN RHO RHOU RNH1 S1PR4 SATB2 SEMA3E SEMA3F SEMA4C SEMA5A 4-Sep SFRP5 SHC4 SIGLEC8 SIGLEC9 SLC1A3 SLC3A2 SLIT1 SLIT3 SOCS1 SPAG9 SREBF1 SRGAP1 STC1 SUZ12 TAX1BP1 TLR2 TLR7 TNC WISP2 WWC1 ZFAND5

SIK3 SKI SLC17A8 SLC1A3 SLC9A4 SOCS1 SOX13 SREBF1 ST14 TET1 TLR2 TNC TP53BP1 TRPM5 UNC13A UPF1 WNT7B WT1 XBP1 YBX2 ZNF521

GADD45G GATA3 GFI1B GLI2 GLI3 GNA15 GRB7 HEYL HIC1 HMOX1 HSPB8 HSPG2 IDO1 IFT172 IGFBP4 IL21R ILKAP INSIG1 IRS1 ITGA1 ITGA6 ITPR3 JAG2 JARID2 JDP2 KIF26A KLF11 LAMA1 LAMB1 LAMC1 LGALS1 LIG1 LINGO1 LPIN1 LRP1 LTB4R LTBP4 LZTS2 MAGI2 MCM10 MCM2 MCM3 MCM4 MCM5 MIA MLLT6 MMP14 MMP19 MNX1 MSTN

THRAP3 TLR2 TNNI2 TP53BP1 TRRAP TSG101 WT1 WWC1 XBP1 YBX2 ZFPM2 ZMIZ1 ZNF536

WT1 XBP1 ZMIZ1

PRKCA PRKDC PTCH1 RAD54L2 RBL1 RFX3 RORB SATB2 SCAF8 SEC61A1 SKI SMARCC2 SOCS1 SOX18 SPIN1 SREBF1 SUZ12 TCF7 TENM1 TET1 THRAP3 TLR2 TNC TNNI2 TP53BP1 TRRAP TSG101 WNT7B WT1 WWC1 XBP1 YBX2 ZFPM2 ZMIZ1 ZNF536

PRKCA PRKDC PTCH1 RAD54L2 RBL1 RFX3 RORB SATB2 SCAF8 SEC61A1 SKI SMARCC2 SOCS1 SOX18 SPIN1 SREBF1 SUZ12 TCF7 TENM1 TET1 THRAP3 TLR2 TNC TNNI2 TP53BP1 TRRAP TSG101 WNT7B WT1 WWC1 XBP1 YBX2 ZFPM2 ZMIZ1 ZNF536

EPX ERAP2 ERMP1 EZH2 F3 FAM208A FAT1 FAT3 FAT4 FCGBP FER1L6 FGFR1 FGFR2 FHOD3 FIGNL1 FKBP10 FNDC1 FNDC7 FRMD4A GATA3 GLI3 GNAL GPR155 GRB7 HIC1 HMCN1 HP HPR IDO1 IFI27 IFI44 IFI44L IGFBP4 IGSF10 IGSF9B IL21R ILKAP ITGA6 ITPR3 JAG2 JARID2 KCNJ12 KIAA0556 KIAA1109 LAMB1 LAMB4 LAMC1 LIG1 LIMCH1 LPO

CSF1R CSMD3 CTSE CTSL CUL9 CYP24A1 DAG1 DENND1B DEPDC5 DHX34 DKK3 DLEC1 DMXL2 DNAH7 DNAJC16 DNMT3B DOCK4 DOCK6 DOT1L DSCAML1 DSP E2F1 ECE1 EDA EFHB EGFR EHHADH EPHA3 EPHB3 EPHB6 EPX ESYT2 EVC2 EZH2 F3 FASN FAT3 FAT4 FCGBP FER1L6 FGFR1 FGFR2 FHOD3 FKBP10 FNDC1 FNDC7 GARNL3 GATA3 GATAD2B GLB1L2

LIG1 LINGO1 LRP1 LTB4R2 LZTS2 MCM10 MCM2 MEFV MIA MMP14 MMS22L MPHOSPH MPO MSTN MYO6 MYOD1 NACC2 NCAM1 NCEH1 NCOA1 NECTIN2 NEO1 NME1 NSD2 ODC1 OSGIN1 PAK6 PER1 PIK3R2 PKMYT1 PLCB1 PLCG2 PLXNB1 PPP1R9B PRKCA PRKDC PTCH1 PTPRF PTPRR RBL1 S1PR4 SEMA3F 4-Sep SH3PXD2A SIGLEC5 SIGLEC8 SIGLEC9 SKI SLC1A3 SLC25A38

DLEC1 DMXL2 DNAH7 DNMT3A DOCK10 DOCK4 DOT1L DPP6 DPT DSP E2F1 EFHB EGFR EPHA3 EPHB6 EPX ERAP2 ERMP1 EZH2 F3 FAM208A FASN FAT1 FAT3 FAT4 FCGBP FER1L6 FGFR1 FGFR2 FHOD3 FIGNL1 FKBP10 FNDC1 FNDC7 FRMD4A GATA3 GLB1L2 GLI3 GNAL GPR155 GRB7 HIC1 HMCN1 HMOX1 HP HPR IDO1 IFI27 IFI44 IFI44L

DHX34 DIP2B DLEC1 DMXL2 DNAH7 DNMT3A DOCK10 DOCK4 DOT1L DPP6 DPT DSP E2F1 EFHB EGFR EPHA3 EPHB6 EPX ERAP2 ERMP1 EZH2 F3 FAM208A FASN FAT1 FAT3 FAT4 FCGBP FER1L6 FGFR1 FGFR2 FHOD3 FIGNL1 FKBP10 FNDC1 FNDC7 FRMD4A GATA3 GLB1L2 GLI3 GNAL GPR155 GRB7 HIC1 HMCN1 HMOX1 HP HPR IDO1 IFI27

FIGNL1 FLCN FNDC1 FOSL1 FSTL3 GADD45G GATA3 GFI1B GLI2 GLI3 GNAL GRB7 HEYL HIC1 HMOX1 HP HSPB8 HSPG2 IDO1 IFI27 IGFBP4 IL21R ILKAP IRS1 ITGA1 ITGA6 ITPR3 JAG2 KLF11 LAMA1 LDLR LGALS1 LIG1 LINGO1 LRP1 LTB4R2 LZTS2 MCM10 MCM2 MEFV MIA MMP14 MMS22L MNX1 MPHOSPH MPO MSTN MYO6 MYOD1 NACC2

ERMP1 EZH2 F3 FAM208A FAT1 FAT3 FAT4 FCGBP FER1L6 FGFR1 FGFR2 FHOD3 FIGNL1 FKBP10 FNDC1 FNDC7 FRMD4A GATA3 GLI3 GNAL GPR155 GRB7 HIC1 HMCN1 HP HPR IDO1 IFI27 IFI44 IGFBP4 IGSF10 IGSF9B IL21R ILKAP ITGA6 ITPR3 JAG2 JARID2 KCNJ12 KIAA0556 KIAA1109 LAMB1 LAMB4 LAMC1 LIG1 LIMCH1 LPO LRBA LRGUK LRP1

CYP24A1 CYP51A1 DAB2IP DCHS1 DDIT4 DDR1 DENND4B DEPDC5 DHRS1 DHX34 DIP2B DLEC1 DMXL2 DNAH7 DNMT3A DOCK10 DOCK4 DOT1L DPP6 DPT DSP E2F1 EFHB EGFR EPHA3 EPHB6 EPX ERAP2 ERMP1 EZH2 F3 FAM208A FASN FAT1 FAT3 FAT4 FCGBP FER1L6 FGFR1 FGFR2 FHOD3 FIGNL1 FKBP10 FNDC1 FNDC7 FOSL1 FRMD4A GARNL3 GATA3 GFI1B

GNAL HEYL HIC1 HMOX1 HSPB8 HSPG2 IDO1 IFI27 IGFBP4 ILKAP IRS1 ITGA1 ITGA6 ITPR3 JAG2 KLF11 LAMA1 LDLR LGALS1 LIG1 LINGO1 LRP1 LTB4R2 LZTS2 MCM10 MCM2 MEFV MIA MMP14 MNX1 MPO MSTN MYO6 MYOD1 NACC2 NCAM1 NCEH1 NCOA1 NECTIN2 NME1 NME2 NRG2 NSD2 ODC1 OSGIN1 PAK6 PDE3A PER1 PIK3R2 PKMYT1

TANC2 TNC TSPEAR WNK4 XBP1 ZFPM2 ZNF521

RFPL4A/RF RFX8 RHBG SALL3 SCN8A SIGLEC1 SIGLEC10 SIGLEC5 SLC43A2 SLIT1 SLIT3 SOCS1 SPIN2A/SP SRGAP1 SRGAP2 SVEP1 SYDE2 TCAP TMEM235 TNXB TONSL TRPM5 TSG101 UNC13A WBP1L YBX2

CSF1R CSMD3 CTSE CTSL CUL9 CYP24A1 DAG1 DENND1B DEPDC5 DHX34 DKK3 DLEC1 DMXL2 DNAH7 DNAJC16 DNMT3B DOCK4 DOCK6 DOT1L DSCAML1 DSP E2F1 ECE1 EDA EFHB EGFR EHHADH EPHA3 EPHB3 EPHB6 EPX ESYT2 EVC2 EZH2 F3 FASN FAT3 FAT4 FCGBP FER1L6 FGFR1 FGFR2 FHOD3 FKBP10 FNDC1 FNDC7 GARNL3 GATA3 GATAD2B GLB1L2

NCEH1 NME1 NOSTRIN NSD2 ODC1 PBRM1 PDE3A PDZD2 PER3 PLCG2 PLEKHG3 POLA1 POLE POU2F2 PPP6R2 PRKCA PRKDC PTPRD PTPRF RELN RFPL4A/RF RHOU RIF1 RORB SALL3 SEMA5A SIGLEC1 SIGLEC10 SIGLEC7 SIGLEC9 SIPA1L2 SKI SLC24A3 SLC9A5 SLIT3 SOCS1 SPAG9 SUZ12 SVEP1 TAX1BP1 TCF7 TET1 THRAP3 TLR2 TLR7 TNIK TP53BP1 VWA7 WT1 XBP1

LAMC1 LDLR LGALS1 LRBA LTBP4 LZTS2 MAN1C1 MIA MMP14 MTSS1 NCAM1 NCOA1 NLRC5 NME1 NTN5 OTOF OTUD4 PARD3B PBRM1 PCNX1 PEAR1 PER1 PER3 PHC1 PLCB1 PLXNA2 PLXNB2 POLA1 POLE PRKCA PRKDC PRSS36 PTCH1 PTK7 PTPRD PTPRF PTPRS RAB6B RAPH1 RBL1 RELN RFPL1/RFF RFPL4A/RF RFX8 RHBG SALL3 SCN8A SIGLEC1 SIGLEC10 SIGLEC12

POLE POU2F2 PPP6R2 PRKDC PTPRD RELN RFPL4A/RF RHOU RIF1 RORB SALL3 SEMA5A SIGLEC10 SIGLEC9 SIPA1L2 SKI SLC24A3 SLC9A5 SLIT3 SOCS1 SPAG9 SUZ12 SVEP1 TAX1BP1 TCF7 TET1 THRAP3 TLR2 TLR7 TP53BP1 VWA7 WT1 XBP1 ZFPM2 ZMIZ1

NCEH1 NME1 NOSTRIN NSD2 ODC1 PBRM1 PDE3A PDZD2 PER3 PLCG2 PLEKHG3 POLA1 POLE POU2F2 PPP6R2 PRKCA PRKDC PTPRD PTPRF RELN RFPL4A/RF RHOU RIF1 RORB SALL3 SEMA5A SIGLEC1 SIGLEC10 SIGLEC9 SIPA1L2 SKI SLC24A3 SLC9A5 SLIT3 SOCS1 SPAG9 SUZ12 SVEP1 TAX1BP1 TCF7 TET1 THRAP3 TLR2 TLR7 TNIK TP53BP1 VWA7 WT1 XBP1 ZFPM2

PLEKHG3 POLA1 POLE POU2F2 PPP6R2 PRKDC PTPRD RELN RFPL4A/RF RHOU RIF1 RORB SALL3 SEMA5A SIGLEC10 SIGLEC9 SIPA1L2 SKI SLC24A3 SLC9A5 SLIT3 SOCS1 SPAG9 SUZ12 SVEP1 TAX1BP1 TCF7 TET1 THRAP3 TLR2 TLR7 TP53BP1 VWA7 WT1 XBP1 ZFPM2 ZMIZ1

TLR2 TP53BP1 TSG101 TYR WT1 XBP1

ITGA8 ITPR3 JAG2 KIF26A KIF5B LAMA1 LAMC1 LDLR LIAS LIG1 LRP1 LRP4 MAGI2 MBNL1 MBTD1 MCM10 MCM2 MED13 MMP14 MNX1 MPO MSTN MTF2 MTSS1 MYOD1 NALCN NCAM1 NCOA1 NRG2 PHC1 PHEX PHF21A PLA2G10 PLCB1 PLCG2 PLXNB2 POLG POU2F2 PRKCA PRKDC PTCH1 PTPRF PTPRS PTX3 RBL1 RFX3 RGMA RIF1 RPL24 SALL3

SLC1A3 SLC25A38 SLC3A2 SLIT1 SNX19 SOCS1 SPAG9 SPON1 SREBF1 SRGAP1 SRGAP2 ST14 STC1 SUZ12 SV2A TDO2 TET1 THRAP3 TLR2 TLR7 TMBIM1 TMEM120A TNC TPM4 TSG101 WDR7 WISP2 WNT7B WT1 XBP1 ZFPM2 ZNF521 ZNF536

SLC1A3 SLC25A38 SLC3A2 SLIT1 SNX19 SOCS1 SPAG9 SPON1 SREBF1 SRGAP1 SRGAP2 ST14 STC1 SUZ12 SV2A TDO2 TET1 THRAP3 TLR2 TLR7 TMBIM1 TMEM120A TNC TPM4 TSG101 WDR7 WISP2 WNT7B WT1 XBP1 ZFPM2 ZNF521 ZNF536

TLR7 TNC TTLL5 WISP2 WNK1 WT1 WWC1 ZFAND5

MTF2 MTSS1 MYOD1 NACC2 NCAM1 NCOA1 NEO1 NME1 NME2 NRG2 NSD2 OCA2 ODC1 OGN ORC1 OSGIN1 PAK6 PBRM1 PDE3A PER1 PHC1 PIK3R2 PLA2G10 PLCD4 PLCG2 PLXNB1 PLXNB2 POLA1 POLG POU2F2 PPP1R9B PRKCA PRKDC PTCH1 PTP4A2 PTPRF PTPRR PTPRS PTX3 RAPH1 RASGRF2 RBL1 RELN RHOU RLTPR RNH1 S1PR4 SCUBE3 SEC61A1 SEMA3F

LRBA LRGUK LRP1 LTBP4 MAGI2 MAST1 MBNL1 MCM2 MMP14 MPO MTF2 NALCN NCAM1 NCKAP5L NCOA1 NEO1 NME1 OCA2 OLFM3 OLFML2A OTC OTUD4 PAQR7 PBRM1 PCNX1 PDE1C PEAR1 PER3 PHC1 PHEX PIEZO2 PIK3R2 PIP5K1B PLCB1 PLCG2 PLXNA2 POLA1 POLE PPM1L PRKCA PRKDC PTCH1 PTPDC1 PTPRD PTPRF PTPRR RAD54L2 RASGRF2 RBL1 RELN

GLB1L3 GLI2 GLI3 GNA15 GYS2 HERC1 HIC1 HMCN1 HPCAL1 HPR HSPG2 IDO1 IFI27 IGFBP4 IGSF10 IGSF9B IL21R INSIG1 IQSEC2 IRS1 ITGA1 ITGA8 ITPR3 JAG2 KCNJ12 KIAA0556 KIAA1109 KLF11 KLHL30 LAMA1 LAMB1 LAMB4 LIG1 LIMCH1 LPIN1 LPO LRBA LRIT1 LRMP LRP1 LZTS2 MAGI2 MARK4 MASP2 MCM10 MCM2 ME1 MEFV MINK1 MLLT6

SLC3A2 SLC9A4 SLIT1 SLIT3 SMOX SOCS1 SPIN1 SREBF1 ST14 STC1 SVIL TAX1BP1 TCF7 TLR2 TLR7 TNC TP53BP1 TSG101 TTR TYR WNK3 WT1 XBP1

IGFBP4 IGSF10 IGSF9B IL21R ILKAP ITGA6 ITPR3 JAG2 JARID2 KCNJ12 KIAA0556 KIAA1109 LAMB1 LAMB4 LAMC1 LGALS1 LIG1 LIMCH1 LPO LRBA LRGUK LRP1 LRP4 LTB4R2 LTBP4 LZTS2 MAGI2 MAST1 MBNL1 MCM2 MCM5 MMP14 MPO MTF2 MXRA5 NALCN NCAM1 NCKAP5L NCOA1 NEO1 NME1 OCA2 ODC1 OLFM3 OLFML2A OTC OTUD4 PAK6 PAQR7 PBRM1

IFI44 IFI44L IGFBP4 IGSF10 IGSF9B IL21R ILKAP ITGA6 ITPR3 JAG2 JARID2 KCNJ12 KIAA0556 KIAA1109 LAMB1 LAMB4 LAMC1 LGALS1 LIG1 LIMCH1 LPO LRBA LRGUK LRP1 LRP4 LTB4R2 LTBP4 LZTS2 MAGI2 MAST1 MBNL1 MCM2 MCM5 MMP14 MPO MTF2 MXRA5 NALCN NCAM1 NCKAP5L NCOA1 NEO1 NME1 OCA2 ODC1 OLFM3 OLFML2A OTC OTUD4 PAK6

NCAM1 NCEH1 NCOA1 NECTIN2 NEO1 NME1 NME2 NRG2 NSD2 ODC1 OSGIN1 PAK6 PDE3A PER1 PIK3R2 PKMYT1 PLA2G10 PLCB1 PLCG2 PLXNB1 PPP1R9B PRKCA PRKDC PTCH1 PTP4A2 PTPRF PTPRR PVR RBL1 RHO S1PR4 SEMA3F 4-Sep SFRP5 SH3PXD2A SHC4 SIGLEC5 SIGLEC7 SIGLEC8 SIGLEC9 SKI SLC1A3 SLC25A38 SLC3A2 SLC9A4 SLIT1 SLIT3 SMOX SOCS1 SPIN1

LTBP4 MAGI2 MAST1 MBNL1 MCM2 MMP14 MPO MTF2 NALCN NCAM1 NCKAP5L NEO1 OCA2 OLFM3 OLFML2A OTC OTUD4 PAQR7 PBRM1 PCNX1 PDE1C PEAR1 PER3 PHC1 PHEX PIEZO2 PIK3R2 PIP5K1B PLCB1 PLCG2 PLXNA2 POLA1 POLE PPM1L PRKCA PRKDC PTCH1 PTPDC1 PTPRD PTPRF PTPRR RAD54L2 RASGRF2 RBL1 RELN RFPL1/RFF RHCG RIF1 SALL3 SATB2

GLB1L2 GLI3 GNAL GPR155 GRB7 HIC1 HMCN1 HMOX1 HP HPR HSD17B1 HSPG2 IDO1 IFI27 IFI44 IFI44L IGFBP4 IGSF10 IGSF9B IL21R ILKAP ITGA6 ITPR3 ITSN2 JAG2 JARID2 KCNJ12 KIAA0556 KIAA1109 KIF26A KLHL30 LAMB1 LAMB4 LAMC1 LGALS1 LIG1 LIMCH1 LPIN1 LPO LRBA LRGUK LRIT1 LRP1 LRP4 LTB4R LTBP4 LZTS2 MAGI2 MAST1 MBNL1

PLCG2 PPP1R9B PRKCA PRKDC PTCH1 PTP4A2 PTPRF RBL1 RHO S1PR4 SEMA3F 4-Sep SFRP5 SHC4 SIGLEC5 SIGLEC8 SIGLEC9 SKI SLC1A3 SLC25A38 SLC9A4 SMOX SOCS1 SREBF1 ST14 STC1 SUZ12 TAX1BP1 TLR2 TLR7 TNC TP53BP1 TSG101 TTR UPF1 WNK3 WT1 XBP1 YBX2 ZFAND5

GLB1L3 GLI2 GLI3 GNA15 GYS2 HERC1 HIC1 HMCN1 HPCAL1 HPR HSPG2 IDO1 IFI27 IGFBP4 IGSF10 IGSF9B IL21R INSIG1 IQSEC2 IRS1 ITGA1 ITGA6 ITGA8 ITPR3 JAG2 KCNJ12 KIAA0556 KIAA1109 KLF11 KLHL30 LAMA1 LAMB1 LAMB4 LDLR LIG1 LIMCH1 LPIN1 LPO LRBA LRIT1 LRMP LRP1 LZTS2 MAGI2 MARK4 MASP2 MCM10 MCM2 ME1 MEFV

ZFPM2 ZMIZ1

SIGLEC5 SLC17A9 SLC43A2 SLC9A4 SLIT1 SLIT3 SOCS1 SPIN2A/SP SPON1 SRGAP1 SRGAP2 SVEP1 SYDE2 TCAP TLR2 TMEM235 TNC TNXB TONSL TP53BP1 TRPM5 TRRAP TSG101 UNC13A VPS13B WBP1L YBX2

ZMIZ1

SATB2 SEMA5A SFRP5 SGK223 SH3PXD2A SIK3 SKI SLC1A3 SLC23A1 SLC3A2 SMARCC2 SOCS1 SREBF1 SRGAP3 ST14 SUZ12 SV2A SYNJ1 TAX1BP1 TENM3 TLR2 TLR7 TNXB TP53BP1 TRRAP TSG101 UNC13A UNC79 UPF1 WNT7B WT1 XBP1 ZFPM2 ZMIZ1

SEMA4C SEMA5A 4-Sep SESN1 SFRP5 SGK223 SHC4 SIGLEC1 SIGLEC10 SIGLEC6 SIGLEC7 SKI SLC1A3 SLC3A2 SLC9A5 SLIT1 SLIT3 SMOX SOCS1 SOX13 SOX18 SREBF1 SRGAP1 SRGAP2 SRGAP3 ST14 STC1 SUZ12 TCF7 TDO2 TENM1 THRAP3 TLR2 TLR7 TNC TNIK TNXB TP53BP1 TRRAP TSG101 TTR TYR UPP1 WISP2 WNK1 WNK4 WNT7B WT1 WWC1 XBP1

RFPL1/RFF RHCG RIF1 RORB SALL3 SATB2 SCAF8 SCN8A SCUBE1 SCUBE3 SDK2 SEC61A1 SEMA3E SEMA4C SGIP1 SH3PXD2A SIGLEC1 SIGLEC11 SIGLEC12 SIPA1L2 SLC17A8 SLC1A3 SLC24A3 SLC7A2 SLC7A6 SLC9A4 SLF1 SLIT3 SLITRK3 SMOX ST14 STC1 SUZ12 SV2A SVEP1 SYNJ1 TANC2 TECPR1 TENM1 TLR7 TNC TNXB TP53BP1 TPM4 TRPM5 TRRAP TSG101 TTC28 TTR UGP2

MMP14 MMP19 MPO MSTN MXRA5 NALCN NCAM1 NCEH1 NCOA1 NEO1 NLGN4X NLGN4Y NLRC5 NRG2 NTN5 NUP210 OCA2 OCM ODC1 OLFM3 OSGIN1 OTOF PAPLN PARD3B PDE1C PDE3A PDZD2 PER3 PFKFB4 PHC1 PLCB1 PLCG2 PLEKHG3 PLXNB1 PLXNB2 POLE POLG PRKCA PRKDC PRSS36 PTCH1 PTK7 PTPRD PTPRF PTPRR PTPRS PTX3 RAD54L2 RASGRF2 RELN

PCNX1 PDE1C PEAR1 PER1 PER3 PHC1 PHEX PIEZO2 PIK3R2 PIP5K1B PLCB1 PLCG2 PLXNA2 PLXNB1 POLA1 POLE PPM1L PRKCA PRKDC PTCH1 PTK7 PTPDC1 PTPRD PTPRF PTPRR RAD54L2 RASGRF2 RBL1 RELN RFPL1/RFF RHCG RIF1 SALL3 SATB2 SCAF8 SCN8A SCUBE1 SCUBE3 SDK2 SEC61A1 SEMA3E SEMA4C SGIP1 SH3PXD2A SIGLEC1 SIGLEC11 SIGLEC12 SIPA1L2 SLC17A8 SLC1A3

PAQR7 PBRM1 PCNX1 PDE1C PEAR1 PER1 PER3 PHC1 PHEX PIEZO2 PIK3R2 PIP5K1B PLCB1 PLCG2 PLXNA2 PLXNB1 POLA1 POLE PPM1L PRKCA PRKDC PTCH1 PTK7 PTPDC1 PTPRD PTPRF PTPRR RAD54L2 RASGRF2 RBL1 RELN RFPL1/RFF RHCG RIF1 RORB SALL3 SATB2 SCAF8 SCN8A SCUBE1 SCUBE3 SDK2 SEC61A1 SEMA3E SEMA4C SGIP1 SH3PXD2A SIGLEC1 SIGLEC11 SIGLEC12

SREBF1 ST14 STC1 SUZ12 SVIL TAX1BP1 TCF7 TLR2 TLR7 TNC TP53BP1 TSG101 TTR TYR UPF1 WNK3 WT1 XBP1 YBX2 ZFAND5 ZFPM2

SCAF8 SCN8A SCUBE1 SCUBE3 SDK2 SEC61A1 SEMA3E SEMA4C SGIP1 SH3PXD2A SIGLEC1 SIGLEC11 SIGLEC12 SIPA1L2 SLC17A8 SLC1A3 SLC24A3 SLC7A2 SLC7A6 SLC9A4 SLF1 SLIT3 SLITRK3 SMOX ST14 STC1 SUZ12 SV2A SVEP1 SYNJ1 TANC2 TECPR1 TENM1 TLR7 TNC TNXB TP53BP1 TPM4 TRPM5 TRRAP TSG101 TTC28 TTR UGP2 VPS13B WDFY3 WDR7 WISP2 WNK3 WT1

MBTD1 MCM2 MCM5 MEFV MMP14 MPO MTF2 MXRA5 NACC2 NALCN NCAM1 NCKAP5L NCOA1 NEO1 NME1 OCA2 ODC1 OLFM3 OLFML2A OTC OTUD4 PAK6 PAQR7 PBRM1 PCNX1 PDE1C PDE3A PDZD2 PEAR1 PER1 PER3 PHC1 PHEX PIEZO2 PIK3R2 PIP5K1B PLCB1 PLCG2 PLXNA2 PLXNB1 POLA1 POLE PPM1L PRKCA PRKDC PRPSAP1 PRSS36 PTCH1 PTK7 PTPDC1

MINK1 MLLT6 MMP14 MMP19 MPO MSTN MXRA5 NALCN NCAM1 NCEH1 NCOA1 NEO1 NLGN4X NLGN4Y NLRC5 NRG2 NTN5 NUP210 OCA2 OCM ODC1 OLFM3 OSGIN1 OTOF PAPLN PARD3B PDE1C PDE3A PDZD2 PER3 PFKFB4 PHC1 PLCB1 PLCG2 PLEKHG3 PLXNB1 PLXNB2 POLE POLG PRKCA PRKDC PRSS36 PTCH1 PTK7 PTPRD PTPRF PTPRR PTPRS PTX3 RAD54L2

ZBED1 ZMIZ1 ZNF521

VPS13B WDFY3 WDR7 WISP2 WNK3 WT1 WWC1 ZBED1 ZFPM2 ZFYVE1 ZMIZ1

RFPL1/RFFRFPL4B RGMA RHBG RHO RHOU RIF1 RORB SAG SALL3 SATB2 SCN2B SCN8A SCUBE1 SCUBE3 SDK2 SEMA3E SEMA3F SEMA3G SEMA5A SGIP1 SH3PXD2ASHROOM4 SIGLEC1 SIGLEC10 SIGLEC11 SIGLEC12 SIGLEC14 SIGLEC6 SIGLEC7 SIGLEC8 SIGLEC9 SLC15A2 SLC16A9 SLC17A8 SLC17A9 SLC22A7 SLC24A3 SLC25A38 SLC43A2 SLC7A2 SLC9A2 SLC9A4 SLF1 SLIT3 SLITRK3 SNX19 SOX13 SPAG9 SPON1

SLC24A3 SLC7A2 SLC7A6 SLC9A4 SLF1 SLIT3 SLITRK3 SMOX SMS SOCS1 SRGAP1 ST14 STC1 SUZ12 SV2A SVEP1 SYNJ1 TANC2 TECPR1 TENM1 THRAP3 TLR2 TLR7 TNC TNXB TP53BP1 TPM4 TRPM5 TRRAP TSG101 TTC28 TTR UGP2 UPP1 VPS13B WDFY3 WDR62 WDR7 WISP2 WNK3 WT1 WWC1 ZBED1 ZFPM2 ZFYVE1 ZMIZ1
SIPA1L2 SLC17A8 SLC1A3 SLC24A3 SLC7A2 SLC7A6 SLC9A4 SLF1 SLIT3 SLITRK3 SMOX SMS SOCS1 SRGAP1 ST14 STC1 SUZ12 SV2A SVEP1 SYNJ1 TANC2 TECPR1 TENM1 THRAP3 TLR2 TLR7 TNC TNXB TP53BP1 TPM4 TRPM5 TRRAP TSG101 TTC28 TTR UGP2 UPP1 VPS13B WDFY3 WDR62 WDR7 WISP2 WNK3 WT1 WWC1 ZBED1 ZFPM2 ZFYVE1 ZMIZ1

WWC1 ZBED1 ZFPM2 ZFYVE1 ZMIZ1
PTPRD PTPRF PTPRR RAD54L2 RASGRF2 RBL1 RELN RFPL1/RFFRHBG RHCG RIF1 SALL3 SATB2 SCAF8 SCN8A SCUBE1 SCUBE3 SDK2 SEC61A1 SEMA3E SEMA4C SEMA5A SGIP1 SH3PXD2ASHROOM4 SIGLEC1 SIGLEC11 SIGLEC12 SIPA1L2 SLC17A8 SLC1A3 SLC24A3 SLC43A2 SLC7A2 SLC7A6 SLC9A4 SLC9A5 SLF1 SLIT3 SLITRK3 SMOX SMS SNX19 SOCS1 SRGAP1 ST14 STC1 SUZ12 SV2A SVEP1

RASGRF2 RBL1 RELN RFPL1/RFFRFPL4B RGMA RHBG RHO RHOU RIF1 RORB SAG SALL3 SATB2 SCN2B SCN8A SCUBE1 SCUBE3 SDK2 SEMA3E SEMA3F SEMA3G SEMA5A SGIP1 SH3PXD2ASHROOM4 SIGLEC1 SIGLEC10 SIGLEC11 SIGLEC12 SIGLEC14 SIGLEC6 SIGLEC7 SIGLEC8 SIGLEC9 SLC15A2 SLC16A9 SLC17A8 SLC17A9 SLC22A7 SLC24A3 SLC25A38 SLC43A2 SLC7A2 SLC9A2 SLC9A4 SLF1 SLIT3 SLITRK3 SNX19

SRGAP2 SRGAP3 ST14 STYK1 SV2A SVEP1 SYNJ1 TANC2 TEKT3 TENM1 TET1 THRAP3 THSD4 TLR2 TLR7 TNC TNXB TP53BP1 TRIML2 TRRAP TYR UNC13A UNC79 USH1G VPS13B VWA7 WDFY3 WDHD1 WDR62 WDR7 WIPF2 WIZ WNT7B WWC1 ZFPM2 ZNF362 ZNF536

SYNJ1 TANC2 TECPR1 TENM1 THRAP3 TLR2 TLR7 TNC TNXB TONSL TP53BP1 TPM4 TRPM5 TRRAP TSG101 TTC28 TTR UGP2 VPS13B WDFY3 WDR62 WDR7 WISP2 WNK1 WNK3 WT1 WWC1 ZBED1 ZFPM2 ZFYVE1 ZMIZ1

SOCS1 SOX13 SPAG9 SPON1 SRGAP2 SRGAP3 ST14 STYK1 SV2A SVEP1 SYNJ1 TANC2 TEKT3 TENM1 TET1 THRAP3 THSD4 TLR2 TLR7 TNC TNXB TP53BP1 TRIML2 TRRAP TYR UNC13A UNC79 USH1G VPS13B VWA7 WDFY3 WDHD1 WDR62 WDR7 WIPF2 WIZ WNT7B WWC1 ZFPM2 ZNF362 ZNF536
