# Supplementary material for: Waveband specific transcriptional control of select genetic pathways in vertebrate skin (Xiphophorus maculatus)
Source: BMC Genomics. 2018 May 10;19:355. doi: 10.1186/s12864-018-4735-5 (PMC5946439; doi:10.1186/s12864-018-4735-5)
Supplement: Supplementary file 2 — Table S2a–k. A list of all differentially modulated genes used by IPA enrichment software to predict the direction of change for each functional class represented in Additional file 1: Table S1. Table a is FL, tables b–e are the 50 nm wavebands and tables g–k are the 10 nm wavebands. (ZIP 701 kb) [file 12864_2018_4735_MOESM2_ESM.zip › TableS2g_500-510nm.pdf]

Functional Class	p-Value	Activation	# Genes	Genes										
failure of kidney	7.30E-03	-2.729	7	AGT	CYP1A1	HMGCR	HMOX1	MST1	PTHLH	WT1				
glomerulosclerosis	9.36E-03	-2.515	5	AGT	HMOX1	PTHLH	SREBF1	WT1						
concentration of triacylg	2.11E-04	-2.297	12	AGT	APC	COL18A1	FASN	GCK	INSIG1	MSTN	PTHLH	RRAD	SIK3	SREBF1
differentiation of cells	4.48E-05	-2.142	51	ADAMTS2(	AGRN	AGT	ALOX15B	ALOXE3	APC	ARHGAP32	COL18A1	CYB5D2	DDIT4	DOT1L
density of neurons	8.29E-03	-2.103	5	ARHGAP32	KALRN	PLXNB1	PTPRF	SLITRK3						
concentration of choles	1.76E-03	2.027	10	AGT	AQP12A/A(	DHCR24	GCK	HMGCR	HP	INSIG1	SIK3	SREBF1	TG	
lymphoid cancer	3.56E-04	2.085	39	ADGRL2	APC	ATR	CARMIL3	CELSR2	CEP192	COL18A1	COL7A1	CUL7	CUL9	CYP1A1
lymphohematopoietic ca	4.31E-05	2.085	45	ADGRL2	ALOX12B	ANXA5	APC	ATR	ATRN	CARMIL3	CELSR2	CEP192	COL18A1	COL7A1
differentiation of connec	1.02E-03	2.089	9	COL11A1	CYP1A1	CYP1A2	DHCR24	GLI2	JDP2	MST1	PTHLH	WT1		
organismal death	2.87E-05	2.121	54	AGRN	AGT	ALOX12B	ALOXE3	APC	ATR	BIRC6	CAPN1	CERK	COL11A1	COL7A1
hepatocellular carcinom	4.69E-11	2.121	100	ABCC5	ABI3BP	ADAMTS1(	ADAMTS2(	ADAMTSL4	AGRN	AGT	AOX1	APC	AQP12A/A(	ARNTL2
migration of epithelial ce	7.79E-03	2.122	5	AGT	COL7A1	EPHB3	MST1	PTPRF						
neonatal death	7.82E-04	2.128	14	ALOX12B	ALOXE3	BIRC6	CUL7	CYP1A2	FAT4	GLI2	LRP6	MNX1	PHF21A	PTHLH
growth of embryonic tis	3.92E-03	2.172	9	AGT	COL18A1	GLI2	LRP6	NCOA1	PTHLH	TNC	TSC1	YY1AP1		
differentiation of connec	8.02E-04	2.173	20	AGT	ALOXE3	APC	EPHB4	FASN	FSTL3	GATA3	GLI2	HMOX1	INSIG1	ISG15
perinatal death	3.80E-04	2.175	18	AGRN	ALOX12B	ALOXE3	BIRC6	CUL7	CYP1A2	FAT4	GLI2	HMOX1	LRP6	MNX1
metabolism of terpenoic	1.56E-04	2.196	12	AGT	APC	CYP1A1	CYP1A2	CYP51A1	DHCR24	EBP	HMGCR	INSIG1	LSS	SQLE
liver cancer	5.57E-11	2.206	102	ABCC5	ABI3BP	ADAMTS1(	ADAMTS2(	ADAMTSL4	AGRN	AGT	AOX1	APC	AQP12A/A(	ARNTL2
hepatobiliary system ca	2.84E-11	2.206	103	ABCC5	ABI3BP	ADAMTS1(	ADAMTS2(	ADAMTSL4	AGRN	AGT	AOX1	APC	AQP12A/A(	ARNTL2
benign neoplasia	1.53E-03	2.227	23	AGRN	ANXA5	APC	ATR	ATRN	COL11A1	COL18A1	COL4A6	COL7A1	CUL9	EPHB3
tumorigenesis of tissue	7.96E-07	2.24	155	ABCC5	ABI3BP	ADAMTS1(	ADAMTS2(	ADAMTSL4	ADGRL2	AGRN	AGT	ALOX12B	ALOX15B	ANKRD1
digestive system cance	2.39E-08	2.241	140	ABCC5	ABI3BP	ADAMTS1(	ADAMTS2(	ADAMTSL4	ADGRL2	AGRN	AGT	ALOX15B	AOX1	APC
liver tumor	1.23E-11	2.316	104	ABCC5	ABI3BP	ADAMTS1(	ADAMTS2(	ADAMTSL4	AGRN	AGT	AOX1	APC	AQP12A/A(	ARNTL2
quantity of leptin	8.04E-03	2.342	5	MSTN	NCOA1	PER1	PER2	SIK3						
epithelial cancer	8.64E-07	2.415	152	ABCC5	ABI3BP	ADAMTS1(	ADAMTS2(	ADAMTSL4	ADGRL2	AGRN	AGT	ALOX12B	ALOX15B	ANKRD1
abdominal cancer	8.10E-07	2.496	151	ABCC5	ABI3BP	ADAMTS1(	ADAMTS2(	ADAMTSL4	ADGRL2	AGRN	AGT	ALOX15B	ANKRD1	ANXA5
steroid metabolism	1.31E-04	2.513	11	AGT	CYP1A1	CYP1A2	CYP51A1	DHCR24	EBP	HMGCR	INSIG1	LSS	SQLE	SREBF1
neoplasia of epithelial c	3.92E-07	2.662	154	ABCC5	ABI3BP	ADAMTS1(	ADAMTS2(	ADAMTSL4	ADGRL2	AGRN	AGT	ALOX12B	ALOX15B	ANKRD1
abdominal neoplasm	1.03E-06	2.67	152	ABCC5	ABI3BP	ADAMTS1(	ADAMTS2(	ADAMTSL4	ADGRL2	AGRN	AGT	ALOX15B	ANKRD1	ANXA5
ploidy	1.89E-03	2.71	7	AGT	APC	CUL9	DOT1L	LRP6	SREBF1	WNK1				
digestive organ tumor	3.74E-09	2.775	143	ABCC5	ABI3BP	ADAMTS1(	ADAMTS2(	ADAMTSL4	ADGRL2	AGRN	AGT	ALOX15B	AOX1	APC
biosynthesis of hydroge	3.30E-03	2.964	5	AGT	CYP1A1	CYP1A2	HBB	TG						
metabolism of eicosano	1.53E-03	2.968	9	AGT	ALOX12B	ALOX15B	CERK	CYP1A1	CYP1A2	FASN	HBB	HMOX1		
fatty acid oxidation	1.01E-04	3.01	9	ACSBG1	AGT	CERK	CYP1A1	CYP1A2	EXTL1	GCK	HMOX1	SREBF1		
lipid oxidation	3.89E-04	3.27	17	AGT	ALOX12B	ALOXE3	APC	CERK	CYP1A1	CYP51A1	DHCR24	EBP	FASN	HBB

TSC1															
EBP	EPHB3	EPHB4	EXTL1	FASN	FSTL3	GATA3	GLI2	GON4L	HMOX1	HSPA5	INSIG1	ISG15	JARID2	JDP2	KALRN
CYP51A1	EPHB3	EPHB4	FAT4	GATA3	HEATR1	HERC1	HIST1H3B	HMGCR	HSPA5	JARID2	KMT2C	LAMB3	LRBA	LRP6	MST1
CUL7	CUL9	CYP1A1	CYP51A1	EPHB3	EPHB4	FASN	FAT4	GATA3	HEATR1	HERC1	HIST1H3B	HMGCR	HMOX1	HSPA5	JARID2
CUL7	CUL9	CYP1A1	CYP1A2	CYP51A1	DOT1L	EPHB3	FASN	FAT4	GATA3	GCK	GLI2	HMGCR	HMOX1	HP	HPR
ATAD2	ATR	ATRN	BCAS3	BIRC6	CARMIL3	CELSR2	CEP192	CLASP1	COL11A1	COL18A1	COL4A6	COL7A1	CUL9	CYP51A1	DDIT4
SIK3	TSC1	WT1													
JDP2	LAMB3	LRP6	MSTN	PTHLH	SIK3	SREBF1	TNC	WT1							
MSTN	NUP98	PHF21A	PTHLH	SIK3	TSC1	WT1									
SREBF1															
ATAD2	ATR	ATRN	BCAS3	BIRC6	CARMIL3	CELSR2	CEP192	CLASP1	COL11A1	COL18A1	COL4A6	COL7A1	CUL9	CYP51A1	DDIT4
ATAD2	ATR	ATRN	BCAS3	BIRC6	CARMIL3	CELSR2	CEP192	CLASP1	COL11A1	COL18A1	COL4A6	COL7A1	CUL9	CYP1A1	CYP51A1
FAM57A	FKBP10	GATA3	GLI2	HMGCR	HMOX1	HP	KMT2C	LAMB3	TG	TNC	TSC1				
ANXA5	AOX1	APC	AQP12A/A(	ARHGAP32	ARNTL2	ATAD2	ATR	ATRN	BCAS3	BIRC6	BPIFC	C6	CAMTA2	CAPN1	CARMIL3
AQP12A/A(	ARHGAP32	ARNTL2	ATAD2	ATR	ATRN	BCAS3	BIRC6	C6	CAMTA2	CAPN1	CARMIL3	CASKIN1	CELSR2	CEP192	CLASP1
ATAD2	ATR	ATRN	BCAS3	BIRC6	CARMIL3	CELSR2	CEP192	CLASP1	COL11A1	COL18A1	COL4A6	COL7A1	CUL9	CYP51A1	DDIT4
ANXA5	AOX1	APC	AQP12A/A(	ARHGAP32	ARNTL2	ATAD2	ATR	ATRN	BCAS3	BIRC6	BPIFC	C6	CAMTA2	CAPN1	CARMIL3
AOX1	APC	AQP12A/A(	ARHGAP32	ARNTL2	ATAD2	ATR	ATRN	BCAS3	BIRC6	BPIFC	C6	CAMTA2	CAPN1	CARMIL3	CASKIN1
ANXA5	AOX1	APC	AQP12A/A(	ARHGAP32	ARNTL2	ATAD2	ATR	ATRN	BCAS3	BIRC6	BPIFC	C6	CAMTA2	CAPN1	CARMIL3
AOX1	APC	AQP12A/A(	ARHGAP32	ARNTL2	ATAD2	ATR	ATRN	BCAS3	BIRC6	BPIFC	C6	CAMTA2	CAPN1	CARMIL3	CASKIN1
AQP12A/A(	ARHGAP32	ARNTL2	ATAD2	ATR	ATRN	BCAS3	BIRC6	C6	CAMTA2	CAPN1	CARMIL3	CASKIN1	CELSR2	CEP192	CLASP1
HMGCR	HMOX1	INSIG1	LSS	MSTN	SREBF1										

LAMB3	LRP6	MNX1	MST1	MSTN	NCOA1	NMRK2	NUP98	PER3	PLXNA4	PTHLH	PTPRF	RRAD	SEMA5A	SHC4	SIK3
NUP98	PER2	PER3	PLXNA4	PRDM10	RFPL4A/RF	RNF123	SEMA5A	SUZ12	SVEP1	VPS13D	WT1				
KMT2C	LAMB3	LRBA	LRP6	MST1	NUP98	PER2	PER3	PLXNA4	PRDM10	PTPRF	RFPL4A/RF	RNF123	SEMA5A	SUZ12	SVEP1
HSPA5	INSIG1	ISG15	KMT2C	LIAS	LRP6	MCM3AP	MED13	MNX1	MSTN	NCOA1	NUP98	PER2	PHF21A	PIK3R4	PTHLH
DHCR24	DIP2B	DMXL2	DOT1L	EMC9	FASN	FAT4	FBRSL1	FKBP10	FRAS1	FREM2	FSTL3	GCK	GCN1	GLI2	GON4L
DHCR24	DIP2B	DMXL2	DOT1L	EMC9	FASN	FAT4	FBRSL1	FKBP10	FRAS1	FREM2	FSTL3	GCK	GCN1	GLI2	GON4L
DDIT4	DHCR24	DIP2B	DMXL2	DOT1L	EMC9	FASN	FAT4	FBRSL1	FKBP10	FRAS1	FREM2	FSTL3	GCK	GCN1	GLI2
CASKIN1	CELSR2	CEP192	CLASP1	COL11A1	COL18A1	COL4A6	COL7A1	CUL7	CUL9	CYP1A1	CYP1A2	CYP51A1	DDIT4	DENND4B	DHCR24
COL11A1	COL18A1	COL4A6	COL7A1	CUL7	CUL9	CYP1A1	CYP1A2	CYP51A1	DDIT4	DENND4B	DHCR24	DIP2B	DMXL2	DOT1L	EBP
DHCR24	DIP2B	DMXL2	DOT1L	EMC9	FASN	FAT4	FBRSL1	FKBP10	FRAS1	FREM2	FSTL3	GCK	GCN1	GLI2	GON4L
CASKIN1	CELSR2	CEP192	CLASP1	COL11A1	COL18A1	COL4A6	COL7A1	CUL7	CUL9	CYP1A1	CYP1A2	CYP51A1	DDIT4	DENND4B	DHCR24
CELSR2	CEP192	CLASP1	COL11A1	COL18A1	COL4A6	COL7A1	CUL7	CUL9	CYP1A1	CYP1A2	CYP51A1	DDIT4	DENND4B	DHCR24	DIP2B
CASKIN1	CELSR2	CEP192	CLASP1	COL11A1	COL18A1	COL4A6	COL7A1	CUL7	CUL9	CYP1A1	CYP1A2	CYP51A1	DDIT4	DENND4B	DHCR24
CELSR2	CEP192	CLASP1	COL11A1	COL18A1	COL4A6	COL7A1	CUL7	CUL9	CYP1A1	CYP1A2	CYP51A1	DDIT4	DENND4B	DHCR24	DIP2B
COL11A1	COL18A1	COL4A6	COL7A1	CUL7	CUL9	CYP1A1	CYP1A2	CYP51A1	DDIT4	DENND4B	DHCR24	DIP2B	DMXL2	DOT1L	EBP

SREBF1	SUZ12	TG	TMBIM1	TNC	TSC1	UBR2	WT1								
VPS13D	WT1														
PTPRF	RPL24	SEMA5A	SIK3	SLC4A1	SREBF1	SUZ12	TRRAP	TSC1	UBR2	WT1					
HBB	HEATR1	HERC1	HERC2	HIST1H3B	HIST1H3G	HMGCR	HMOX1	HP	HSPA5	INSIG1	JARID2	JDP2	KALRN	KIAA0556	KIAA1109
HBB	HEATR1	HERC1	HERC2	HIST1H3B	HIST1H3G	HMGCR	HMOX1	HP	HSPA5	INSIG1	ISG15	JARID2	JDP2	KALRN	KIAA0556
GON4L	HBB	HEATR1	HERC1	HERC2	HIST1H3B	HIST1H3G	HMGCR	HMOX1	HP	HSPA5	INSIG1	ISG15	JARID2	JDP2	KALRN
DIP2B	DMXL2	DOT1L	DQX1	EBP	EMC9	EPHB3	EPHB4	FAM134B	FAM57A	FASN	FAT4	FBRSL1	FKBP10	FRAS1	FREM2
EMC9	EPHB4	FAM134B	FASN	FAT4	FBRSL1	FKBP10	FRAS1	FREM2	FSTL3	GATA3	GCK	GCN1	GLI2	GON4L	HBB
HBB	HEATR1	HERC1	HERC2	HIST1H3B	HIST1H3G	HMGCR	HMOX1	HP	HSPA5	INSIG1	ISG15	JARID2	JDP2	KALRN	KIAA0556
DIP2B	DMXL2	DOT1L	DQX1	EBP	EMC9	EPHB4	FAM134B	FASN	FAT4	FBRSL1	FKBP10	FRAS1	FREM2	FSTL3	GATA3
DMXL2	DOT1L	DQX1	EBP	EMC9	EPHB4	FAM134B	FASN	FAT4	FBRSL1	FKBP10	FRAS1	FREM2	FSTL3	GATA3	GCK
DIP2B	DMXL2	DOT1L	DQX1	EBP	EMC9	EPHB3	EPHB4	FAM134B	FAM57A	FASN	FAT4	FBRSL1	FKBP10	FRAS1	FREM2
DMXL2	DOT1L	DQX1	EBP	EMC9	EPHB3	EPHB4	FAM134B	FASN	FAT4	FBRSL1	FKBP10	FRAS1	FREM2	FSTL3	GATA3
EMC9	EPHB3	EPHB4	FAM134B	FASN	FAT4	FBRSL1	FKBP10	FRAS1	FREM2	FSTL3	GATA3	GCK	GCN1	GLI2	GON4L

KMT2C	LAMB3	LRP6	MCM3AP	MED13	NAALADL2	NUP205	NUP98	PER1	PER3	PFAS	PIK3R4	PLEKHA5	PLXNA4	PLXNB1	PRR12
KIAA1109	KMT2C	LAMB3	LRP6	MCM3AP	MED13	NAALADL2	NCOA1	NUP205	NUP98	PER1	PER3	PFAS	PIK3R4	PLEKHA5	PLXNA4
KIAA0556	KIAA1109	KMT2C	LAMB3	LRP6	MCM3AP	MED13	NAALADL2	NCOA1	NUP205	NUP98	PER1	PER3	PFAS	PIK3R4	PLEKHA5
FSTL3	GATA3	GCK	GCN1	GLI2	GON4L	HBB	HBD	HBE1	HEATR1	HERC1	HERC2	HIST1H3B	HIST1H3G	HMGCR	HMOX1
HEATR1	HERC1	HERC2	HIST1H3B	HIST1H3G	HMGCR	HMOX1	HP	HSPA5	INSIG1	ISG15	JARID2	JDP2	KALRN	KIAA0556	KIAA1109
KIAA1109	KMT2C	LAMB3	LRP6	MCM3AP	MED13	MST1	NAALADL2	NCOA1	NUP205	NUP98	PER1	PER3	PFAS	PIK3R4	PLEKHA5
GCK	GCN1	GLI2	GON4L	HBB	HBD	HBE1	HEATR1	HERC1	HERC2	HIST1H3B	HIST1H3G	HMGCR	HMOX1	HP	HPR
GCN1	GLI2	GON4L	HBB	HBD	HBE1	HEATR1	HERC1	HERC2	HIST1H3B	HIST1H3G	HMGCR	HMOX1	HP	HPR	HSPA5
FSTL3	GATA3	GCK	GCN1	GLI2	GON4L	HBB	HBD	HBE1	HEATR1	HERC1	HERC2	HIST1H3B	HIST1H3G	HMGCR	HMOX1
GCK	GCN1	GLI2	GON4L	HBB	HBD	HBE1	HEATR1	HERC1	HERC2	HIST1H3B	HIST1H3G	HMGCR	HMOX1	HP	HPR
HBB	HEATR1	HERC1	HERC2	HIST1H3B	HIST1H3G	HMGCR	HMOX1	HP	HSPA5	INSIG1	ISG15	JARID2	JDP2	KALRN	KIAA0556

PTPRB	PTPRF	RFPL1/RFF	RRAD	SHC4	SLC4A1	SLITRK3	SQLE	SREBF1	SVEP1	TCF20	TECPR1	TG	TMEM63B	TONSL	TRIML2

PLXNB1	PRR12	PTPRB	PTPRF	RFPL1/RFF	RRAD	SHC4	SLC4A1	SLITRK3	SQLE	SREBF1	SVEP1	TCF20	TECPR1	TG	TMEM63B
PLXNA4	PLXNB1	PRR12	PTPRB	PTPRF	RFPL1/RFF	RRAD	SHC4	SLC4A1	SLITRK3	SQLE	SREBF1	SVEP1	TCF20	TECPR1	TG

HP	HPR	HSPA5	INSIG1	ISG15	JARID2	JDP2	KALRN	KIAA0556	KIAA1109	KLF11	KMT2C	LAMB3	LAMB4	LIAS	LRBA
KLF11	KMT2C	LAMB3	LAMB4	LIAS	LRBA	LRP6	MCM3AP	MED13	MEFV	MSTN	NAALADL2	NACC2	NCOA1	NR1D2	NUP205
PLXNA4	PLXNB1	PRR12	PTPRB	PTPRF	RFPL1/RFF	RRAD	SHC4	SLC4A1	SLITRK3	SQLE	SREBF1	SVEP1	TCF20	TECPR1	TG

HSPA5	INSIG1	ISG15	JARID2	JDP2	KALRN	KIAA0556	KIAA1109	KLF11	KMT2C	LAMB3	LAMB4	LIAS	LRBA	LRP6	MCM3AP
INSIG1	ISG15	JARID2	JDP2	KALRN	KIAA0556	KIAA1109	KLF11	KMT2C	LAMB3	LAMB4	LIAS	LRBA	LRP6	MCM3AP	MED13

HP	HPR	HSPA5	INSIG1	ISG15	JARID2	JDP2	KALRN	KIAA0556	KIAA1109	KLF11	KMT2C	LAMB3	LAMB4	LIAS	LRBA
HSPA5	INSIG1	ISG15	JARID2	JDP2	KALRN	KIAA0556	KIAA1109	KLF11	KMT2C	LAMB3	LAMB4	LIAS	LRBA	LRP6	MCM3AP

KIAA1109	KLF11	KMT2C	LAMB3	LAMB4	LIAS	LRBA	LRP6	MCM3AP	MED13	MEFV	MST1	MSTN	NAALADL2	NACC2	NCOA1

TRRAP	UBR2	VPS13B	VPS13C	VPS13D	WDFY3	WDR17	WNK1	WT1

TONSL	TRIML2	TRRAP	UBR2	VPS13B	VPS13C	VPS13D	WDFY3	WDR17	WNK1	WT1	
TMEM63B	TONSL	TRIML2	TRRAP	UBR2	VPS13B	VPS13C	VPS13D	WDFY3	WDR17	WNK1	WT1

LRP6	MCM3AP	MED13	MEFV	MST1	MSTN	NAALADL2	NACC2	NCOA1	NR1D2	NUP205	NUP98	PER1	PER2	PER3	PFAS
NUP98	PER1	PER2	PER3	PFAS	PHF21A	PIK3R4	PLEKHA5	PLXNA4	PLXNB1	PRR12	PRSS36	PTHLH	PTPRB	PTPRF	RFPL1/RFF
TMEM63B	TONSL	TRIML2	TRRAP	TSC1	UBR2	VPS13B	VPS13C	VPS13D	WDFY3	WDR17	WNK1	WT1			

MED13	MEFV	MST1	MSTN	NAALADL2	NACC2	NCOA1	NR1D2	NUP205	NUP98	PER1	PER2	PER3	PFAS	PHF21A	PIK3R4
MEFV	MST1	MSTN	NAALADL2	NACC2	NCOA1	NR1D2	NUP205	NUP98	PER1	PER2	PER3	PFAS	PHF21A	PIK3R4	PLEKHA5

LRP6	MCM3AP	MED13	MEFV	MST1	MSTN	NAALADL2	NACC2	NCOA1	NR1D2	NUP205	NUP98	PER1	PER2	PER3	PFAS
MED13	MEFV	MST1	MSTN	NAALADL2	NACC2	NCOA1	NR1D2	NUP205	NUP98	PER1	PER2	PER3	PFAS	PHF21A	PIK3R4

NR1D2	NUP205	NUP98	PER1	PER2	PER3	PFAS	PHF21A	PIK3R4	PLEKHA5	PLXNA4	PLXNB1	PRR12	PRSS36	PTHLH	PTPRB

PHF21A	PIK3R4	PLEKHA5	PLXNA4	PLXNB1	PRDM10	PRR12	PRSS36	PTHLH	PTPRB	PTPRF	RFPL1/RFF	RFPL4A/RF	RHBG	RNF123	RRAD
RHBG	RNF123	RRAD	SEC16A	SEMA5A	SHC4	SIK3	SLC22A7	SLC4A1	SLITRK3	SMG8	SQLE	SREBF1	SVEP1	TCF20	TECPR1
PLEKHA5	PLXNA4	PLXNB1	PRDM10	PRR12	PRSS36	PTHLH	PTPRB	PTPRF	RFPL1/RFF	RHBG	RNF123	RRAD	SEC16A	SEMA5A	SHC4
PLXNA4	PLXNB1	PRDM10	PRR12	PRSS36	PTHLH	PTPRB	PTPRF	RFPL1/RFF	RHBG	RNF123	RRAD	SEC16A	SEMA5A	SHC4	SIK3
PHF21A	PIK3R4	PLEKHA5	PLXNA4	PLXNB1	PRDM10	PRR12	PRSS36	PTHLH	PTPRB	PTPRF	RFPL1/RFF	RHBG	RNF123	RRAD	SEC16A
PLEKHA5	PLXNA4	PLXNB1	PRDM10	PRR12	PRSS36	PTHLH	PTPRB	PTPRF	RFPL1/RFF	RHBG	RNF123	RRAD	SEC16A	SEMA5A	SHC4
PTPRF	RFPL1/RFF	RHBG	RNF123	RRAD	SEC16A	SEMA5A	SHC4	SIK3	SLC22A7	SLC4A1	SLITRK3	SMG8	SQLE	SREBF1	SVEP1

SEC16A	SEMA5A	SHC4	SIK3	SLC22A7	SLC4A1	SLITRK3	SMG8	SQLE	SREBF1	SUZ12	SVEP1	TCF20	TECPR1	TG	TMEM63B
TG	TMEM63B	TNC	TONSL	TRIML2	TRRAP	UBR2	VAT1L	VPS13B	VPS13C	VPS13D	WDFY3	WDR17	WDR59	WDR90	WNK1
SIK3	SLC22A7	SLC4A1	SLITRK3	SMG8	SQLE	SREBF1	SUZ12	SVEP1	TCF20	TECPR1	TG	TMEM63B	TNC	TONSL	TRIML2
SLC22A7	SLC4A1	SLITRK3	SMG8	SQLE	SREBF1	SUZ12	SVEP1	TCF20	TECPR1	TG	TMEM63B	TNC	TONSL	TRIML2	TRRAP
SEMA5A	SHC4	SIK3	SLC22A7	SLC4A1	SLITRK3	SMG8	SQLE	SREBF1	SUZ12	SVEP1	TCF20	TECPR1	TG	TMEM63B	TNC
SIK3	SLC22A7	SLC4A1	SLITRK3	SMG8	SQLE	SREBF1	SUZ12	SVEP1	TCF20	TECPR1	TG	TMEM63B	TNC	TONSL	TRIML2
TCF20	TECPR1	TG	TMEM63B	TNC	TONSL	TRIML2	TRRAP	TSC1	UBR2	VAT1L	VPS13B	VPS13C	VPS13D	WDFY3	WDR17

TNC TONSL TRIML2 TRRAP TSC1 UBR2 VAT1L VPS13B VPS13C VPS13D WDFY3 WDR17 WDR59 WDR90 WNK1 WT1
WT1

TRRAP TSC1 UBR2 VAT1L VPS13B VPS13C VPS13D WDFY3 WDR17 WDR59 WDR90 WNK1 WT1
TSC1 UBR2 VAT1L VPS13B VPS13C VPS13D WDFY3 WDR17 WDR59 WDR90 WNK1 WT1

TONSL TRIML2 TRRAP TSC1 UBR2 VAT1L VPS13B VPS13C VPS13D WDFY3 WDR17 WDR59 WDR90 WNK1 WT1
TRRAP TSC1 UBR2 VAT1L VPS13B VPS13C VPS13D WDFY3 WDR17 WDR59 WDR90 WNK1 WT1

WDR59 WDR90 WNK1 WT1
