# Supplementary material for: Waveband specific transcriptional control of select genetic pathways in vertebrate skin (Xiphophorus maculatus)
Source: BMC Genomics. 2018 May 10;19:355. doi: 10.1186/s12864-018-4735-5 (PMC5946439; doi:10.1186/s12864-018-4735-5)
Supplement: Supplementary file 2 — Table S2a–k. A list of all differentially modulated genes used by IPA enrichment software to predict the direction of change for each functional class represented in Additional file 1: Table S1. Table a is FL, tables b–e are the 50 nm wavebands and tables g–k are the 10 nm wavebands. (ZIP 701 kb) [file 12864_2018_4735_MOESM2_ESM.zip › TableS2f_550-600nm.pdf]

Diseases or Functions Annotation	p-Value	Activation	# Genes	Genes							
inflammation	5.87E-05	-3.86	11	ATRN	COL11A1	COL1A1	COL1A2	DENND4B	ENO2	EPHB3	LRBA
cell movement	1.59E-07	-3.552	51	ADAMTS7	BCAS3	CD151	CELSR2	CNTNAP2	COL11A1	COL17A1	COL1A1
development of neurons	5.67E-05	-3.525	22	AGRN	ALS2	ARHGAP32	CELSR2	CNTNAP2	COL4A1	CUL7	EGR3
formation of cellular protrusions	1.55E-06	-3.5	27	AGRN	ALS2	ARHGAP32	BCAS3	CELSR2	CNTNAP2	CUL7	DNM1
neuritogenesis	1.43E-05	-3.23	19	AGRN	ALS2	ARHGAP32	CELSR2	CNTNAP2	CUL7	EGR3	EPHB3
quantity of endocrine cells	4.31E-03	-3.144	5	CACNA1D	COL5A3	INS	PKD1	TG			
branching of cells	1.72E-03	-3.001	11	AGRN	ARHGAP32	CNTNAP2	EGR3	EPHB3	EPHB4	MAGI2	PKD1
morphogenesis of neurites	1.95E-06	-2.859	17	AGRN	ALS2	ARHGAP32	CELSR2	CNTNAP2	CUL7	EGR3	EPHB3
sprouting	2.23E-04	-2.736	13	AGRN	ARHGAP32	CNTNAP2	EGR3	EPHB3	EPHB4	HSPG2	INS
quantity of neurons	3.80E-04	-2.707	13	AGRN	ALS2	CACNA1D	CNTNAP2	DNM1	EGR3	FLNA	GCG
quantity of cells	2.62E-04	-2.653	36	AGRN	ALS2	ARNTL2	BIRC6	CACNA1D	CD151	CNTNAP2	COL1A1
migration of cells	1.47E-06	-2.633	45	ADAMTS7	BCAS3	CD151	CELSR2	CNTNAP2	COL11A1	COL17A1	COL1A1
branching of neurites	2.18E-03	-2.581	9	AGRN	ARHGAP32	CNTNAP2	EGR3	EPHB3	MAGI2	PTPRF	RELN
microtubule arrangement	1.72E-05	-2.547	30	AGRN	ALS2	ARHGAP32	BCAS3	CELSR2	CNTNAP2	CUL7	DCLK2
differentiation of cells	1.78E-04	-2.501	44	ADAMTS20	ADAMTS7	AGRN	ARHGAP32	AXIN2	CACNA1D	CASZ1	CDON
dendritic growth/branching	6.52E-03	-2.446	7	AGRN	ARHGAP32	CNTNAP2	EPHB3	MAGI2	PTPRF	RELN	
collapse of growth cone	3.11E-04	-2.387	5	DNM1	EPHB3	NEO1	PLXNA1	PLXNB1			
vasculogenesis	2.79E-05	-2.354	22	ABRA	ADAMTS2	CD151	COL15A1	COL1A1	COL4A1	CUL7	EGR3
formation of filopodia	3.78E-03	-2.342	6	AGRN	BCAS3	DNM1	MKL1	NEO1	RELN		
generation of cells	8.89E-06	-2.325	44	ADAMTS2	ADAMTS7	AGRN	ALS2	ARHGAP32	AXIN2	CACNA1D	CD151
organization of cytoplasm	1.33E-05	-2.232	36	AGRN	ALS2	ARHGAP32	BCAS3	CELSR2	CNTNAP2	CUL7	DCLK2
formation of muscle	1.08E-05	-2.154	15	CDON	COL11A1	COL6A3	DNM1	EGR3	HSPG2	INS	KLHL41
invasion of cells	7.62E-03	-2.141	18	ADAMTS7	CD151	COL11A1	COL7A1	DOCK4	EPHB4	FAT1	FLNA
axonogenesis	2.37E-04	-2.11	9	AGRN	ALS2	ARHGAP32	EGR3	EPHB3	PARD3	PLXNB1	RELN
ploidy of cells	6.13E-03	-2.091	5	DCLK2	DOT1L	LRP6	MKL1	WNK1			
organization of cytoskeleton	4.34E-06	-2.07	35	AGRN	ALS2	ARHGAP32	BCAS3	CELSR2	CNTNAP2	CUL7	DCLK2
angiogenesis	3.50E-06	-2.049	27	ABRA	ADAMTS2	BIRC6	CD151	COL15A1	COL1A1	COL1A2	COL4A1
thoracic cancer	9.10E-05	2.103	26	AGRN	AXIN2	CD151	CLK4	CNTNAP2	COL11A1	COL12A1	DMXL2
scattering	1.14E-03	2.292	5	CD151	MKL1	PKD1	PTPN23	RELN			
fatty acid oxidation	2.76E-05	2.70	11	CIDEC	FASN	GCG	HMGA1	INS	NCOA1	PARD3	PTPRF
congenital malformation of brain	2.74E-05	2.951	12	CDON	CNTNAP2	COL4A1	FLNA	HSPG2	LAMB1	LRP6	NDST3
uptake of monosaccharide	3.63E-03	3.097	9	COL5A3	GCG	HMGA1	INS	NCOA1	PARD3	PTPRF	RRAD
Bleeding	4.07E-03	3.18	11	BIRC6	CACNA1D	CDON	COL1A1	COL4A1	CUL7	FLNA	HSPG2
midline defect	4.05E-03	3.395	8	EPHB3	FLNA	HSPG2	LRP6	PKD1	PTPRF	PTPRS	RERE
congenital malformation of skeleton	1.46E-03	3.429	12	AXIN2	CDON	EPHB3	FLNA	GDF6	GLI3	HSPG2	LRP6
uptake of D-glucose	2.94E-03	3.488	8	COL5A3	GCG	HMGA1	INS	NCOA1	PARD3	PTPRF	SST
cell proliferation	3.30E-10	3.50	72	ADAMTS7	ALS2	AXIN2	BIRC6	BPTF	CACNA1D	CASZ1	CD151
congenital anomaly of musculoskelet	3.39E-07	3.613	25	ADAMTS2	ALS2	AXIN2	CDON	COL11A1	COL1A1	COL1A2	COL4A1
neonatal death	4.02E-03	3.724	11	BIRC6	CDON	CUL7	DNM1	LRP6	MAGI2	NDST3	PHF21A
perinatal death	3.46E-04	4.501	16	AGRN	BIRC6	CDON	COL12A1	CUL7	DNM1	HSPG2	LRP6
organismal death	1.53E-05	4.529	49	AGRN	BIRC6	BPTF	CASZ1	CD151	CDON	COL11A1	COL12A1

SLIT3	TAX1BP1	XIRP1											
COL4A1	COL7A1	DNM1	DOCK4	EPHB3	EPHB4	FASN	FAT1	FAT3	FLNA	GLI3	HMGA1	INS	LAMB1
EPHB3	GLI3	HERC1	LAMB1	LRP6	MAGI2	PARD3	PLXNB1	PTPRF	RELN	RERE	SLIT3	TNIK	ZDHHC8
EGR3	EPHB3	FASN	FLNA	HERC1	KLHL41	LAMB1	MAGI2	MKL1	NEO1	PARD3	PLXNB1	PTPN23	PTPRF
HERC1	LAMB1	MAGI2	PARD3	PLXNB1	PTPRF	RELN	RERE	SLIT3	TNIK	ZDHHC8			
PTPRF	RELN	ZDHHC8											
HERC1	LAMB1	MAGI2	PLXNB1	PTPRF	RELN	RERE	TNIK	ZDHHC8					
MAGI2	PKD1	PTPRF	RELN	ZDHHC8									
HSPB8	PTPRF	PTPRS	RELN	RERE									
COL5A3	CUL7	DNM1	DOT1L	EGR3	FLNA	GCG	GDF6	HMGA1	HSPB8	HSPG2	INS	JARID2	JMJD1C
COL4A1	COL7A1	DOCK4	EPHB3	EPHB4	FASN	FAT1	FAT3	FLNA	GLI3	INS	LAMB1	LAMB3	LRP6
ZDHHC8													
DNM1	DOCK4	EGR3	EPHB3	FASN	FLNA	HERC1	KLHL41	LAMB1	MAGI2	MKL1	NEO1	PARD3	PLXNB1
CNTNAP2	COL24A1	COL4A1	DOCK4	DOT1L	EGR3	EPHB3	EPHB4	FASN	GCG	GDF6	GLI3	HMGA1	HSPG2
EPHB3	EPHB4	FLNA	GLI3	HSPG2	INS	NCOA1	PLXNB1	PRKDC	PTPRB	SLIT3	SST	TAB2	WNK1
CELSR2	CNTNAP2	COL11A1	COL1A1	COL4A1	CUL7	EGR3	EPHB3	EPHB4	FLNA	GCG	GLI3	HERC1	HERC2
DNM1	DOCK4	EGR3	EPHB3	FASN	FAT1	FLNA	HERC1	INS	KLHL41	LAMB1	MAGI2	MKL1	NEO1
NEO1	PLXNB1	SCN8A	SLIT3	SVIL	TCAP	XIRP1							
HMGA1	LAMB3	MAGI1	NCOA1	PLXNB1	PTPN23	SCN8A	SST	SUZ12	WNK1				
ZDHHC8													
DNM1	DOCK4	EGR3	EPHB3	FASN	FAT1	FLNA	HERC1	INS	KLHL41	LAMB1	MAGI2	MKL1	NEO1
COL5A1	CUL7	EGR3	EPHB3	EPHB4	FLNA	GLI3	HSPG2	INS	NCOA1	PKD1	PLXNB1	PRKDC	PTPRB
EGR3	ENO2	EPHB4	FASN	FAT1	HERC2	LAMB3	LAMB4	MAGI1	NAV3	NCOA1	PLXNA1	PLXNB1	POLR2A
SST	TG	TGM1											
PTPRF	PTPRS	RELN	RERE										
SST													
INS	PKD1	TAB2											
NDST3	PKD1	PTPRF	PTPRS										
CDON	CELSR2	CLEC19A	CNTNAP2	COL11A1	COL12A1	COL15A1	COL1A2	COL21A1	COL24A1	COL4A1	COL5A3	COL6A3	DENND4B
COL5A1	COL6A3	EPHB3	FLNA	GDF6	GLI3	HSPG2	KLHL40	KLHL41	LMOD3	LRP6	NDST3	PIEZO2	PKD1
PKD1	PTPRS	TGM1											
MAGI2	MKL1	NDST3	PHF21A	PKD1	PTPRS	TGM1	UNC79						
COL1A1	COL4A1	COL5A1	COL7A1	CUL7	DNM1	DOT1L	EGR3	EPHB3	FASN	FAT1	FLNA	GCG	GDF6

LAMB3	LRP6	MAGI1	MAGI2	MKL1	NDST3	NEO1	PARD3	PCOLCE2	PKD1	PLXNA1	PLXNB1	PTPN23	PTPRF
RELN	RERE	SLIT3	TNIK	ZDHHC8									
KLF13	LRP6	MKL1	NDST3	NEO1	PER2	PKD1	PRKDC	PTPRF	PTPRS	RELN	RERE	SST	TG
MAGI1	MAGI2	MKL1	NDST3	NEO1	PCOLCE2	PKD1	PLXNA1	PLXNB1	PTPRF	RAMP1	RELN	RERE	SEMA4C
PTPN23	PTPRF	RELN	RERE	RRAD	SLIT3	TNIK	ZDHHC8						
INS	JARID2	JMJD1C	KLF13	KLHL41	LAMB3	LRP6	MAGI2	MKL1	NCOA1	PKD1	PRKDC	PRRC2C	PTPRF
HMGA1	HSPG2	JMJD1C	LAMB1	LRP6	MAGI2	MKL1	NCOA1	PARD3	PKD1	PLXNB1	PRKDC	PTPRF	RELN
PARD3	PKD1	PLXNB1	PRKDC	PTPN23	PTPRF	RELN	RERE	RRAD	SEC16A	SLIT3	STK35	TNIK	ZDHHC8
PARD3	PKD1	PLXNB1	PRKDC	PTPN23	PTPRF	RELN	RERE	RRAD	SLIT3	STK35	TNIK	ZDHHC8	
RAMP1	SLIT3	SST	TAB2	WNK1									
PRKDC	STK35	WNK1	XIRP1										
DMXL2	DNM1	DOCK4	EGR3	FASN	FAT1	FAT3	FBRS	FBRSL1	GDF6	GLI3	HERC2	HMGA1	HSPG2
PTPRF	PTPRS	TCAP											
HSPB8	HSPG2	INS	KLHL40	LRP6	MAGI2	MCM3AP	MKL1	MRC1	NCOA1	NDST3	PARD3	PER2	PHF21A

RAMP1	RELN	RERE	SEMA4C	SHC4	SLIT3	SMG1	SST	STK35	SUZ12	TAB2	TAX1BP1	TG	WNK1
SHC4	SLIT3	SST	STK35	SUZ12	TAB2	TAX1BP1	TG	ZNF703					
RELN	RRAD	SEMA4C	SHC4	SUZ12	TG	TGM1	ZDHHC8						
RERE	SLIT3	SST	TAB2	TCAP	TNIK	WNK1	ZDHHC8						
JARID2	KLF11	KLHL30	KLHL41	LRBA	MAGI1	MAGI2	MRC1	MURC	NAV3	NCOA7	NHSL1	PARD3	PCOLCE2
PKD1	POLR2A	PRKDC	PTPRF	PTPRS	SMG1	SUZ12	TAB2	TAX1BP1	TENM3	TGM1	TRRAP	UNC79	

ZNF703

PHF21A	PKD1	PLXNB1	PRKDC	PRR12	PTPRF	PTPRS	RELN	SCN8A	SLIT3	STK35	SUZ12	SVEP1	SVIL

TAX1BP1	TNIK	TNRC18	TRRAP	TTC28	UNC79	VPS13B	ZNF668
