# Supplementary material for: Waveband specific transcriptional control of select genetic pathways in vertebrate skin (Xiphophorus maculatus)
Source: BMC Genomics. 2018 May 10;19:355. doi: 10.1186/s12864-018-4735-5 (PMC5946439; doi:10.1186/s12864-018-4735-5)
Supplement: Supplementary file 2 — Table S2a–k. A list of all differentially modulated genes used by IPA enrichment software to predict the direction of change for each functional class represented in Additional file 1: Table S1. Table a is FL, tables b–e are the 50 nm wavebands and tables g–k are the 10 nm wavebands. (ZIP 701 kb) [file 12864_2018_4735_MOESM2_ESM.zip › TableS2e_500-550nm.pdf]

Diseases or Functions	p-Value	Activation	# Genes	Genes						
G2/M Phase	6.30E-03	-3.61	13	ALPK3	ARID1A	COL11A1	DENND4B	GLB1L3	GLTSCR1	KIF21A
hypertrophy of heart	1.29E-06	-3.368	17	ANKRD1	CSRP3	FBXO32	HMOX1	HSPB8	JARID2	MAPK8
DNA repair	2.62E-03	-3.00	45	ACSL1	ALPK3	AMPD1	ARID1A	ASB10	BCAP29	BTBD11
fibrosis of heart	1.19E-03	-2.664	8	ACSL1	CSRP3	HMOX1	HSPB8	MAPK8	PFKFB1	RERE
chromosomal alignment	1.38E-08	-2.52	66	ABCB11	ARID1A	BHLHE40	BST1	CALCA	CAMK1G	CAPN3
Hypertrophy	2.38E-07	-2.368	17	ANKRD1	CSRP3	FBXO32	HMOX1	HSPB8	JARID2	MAPK8
cell death of muscle cell	9.33E-04	-2.205	12	ANKRD1	CALCA	GAPDH	HMOX1	HSPB8	KLHL40	MAPK8
M phase	9.03E-05	-2.17	26	ARID1A	CAMK1G	CAPN3	CELSR2	CHRNA4	CTNNA2	DHH
inflammation	5.70E-05	-2.14	8	C4A/C4B	CALCA	DGKZ	FBXO32	GATA3	HMOX1	MAPK8
S phase	4.47E-03	-2.03	40	ABCB11	ABHD8	ABRA	AMPD1	ARID1A	BST1	CELSR2
development of body tru	1.30E-03	-2	30	ALPK3	ANKRD1	ARID1A	CAPN3	COL11A1	CSRP3	CYP1A2
proliferation of embryon	9.53E-03	2.067	6	ESM1	HSP90AA1	HSPA4	MYOC	NOX1	PTP4A3	
lung cancer	7.66E-03	2.103	25	ARID1A	CALCA	CD151	CHRNA4	CLK1	COL11A1	DNAJB4
fatty acid oxidation	8.63E-05	2.11	12	CETP	DGKZ	GAPDH	GPD1	GYS2	NOX1	PFKFB1
adipogenesis of cells	8.93E-03	2.179	5	BHLHE40	GATA3	ME1	NR4A3	PER3		
adipogenesis	2.77E-03	2.179	7	BHLHE40	CREB5	GATA3	ME1	NR4A3	PER3	PPARGC1A
contraction of heart	5.72E-06	2.206	11	CALCA	CSRP3	HMOX1	KCNJ12	LMOD2	MAPK8	SCN1B
function of heart	2.96E-05	2.215	12	ABRA	CSRP3	FBXO32	HMOX1	HSPB8	MAPK8	PFKFB1
activation of DNA endog	5.33E-03	2.657	27	ABRA	ANKRD1	ARID1A	BHLHE40	CALCOCO	CREB5	CSRP3
cancer of cells	2.07E-03	2.732	64	ABCB11	ADAM18	ARID1A	CAMTA1	CD151	CELSR2	CHRNB1
transcription of cells	4.25E-03	2.744	33	ABRA	ANKRD1	ARID1A	BHLHE40	CALCA	CALCOCO	CAPN3
heart rate	1.23E-04	2.761	12	CALCA	CSRP3	HMOX1	KCNJ12	LMOD2	MAPK8	PPARGC1A
neoplasia of cells	1.81E-03	2.841	66	ABCB11	ADAM18	ARID1A	CAMTA1	CD151	CELSR2	CHRNB1
contractility of cardiac m	6.26E-07	2.854	11	CSRP3	FBXO32	HMOX1	HSPB8	MAPK8	PFKFB1	PPARGC1A
consumption of oxygen	2.69E-03	2.887	7	ACSL1	CREB5	HMOX1	NR4A3	PKM	PPARGC1A	SIK3
function of muscle	5.61E-05	2.892	14	CALCA	CSRP3	FBXO32	HMOX1	HSPB8	MAPK8	PFKFB1
contractility of muscle	2.93E-07	3.287	13	CSRP3	FBXO32	HMOX1	HSPB8	MAPK8	PDK2	PFKFB1
lipid oxidation	1.37E-04	3.95	14	ABCB11	ACSL1	ITPR3	MAPK8	MTNR1B	MVP	PDK2

	LARP4B	MYH13	MYOM1	PSME4	STEAP4	SVIL							
	MYOM1	NR4A3	PFKFB1	PFKM	PPARGC1A	RRAD	TCAP	TRIM55	TRIM63	XIRP1			
	CHRNA4	CORO1C	CTNNA2	CYP1A2	CYP2W1	DNAJB5	DPP10	DUSP26	EEF2	EPRS	EPX	GATA3	HIPK3
	XIRP1												
	CD151	CELSR2	CHRNA4	CHRNB1	CREB5	CSRP3	CTNNA2	CYP1A2	DGKZ	DHH	DKK2	EEF2	EPX
	MYOM1	NR4A3	PFKFB1	PFKM	PPARGC1A	RRAD	TCAP	TRIM55	TRIM63	XIRP1			
	NR4A3	PFKM	PPARGC1A	RRAD	TNNT1								
	FAM107A	GAPDH	KIRREL3	LGI1	LMOD2	MAPK8	MYOC	MYOM1	NR4A3	NYAP2	OBSL1	PTPRF	RERE
	MTMR4												
	CLK1	CTNNA2	DENND4B	DPP10	ESYT2	FHL1	GAPDH	GATA3	KBTBD3	KIF21A	KIRREL3	KLHL30	LRBA
	DHRS3	FHL1	GATA3	HMOX1	HSPB11	HSPB8	JARID2	LMOD2	MAPK8	MYF6	PKM	PPARGC1A	PRKDC
	FHL1	GATA3	GLB1L3	HSP90AA1	ITPR3	KIF21A	MAGI1	MTNR1B	OPCML	PFKM	PKM	POLR2A	PPP1R3A
	PFKM	PIK3C2B	PKM	PPARGC1A	PPP1R3A								
PPARGC1A													
	SP4	TCAP	TNNI1	TRIM63									
	PPARGC1A	RRAD	TRIM55	TRIM63	XIRP1								
	DNAJB5	DUSP22	DUSP26	GATA3	HELT	HIPK3	HIVEP1	JARID2	MED13	MYF6	NR4A3	PER3	POLR2A
	CLK1	COL11A1	COL4A6	CSRP3	CTNNA2	DENND4B	DKK2	DPP10	EPX	ESYT2	FAM134B	FAM219A	FRMD3
	CELSR2	CREB5	CSRP3	DNAJB5	DUSP22	DUSP26	GATA3	HELT	HIPK3	HIVEP1	JARID2	MAPK8	MED13
	SCN1B	SP4	TCAP	TNNI1	TRIM63								
	CLK1	COL11A1	COL4A6	CSRP3	CTNNA2	DENND4B	DKK2	DNAJB4	DPP10	EPX	ESYT2	FAM134B	FAM219A
	RRAD	TRIM55	TRIM63	XIRP1									
	PPARGC1A	RRAD	TNNT1	TRIM55	TRIM63	WFIKKN2	XIRP1						
	PPARGC1A	RRAD	TNNT1	TRIM55	TRIM63	XIRP1							
	PGAM2	PTP4A3	PTPRF	RRAD	SSTR2	STEAP4	USP13						

ITPR3	JARID2	KBTBD3	KIF21A	KIRREL3	LMOD2	LRBA	MYF6	MYOM1	NETO1	NRAP	NYAP2	OBSL1
FAM107A	FAM134B	FBXO32	GAPDH	GATA3	HMOX1	HSP90AA1	HSPA4	ITPR3	KIRREL3	KLHL40	LGI1	LMOD2
SLIT3	SLITRK3	SP4	TCAP	TNIK	XIRP1							
LRRC30	MAP3K20	MYH13	NOX1	NRAP	OPCML	PFAS	PFKM	PIK3C2B	PKM	PPP1R3A	PRKDC	SIK3
PTPRF	SIK3	TCAP	TLX1	TNNI1	TP53BP2	TRIM55	TRIM63	XIRP1	XIRP2			
PROM1	PSME4	SCN1B	SLITRK3	TLX1								
PPARGC1/	PRKDC	SP4	TAF3	TLX1	VGLL2	ZBTB16						
FRMD5	GATA3	GLB1L3	GYS2	HMOX1	HSP90AA1	IQSEC2	JARID2	KCNF1	KIF21A	KLHL30	MAGI1	MAP3K20
MYF6	NR4A3	PER3	POLR2A	PPARGC1/	PRKDC	SP4	TAF3	TLX1	USP13	VGLL2	ZBTB16	ZNF703
FRMD3	FRMD5	GATA3	GLB1L3	GYS2	HMOX1	HSP90AA1	IQSEC2	JARID2	KCNF1	KIF21A	KLHL30	MAGI1

PER3	PKM	PPP1R3A	PRKDC	PSME4	PTPRF	RHCG	SLITRK3	SYNPO2L	TENM1	WDR17	XIRP2	
MAPK8	MYF6	MYOC	MYOM1	NOX1	NYAP2	OBSL1	PFKFB1	PFKM	PPARGC1A	PPP1R3A	PRKDC	PROM1
SLITRK3	SVIL	SYNPO2L	TNNI1	TP53BP2	TRIM63	XIRP2						
MTMR4	NR4A3	NYAP2	OBSL1	OPCML	PER3	PIK3C2B	PKM	PPARGC1A	PPP1R3A	PRKDC	PRR12	PRRC2A
MAP3K20	MTMR4	NR4A3	NYAP2	OBSL1	OPCML	PER3	PIK3C2B	PKM	PPARGC1A	PPP1R3A	PRKDC	PROM1

PSME4	PTPRF	RRAD	RERE	SCN1B	SIK3	SLIT3	SLITRK3	SP4	TCAP	TENM1	TLX1	TNIK

PSME4	PTPRF	PYGM	RPH3A	SERPING1	SLIT3	SLITRK3	SP4	SSTR2	STEAP4	TLX1	TNIK	TNNI1
PRR12	PRRC2A	PSME4	PTPRF	PYGM	RPH3A	SERPING1	SLIT3	SLITRK3	SP4	SSTR2	STEAP4	TLX1

TNNT1	TP53BP2	TRIM55	TRIM63	WDR17	XIRP1	ZBTB16
TP53BP2	TXLNB	WDR17	XIRP2	ZBTB16		
TNIK	TNNI1	TP53BP2	TXLNB	WDR17	XIRP2	ZBTB16
