# Supplementary material for: Waveband specific transcriptional control of select genetic pathways in vertebrate skin (Xiphophorus maculatus)
Source: BMC Genomics. 2018 May 10;19:355. doi: 10.1186/s12864-018-4735-5 (PMC5946439; doi:10.1186/s12864-018-4735-5)
Supplement: Supplementary file 2 — Table S2a–k. A list of all differentially modulated genes used by IPA enrichment software to predict the direction of change for each functional class represented in Additional file 1: Table S1. Table a is FL, tables b–e are the 50 nm wavebands and tables g–k are the 10 nm wavebands. (ZIP 701 kb) [file 12864_2018_4735_MOESM2_ESM.zip › TableS2d_450-500nm.pdf]

Functional Class	p-Value	Activation	# Genes	Genes
glucose metabolism disorder	4.65E-09	-3.736	24	ACSS1 ATP2A2 CA4 CEBPB CREB5 GCK HP HSD17B1 HYOU1 IL1B IL1R2 KDR LDLR MME MMP9 PTAFR RGS5 SOAT1 STEAP4 TGM2 TLR2 TLR5 TNFRSF9 VEPH1
concentration of D-glucose	7.58E-04	-3.169	7	ATP2A2 GCK IL1B LDLR MMP9 STEAP4 TLR2
infection of mammalia	1.54E-08	-3.149	12	C5AR1 CEBPB CH25H CSF3 IL1B MMP9 PTAFR PTX3 RIPK3 TLR2 TLR5 TNFRSF9
insulin resistance	9.75E-09	-2.98	12	ATP2A2 CA4 GCK HYOU1 IL1B KDR LDLR SOAT1 STEAP4 TGM2 TLR2 TLR5
Organ Degeneration	8.91E-05	-2.959	10	CA4 GGT1 GNAT1 IL1B KDR LDLR MME MMP9 RHO TLR2
organismal death	2.23E-08	-2.923	32	ACSS1 ANGPT2 ATP2A2 C5AR1 CA4 CEBPB CREB5 CSF3 CYP24A1 F8 GCK GGT1 HP HSD17B1 HSP90B1 HYOU1 IL1B KDR LDLR MME MMP9 PFKFB3 PTAFR PTX3 RIPK3 SALL1 SLC11A2 TGM2 TIMP2 TLR2 TLR5 TNFRSF9
obesity	2.10E-04	-2.72	9	ANGPT2 CA4 CEBPB GCK IL1B LDLR MMP9 TIMP2 TLR5
retinal degeneration	1.04E-04	-2.66	7	CA4 GNAT1 IL1B KDR LDLR RHO TLR2
apoptosis of neurons	1.64E-04	-2.475	9	ADAM8 C5AR1 CEBPB CSF3 HYOU1 IL1B KDR MMP9 TLR2
quantity of muscle cells	9.34E-05	-2.452	5	ANGPT2 HSP90B1 IL1B LDLR TNFRSF9
neuronal cell death	5.19E-04	-2.113	11	ADAM8 C5AR1 CEBPB CSF3 HYOU1 IL1B KDR MMP9 SLC11A2 TGM2 TLR2
apoptosis	8.50E-06	-2.04	34	ADAM8 ANGPT2 ANKRD1 ARG2 BCL6B C5AR1 CA4 CEBPB CSF3 GCK GGT1 GNAT1 HSH2D HSP90B1 HYOU1 IGFBP6 IL1B KDR LDLR MME MMP9 NACC2 PTAFR RGS5 RHO RIPK3 S1PR4 SALL1 SOAT1 TGM2 TIMP2 TLR2 TNFAIP2 TNFRSF9
stress response of cells	4.54E-05	2	6	ANGPT2 ATP2A2 CEBPB HSP90B1 IL1B MMP9
synthesis of prostaglandin	1.74E-04	2.015	6	CEBPB IL1B IL1R2 LDLR PTAFR SOAT1
recruitment of leukocytes	2.06E-07	2.018	11	ANGPT2 C5AR1 CSF3 IL1B KDR LTB4R MMP9 PTX3 TLR2 TLR5 TNFRSF9
synthesis of prostaglandin E2	4.57E-04	2.023	5	CEBPB IL1B IL1R2 PTAFR SOAT1
apoptosis of phagocytes	1.61E-07	2.04	9	ADAM8 CEBPB CSF3 IL1B LDLR RIPK3 SOAT1 TLR2 TNFRSF9
cellular infiltration by macrophages	4.00E-07	2.04	8	ANGPT2 C5AR1 IL1B KDR LDLR PTX3 TLR2 TNFRSF9
cell death of tumor cell lines	1.51E-04	2.058	19	ANGPT2 ANKRD1 ATP2A2 BCL6B C5AR1 CEBPB GGT1 HCAR1 HSH2D IGFBP6 IL1B MME MMP9 NACC2 PTAFR RIPK3 SLC11A2 TGM2 TLR2
engulfment of myeloid cells	6.75E-09	2.062	9	CEBPB CH25H CSF3 HYOU1 IL1B PTX3 SOAT1 TGM2 TLR2
quantity of myeloid cells	3.38E-07	2.075	11	ADAM8 BCL6B C5AR1 CSF3 IL1B LDLR MMP9 TIMP2 TLR2 TLR5 TNFRSF9
apoptosis of antigen presenting cells	1.12E-07	2.076	8	ADAM8 CEBPB CSF3 IL1B LDLR RIPK3 SOAT1 TLR2
activation of central nervous system cells	9.81E-07	2.092	6	ANGPT2 CEBPB CSF3 IL1B MMP9 TLR2
growth of epithelial tissue	3.90E-06	2.1	14	ANGPT2 C5AR1 CEBPB IL1B KDR LTB4R MMP9 PFKFB3 PTAFR RGS5 TGM2 TIMP2 TLR2 TYRP1
quantity of granulocytes	8.74E-07	2.109	10	ADAM8 BCL6B C5AR1 CSF3 IL1B LDLR MMP9 TIMP2 TLR2 TLR5
neovascularization of choroid	8.70E-07	2.114	5	C5AR1 IL1B KDR MMP9 TIMP2
cellular infiltration of phagocytes	1.10E-07	2.117	9	ANGPT2 C5AR1 IL1B KDR LDLR MMP9 PTX3 TLR2 TNFRSF9
infiltration by neutrophils	3.08E-08	2.123	9	ADAM8 ANGPT2 C5AR1 CSF3 IL1B LTB4R MMP9 PTX3 TLR2
recruitment of neutrophils	1.18E-04	2.125	6	C5AR1 IL1B MMP9 PTX3 TLR2 TLR5
activation of endothelial cells	2.52E-07	2.146	6	ANGPT2 CSF3 IL1B KDR MMP9 TLR2
expansion of mononuclear leukocytes	8.86E-04	2.151	5	C5AR1 CSF3 IL1B TLR2 TNFRSF9
fatty acid metabolism	4.95E-05	2.155	12	ACSS1 APOF C5AR1 CEBPB GGT1 IL1B IL1R2 LDLR LTB4R PTAFR SOAT1 TLR2
synthesis of eicosanoid	1.22E-07	2.158	10	C5AR1 CEBPB GGT1 IL1B IL1R2 LDLR LTB4R PTAFR SOAT1 TLR2
response of myeloid cells	1.81E-08	2.165	10	C5AR1 CEBPB CH25H CSF3 IL1B PTAFR PTX3 SOAT1 TGM2 TLR2
quantity of metal	5.55E-05	2.165	11	ANGPT2 ATP2A2 C5AR1 GNAT1 HP HYOU1 IL1B KDR SLC11A2 TGM2 TNFRSF9
cellular homeostasis	6.16E-05	2.177	20	ANGPT2 ATP2A2 C5AR1 CEBPB GCK GGT1 HSP90B1 IL1B KDR LDLR LTB4R RIPK3 S1PR4 SLC11A2 SOAT1 TGM2 TIMP2 TLR2 TLR5 TNFRSF9
apoptosis of myeloid cells	1.31E-07	2.181	9	ADAM8 CEBPB CSF3 IL1B LDLR RIPK3 SOAT1 TLR2 TNFRSF9
hypersensitive reaction	1.01E-04	2.195	9	ANGPT2 CEBPB GPR84 IL1B MME MMP9 PTAFR TLR2 TNFRSF9
quantity of cells	2.57E-08	2.198	27	ADAM8 ANGPT2 BCL6B C5AR1 CEBPB CSF3 GGT1 GNAT1 HSP90B1 IGFBP6 IL1B KDR LDLR LTB4R MME MMP9 PTAFR RHO RIPK3 SLC11A2 SOAT1 STEAP4 TGM2 TIMP2 TLR2 TLR5 TNFRSF9
response of phagocytes	3.27E-08	2.206	10	C5AR1 CEBPB CH25H CSF3 IL1B PTAFR PTX3 SOAT1 TGM2 TLR2
transmigration of leukocytes	1.39E-04	2.21	5	IL1B LDLR MMP9 TGM2 TIMP2
arthritis	2.69E-05	2.22	15	ADAM8 C5AR1 CEBPB CSF3 HP HSP90B1 IL1B IL1R2 KDR LTB4R MME MMP9 SQLE STEAP4 TLR2
stimulation of leukocytes	1.02E-05	2.221	7	C5AR1 CSF3 HSP90B1 IL1B TLR2 TLR5 TNFRSF9
cell movement of mononuclear leukocytes	1.62E-07	2.221	13	ADAM8 ANGPT2 CSF3 HP HYOU1 IL1B KDR LDLR MMP9 TGM2 TIMP2 TLR2 TNFRSF9
quantity of neutrophils	3.95E-07	2.237	9	BCL6B C5AR1 CSF3 IL1B LDLR MMP9 TIMP2 TLR2 TLR5
quantity of leukocytes	3.29E-08	2.245	19	ADAM8 BCL6B C5AR1 CEBPB CSF3 HSP90B1 IL1B LDLR LTB4R MMP9 PTAFR RIPK3 SOAT1 STEAP4 TGM2 TIMP2 TLR2 TLR5 TNFRSF9
cell viability	6.00E-09	2.25	54	ADAM8 ANGPT2 ANKRD1 ARG2 ATP2A2 BCL6B C5AR1 CA4 CEBPB CSF3 CYP24A1 DENND4A DNAH7 F13B F8 GCK GGT1 GNAT1 HCAR1 HP HSD17B1 HSH2D HSP90B1 HYOU1 IGFBP6 IL1B KDR LDLR MME MMP9 NACC2 OCSTAMP PFKFB3 PTAFR RGS5 RHO RIPK3 S1PR4 SALL1
permeability of cells	6.74E-05	2.252	5	ANGPT2 C5AR1 IL1B KDR TIMP2
generation of cells	5.07E-04	2.26	21	ANGPT2 C5AR1 CEBPB CSF3 GGT1 GNAT1 HSP90B1 IL1B KDR LDLR MMP9 PFKFB3 RGS5 RHO RIPK3 SLC11A2 SOAT1 TIMP2 TLR2 TLR5 TNFRSF9
extravasation	9.48E-08	2.264	7	ADAM8 ANGPT2 IL1B LDLR MME PTAFR TLR2
migration of carcinoma cell lines	6.74E-04	2.27	5	CEBPB CSF3 IL1B TGM2 TLR2
stimulation of phagocytes	5.44E-06	2.274	5	C5AR1 CSF3 IL1B TLR2 TLR5
vascularization	5.01E-07	2.299	9	ARHGEF4 C5AR1 CSF3 HYOU1 IL1B KDR MMP9 TIMP2 TLR2
proliferation of lymphatic system cells	1.78E-04	2.303	6	CSF3 IL1B KDR LTB4R TLR2 TNFRSF9
quantity of macrophages	1.61E-06	2.312	8	ADAM8 C5AR1 IL1B LDLR MMP9 SOAT1 STEAP4 TGM2
adhesion of blood cells	7.76E-05	2.315	9	ANGPT2 IL1B LTB4R OCSTAMP PTX3 TGM2 TLR2 TLR5 TNFRSF9
angiogenesis of lesion	1.15E-04	2.342	5	CSF3 IL1B KDR MMP9 TIMP2
recruitment of granulocytes	3.42E-05	2.351	7	C5AR1 IL1B LTB4R MMP9 PTX3 TLR2 TLR5
activation of macrophages	9.80E-05	2.369	7	ANGPT2 CEBPB CSF3 IL1B MMP9 TGM2 TLR2
development of body trunk	4.57E-05	2.38	17	ANKRD1 ATP2A2 CEBPB CSF3 GGT1 HP IL1B KDR MMP9 RIPK3 S1PR4 SALL1 TGM2 THRSP TIMP2 TLR2 XIRP2
delayed hypersensitive reaction	1.35E-04	2.387	5	ANGPT2 CEBPB IL1B MMP9 TNFRSF9
metastasis of tumor cell lines	9.04E-04	2.4	5	ANGPT2 CSF3 HCAR1 MMP9 TLR2
migration of smooth cells	4.15E-05	2.406	6	ANGPT2 IL1B KDR LDLR MMP9 TLR2
adhesion of connective tissue cells	4.04E-04	2.41	5	ANGPT2 IL1B LDLR MME TIMP2
cell movement of leukocytes	1.71E-07	2.412	17	ADAM8 ANGPT2 C5AR1 CSF3 HP HYOU1 IL1B KDR LDLR LTB4R MMP9 PTX3 S1PR4 TGM2 TIMP2 TLR2 TNFRSF9
Rheumatic Disease	3.93E-05	2.423	16	ADAM8 C5AR1 CEBPB CSF3 HP HSP90B1 IL1B IL1R2 KDR LTB4R MME MMP9 SQLE STEAP4 TLR2 TNFRSF9
cell movement of phagocytes	5.34E-08	2.427	15	ADAM8 ANGPT2 C5AR1 CSF3 HP IL1B KDR LDLR LTB4R MMP9 PTX3 S1PR4 TIMP2 TLR2 TNFRSF9
apoptosis of leukocytes	5.30E-05	2.434	10	ADAM8 CEBPB CSF3 HSH2D IL1B LDLR RIPK3 SOAT1 TLR2 TNFRSF9
response of granulocytes	5.56E-05	2.446	5	C5AR1 CSF3 PTAFR PTX3 TLR2
cell movement of monocytes	5.02E-06	2.455	7	ANGPT2 CSF3 HP IL1B LDLR MMP9 TIMP2
immune response of cells	9.37E-05	2.455	11	C5AR1 CEBPB CH25H CSF3 IL1B PTX3 SOAT1 TGM2 TLR2 TLR5 TNFRSF9
development of abdomen	2.89E-04	2.461	10	CEBPB CSF3 GGT1 HP IL1B KDR MMP9 RIPK3 SALL1 TIMP2
neovascularization	4.28E-05	2.462	6	C5AR1 CSF3 IL1B KDR MMP9 TIMP2
quantity of Ca2+	2.93E-04	2.464	9	ANGPT2 ATP2A2 C5AR1 GNAT1 HYOU1 IL1B KDR TGM2 TNFRSF9
chronic inflammatory disorder	1.59E-04	2.471	14	ADAM8 C5AR1 CEBPB CSF3 HP HSP90B1 IL1B IL1R2 MME MMP9 SQLE STEAP4 TLR2 TLR5
chemotaxis of phagocytes	3.11E-04	2.474	7	ANGPT2 C5AR1 HP IL1B LDLR LTB4R TLR2
activation of neutrophils	1.64E-06	2.476	6	C5AR1 CSF3 IL1B LTB4R TLR2 TNFRSF9
synthesis of reactive oxygen species	3.82E-04	2.495	10	ANGPT2 C5AR1 CSF3 GGT1 HP IL1B RIPK3 TGM2 TLR2 TLR5
necrosis	4.27E-05	2.50	37	ADAM8 ANGPT2 ANKRD1 ARG2 ATP2A2 BCL6B C5AR1 CA4 CEBPB CSF3 DENND4A GCK GGT1 HCAR1 HSH2D HSP90B1 HYOU1 IGFBP6 IL1B KDR LDLR MME MMP9 NACC2 PFKFB3 PTAFR RGS5 RIPK3 S1PR4 SALL1 SLC11A2 SOAT1 TGM2 TIMP2 TLR2 TNFRSF9 TYRP1
production of superoxide	2.96E-05	2.519	6	ANGPT2 C5AR1 CSF3 IL1B TGM2 TLR2
immune response of leukocytes	1.09E-06	2.53	10	C5AR1 CEBPB CH25H CSF3 IL1B PTX3 SOAT1 TGM2 TLR2 TNFRSF9
cell death	2.85E-11	2.563	41	ADAM8 ANGPT2 ANKRD1 ARG2 ATP2A2 BCL6B C5AR1 CA4 CEBPB CSF3 DENND4A GCK GGT1 GNAT1 HCAR1 HP HSH2D HSP90B1 HYOU1 IGFBP6 IL1B KDR LDLR MME MMP9 NACC2 PFKFB3 PTAFR RGS5 RHO RIPK3 S1PR4 SALL1 SLC11A2 SOAT1 TGM2 TIMP2 TLR2 TNFAIP2
proliferation of epithelial cells	9.24E-05	2.564	10	C5AR1 CEBPB IL1B KDR LTB4R MMP9 PTAFR TGM2 TLR2 TYRP1
quantity of phagocytes	3.65E-08	2.565	13	ADAM8 BCL6B C5AR1 CSF3 IL1B LDLR MMP9 SOAT1 STEAP4 TGM2 TIMP2 TLR2 TLR5
engulfment of cells	2.07E-05	2.624	10	CEBPB CH25H CSF3 HP HYOU1 IL1B PTX3 SOAT1 TGM2 TLR2
cell death of lymphoid organ	7.68E-05	2.633	6	ADAM8 C5AR1 IL1B RIPK3 TGM2 TLR2
activation of antigen presenting cells	9.86E-05	2.634	8	ANGPT2 CEBPB CSF3 HSP90B1 IL1B MMP9 TGM2 TLR2
apoptosis of macrophages	1.92E-07	2.639	7	ADAM8 CEBPB IL1B LDLR RIPK3 SOAT1 TLR2
cellular infiltration by granulocytes	2.02E-09	2.662	11	ADAM8 ANGPT2 C5AR1 CSF3 IL1B LTB4R MMP9 PTX3 TGM2 TLR2 TNFRSF9
growth of tumor	1.00E-04	2.668	13	ANGPT2 ARHGEF4 CSF3 HYOU1 IL1B KDR MME MMP9 PFKFB3 TGM2 TIMP2 TLR2 TNFRSF9
recruitment of phagocytes	1.21E-06	2.672	9	ANGPT2 C5AR1 IL1B KDR LTB4R MMP9 PTX3 TLR2 TLR5
vascularization of body region	1.30E-05	2.674	6	C5AR1 CSF3 IL1B KDR MMP9 TIMP2
differentiation of blood cells	5.76E-04	2.686	12	ANGPT2 C5AR1 CEBPB CSF3 HSP90B1 IL1B KDR MMP9 RIPK3 TLR2 TLR5 TNFRSF9
cellular infiltration by leukocytes	3.39E-09	2.694	14	ADAM8 ANGPT2 C5AR1 CSF3 HYOU1 IL1B KDR LDLR LTB4R MMP9 PTX3 TGM2 TLR2 TNFRSF9
migration of cells	3.12E-06	2.699	25	ADAM8 ANGPT2 ARHGEF4 C5AR1 CEBPB CSF3 HP HSP90B1 HYOU1 IGFBP6 IL1B KDR LDLR LTB4R MME MMP9 PTAFR PTX3 RHO S1PR4 TGM2 TIMP2 TLR2 TLR5 TNFRSF9
metastasis	7.06E-05	2.732	13	ANGPT2 C5AR1 CSF3 HCAR1 HSP90B1 HYOU1 IL1B KDR MME MMP9 PTX3 TGM2 TLR2
advanced malignant tumor	1.16E-05	2.732	15	ANGPT2 C5AR1 CSF3 HCAR1 HSP90B1 HYOU1 IL1B KDR LPAR5 MME MMP9 PTAFR PTX3 TGM2 TLR2
chemotaxis of myeloid cells	2.97E-04	2.747	7	ANGPT2 C5AR1 HP IL1B LDLR LTB4R TLR2
stimulation of cells	1.47E-05	2.756	9	ANGPT2 C5AR1 CSF3 GCK HSP90B1 IL1B TLR2 TLR5 TNFRSF9
synthesis of lipid	3.43E-10	2.757	20	ACSS1 ANGPT2 C5AR1 CEBPB CH25H CYP24A1 DOLK GGT1 HSD17B1 IL1B IL1R2 KDR LDLR LTB4R MMP9 PTAFR PTX3 SOAT1 THRSP TLR2
metabolism of sterol	4.46E-06	2.788	6	APOF CH25H CYP24A1 LDLR SOAT1 SQLE
permeability of vascular system	5.02E-06	2.821	7	ANGPT2 IL1B KDR LDLR MMP9 PTX3 TIMP2
production of reactive oxygen species	1.75E-04	2.825	9	ANGPT2 C5AR1 CSF3 GGT1 IL1B RIPK3 TGM2 TLR2 TLR5
cell death of immune cells	6.97E-05	2.826	12	ADAM8 CEBPB CSF3 GGT1 HSH2D IL1B LDLR RIPK3 SOAT1 TGM2 TLR2 TNFRSF9
activation of myeloid cells	4.92E-06	2.838	10	ANGPT2 C5AR1 CEBPB CSF3 IL1B LTB4R MMP9 TGM2 TLR2 TNFRSF9
cell movement of myeloid cells	4.53E-08	2.843	15	ADAM8 ANGPT2 C5AR1 CSF3 HP IL1B KDR LDLR LTB4R MMP9 PTX3 TGM2 TIMP2 TLR2 TNFRSF9
angiogenesis	5.04E-08	2.85	19	ADAM8 ANGPT2 ARHGEF4 BCL6B C5AR1 CSF3 HP IL1B KDR LTB4R MMP9 PFKFB3 PTAFR PTX3 RGS5 S1PR4 TGM2 TIMP2 TLR2
vasculogenesis	1.14E-05	2.853	14	ANGPT2 BCL6B C5AR1 CSF3 IL1B KDR LTB4R MMP9 PFKFB3 PTAFR PTX3 RGS5 TGM2 TIMP2
inflammation	4.78E-09	2.881	19	ADAM8 ANGPT2 C5AR1 CEBPB CSF3 GGT1 HP IL1B KDR LDLR LTB4R MMP9 PTAFR PTX3 RIPK3 TGM2 TLR2 TLR5 TNFRSF9
recruitment of macrophages	1.53E-07	2.89	7	ANGPT2 C5AR1 IL1B KDR LTB4R MMP9 TLR2
cell death of lymphocytes	3.77E-04	2.89	8	ADAM8 CSF3 HSH2D IL1B RIPK3 TGM2 TLR2 TNFRSF9
adhesion of immune cells	2.15E-04	2.914	8	ANGPT2 IL1B LTB4R OCSTAMP TGM2 TLR2 TLR5 TNFRSF9
quantity of antigen presenting cells	2.24E-07	2.97	10	ADAM8 C5AR1 CSF3 IL1B LDLR MMP9 SOAT1 STEAP4 TGM2 TLR2
leukocyte migration	3.86E-08	3.006	19	ADAM8 ANGPT2 C5AR1 CSF3 HP HYOU1 IL1B KDR LDLR LTB4R MMP9 PTAFR PTX3 S1PR4 TGM2 TIMP2 TLR2 TLR5 TNFRSF9
activation of cells	1.33E-06	3.017	18	ADAM8 ANGPT2 C5AR1 CEBPB CSF3 GGT1 HSH2D HSP90B1 HYOU1 IL1B KDR LDLR LTB4R MMP9 TGM2 TLR2 TLR5 TNFRSF9
differentiation of cells	1.45E-04	3.022	24	ADAM8 ANGPT2 ATP2A2 C5AR1 CEBPB CREB5 CSF3 HSP90B1 IGFBP6 IL1B KDR LDLR MME MMP9 OCSTAMP RIPK3 S1PR4 SALL1 TGM2 TIMP2 TLR2 TLR5 TNFRSF9 TYRP1
expansion of cells	3.11E-04	3.037	7	C5AR1 CEBPB CSF3 IL1B KDR TLR2 TNFRSF9
cell viability of blood cells	1.90E-04	3.039	7	CEBPB CSF3 HSH2D IL1B KDR TGM2 TNFRSF9
cell viability of leukocytes	6.67E-04	3.043	6	CEBPB CSF3 HSH2D IL1B TGM2 TNFRSF9
chemotaxis of leukocytes	4.33E-06	3.082	10	ADAM8 ANGPT2 C5AR1 HP IL1B KDR LDLR LTB4R MMP9 TLR2
recruitment of antigen presenting cells	2.23E-08	3.121	8	ANGPT2 C5AR1 IL1B KDR LTB4R MMP9 TLR2 TLR5
T cell development	7.14E-04	3.151	9	C5AR1 CEBPB HSP90B1 IL1B KDR RIPK3 TLR2 TLR5 TNFRSF9
cell proliferation	6.95E-07	3.15	40	ADAM8 ANGPT2 ANKRD1 ARHGEF4 ATP2A2 BCL6B C5AR1 CCDC169 CEBPB CH25H CREB5 CSF3 CYP24A1 DENND4A DNAH7 F13B F8 GCK GGT1 GNAT1 HP HSD17B1 HSP90B1 HYOU1 IGFBP6 IL1B IL1R2 KDR LDLR LPAR5 MME MMP9 SLC11A2 SOAT1 TGM2 TIMP2 TLR2 TLR5 TNFRSF9
glucose tolerance	1.42E-05	3.178	8	ATP2A2 CREB5 GCK HYOU1 LDLR STEAP4 TGM2 TLR2
activation of phagocytes	1.65E-07	3.232	12	ANGPT2 C5AR1 CEBPB CSF3 HSP90B1 IL1B LTB4R MMP9 TGM2 TLR2 TLR5 TNFRSF9
apoptosis of lymphoid organ	4.25E-04	3.236	5	ADAM8 C5AR1 IL1B RIPK3 TLR2
steroid metabolism	1.73E-06	3.274	9	APOF CH25H CYP24A1 HSD17B1 IL1B LDLR MMP9 SOAT1 SQLE
migration of tumor cell lines	1.16E-04	3.331	12	ANGPT2 ARHGEF4 CEBPB CSF3 IGFBP6 IL1B KDR MME MMP9 TGM2 TIMP2 TLR2
response of mononuclear leukocytes	5.87E-05	3.39	7	C5AR1 CH25H HSP90B1 IL1B PTAFR TLR2 TNFRSF9
activation of leukocytes	1.19E-06	3.391	15	ANGPT2 C5AR1 CEBPB CSF3 HSH2D HSP90B1 HYOU1 IL1B LDLR LTB4R MMP9 TGM2 TLR2 TLR5 TNFRSF9
homing of cells	1.47E-05	3.403	12	ADAM8 ANGPT2 C5AR1 CSF3 HP IL1B KDR LDLR LTB4R MMP9 PTAFR TLR2
growth of embryos	9.93E-04	3.433	6	CSF3 KDR MMP9 S1PR4 SALL1 TIMP2
development of leukocytes	3.78E-05	3.557	12	C5AR1 CEBPB CSF3 HSP90B1 IL1B KDR LDLR RIPK3 SOAT1 TLR2 TLR5 TNFRSF9
chemotaxis of cells	3.44E-05	3.568	11	ADAM8 ANGPT2 C5AR1 HP IL1B KDR LDLR LTB4R MMP9 PTAFR TLR2
metabolism of membrane lipid derivative	1.09E-05	3.668	10	APOF CH25H CYP24A1 IL1B KDR LDLR PTAFR PTX3 SOAT1 SQLE
development of blood cells	5.65E-06	3.731	14	ANGPT2 C5AR1 CEBPB CSF3 HSP90B1 IL1B KDR LDLR RIPK3 SLC11A2 SOAT1 TLR2 TLR5 TNFRSF9

SLC11A2 SOAT1 SPSB4 SQLE STEAP4 TGM2 THRSP TIMP2 TLR2 TNFAIP2 TNFRSF9 TYRP1 VEPH1 VWA5A XIRP2

TNFRSF9 TYRP1

TYRP1
