# Supplementary material for: Waveband specific transcriptional control of select genetic pathways in vertebrate skin (Xiphophorus maculatus)
Source: BMC Genomics. 2018 May 10;19:355. doi: 10.1186/s12864-018-4735-5 (PMC5946439; doi:10.1186/s12864-018-4735-5)
Supplement: Supplementary file 2 — Table S2a–k. A list of all differentially modulated genes used by IPA enrichment software to predict the direction of change for each functional class represented in Additional file 1: Table S1. Table a is FL, tables b–e are the 50 nm wavebands and tables g–k are the 10 nm wavebands. (ZIP 701 kb) [file 12864_2018_4735_MOESM2_ESM.zip › TableS2c_400-450nm.pdf]

Functional Class	p-Value	Activation	# Genes	Genes				
cell death	9.75E-04	2.12	5	HMOX1	KCNJ12	NR4A3	PPARGC1A	YBX2
cell proliferation	8.23E-04	2.00	5	HMOX1	KCNJ12	NR4A3	PPARGC1A	YBX2
cell viability	9.20E-04	2.00	5	HMOX1	KCNJ12	NR4A3	PPARGC1A	YBX2
