# Supplementary material for: Waveband specific transcriptional control of select genetic pathways in vertebrate skin (Xiphophorus maculatus)
Source: BMC Genomics. 2018 May 10;19:355. doi: 10.1186/s12864-018-4735-5 (PMC5946439; doi:10.1186/s12864-018-4735-5)
Supplement: Supplementary file 2 — Table S2a–k. A list of all differentially modulated genes used by IPA enrichment software to predict the direction of change for each functional class represented in Additional file 1: Table S1. Table a is FL, tables b–e are the 50 nm wavebands and tables g–k are the 10 nm wavebands. (ZIP 701 kb) [file 12864_2018_4735_MOESM2_ESM.zip › TableS2b_350-400nm.pdf]

Functional Class	p-Value	Activation :	# Genes	Genes						
apoptosis of muscle cells	3.75E-03	-3.21	6	CAPN1	CXCL12	HMOX1	MPO	NR4A3	PPARGC1A	
inflammation of organ	1.65E-03	-3.05	17	ANXA1	ATM	BHLHE40	CA1	CA13	CA2	CA3
size of lesion	3.30E-03	-2.82	6	ANXA1	BMPR1B	CAPN1	CXCL12	HMOX1	MPO	
necrosis	1.24E-04	-2.78	33	ANXA1	ATM	BMPR1B	CDC34	CTBP2	CXCL12	DNAJB1
organismal death	8.02E-04	-2.67	37	ANXA1	ARNTL	ATM	BMPR1B	CDC34	CTBP2	CXCL12
check point control	8.00E-04	-2.58	31	ALPK3	ANXA1	ARNTL	ATM	BMPR1B	CAMK1G	CTBP2
cell death	4.43E-03	-2.50	8	ATM	CA3	HMOX1	ITPR1	MLKL	RAB25	SLC9A3R2
differentiation of connective tissue	6.60E-03	-2.40	13	ATM	BMPR1B	CA3	CTBP2	FAAH	GALNT3	GNPNAT1
weight loss	4.19E-04	-2.40	7	ARNTL	CLOCK	CYP1A1	HMOX1	NR4A3	PNRC2	PPARGC1A
inflammation	9.15E-04	-2.35	24	ANXA1	ARNTL	ATM	ATP1B2	CLIC3	CXCL12	DENND3
behavior	1.85E-05	-2.25	19	ARNTL	ATM	ATP1B2	BMPR1B	CAPN1	CLOCK	CYP1A2
dna repair	6.12E-03	-2.11	16	ARNTL	BMPR1B	CTBP2	ETV7	EZR	HOXC13	IRF1
quantity of protein in blood	1.17E-03	-2.11	10	ARNTL	ATM	CYP1A1	GALNT3	HMOX1	PER1	PER2
cell viability	5.22E-03	-2.02	22	ANXA1	ATM	BMPR1B	CTBP2	CXCL12	CYP1A1	ELOVL7
apoptosis	5.22E-03	2.05	16	ANXA1	ATM	BMPR1B	CDC34	CXCL12	EZR	GALNT3
cell death of myeloid cells	1.21E-03	2.06	6	ANXA1	CXCL12	HMOX1	MPO	PDGFC	TPSAB1/TPSB2	
transport of molecule	7.86E-04	2.19	22	ANXA1	ARNTL	ATM	ATP1B2	BHLHE40	CA2	CLIC3
cell death of phagocytes	1.69E-04	2.37	7	ANXA1	CXCL12	HMOX1	MPO	NR4A3	PDGFC	TPSAB1/TP
ingestion	1.31E-05	2.39	8	ARNTL	ATM	ATP1B2	HOMER2	NPPC	NR4A3	PER1
oxidation of lipid	1.21E-03	3.17	6	CYP1A1	CYP1A2	HACL1	LPIN1	NR4A3	PPARGC1A	
cell death of fibroblast cell lines	3.03E-03	3.19	11	ANXA1	ATM	CLOCK	CXCL12	EZR	HMOX1	ITPR1
differentiation of connective tissue	1.69E-03	3.21	8	ARNTL	BMPR1B	CLEC3B	CYP1A1	CYP1A2	LPIN1	PER1
engulfment of tumor cell lines	1.02E-03	3.22	5	ANXA1	CLIC3	CXCL12	EZR	HMOX1		
apoptosis of leukocytes	1.93E-03	3.43	9	ANXA1	ATM	CXCL12	EZR	ITPR1	MPO	NR4A3

	CXCL12	FAAH	HMOX1	LPIN1	MPO	PER1	PPARGC1A	TGM3	TGM6	TMEM173			
	DPM3	DSG2	EMP1	EZR	GALNT3	GALNT5	GNPNAT1	IDH2	IRF1	ITPR1	MAP2K6	MLKL	NCEH1
	DNAJB1	DPM3	DSG2	EMP1	EZR	GALNT3	GALNT5	GNPNAT1	HUNK	IDH2	IRF1	ITPR1	MAP2K6
	CXCL12	EMP1	ETV7	EVPL	EZR	GNA15	HUNK	IDH2	IRF1	ITPR1	MAP2K6	MYO5B	NPPC
	TGM1												
	HMOX1	ITPR1	MLKL	RAB25	SLC9A3R2	TGM1							
PPARGC1A													
	EZR	ITPR1	MAP2K6	MYO5B	NCEH1	NIPA2	NPPC	NR4A3	OSBPL2	PAX6	PDYN	PER2	PPARGC1A
	FAAH	HMOX1	HOMER2	MYH14	NR4A3	PER1	PER2	PER3	PNRC2	PPARGC1A	PRSS12	TGM1	
	NR4A3	PAX6	PER2	PPARGC1A	SAMD11	SOX1	SOX2	TMEM173	WNT5A				
	PNRC2	PPARGC1A	TMEM173										
	EMP1	ETV7	EZR	FOXQ1	GNA15	IDH2	IRF1	ITPR1	LRRN1	MAP2K6	MYH14	PDGFC	SOX2
	IRF1	ITPR1	MAP2K6	NCEH1	NR4A3	PDGFC	PPARGC1A	STAP2	WNT5A				
	CXCL12	DENND3	EZR	HMOX1	ITPR1	MPO	MYO5B	NPPC	NR4A3	PER1	PER2	PPARGC1A	RHBG
TPSAB1/TPSB2													
	PER2												
	MPO	NR4A3	PDGFC	TPSAB1/TPSB2									
	PPARGC1A												
	PDGFC	TPSAB1/TPSB2											

NR4A3	PAX6	PDGFC	PLEKHF1	PPARGC1/	RAB25	SIGIRR	SLC9A3R2	SOX2	STAP2	TGM1	TMEM173	WNT5A
MLKL	NCEH1	NR4A3	PAX6	PDGFC	PDYN	PLEKHF1	PPARGC1/	RAB25	SEC23B	SIGIRR	SLC9A3R2	SOX2
NR4A3	PAX6	PPARGC1/	PRSS12	RSAD2	SCEL	SOX1	SOX2	SOX3	TGM1	WNT5A		

RANGAP1	RSAD2	SEC23B	SLC9A3R2

STAP2	WNT5A

SLC9A3R2	TPSD1

STAP2	TGM1	TMEM173	WNT5A
