# Supplementary material for: Waveband specific transcriptional control of select genetic pathways in vertebrate skin (Xiphophorus maculatus)
Source: BMC Genomics. 2018 May 10;19:355. doi: 10.1186/s12864-018-4735-5 (PMC5946439; doi:10.1186/s12864-018-4735-5)
Supplement: Supplementary file 2 — Table S2a–k. A list of all differentially modulated genes used by IPA enrichment software to predict the direction of change for each functional class represented in Additional file 1: Table S1. Table a is FL, tables b–e are the 50 nm wavebands and tables g–k are the 10 nm wavebands. (ZIP 701 kb) [file 12864_2018_4735_MOESM2_ESM.zip › TableS2a_FL.pdf]

Diseases or Functions Annotation	p-Value	Activation	# Molecule	Molecules																	
cytokinesis	4.70E-16	-2.69	29	ANLN	AURKA	AURKB	BRCA2	CCNB1	CDC20	CDC25B	CDC6	CENPJ	CEP55	CIT	DIAPH3	ECT2	INCENP	KIF14	KIF20A	KIF20B	KIF23
cytokinesis of tumor cell lines	1.58E-12	-2.07	15	ANLN	AURKB	CEP55	DIAPH3	ECT2	KIF14	KIF20A	KIF20B	KIF23	KIFC1	L3MBTL1	MASTL	NPM1	RACGAP1	TOP2A			
M phase of tumor cell lines	8.47E-22	-2.63	26	ANLN	AURKB	CDC25B	CDT1	CEP55	DIAPH3	ECT2	FBXO5	KIF14	KIF20A	KIF20B	KIF23	KIFC1	L3MBTL1	MAD2L1	MASTL	NCAPD2	NDC80
M phase	1.22E-29	-2.52	47	ANLN	ATM	AURKA	AURKB	BRCA2	BUB1B	CCNB1	CDC20	CDC25B	CDC6	CDK1	CDT1	CENPE	CENPJ	CEP55	CIT	CKS2	DIAPH3
S phase	1.44E-08	-2.06	16	ATM	BHLHE40	CCNA2	CDK1	E2F1	E2F8	FOXM1	HMOX1	MCM10	MCM3	MYBL2	MYC	PLK1	POLA1	RPA1	TYMS		
G2/M phase	3.32E-10	-2.00	26	ATM	AURKA	BRCA1	CCNA2	CCNB1	CDC25B	CDK1	CENPJ	CIT	CLSPN	DTL	FANCD2	FOXM1	HMGA1	MAD2L1	MAP2K6	MASTL	MCM10
inflammation	7.23E-08	2.02	8	DHFR	FBXO32	POLE	POLE2	RRM2	SLC2A4	TOP2A	TYMS										
aneuploidy	2.78E-06	2.52	10	ATM	AURKA	BRCA1	BUB1	BUB1B	CDC20	CENPE	ESPL1	MAD2L1	PTTG1								
cellular recombination	2.35E-10	-3.06	16	ATM	BRCA1	BRCA2	EXO1	FANCD2	MCM8	MMS22L	PALB2	PARPBP	RAD51	RAD52	RAD54B	RECQL4	RFC3	RPA2	TONSL		
homologous recombination	1.58E-10	-2.97	18	ATM	BRCA1	BRCA2	FANCD2	MCM8	MMS22L	PALB2	PARPBP	PRKDC	RAD51	RAD52	RAD54B	RAD54L	RECQL4	RFC3	RPA2	TONSL	TRIP13
homologous recombination of cells	3.94E-08	-2.90	13	ATM	BRCA1	BRCA2	FANCD2	MCM8	MMS22L	PALB2	PARPBP	RAD51	RECQL4	RFC3	RPA2	TONSL					
homologous recombination of DNA	1.44E-08	-2.40	8	BRCA1	BRCA2	MMS22L	PRKDC	RAD51	RAD52	RAD54L	TONSL										
checkpoint control	1.37E-16	-2.29	22	ATM	BRCA1	BUB1	BUB1B	CCNB1	CCNB2	CDC20	CDC6	CKS2	E2F1	EXO1	FANCD2	KNTC1	MAD2L1	MAD2L1BP	MDC1	MTBP	MYC
cell survival	1.29E-11	-5.24	82	AGTPBP1	AIMP1	ALS2	ANLN	ASCC3	ASNS	ATM	AURKA	AURKB	BARD1	BHLHE40	BRCA1	BRCA2	BRIP1	BUB1B	CCNA2	CCNB1	CDK1
cell viability	1.99E-11	-5.08	78	AGTPBP1	AIMP1	ALS2	ANLN	ASCC3	ASNS	ATM	AURKA	AURKB	BARD1	BHLHE40	BRCA1	BRCA2	BRIP1	BUB1B	CCNA2	CCNB1	CDK1
cell viability of tumor cell lines	2.16E-12	-4.69	58	AIMP1	ALS2	ANLN	ASCC3	ASNS	ATM	AURKA	AURKB	BARD1	BHLHE40	BRCA1	BRCA2	BRIP1	BUB1B	CCNA2	CCNB1	CENPA	CKAP5
cell viability of cervical cancer cell lines	5.07E-05	-3.39	16	ATM	AURKA	AURKB	BRCA1	BRCA2	BUB1B	CCNA2	CENPA	MMS22L	PRKDC	RAD51	RAD52	RPA1	SKA1	TONSL	XRCC4		
cell death of cervical cancer cell lines	1.19E-12	2.32	36	AURKB	BRCA1	BUB1	BUB1B	CASC5	CCNB1	CDC20	CDC6	CDK1	CENPE	CKAP5	E2F1	HAUS8	KIF14	MAD2L1	MAN2C1	MAP2K6	MCM10
apoptosis of germ cells	1.32E-05	2.62	13	ATM	BRCA1	CHTF18	DNMT1	EXO1	HSP90AA1	KIF18A	MCM8	MYC	PALB2	PRKDC	TSC22D3	YBX2					
apoptosis of cervical cancer cell lines	5.50E-10	2.79	28	BRCA1	BUB1	CASC5	CCNB1	CDC20	CDC6	CDK1	CENPE	CKAP5	E2F1	KIF14	MAN2C1	MCM10	MSTN	MYC	NASP	NDC80	NUF2
apoptosis of epithelial cells	6.89E-15	3.46	83	AIMP1	ALS2	ASNS	ATAD2	ATM	AURKA	BARD1	BHLHE40	BRCA1	BRCA2	BUB1	CASC5	CCNB1	CDC20	CDC6	CDK1	CENPE	CKAP5
cell death of epithelial cells	1.83E-20	3.68	108	AIMP1	ALS2	ASNS	ATAD2	ATM	AURKA	AURKB	BARD1	BHLHE40	BRCA1	BRCA2	BUB1	BUB1B	CABLES1	CASC5	CCNB1	CDC20	CDC6
necrosis	5.46E-16	4.06	108	AIMP1	ALS2	ASNS	ATAD2	ATM	AURKA	AURKB	BARD1	BHLHE40	BRCA1	BRCA2	BUB1	BUB1B	CABLES1	CASC5	CCNB1	CDC20	CDC6
apoptosis	7.28E-14	4.15	135	ADRB2	AIMP1	ALS2	ASNS	ATAD2	ATM	AURKA	AURKB	BARD1	BHLHE40	BRCA1	BRCA2	BUB1	CABLES1	CASC5	CCNA2	CCNB1	CDC20
cell death	6.40E-18	5.03	172	ABCC5	ADRB2	AGTPBP1	AIMP1	ALS2	ARNTL	ASNS	ATAD2	ATM	AURKA	AURKB	BARD1	BHLHE40	BRCA1	BRCA2	BUB1	BUB1B	C7
repair of cells	2.55E-07	-2.65	12	ATM	BRCA1	BRCA2	KPNA2	MCM8	NPM1	PALB2	PCNA	PRKDC	RAD51	RAD52	RPA1						
organization of cytoskeleton	6.60E-08	-3.25	73	AGTPBP1	ALS2	ASPM	AURKA	AURKB	BCAS3	BORA	BRCA1	CCNB1	CDC20	CDC25B	CDK1	CELSR2	CENPE	CENPF	CENPJ	CEP192	CEP70
organization of cytoplasm	3.29E-09	-3.25	82	AGTPBP1	ALS2	ASPM	AURKA	AURKB	BCAS3	BORA	BRCA1	CCNB1	CDC20	CDC25B	CDK1	CELSR2	CENPE	CENPF	CENPJ	CEP192	CEP70
microtubule arrangement	6.31E-06	-2.83	59	AGTPBP1	ALS2	AURKA	BCAS3	BRCA1	CDC20	CDC25B	CDK1	CELSR2	CENPE	CENPF	CENPJ	CEP192	CEP70	CIT	CKAP5	CYR61	DACT1
association of chromatin	1.39E-11	-2.78	9	BRCA1	CDC6	CDT1	HELLS	MCM4	RPA1	RPA2	SUV39H1	WDHD1									
association of chromosome components	6.07E-11	-2.16	10	ATM	BRCA1	CDC6	CDT1	HELLS	MCM4	RPA1	RPA2	SUV39H1	WDHD1								
chromosomal congression of chromosomes	1.40E-18	-2.54	11	AURKB	CENPE	KIF14	KIF18A	KIF22	KIF2C	KIFC1	MAD2L1	NDC80	NET1	PLK1							
chromosomal alignment	8.77E-23	-2.49	17	AURKA	CCNA2	CENPE	DLGAP5	KIF14	KIF18A	KIF22	KIF2C	KIFC1	MIS12	NCAPD2	NCAPG	NCAPG2	PLK1	SGO1	SMC4	TTK	
lymphoproliferative malignancy	5.37E-07	2.06	118	ACAD11	ADRB2	AGL	AIMP1	ALS2	ANLN	ASCC3	ASPM	ATM	AURKA	AURKB	BARD1	BDH2	BRCA1	BRCA2	BUB1B	C7	CASC5
repair of DNA	3.60E-23	-3.34	45	ASCC3	ATM	BRCA1	BRCA2	BRIP1	CDK1	E2F1	EXO1	FANCD2	FANCI	FANCL	FIGNL1	HMGA1	HMOX1	KPNA2	LIG1	MSH6	MUTYH
metabolism of DNA	4.85E-17	-3.23	44	ATM	BARD1	BRCA1	BRCA2	BRIP1	CCNA2	CDC6	CDK1	CDT1	CKS2	E2F1	EXO1	FOXM1	HMGA1	KPNA2	LIG1	MCM2	MCM3
DNA replication	2.76E-20	-2.66	38	ATM	BRCA1	BRCA2	BRIP1	CCNA2	CDC6	CDK1	CDT1	CKS2	E2F1	FOXM1	HMGA1	LIG1	MCM2	MCM3	MCM8	MYC	NASP
fatty acid oxidation	2.04E-08	2.32	14	AMT	ASNS	BHMT	GGT5	HGD	HPD	MTR	MYC	OAT	PAH	PHGDH	SHMT1	SHMT2	SMS				
chromosomal aberration	1.22E-05	2.78	12	ATM	BRCA1	BRCA2	BUB1B	EXO1	FANCD2	LIG1	MAD2L1	MYC	PALB2	POLA1	TYMS						
chromosomal instability	9.69E-07	2.79	8	ATM	BRCA1	BRCA2	EXO1	FANCD2	LIG1	MAD2L1	PALB2										
breakage of chromosomes	2.90E-10	3.12	12	ATM	BRCA1	BRIP1	DNMT3B	MAD2L1	MCM8	MYC	NPM1	PALB2	PTTG1	RECQL4	XRCC4						
proliferation of cells	2.29E-15	-5.98	176	ABCC5	ADRB2	AIMP1	ALS2	ARNTL	ASCC3	ASNS	ASPM	ATAD2	ATM	AURKA	AURKB	BARD1	BHLHE40	BHMT	BRCA1	BRCA2	BRIP1
cell proliferation of tumor cell lines	1.55E-07	-4.39	79	ASCC3	ASNS	ATAD2	ATM	AURKA	AURKB	BARD1	BRCA1	BRCA2	BUB1	CABLES1	CCNA2	CCNB1	CCNB2	CDC25B	CDCA8	CDK1	CDT1
cell proliferation of breast cancer cell lines	9.30E-06	-2.97	28	ATAD2	ATM	BRCA1	BRCA2	BUB1	CDC25B	CDK1	CYR61	DLGAP5	DNMT3B	E2F1	FOSL1	FOXM1	HMGA1	KLF5	KPNA2	MAP2K6	MELK
proliferation of connective tissue cells	2.15E-06	-3.57	36	AIMP1	ATM	BRCA1	BRCA2	BUB1	CCNA2	CCNB1	CCNF	CDC6	CYR61	E2F1	ESPL1	FANCD2	FIGNL1	FOSL1	HMMR	HMOX1	KLF5
cell proliferation of fibroblasts	4.99E-08	-2.56	28	AIMP1	ATM	BRCA1	BRCA2	BUB1	CCNA2	CCNB1	CCNF	CDC6	E2F1	ESPL1	FANCD2	FOSL1	HMMR	HMOX1	LIG1	MAP2K6	MCM8
growth of connective tissue	2.53E-06	-3.38	38	AIMP1	ATM	BRCA1	BRCA2	BUB1	CCNA2	CCNB1	CCNF	CDC6	CYR61	E2F1	ESPL1	FANCD2	FIGNL1	FOSL1	HMGA1	HMMR	HMOX1
lipid oxidation	5.15E-05	2.11	26	ACRC	ADM2	BCAS3	BUB1B	CEP192	DHFR	E2F1	EPHB6	FOSL1	GSTT1	HPX	HSP90AA1	KCNK2	KIF20B	KIF23	MEGF6	MKI67	ODC1
DNA damage	5.05E-12	2.29	25	ATM	BRCA1	BRCA2	BRIP1	CDC6	CENPE	CHAF1A	DNMT1	DNMT3B	E2F1	FANCD2	LIG1	MCM10	MSH6	MUTYH	MYC	NPM1	PBK
T-cell lymphoproliferative disorder	1.83E-05	2.36	22	ATM	AURKA	BRCA1	BRCA2	CYR61	DHFR	E2F1	MKI67	MYC	ODC1	POLA1	POLD1	POLE	POLE2	PRKCB	PRKDC	RAD52	RPA1
neoplasia of leukocytes	1.20E-05	2.36	23	ATM	AURKA	BRCA1	BRCA2	CYR61	DDR1	DHFR	E2F1	MKI67	MYC	ODC1	POLA1	POLD1	POLE	POLE2	PRKCB	PRKDC	RAD52
T-cell non-Hodgkin's disease	1.52E-06	2.36	20	ATM	AURKA	BRCA1	BRCA2	CYR61	DHFR	E2F1	MKI67	MYC	ODC1	POLE	POLE2	PRKCB	PRKDC	RAD52	RPA1	RRM2	STMN1
non-Hodgkin's disease	3.41E-07	2.37	37	ATM	AURKA	AURKB	BRCA1	BRCA2	BUB1B	CCNA2	CCNF	CDC25B	CELSR2	CYR61	DHFR	DNMT3B	E2F1	HMGA1	MCM5	MKI67	MYC
lymphocytic cancer	9.57E-09	2.60	50	AIMP1	ATM	AURKA	AURKB	BRCA1	BRCA2	BUB1B	CCNA2	CCNF	CDC25B	CELSR2	CYR61	DCK	DHFR	DNMT3B	E2F1	ESPL1	EXO1
digestive tract cancer	3.27E-19	-2.41	353	ABCC5	ABHD14B	ABHD2	ACRC	ACSF3	ADAMTSL1	ADCY2	ADM2	AGL	AGTPBP1	AIMP1	AKR1D1	ALS2	AMT	ANKLE1	ANLN	ARHGAP11	ARHGAP19
colorectal neoplasia	1.27E-13	-2.17	233	ABCC5	ABHD14B	ABHD2	ADAMTSL1	ADCY2	AGL	AGTPBP1	AIMP1	ALS2	ARHGAP11	ASCC3	ASPM	ASTL	ATAD2	ATM	AURKA	BARD1	BCAS3
pancreatic tumor	9.09E-07	-2.09	170	ABCC5	ACRC	ACSF3	ADAMTSL1	ADCY2	AGTPBP1	AKR1D1	ALS2	AMT	ANLN	ARNTL	ASPM	ATAD2	ATM	AURKA	AURKB	BCAS3	BDH2
colorectal cancer	6.95E-13	-2.00	229	ABCC5	ABHD14B	ABHD2	ADAMTSL1	ADCY2	AGL	AGTPBP1	AIMP1	ALS2	ARHGAP11	ASCC3	ASPM	ASTL	ATAD2	ATM	AURKA	BARD1	BCAS3
malignant neoplasm of large intestine	2.77E-16	-2.00	306	ABCC5	ABHD14B	ABHD2	ACRC	ACSF3	ADAMTSL1	ADCY2	ADM2	AGL	AGTPBP1	AIMP1	ALS2	ANKLE1	ANLN	ARHGAP11	ARHGAP19	ARNTL	ASCC3
abdominal carcinoma	8.14E-10	2.00	265	ABCC5	ABHD14B	ABHD2	ACAD11	ACRC	ADAMTSL1	ADCY2	ADRB2	AGL	AGTPBP1	ALS2	AMT	ANLN	ARHGAP11	ARHGAP19	ARNTL	ASCC3	ASPM
formation of mitotic spindle	1.37E-16	2.04	19	AURKA	CEP192	CKAP5	FBXO5	HAUS1	HAUS2	HAUS3	HAUS5	HAUS7	HAUS8	HELLS	KIF2C	KIFC1	NUF2	NUMA1	PLK1	SASS6	STMN1
ocular tumor	9.55E-14	2.13	114	ABCC5	ADAMTSL1	ADCY2	ADRB2	ALS2	ANKLE1	ARHGAP19	ARNTL	ATM	AURKB	BARD1	BCAS3	BHLHE40	BRCA1	BRCA2	BRIP1	CCNA2	CCNB1
carcinoma in lung	1.13E-10	2.14	86	ABCC5	ADAMTSL1	ADCY2	ATM	AURKA	AURKB	BARD1	BCAS3	BRCA1	BRCA2	BUB1B	CASC5	CCNA2	CCNB1	CCNB2	CDC20	CDC6	CDCA8
tumorigenesis of reproductive cells	2.86E-13	2.18	182	ACAD11	ACRC	ADAMTSL1	ADCY2	ADRB2	AGL	AGTPBP1	ALS2	ANLN	ARHGAP11	ARHGAP19	ARNTL	ASCC3	ASPM	ATAD2	ATM	AURKA	AURKB
non-small cell lung cancer	3.43E-10	2.19	83	ABCC5	ADAMTSL1	ADCY2	ATM	AURKA	AURKB	BARD1	BCAS3	BRCA1	BRCA2	BUB1B	CASC5	CCNA2	CCNB1	CCNB2	CDC20	CDC6	CDCA8
non small cell lung adenocarcinoma	7.60E-09	2.20	67	ADAMTSL1	ADCY2	ATM	BCAS3	BRCA1	BRCA2	BUB1B	CASC5	CCNA2	CCNB1	CCNB2	CDC20	CDC6	CDK1	CENPF	CENPJ	CEP192	CHAF1A
incidence of lymphoma	6.56E-08	2.21	14	ATM	BRCA1	BRCA2	E2F1	EXO1	HMGA1	MSH6	MUTYH	MYC	PER2	PRKDC	RECQL4	UNG	XRCC4				
hematologic cancer of cells	1.76E-05	2.38	22	ATM	AURKA	BRCA1	BRCA2	CYR61	DHFR	E2F1	MKI67	MYC	ODC1	POLA1	POLD1	POLE	POLE2	PRKCB	PRKDC	RAD52	RPA1
incidence of malignant tumor	1.48E-08	2.89	20	ATM	AURKB	BHMT	BRCA1	BRCA2	CABLES1	E2F1	EXO1	FANCD2	HMGA1	MAD2L1	MSH6	MUTYH	MYC	PER2	PRKDC	RECQL4	UNG
adenocarcinoma	7.10E-17	2.94	342	ABCC5	ABHD14B	ABHD2	ACAD11	ACRC	ACSF3	ADAMTSL1	ADCY2	ADM2	ADRB2	AGL	AGTPBP1	ALS2	AMT	ANKLE1	ANLN	ARHGAP11	ARHGAP19
incidence of tumor	4.67E-11	2.96	31	ATM	AURKA	AURKB	BHMT	BRCA1	BRCA2	BUB1	CABLES1	CDCA7	E2F1	EXO1	FANCD2	HMGA1	HMMR	IL12B	MAD2L1	MCM8	MSH6
death of embryo	1.72E-12	3.43	22	AURKA	BRCA2	CDC20	CENPJ	CENPU	CHAF1A	DTL	ECT2	FASN	FBXO5	KIF22	KLF5	MCM10	METAP2	MIS18A	NASP	NR4A3	PCNA
organismal growth failure	9.66E-07	3.61	37	ARNTL	ATM	AURKA	BRCA1	BRCA2	CDC6	CDT1	CENPJ	DDR1	E2F8	EHHADH	ESPL1	FANCD2	GGT5	HAP1	HMOX1	HSD17B4	LIG1
organismal death	2.28E-14	7.87	128	ADRB2	AGL	ARNTL	ATM	AURKA	AURKB	BARD1	BRCA1	BRCA2	BRIP1	BUB1	BUB1B	CCNA2	CCNB2	CCNF	CDC20	CDC45	CDK1

KIFC1	L3MBTL1	MASTL	MYH9	NPM1	NUSAP1	PLK1	PRC1	RACGAP1	TOP2A	UBE2C														
NPM1	NUF2	PLK1	PTTG1	RACGAP1	SPDL1	TOP2A	TRIP13																	
ECT2	FBXO5	INCENP	KIF14	KIF20A	KIF20B	KIF23	KIFC1	L3MBTL1	MAD2L1	MASTL	MCM4	MCM8	MYH9	NCAPD2	NDC80	NPM1	NUF2	NUMA1	NUSAP1	PLK1	PRC1	PTTG1	RACGAP1	RAD51
MDC1	MELK	MYBL2	MYC	NPM1	PLK1	PTTG1	RPA1																	
NDC80	PLK1	PTTG1	ZWILCH																					
CENPA	CKAP5	CLOCK	CYR61	DCK	DNMT1	E2F1	EGR3	EXO1	FANCD2	FASN	FBXO32	FBXO5	FKBP5	FOSL1	FOXM1	HAP1	HMGA1	HMOX1	KIF18A	KIFC1	KLF5	KLF6	LIG1	MAD2L1
CENPA	CKAP5	CLOCK	CYR61	DCK	DNMT1	E2F1	EGR3	FANCD2	FASN	FBXO32	FBXO5	FKBP5	FOSL1	FOXM1	HAP1	HMGA1	HMOX1	KIF18A	KIFC1	KLF5	KLF6	LIG1	MAD2L1	MAP2K6
DCK	E2F1	FASN	FBXO5	FKBP5	FOSL1	FOXM1	HMGA1	HMOX1	KIF18A	KIFC1	KLF5	KLF6	LIG1	MAD2L1	MAP2K6	MMS22L	MYC	NDC80	NUF2	PBK	PCNA	PLK1	PRKCA	PRKDC
MMS22L	MSTN	MYC	NASP	NDC80	NUF2	PBK	PLK1	PLK4	PRKDC	RACGAP1	RPA1	SPAG5	TACC3	TOP2A	TPX2	TTK	UCP2							
PBK	PLK1	PLK4	PRKDC	RACGAP1	RPA1	SPAG5	TACC3	TPX2	TTK															
CYR61	DHCR24	DNMT1	DTYMK	E2F1	EGR3	EPHB6	FASN	FBXO32	FBXO5	FKBP5	FOSL1	GMDS	HAP1	HMGA1	HMMR	HMOX1	KIF14	KLF5	KLF6	KPNA2	LIG1	MAD2L1	MAN2C1	MAP2K6
CDK1	CDT1	CENPE	CENPI	CKAP5	CLSPN	CLYBL	CYR61	DCK	DCTD	DHCR24	DHFR	DNMT1	DTL	DTYMK	E2F1	EGR3	EPHB6	EYS	FANCL	FASN	FBXO32	FBXO5	FKBP5	FOSL1
CDK1	CDT1	CENPE	CENPI	CKAP5	CLSPN	CLYBL	CYR61	DCK	DCTD	DHCR24	DHFR	DNMT1	DTL	DTYMK	E2F1	EGR3	EPHB6	EYS	FANCL	FASN	FBXO32	FBXO5	FKBP5	FOSL1
CDC25B	CDC45	CDC6	CDK1	CENPA	CENPE	CENPF	CENPJ	CHTF18	CIT	CKAP5	CTNNBL1	CYR61	DCK	DDR1	DEPTOR	DHCR24	DHFR	DNMT1	DNMT3B	DTYMK	E2F1	E2F8	ECT2	EGR3
CABLES1	CASC5	CCNA2	CCNB1	CDC20	CDC25B	CDC45	CDC6	CDK1	CDT1	CENPA	CENPE	CENPF	CENPI	CENPJ	CHTF18	CIT	CKAP5	CLOCK	CLSPN	CLYBL	CTNNBL1	CYR61	DCK	DCTD
CIT	CKAP5	CYR61	DACT1	DDR1	DIAPH3	DLGAP5	ECT2	EGR3	EPHB6	ESPL1	FASN	FBXO5	FHL3	FOXM1	HAP1	HAUS8	HSP90AA1	KALRN	KIF18A	KIF20B	KIF23	KIF2C	KIFC1	KLF5
CIT	CKAP5	CYR61	DACT1	DDR1	DIAPH3	DLGAP5	ECT2	EGR3	EPHB6	ESPL1	FASN	FBXO5	FHL3	FOXM1	HAP1	HAUS1	HAUS2	HAUS3	HAUS5	HAUS7	HAUS8	HSP90AA1	KALRN	KIF18A
DDR1	DIAPH3	DLGAP5	ECT2	EGR3	EPHB6	FASN	FBXO5	FOXM1	HAP1	HAUS8	HSP90AA1	KALRN	KIF18A	KIF20B	KIF23	KIF2C	KIFC1	KLF5	MAD2L1	MID1IP1	MTBP	MYC	MYH9	NDC80
CCNA2	CCNF	CDC25B	CDK1	CELSR2	CENPE	CENPF	CENPI	CENPJ	CENPN	CEP192	CEP55	CKAP2L	COL11A1	CPT1A	CYR61	DACT1	DCHS2	DCK	DCTD	DDR1	DENND3	DHFR	DNAH7	DNMT1
NEIL3	NFE2L1	NPM1	PALB2	PCNA	POLA1	POLD1	POLE	POLE2	POLG	POLQ	PRKDC	RAD51	RAD52	RAD54B	RAD54L	RECQL4	RNASEH2A	RPA1	RPA2	SUV39H1	TOP2A	TRIP13	TYMS	UBE2T
MCM8	MYC	NASP	NPM1	NR4A3	ORC1	ORC2	ORC3	ORC5	PCNA	PLK1	POLA1	POLA2	POLD1	POLE	POLE2	POLG	RAD51	RECQL4	RFC3	RNASEH2A	RPA1	RPA2	SMARCAL1	TIMELESS
NPM1	NR4A3	ORC1	ORC2	ORC3	ORC5	PCNA	PLK1	POLA1	POLA2	POLD1	POLE	POLE2	POLG	RECQL4	RFC3	RNASEH2A	RPA1	RPA2	TIMELESS					
BUB1	BUB1B	CABLES1	CCNA2	CCNB1	CCNB2	CCNF	CDC20	CDC25B	CDC45	CDC6	CDCA7	CDCA8	CDK1	CDT1	CENPA	CENPF	CENPJ	CKS2	CLOCK	CLSPN	CLYBL	CYR61	DCK	DDR1
CENPA	CLYBL	CYR61	DLGAP5	DNMT3B	DTL	E2F1	E2F8	ECT2	EPHB6	FASN	FBXO32	FKBP5	FOSL1	FOXM1	HCAR1	HMGA1	HMMR	HMOX1	HSP90AA1	KCNK2	KIF20A	KIF2C	KLF5	KLF6
MYC	ODC1	PBK	PLK1	PRKCA	PRKCB	RFC3	TRAIP	TYMS	WWOX															
LIG1	MAD2L1	MAP2K6	MCM8	MKI67	MSTN	MYBL2	MYC	NPM1	PRKCA	PRKDC	PTTG1	RBL1	SMARCAL1	STMN1	TXNRD2	UBE2C	XRCC4							
MSTN	MYBL2	MYC	NPM1	PRKDC	PTTG1	RBL1	SMARCAL1	TXNRD2	XRCC4															
KLF5	LIG1	MAD2L1	MAP2K6	MCM8	MKI67	MSTN	MYBL2	MYC	MYH9	NPM1	PRKCA	PRKDC	PTTG1	RBL1	SMARCAL1	STMN1	TXNRD2	UBE2C	XRCC4					
PLK1	PRC1	PRKCB	RRM2	TICRR	TOP2A	TYMS	UBE2C																	
PLK1	PRKDC	RAD51	RAD52	RPA1	RRM2	TOP2A																		
RRM2	STMN1	TOP2A	TYMS																					
RPA1	RRM2	STMN1	TOP2A	TYMS																				
TOP2A	TYMS																							
ODC1	POLA1	POLD1	POLE	POLE2	PRKCB	PRKDC	RAD52	RAD54B	RAD54L	RPA1	RRM2	SHMT2	SLC29A2	STMN1	TOP2A	TYMS	UNG	XRCC4						
GSTT1	HMGA1	MCM5	MKI67	MSH6	MUTYH	MYC	NPM1	ODC1	PER2	POLA1	POLD1	POLE	POLE2	PRIM2	PRKCB	PRKDC	RAD52	RAD54B	RAD54L	RECQL4	RPA1	RRM2	SHMT2	SLC29A2
ARNTL	ASCC3	ASPM	ASTL	ATAD2	ATM	AURKA	AURKB	BARD1	BCAS3	BDH2	BHLHE40	BHMT	BNC2	BORA	BRCA1	BRCA2	BRIP1	BUB1	BUB1B	C7	CABLES1	CASC5	CCDC171	CCDC77
BHLHE40	BNC2	BRCA1	BRCA2	BRIP1	BUB1	BUB1B	CASC5	CCDC171	CCNA2	CCNB1	CCNB2	CCSAP	CDC45	CDC6	CDCA7	CDCA8	CDK1	CELSR2	CENPA	CENPF	CENPN	CENPP	CENPU	CEP55
BHLHE40	BHMT	BNC2	BRCA1	BUB1	BUB1B	C7	CASC5	CCNA2	CCNB1	CCNB2	CDC20	CDC25B	CDC45	CELSR2	CENPA	CENPE	CENPF	CENPI	CENPP	CENPU	CEP192	CHDH	CHTF18	CIART
BHLHE40	BNC2	BRCA1	BRCA2	BRIP1	BUB1	BUB1B	CASC5	CCDC171	CCNA2	CCNB2	CCSAP	CDC45	CDC6	CDCA7	CDCA8	CELSR2	CENPA	CENPF	CENPN	CENPP	CENPU	CEP55	CHAF1A	CHDH
ASPM	ASTL	ATAD2	ATM	AURKA	AURKB	BARD1	BCAS3	BHLHE40	BNC2	BORA	BRCA1	BRCA2	BRIP1	BUB1	BUB1B	C7	CABLES1	CASC5	CCDC171	CCDC77	CCNA2	CCNB1	CCNB2	CCNF
ASTL	ATAD2	ATM	AURKA	AURKB	BARD1	BCAS3	BHLHE40	BNC2	BRCA1	BRCA2	BRIP1	BUB1	BUB1B	C7	CABLES1	CASC5	CCDC171	CCNB3	CCSAP	CDC45	CDC6	CDCA7	CELSR2	CENPE
TPX2																								
CCNB2	CDC20	CDC25B	CDC6	CDCA7	CDK1	CDT1	CIT	CKAP5	CLOCK	COL11A1	CYR61	DDR1	DHFR	DNAH7	DNMT1	DNMT3B	E2F1	EGR3	EMP3	EPHB6	EXOC4	FANCD2	FASN	FBXO5
CDK1	CENPF	CENPJ	CEP192	CHAF1A	CKS2	CLOCK	COL11A1	CTNNBL1	DCK	DDR1	DHFR	DNAH7	DNMT1	E2F1	ECT2	EGR3	EHHADH	EMP3	EPHB6	EYS	FANCD2	FANCL	FASN	FGGY
BARD1	BNC2	BRCA1	BRCA2	BRIP1	BUB1	BUB1B	C7	CABLES1	CASC5	CCDC171	CCNA2	CCNB1	CCNB2	CCNB3	CDC20	CDC6	CDK1	CENPA	CENPE	CENPF	CENPI	CENPJ	CENPN	CEP55
CDK1	CENPF	CENPJ	CEP192	CHAF1A	CKS2	CLOCK	COL11A1	CTNNBL1	DCK	DDR1	DHFR	DNAH7	DNMT1	E2F1	ECT2	EGR3	EHHADH	EMP3	EPHB6	EYS	FANCD2	FASN	FGGY	FOCAD
CKS2	CLOCK	COL11A1	CTNNBL1	DDR1	DHFR	DNAH7	DNMT1	E2F1	ECT2	EGR3	EHHADH	EMP3	EPHB6	EYS	FANCD2	FGGY	FOCAD	FOXM1	GALNT14	H2AFV	HAUS5	HMGA1	HSD17B4	KALRN
RRM2	STMN1	TOP2A	TYMS																					
WWOX	XRCC4																							
ARNTL	ASCC3	ASPM	ASTL	ATAD2	ATM	AURKA	AURKB	BARD1	BCAS3	BHLHE40	BHMT	BNC2	BORA	BRCA1	BRCA2	BRIP1	BUB1	BUB1B	C7	CABLES1	CASC5	CCDC171	CCDC77	CCNA2
MUTYH	MYC	PER2	POLD1	PRKCA	PRKCB	PRKDC	PTGER1	RBL1	RECQL4	UNG	WWOX	XRCC4												
POLG	RAD51	TKT	TRAIP																					
MYC	NFE2L1	NR4A3	ORC1	PALB2	POLG	PTTG1	RBL1	RECQL4	SLC2A4	SLC4A1	SMARCAL1	TACC3	TKT	TNNT2	TRAIP	UHRF1	WWOX	XRCC4						
CENPE	CENPJ	CENPU	CHAF1A	CHTF18	CIT	CKAP5	CKS2	COL11A1	CPT1A	CYR61	DACT1	DDR1	DIAPH3	DNMT1	DNMT3B	DTL	E2F1	E2F8	ECT2	EGR3	EHHADH	ESPL1	EXO1	FANCD2

SPDL1	TOP2A	TRIP13	UBE2C

MAP2K6	METAP2	MMS22L	MYBL2	MYC	NDC80	NFIL3	NR4A3	NUF2	ODC1	PALB2	PBK	PCNA	PLK1	PRKCA	PRKCB	PRKDC	RAD51	RAD52	RAD54B	RAD54L	RPA1	RRM2	SHMT2	SKA1
METAP2	MMS22L	MYBL2	MYC	NDC80	NFIL3	NR4A3	NUF2	PALB2	PBK	PCNA	PLK1	PRKCA	PRKCB	PRKDC	RAD51	RAD52	RAD54B	RAD54L	RPA1	RRM2	SHMT2	SKA1	SLC2A4	STMN1
RAD51	RAD52	RAD54B	RAD54L	RPA1	RRM2	SKA1	SLC2A4	TGM2	TONSL	TTK	TYMS	UHRF1	WWOX	XRCC4										

MAT2A	MCM10	MDC1	MELK	MSTN	MTFP1	MYC	NASP	NDC80	NFE2L1	NFIL3	NPM1	NUF2	ODC1	PBK	PCNA	PER1	PHGDH	PKMYT1	PLK1	PLK4	PRKCA	PRKCB	PRKDC	PTTG1
GMDS	HAP1	HAUS8	HCAR1	HMGA1	HMMR	HMOX1	INCENP	KIF14	KLF5	KLF6	KPNA2	LIG1	MAD2L1	MAN2C1	MAP2K6	MAT2A	MCM10	MDC1	MELK	MMS22L	MSTN	MTFP1	MYBL2	MYC
GMDS	HAP1	HAUS8	HCAR1	HMGA1	HMMR	HMOX1	INCENP	KIF14	KLF5	KLF6	KPNA2	LIG1	MAD2L1	MAN2C1	MAP2K6	MAT2A	MCM10	MDC1	MELK	MMS22L	MSTN	MTFP1	MYBL2	MYC
EPHB6	ESPL1	EXO1	FASN	FBXO32	FBXO5	FIGNL1	FKBP5	FOSL1	G2E3	GMDS	HAP1	HAUS1	HELLS	HMGA1	HMMR	HMOX1	HSP90AA1	IL12B	KIF14	KIF18A	KLF5	KLF6	KPNA2	LIG1
DDR1	DEPTOR	DHCR24	DHFR	DNMT1	DNMT3B	DTL	DTYMK	E2F1	E2F8	ECT2	EGR3	EMP3	EPHB6	ESPL1	EXO1	EYS	FAAH	FANCD2	FANCL	FASN	FBXO32	FBXO5	FIGNL1	FKBP5

KNSTRN	LIG1	MAD2L1	MAP2K6	MID1IP1	MTBP	MYC	MYH9	NDC80	NET1	NFIX	NUMA1	NUSAP1	PHGDH	PIBF1	PLA2G10	PLK4	PRC1	PRKCA	PRKCB	PRKDC	RACGAP1	SPAG5	STK35	STMN1
KIF20B	KIF23	KIF2C	KIFC1	KLF5	KNSTRN	LIG1	MAD2L1	MAP2K6	MID1IP1	MTBP	MTFP1	MTFR2	MYC	MYH9	NDC80	NET1	NFIX	NUMA1	NUSAP1	PHGDH	PIBF1	PLA2G10	PLK1	PLK4
NET1	NFIX	NUMA1	NUSAP1	PHGDH	PIBF1	PLA2G10	PLK4	PRC1	PRKCA	PRKCB	RACGAP1	STMN1	TACC3	TNIK	TPX2									

DNMT3B	E2F1	E2F8	EFL1	ESPL1	EXO1	EYS	FANCL	FOXM1	GSTT1	HACL1	HELLS	HMGA1	KALRN	KIF18A	KIF20B	KLHL38	KPNA2	LRBA	MAN2C1	MAP2K6	MASTL	MCM5	MCM6	MELK
UNG	XRCC4																							
TOP2A																								

DEPTOR	DHCR24	DHFR	DLGAP5	DNMT1	DNMT3B	DTL	DTYMK	E2F1	E2F8	EBI3	ECT2	EGR3	EMP3	EPHB6	ESPL1	EXOC4	FAM83D	FANCD2	FANCL	FASN	FBXO32	FIGNL1	FKBP5	FOSL1
KPNA2	LIG1	MAP2K6	MCM2	MELK	MKI67	MSTN	MYC	NASP	NCAPG	NET1	NPM1	NUMA1	ODC1	PBK	PCNA	PLK1	PRKCA	PRKCB	PTTG1	RAB30	RAD51	RBL1	RFC3	RRM2

STMN1	TOP2A	TPX2	TYMS	UNG	WWOX	XRCC4																		
CCNA2	CCNB1	CCNB2	CCNB3	CCNF	CCSAP	CDC20	CDC25B	CDC45	CDC6	CDCA7	CDCA8	CDK1	CELSR2	CENPA	CENPE	CENPF	CENPI	CENPJ	CENPK	CENPN	CENPO	CENPP	CENPU	CEP192
CHAF1A	CHDH	CHTF18	CIT	CLOCK	CLYBL	COL11A1	CPT1A	CTNNBL1	DCHS2	DDR1	DENND3	DEPDC1B	DHFR	DIAPH3	DLGAP5	DNAH7	DNMT1	DNMT3B	E2F1	E2F8	EBI3	EGR3	EHHADH	ESPL1
CIT	CKAP5	CKS2	COL11A1	CYR61	DACT1	DCHS2	DCK	DENND3	DHCR24	DHFR	DIAPH3	DNAH7	E2F1	E2F8	ECT2	ELMOD3	ESPL1	EYS	FAM83D	FANCD2	FANCI	FASN	FBXL17	FOCAD
CHTF18	CIT	CLOCK	CLYBL	COL11A1	CPT1A	CTNNBL1	DCHS2	DDR1	DENND3	DEPDC1B	DHFR	DIAPH3	DLGAP5	DNAH7	DNMT1	E2F1	E2F8	EBI3	EGR3	EHHADH	ESPL1	EXO1	EYS	FAM83D
CCSAP	CDC25B	CDC45	CDC6	CDCA7	CDCA8	CDK1	CELSR2	CENPA	CENPE	CENPF	CENPK	CENPN	CENPP	CENPU	CEP55	CEP70	CHAF1A	CHDH	CHTF18	CIT	CKAP2L	CKAP5	CLOCK	CLSPN
CENPF	CENPI	CENPJ	CENPN	CENPP	CEP55	CEP70	CHAF1A	CHAF1B	CHDH	CHTF18	CIART	CIT	CKAP5	CLOCK	CLYBL	COL11A1	CPT1A	CTNNBL1	CYR61	DACT1	DCHS2	DDR1	DENND3	DEPDC1B

FGGY	FHL3	FKBP5	FOSL1	FOXM1	GINS4	HMMR	HMOX1	HSP90AA1	IL12B	KALRN	KCNK2	KIF22	KIF2C	KLF6	KPNA2	LPL	MCM10	MCM2	MCM4	MCM6	MELK	METAP2	MKI67	MSH6
FOCAD	FOXM1	GALNT14	H2AFV	HAUS5	HELLS	HMGA1	HSD17B4	HSP90AA1	KALRN	KIF23	KLF5	KNTC1	L3MBTL1	LIG1	MAD2L1	MCM2	MCM5	MKI67	MUTYH	MYBL2	MYC	MYH9	NPM1	ODC1
CEP70	CHAF1A	CHAF1B	CIART	CIT	CKAP5	COL11A1	CYR61	DACT1	DDR1	DENND3	DEPDC1B	DHCR24	DHFR	DIAPH3	DNAH7	DNMT1	E2F1	E2F8	ECT2	EFL1	EGR3	EMP3	EPHB6	ESPL1
FOXM1	GALNT14	H2AFV	HAUS5	HELLS	HMGA1	HSD17B4	HSP90AA1	KALRN	KIF23	KLF5	KNTC1	L3MBTL1	LIG1	MAD2L1	MCM2	MCM5	MKI67	MUTYH	MYBL2	MYC	MYH9	NPM1	PCNA	PCOLCE
KIF23	KNTC1	L3MBTL1	LIG1	MAD2L1	MCM5	MKI67	MUTYH	MYBL2	MYC	MYH9	NPM1	PCNA	PCOLCE	POLE	POLE2	POLQ	RNF219	RRM2	SVEP1	TNIK	TPX2	TYMS	UBE2C	

CCNB1	CCNB2	CCNB3	CCNF	CCSAP	CDC20	CDC25B	CDC45	CDC6	CDCA7	CDK1	CELSR2	CENPE	CENPF	CENPI	CENPJ	CENPK	CENPN	CENPO	CENPP	CEP192	CEP55	CEP70	CHAF1A	CHAF1B

FANCL	FASN	FBXO5	FOSL1	FOXM1	G2E3	GGT5	GINS4	HAP1	HELLS	HMOX1	HSD17B4	HSP90AA1	IL12B	INCENP	KCNK2	KIF22	KLF5	KLF6	LIG1	LPL	MAD2L1	MASTL	MCM10	MCM2

SLC2A4	STMN1	SUV39H1	TGM2	TONSL	TOP2A	TSC22D3	TTK	TYMS	UCP2	UHRF1	UNG	WWOX	XRCC4											
SUV39H1	TGM2	TONSL	TSC22D3	TTK	TYMS	UHRF1	UNG	WWOX	XRCC4															
RACGAP1	RPA1	RPA2	RRM2	SPAG5	STMN1	TACC3	TGM2	TOP2A	TPX2	TRAIP	TSC22D3	TTK	TYMS	WWOX										
NASP	NDC80	NFE2L1	NFIL3	NFIX	NIF3L1	NPM1	NUF2	ODC1	PBK	PCNA	PER1	PHGDH	PKMYT1	PLK1	PLK4	PRKCA	PRKCB	PRKDC	PTTG1	RACGAP1	RAD51	RNASEH2A	RPA1	RPA2
NASP	NDC80	NFE2L1	NFIL3	NFIX	NIF3L1	NPM1	NUF2	ODC1	PBK	PCNA	PER1	PHGDH	PKMYT1	PLK1	PLK4	PRKCA	PRKCB	PRKDC	PTTG1	RACGAP1	RAD51	RNASEH2A	RPA1	RPA2
MAD2L1	MAN2C1	MAP2K6	MAT2A	MCM10	MCM2	MCM8	MDC1	MELK	MIS18A	MKI67	MSH6	MSTN	MTFP1	MUTYH	MYBL2	MYC	NASP	NCAPG2	NCAPH2	NDC80	NET1	NFE2L1	NFIL3	NPM1
FOSL1	FOXM1	G2E3	GMDS	HAP1	HAUS1	HAUS8	HCAR1	HELLS	HMGA1	HMMR	HMOX1	HSD17B4	HSP90AA1	IL12B	INCENP	KCNK2	KIF14	KIF18A	KLF5	KLF6	KPNA2	LIG1	MAD2L1	MAN2C1
TACC3	TGM2	TNIK	TPX2	TTK																				
PRC1	PRKCA	PRKCB	PRKDC	RAB30	RACGAP1	SPAG5	STK35	STMN1	TACC3	TGM2	TNIK	TPX2	TTK											
MKI67	MMS22L	MSH6	MTR	MUTYH	MYC	NBAS	NCAPH	NFIX	NPM1	NUMA1	ODC1	PASK	PCNA	PER2	PER3	PEX11G	PLK1	POLA1	POLD1	POLE	POLE2	POLQ	PRIM2	PRKCB
FOXM1	FUT9	GGT5	HAP1	HCAR1	HELLS	HMGA1	HMMR	HMOX1	HP1BP3	HPX	HSP90AA1	IL12B	KALRN	KCNK2	KIF14	KIF15	KIF18A	KIF20A	KIF20B	KIF23	KIF2C	KLF5	KLF6	KPNA2
SLC2A4	STMN1	SUV39H1	TGM2	TNIK	TPX2	TRAIP	TYMS	UBE2C	UHRF1	WWOX														
CEP55	CEP70	CHAF1A	CHDH	CHTF18	CIART	CIT	CKAP2L	CKAP5	CKS2	CLOCK	CLSPN	CLYBL	COL11A1	CPT1A	CTNNBL1	CYR61	DACT1	DCHS2	DCK	DCTD	DDR1	DENND3	DENND6B	DEPDC1B
EXO1	EYS	FAM83D	FANCD2	FANCL	FASN	FBXL17	FGGY	FKBP5	FOCAD	FOXM1	G2E3	GALNT14	GANC	GGT5	GINS2	GINS3	GSTT1	GTSE1	HAP1	HAUS3	HAUS5	HAUS7	HAUS8	HELLS
FOXM1	G2E3	GGT5	GSTT1	GTSE1	HACL1	HAP1	HAUS2	HAUS8	HGD	HMMR	HMOX1	HPD	HPX	HSP90AA1	IQGAP3	KALRN	KCNK2	KIF14	KIF15	KIF20B	KIF23	KIF26B	KIFC1	KLHL31
FANCD2	FANCL	FASN	FBXL17	FGGY	FKBP5	FOCAD	FOXM1	G2E3	GALNT14	GANC	GGT5	GINS2	GINS3	GSTT1	GTSE1	HAP1	HAUS3	HAUS5	HAUS7	HAUS8	HELLS	HMGA1	HMMR	HMOX1
CLYBL	COL11A1	CPT1A	CTNNBL1	CYR61	DCHS2	DDR1	DENND3	DENND6B	DEPDC1B	DHFR	DIAPH3	DLGAP5	DNAH7	DNMT1	DNMT3B	DTL	E2F1	E2F8	EBI3	EFL1	EGR3	EHHADH	ELMOD3	EMP3
DEPTOR	DIAPH3	DLGAP5	DNAH7	DNMT1	DNMT3B	E2F8	ECT2	EFL1	EGR3	EHHADH	EPHB6	ESPL1	EXO1	EYS	FAAH	FAM83D	FANCD2	FANCL	FASN	FBXL17	FBXO32	FBXO5	FGGY	FIGNL1
MYBL2	MYC	MYH9	NASP	NCAPD2	NFE2L1	NFIL3	NR4A3	ODC1	PALB2	PCNA	PCOLCE	PLCH2	POLA1	POLD1	POLE	POLE2	POLQ	PRC1	PRKCA	PRKCB	PRKDC	PTTG1	RAD51	RAD54L
PCNA	PCOLCE	POLE	POLE2	POLQ	PRIM2	PRKCB	RNF219	RRM2	SLC29A2	STMN1	SVEP1	TNIK	TOP2A	TPX2	TYMS	UBE2C	WWOX							
EXO1	EYS	FAM83D	FANCD2	FANCL	FBXO5	FIGNL1	FOCAD	FOXM1	GGT5	GINS4	GMDS	HACL1	HAUS3	HAUS7	HAUS8	HMGA1	HMMR	HP1BP3	HPD	HPX	HSP90AA1	IL12B	KALRN	KIF14
POLE	POLE2	POLQ	PRIM2	PRKCB	RNF219	RRM2	SLC29A2	STMN1	SVEP1	TNIK	TOP2A	TPX2	TYMS	UBE2C										
CHDH	CHTF18	CIART	CIT	CKAP2L	CKAP5	CKS2	CLOCK	CLSPN	CLYBL	COL11A1	CPT1A	CTNNBL1	CYR61	DACT1	DCHS2	DCTD	DDR1	DENND3	DENND6B	DEPDC1B	DEPTOR	DHFR	DIAPH3	DLGAP5
METAP2	MIS18A	MKI67	MSH6	MSTN	MTF2	MTR	MUTYH	MYBL2	MYC	MYH9	NASP	NCAPG2	NCAPH	NCAPH2	NDC80	NFE2L1	NFIL3	NFIX	NPM1	NR4A3	NUMA1	NUSAP1	OAT	PALB2

RRM2	SLC29A2	SPAG5	STMN1	TACC3	TGM2	TOP2A	TPX2	TRAIP	TSC22D3	TTK	TYMS	UBE2C	UCP2	WWOX										
RRM2	SLC29A2	SPAG5	STMN1	TACC3	TGM2	TOP2A	TPX2	TRAIP	TSC22D3	TTK	TYMS	UBE2C	UCP2	WWOX										
NR4A3	NUF2	NUMA1	NUSAP1	ODC1	ORC2	PALB2	PBK	PCNA	PER1	PHGDH	PKMYT1	PLK1	PLK4	PRKCA	PRKCB	PRKDC	PTGER1	PTTG1	RACGAP1	RAD51	RBL1	RGS5	RPA1	RPA2
MAP2K6	MAT2A	MCM10	MCM2	MCM8	MDC1	MELK	METAP2	MIS18A	MKI67	MMS22L	MSH6	MSTN	MTBP	MTFP1	MUTYH	MYBL2	MYC	MYH9	NASP	NCAPG2	NCAPH2	NDC80	NET1	NFE2L1
PRKDC	RAD51	RAD52	RAD54B	RAD54L	RECQL4	RPA1	RRM2	SHMT1	SHMT2	SLC29A2	SMARCAL1	SMCHD1	STMN1	SVEP1	TOP2A	TPX2	TRIM63	TYMS	UBE2C	UNG	WDR90	WWOX	XRCC4	YBX2
LIG1	MAD2L1	MAP2K6	MCM10	MCM2	MCM3	MCM4	MCM5	MCM8	MELK	METAP2	MKI67	MSTN	MTBP	MTF2	MYBL2	MYC	MYH9	NASP	NCAPG	NEIL3	NET1	NFIL3	NFIX	NPM1
DEPTOR	DHCR24	DHFR	DIAPH3	DLGAP5	DNAH7	DNMT1	DNMT3B	DTL	E2F1	E2F8	EBI3	ECT2	EFL1	EGR3	EHHADH	ELMOD3	EMP3	ESPL1	EXO1	EXOC4	EYS	FAM172A	FAM64A	FAM83D
HMGA1	HMMR	HMOX1	HP1BP3	HPD	HSD17B4	IQGAP3	KALRN	KCNK2	KIF14	KIF20A	KIF20B	KIF23	KIF26B	KIFC1	KLF5	KLF6	KLHL31	KLHL38	KNSTRN	KNTC1	KPNA2	L3MBTL1	LPL	LRBA
KLHL38	KNTC1	MAT2A	MCM3	MCM4	MCM6	MDC1	MEGF6	MKI67	MMS22L	MSH6	MTBP	MTR	MYC	NASP	NBAS	NCAPD2	NCAPG	NCAPG2	NEIL3	NET1	NFE2L1	NFIX	NPM1	NR4A3
HP1BP3	HPD	HSD17B4	IQGAP3	KALRN	KCNK2	KIF14	KIF20A	KIF20B	KIF23	KIF26B	KIFC1	KLF5	KLF6	KLHL31	KLHL38	KNSTRN	KNTC1	KPNA2	L3MBTL1	LPL	LRBA	LRRCC1	MAD2L1	MAN1C1
ESPL1	EXO1	EXOC4	EYS	FAM172A	FAM64A	FAM83D	FANCD2	FANCI	FANCL	FASN	FBXL17	FGGY	FIGNL1	FKBP5	FOCAD	FOSL1	FOXM1	G2E3	GALNT14	GANC	GGT5	GINS2	GINS3	GINS4
FOCAD	FOXM1	G2E3	GALNT14	GANC	GGT5	GINS4	GMDS	GSTT1	GTSE1	HACL1	HAP1	HAUS3	HAUS5	HAUS7	HAUS8	HELLS	HMMR	HP1BP3	HPD	HPX	HSD17B4	HSP90AA1	IQGAP3	KALRN
RALGAPA2	RFC3	RNASEH2A	RPA2	RRM2	SGO1	SHMT2	SRL	STMN1	TACC3	TNNT2	TOP2A	TRAIP	TTK	TYMS	UBE2C	UHRF1	UPP1	WWOX	YBX2	ZNF367				
KIF15	KIF20A	KIF20B	KIF23	KIF2C	KIFC1	KLF6	KPNA2	LIG1	LRBA	LRRCC1	MAN1C1	MAP2K6	MASTL	MCM2	MCM8	MDC1	METTL15	MKI67	MSH6	MSTN	MTF2	MTFR2	MTR	MUTYH
DNAH7	DNMT1	DNMT3B	DTL	E2F1	E2F8	ECT2	EFL1	EGR3	EHHADH	ELMOD3	EMP3	EPHB6	ESPL1	EXO1	EXOC4	EYS	FAAH	FAM172A	FAM64A	FAM83D	FANCD2	FANCI	FANCL	FASN
PCNA	PER2	PHGDH	PLA2G10	PLK4	POLD1	POLG	PRKCA	PRKDC	PTTG1	RAD51	RAD52	RAD54B	RAD54L	RBL1	RECQL4	SHMT2	SLC2A4	SLC4A1	SMARCAL1	SMC2	TACC3	TGM2	TIMELESS	TKT

RRM2	SPAG5	STMN1	TACC3	TGM2	TNNT2	TOP2A	TPX2	TRAIP	TSC22D3	TTK	TYMS	UCP2	UNG	WWOX	XRCC4	YBX2								
NFIL3	NFIX	NIF3L1	NPM1	NR4A3	NUF2	NUMA1	NUSAP1	ODC1	ORC2	PALB2	PBK	PCNA	PER1	PHGDH	PKMYT1	PLA2G10	PLK1	PLK4	PRKCA	PRKCB	PRKDC	PTGER1	PTTG1	RACGAP1

NR4A3	NUMA1	NUPR2	ODC1	ORC1	ORC5	PBK	PCNA	PER1	PLA2G10	PLK1	PLK4	POLA1	POLG	PRC1	PRKCA	PRKCB	PRKDC	PTGER1	PTTG1	RAB30	RACGAP1	RAD51	RAD52	RBL1

FANCD2	FANCI	FANCL	FASN	FBXL17	FBXO32	FBXO5	FGGY	FIGNL1	FKBP5	FOCAD	FOSL1	FOXM1	G2E3	GALNT14	GANC	GGT5	GINS2	GINS3	GINS4	GMDS	GRHPR	GSTT1	GTSE1	H2AFV
LRRCC1	MAD2L1	MAN1C1	MAN2C1	MASTL	MAT2A	MCM10	MCM3	MCM4	MCM5	MCM8	MEGF6	MELK	MKI67	MSH6	MSTN	MTF2	MTR	MUTYH	MYBL2	MYC	MYH9	NCAPG	NCAPH	NCAPH2
NUMA1	NUSAP1	ODC1	PER1	PER3	PHF19	PHGDH	PIBF1	PIF1	PLK1	POLA1	POLD1	POLE	POLG	POLQ	PRC1	PRIM2	PRKCB	PRKDC	PTER	PTTG1	RABGAP1	RAD54B	RALGAPA2	RFC3
MAN2C1	MASTL	MAT2A	MCM10	MCM3	MCM4	MCM5	MCM8	MEGF6	MELK	MKI67	MSH6	MSTN	MTF2	MTR	MUTYH	MYBL2	MYC	MYH9	NCAPG	NCAPH	NCAPH2	NET1	NFE2L1	NNT
GMDS	GSTT1	GTSE1	HACL1	HAP1	HAUS3	HAUS5	HAUS7	HAUS8	HELLS	HMGA1	HMMR	HMOX1	HP1BP3	HPD	HSD17B4	IQGAP3	KALRN	KCNK2	KIF14	KIF15	KIF20A	KIF20B	KIF22	KIF23
KIF14	KIF15	KIF20A	KIF20B	KIF23	KIF26B	KIF2C	KIFC1	KLF5	KLF6	KLHL31	KLHL38	KNSTRN	KNTC1	KPNA2	L3MBTL1	LIG1	LPL	LRBA	LRRCC1	MAN1C1	MAN2C1	MAP2K6	MASTL	MCM10

MYBL2	MYC	MYH9	NCAPG	NFIL3	NFIX	NPM1	NR4A3	NSL1	NUF2	NUSAP1	OAT	ODC1	ORC2	ORC3	PAH	PALB2	PASK	PCNA	PER3	PHF19	PIBF1	PLK1	POLA1	POLE

FBXL17	FBXO32	FBXO5	FGGY	FIGNL1	FOCAD	FOSL1	FOXM1	G2E3	GALNT14	GANC	GGT5	GINS4	GMDS	GRHPR	GSTT1	GTSE1	H2AFV	HACL1	HAP1	HAUS3	HAUS5	HAUS7	HAUS8	HELLS

TNNT2	TOP2A	TRAIP	TRIM63	TXNRD2	UCP2	UHRF1	UNG	WWOX	XRCC4

RAD51	RBL1	RGS5	RNASEH2A	RPA1	RPA2	RRM2	SLC29A2	SLC2A4	SLC4A1	SPAG5	SPTB	STMN1	TACC3	TGM2	TNNT2	TOP2A	TPX2	TRAIP	TSC22D3	TTK	TXNRD2	TYMS	UBE2C	UCP2
RECQL4	RFC3	RGS5	RIDA	RPA1	RRM2	RTKN2	SHMT1	SHMT2	SLC29A2	SLC2A4	SLC4A1	SMARCAL1	STMN1	SUV39H1	TACC3	TGM2	TIMELESS	TNIK	TOP2A	TPX2	TRAIP	TSC22D3	TTK	TXNRD2
HACL1	HAP1	HAUS2	HAUS3	HAUS5	HAUS7	HAUS8	HCAR1	HELLS	HGD	HMGA1	HMMR	HMOX1	HP1BP3	HPD	HPX	HSD17B4	HSP90AA1	INCENP	IQGAP3	KALRN	KCNK2	KIF14	KIF15	KIF20A
NET1	NFE2L1	NNT	NR1D2	ODC1	ORC1	ORC5	PALB2	PARPBP	PASK	PBK	PCNA	PCOLCE	PER1	PER2	PEX11G	PGAM1	PHF19	PIGG	PLA2G10	PLCH2	PLK1	PLK4	POLA1	POLD1
RRM2	RTKN2	SASS6	SLC16A12	SLC45A4	SLC4A1	SMCHD1	SPTB	SVEP1	TECPR1	TGM2	THYN1	TICRR	TNIK	TNNT2	TONSL	TOP2A	TPX2	TRIM63	TTK	TXNRD2	TYMS	UBE2C	UBE2T	UCP2
NR1D2	ODC1	ORC1	ORC5	PALB2	PARPBP	PASK	PBK	PCNA	PCOLCE	PER1	PER2	PEX11G	PGAM1	PIGG	PLA2G10	PLCH2	PLK1	PLK4	POLA1	POLD1	POLE	POLE2	POLG	POLQ
KIF26B	KIFC1	KLF5	KLF6	KLHL31	KLHL38	KNSTRN	KNTC1	KPNA2	L3MBTL1	LHPP	LLPH	LPL	LRBA	LRRCC1	MAD2L1	MAN1C1	MAN2C1	MASTL	MAT2A	MCM10	MCM2	MCM3	MCM4	MCM5
MCM2	MCM4	MCM8	MDC1	MEGF6	MELK	METAP2	METTL15	MKI67	MSH6	MSTN	MTF2	MTFR2	MTR	MUTYH	MYBL2	MYC	MYH9	NASP	NCAPG	NCAPH	NCAPH2	NET1	NFE2L1	NFIL3
POLE2	POLQ	PRIM2	PRKCA	PRKCB	PRKDC	PTGER1	PTTG1	RABGAP1	RACGAP1	RAD54B	RALGAPA2	RBL1	RGS5	RHCG	RRM2	RTKN2	SHMT2	SLC16A12	SMARCAL1	SMC4	SPTB	STMN1	SVEP1	TECPR1
HMGA1	HMMR	HMOX1	HP1BP3	HPD	HPX	HSD17B4	HSP90AA1	IL12B	INCENP	IQGAP3	KALRN	KCNK2	KIF14	KIF15	KIF20A	KIF20B	KIF22	KIF23	KIF26B	KIF2C	KIFC1	KLF5	KLF6	KLHL31

UNG	WWOX	XRCC4	YBX2

TYMS	UBE2C	UCP2	UHRF1	UPP1	WNK2	WWOX	XRCC4

KIF20B	KIF22	KIF23	KIF26B	KIF2C	KIFC1	KLF5	KLF6	KLHL31	KLHL38	KNSTRN	KNTC1	KPNA2	L3MBTL1	LHPP	LLPH	LPL	LRBA	LRRCC1	MAD2L1	MAN1C1	MAN2C1	MASTL	MAT2A	MCM10
POLE	POLE2	POLG	POLQ	PRDM11	PRKCB	PRKDC	PTGER1	PTTG1	RAB30	RABGAP1	RACGAP1	RAD51	RAD52	RAD54B	RAD54L	RALGAPA2	RAVER1	RGS5	RHCG	RNASEH2A	RPA1	RRM2	RTKN2	SASS6
WDHD1	WNK2																							
PRDM11	PRKCB	PRKDC	PTGER1	PTTG1	RAB30	RABGAP1	RACGAP1	RAD51	RAD52	RAD54B	RAD54L	RALGAPA2	RAVER1	RGS5	RHCG	RNASEH2A	RPA1	RRM2	RTKN2	SASS6	SGO1	SLC16A12	SLC22A16	SLC29A2
MCM8	MDC1	MEGF6	MELK	MKI67	MMS22L	MSH6	MSTN	MTF2	MTFR2	MTR	MUTYH	MYBL2	MYC	MYH9	NBAS	NCAPD2	NCAPG	NCAPG2	NCAPH	NCAPH2	NEIL3	NET1	NFE2L1	NNT
NFIX	NNT	NPM1	NR1D2	NR4A3	NUF2	NUMA1	NUSAP1	OAT	ODC1	ORC3	ORC5	PAH	PALB2	PASK	PCOLCE	PER1	PER2	PER3	PEX11G	PGAM1	PHGDH	PIBF1	PIGG	PLA2G10

TGM2	THADA	TICRR	TNNT2	TOP2A	TPX2	TRIP13	TSC22D3	TTK	TYMS	UBE2C	WWOX	ZNF367	ZWILCH

KLHL38	KNSTRN	KNTC1	KPNA2	L3MBTL1	LHPP	LIG1	LLPH	LMBR1L	LPL	LRBA	LRRCC1	MAD2L1	MAN1C1	MAN2C1	MAP2K6	MASTL	MCM10	MCM2	MCM4	MCM5	MCM8	MDC1	MEGF6	MELK

MCM2	MCM3	MCM4	MCM5	MCM6	MCM8	MDC1	MEGF6	MELK	MIS12	MKI67	MMS22L	MSH6	MSTN	MTBP	MTF2	MTFR2	MTR	MUTYH	MYBL2	MYC	MYH9	NASP	NBAS	NCAPD2
SGO1	SLC16A12	SLC22A16	SLC29A2	SLC45A4	SLCO5A1	SMARCAL´	SMC2	SMC4	SMCHD1	SOX3	SPTB	ST6GALNA	STK35	SUV39H1	SVEP1	TACC3	TECPR1	THADA	THYN1	TICRR	TIMELESS	TKT	TMX3	TNIK

SLC45A4	SLCO5A1	SMARCAL´	SMC2	SMC4	SMCHD1	SOX3	SPTB	ST6GALNA	STK35	SUV39H1	SVEP1	TACC3	TECPR1	THADA	THYN1	TICRR	TIMELESS	TKT	TMX3	TNIK	TOP2A	TPX2	TRAIP	TRIP13
NR1D2	NR4A3	NUMA1	NUPR2	NUSAP1	ODC1	ORC1	ORC2	ORC3	ORC5	PALB2	PARPBP	PASK	PBK	PCNA	PCOLCE	PER1	PER2	PER3	PEX11G	PGAM1	PHF19	PIBF1	PIGG	PLA2G10
PLCH2	PLK1	PLK4	POLA1	POLD1	POLE	POLE2	POLG	POLQ	PRDM11	PRIM2	PRKCA	PRKDC	PTGER1	RAB30	RABGAP1	RACGAP1	RAD52	RAD54B	RAD54L	RALGAPA2	RAVER1	RBL1	RGS5	RHCG

METAP2	METTL15	MIS12	MKI67	MMS22L	MSH6	MSTN	MTF2	MTFR2	MTR	MUTYH	MYBL2	MYC	MYH9	NASP	NBAS	NCAPD2	NCAPG	NCAPG2	NCAPH	NCAPH2	NEIL3	NET1	NFE2L1	NFIL3

NCAPG	NCAPG2	NCAPH	NCAPH2	NEIL3	NET1	NFE2L1	NFIX	NIF3L1	NNT	NPM1	NR1D2	NR4A3	NUF2	NUMA1	NUPR2	NUSAP1	ODC1	ORC1	ORC2	ORC3	ORC5	PAH	PALB2	PARPBP
TOP2A	TPX2	TRAIP	TRIP13	TTK	TYMS	UBE2C	UCP2	UNG	WDHD1	WDR90	WNK2	WWOX	YBX2	ZWILCH										

TTK	TYMS	UBE2C	UCP2	UNG	WDHD1	WDR90	WNK2	WWOX	YBX2	ZWILCH															
PLCH2	PLK1	PLK4	POLA1	POLD1	POLE	POLE2	POLG	POLQ	PRC1	PRDM11	PRIM2	PRKCA	PRKCB	PRKDC	PTGER1	PTTG1	RAB30	RABGAP1	RACGAP1	RAD51	RAD52	RAD54B	RAD54L	RALGAPA2
RPA1	RRM2	RTKN2	SASS6	SGO1	SLC16A12	SLC22A16	SLC29A2	SLC45A4	SLC4A1	SLCO5A1	SMARCAL1	SMC2	SMC4	SMCHD1	SOX3	SPTB	ST6GALNA	STMN1	SUV39H1	SVEP1	TACC3	TECPR1	TGM2	THADA

NFIX	NIF3L1	NNT	NPM1	NR1D2	NR4A3	NUF2	NUMA1	NUPR2	NUSAP1	OAT	ODC1	ORC1	ORC2	ORC3	ORC5	PAH	PALB2	PASK	PBK	PCNA	PCOLCE	PER1	PER2	PER3

PASK	PBK	PCNA	PCOLCE	PER1	PER2	PER3	PEX11G	PGAM1	PHF19	PHGDH	PIBF1	PIF1	PIGG	PLA2G10	PLCH2	PLK1	PLK4	POLA1	POLD1	POLE	POLE2	POLG	POLQ	PRC1

RAVER1	RECQL4	RFC3	RGS5	RHCG	RNASEH2A	RNF219	RPA1	RPA2	RRM2	RTKN2	SASS6	SFXN5	SGO1	SHMT1	SHMT2	SLC16A12	SLC22A16	SLC29A2	SLC45A4	SLC4A1	SLCO5A1	SMARCAL1	SMC2	SMC4
THYN1	TICRR	TIMELESS	TKT	TMX3	TNIK	TNNT2	TOP2A	TPX2	TRAIP	TSC22D3	TTK	TYMS	UBE2C	UCP2	WDHD1	WDR90	WNK2	WWOX	YBX2	ZNF367	ZWILCH			

PEX11G	PGAM1	PHF19	PHGDH	PIBF1	PIGG	PLA2G10	PLCH2	PLK1	PLK4	POLA1	POLD1	POLE	POLE2	POLG	POLQ	PRC1	PRDM11	PRIM2	PRKCA	PRKCB	PRKDC	PTGER1	PTTG1	RAB30

PRDM11	PRIM2	PRKCA	PRKCB	PRKDC	PTER	PTGER1	PTTG1	RAB30	RABGAP1	RACGAP1	RAD51	RAD52	RAD54B	RAD54L	RALGAPA2	RAVER1	RECQL4	RFC3	RGS5	RHCG	RNASEH2A	RNF219	RPA1	RPA2	
SMCHD1	SMS	SOX3	SPAG5	SPTB	ST6GALNA	STAC2	STK35	SUV39H1	SVEP1	TACC3	TECPR1	TGM2	THADA	THYN1	TICRR	TIMELESS	TKT	TMX3	TNIK	TNNT2	TONSL	TOP2A	TPX2	TRAIP	
RABGAP1	RACGAP1	RAD51	RAD52	RAD54B	RAD54L	RALGAPA2	RAVER1	RBL1	RECQL4	RFC3	RGS5	RHCG	RNASEH2A	RNF219	RPA1	RPA2	RRM2	RTKN2	SASS6	SFXN5	SGO1	SHMT1	SHMT2	SLC16A12	

RRM2	RTKN2	SASS6	SFXN5	SGO1	SHMT1	SHMT2	SLC16A12	SLC22A16	SLC29A2	SLC45A4	SLC4A1	SLCO5A1	SMARCAL´	SMC2	SMC4	SMCHD1	SMS	SOX3	SPAG5	SPTB	ST6GALNA	STAC2	STK35	SUV39H1
TRIP13	TTK	TXLNB	TYMS	UBE2C	UCP2	UNG	WDHD1	WDR90	WNK2	WWOX	YBX2	ZWILCH												
SLC22A16	SLC29A2	SLC45A4	SLC4A1	SLCO5A1	SMARCAL´	SMC2	SMC4	SMCHD1	SMS	SOX3	SPAG5	SPTB	ST6GALNA	STAC2	STMN1	SUV39H1	SVEP1	TACC3	TECPR1	TGM2	THADA	THYN1	TICRR	TIMELESS

SVEP1 TACC3 TECPR1 TGM2 THADA THYN1 TICRR TIMELESS TKT TM7SF2 TMX3 TNIK TNNT2 TONSL TOP2A TPX2 TRAIP TRIM63 TRIP13 TTK TXLNB TXNRD2 TYMS UBE2C UBE2T

TKT TM7SF2 TMX3 TNIK TNNT2 TONSL TOP2A TPX2 TRAIP TRIM63 TRIP13 TSC22D3 TTK TXLNB TYMS UBE2C UCP2 WDHD1 WDR90 WNK2 WWOX YBX2 ZNF367 ZWILCH

UCP2 UHRF1 UNG WDHD1 WDR90 WNK2 WWOX YBX2 ZNF367 ZWILCH
