# Supplementary material for: Waveband specific transcriptional control of select genetic pathways in vertebrate skin (Xiphophorus maculatus)
Source: BMC Genomics. 2018 May 10;19:355. doi: 10.1186/s12864-018-4735-5 (PMC5946439; doi:10.1186/s12864-018-4735-5)
Supplement: Supplementary file 3 — Table S3. A complete list of all NanoString targets and probe sequences used to verify the RNA-Seq data for each waveband exposure. (ZIP 242 kb) [file 12864_2018_4735_MOESM3_ESM.zip › TableS3k_540-550nm.pdf]

**Function**	fatty acid oxi	organismal death
**z-score**	2.19	2.646
**number of ge**	6	38
**molecules**	GHR	AEBP1
	IGFBP5	AGRN
	LPL	ALOX12B
	MSTN	ALOXE3
	RBL1	ATM
	SIK3	ATR
		CDC45
		CDON
		COL10A1
		COL11A1
		COL1A1
		COL2A1
		COL5A1
		COL5A2
		COL7A1
		CSF1R
		CXCL12
		CYP1A1
		CYP1A2
		DOT1L
		GATA3
		GHR
		GPHN
		IGFBP5
		LPL
		MCM10
		MCM2
		MNX1
		MSTN
		POSTN
		RBL1
		RPL24
		SALL3
		SEMA5A
		SIK3
		SLC14A1
		SUZ12
		TRRAP
