# Supplementary material for: Waveband specific transcriptional control of select genetic pathways in vertebrate skin (Xiphophorus maculatus)
Source: BMC Genomics. 2018 May 10;19:355. doi: 10.1186/s12864-018-4735-5 (PMC5946439; doi:10.1186/s12864-018-4735-5)
Supplement: Supplementary file 3 — Table S3. A complete list of all NanoString targets and probe sequences used to verify the RNA-Seq data for each waveband exposure. (ZIP 242 kb) [file 12864_2018_4735_MOESM3_ESM.zip › TableS3j_530-540nm.pdf]

Function	dna repair	dna replicatio	chromosoma	organismal death
z-score	-2.59	-2.32	-2.37	3.35
number of ge	15	35	54	64
molecules	ADGRL2	ADORA1	ADAM8	ADORA1
	AGRN	AGRN	ADORA1	AEBP1
	ATM	ANXA5	ALOX15B	AGRN
	CHRNB2	AQP3	ANPEP	ALAS2
	DNMT3B	ATM	ANXA5	ALOX12B
	EPHB3	ATR	AQP3	ALOXE3
	GPM6A	CA2	ATM	ATM
	HMOX1	CERK	C6	ATR
	MMS22L	CNTFR	CAPN1	CAPN1
	PANX3	COL18A1	CELSR2	CDC45
	SLITRK3	COL1A1	COL11A1	CDON
	SRPX2	CSF1R	COL18A1	CERK
	THBS2	EPHB3	COL1A1	CHRNB2
	TNC	EPHB4	COL2A1	CNTFR
	TONSL	FA2H	COL7A1	COL10A1
		GATA1	CSF1R	COL11A1
		GFRA2	CTSE	COL1A1
		HBB	CYP1A1	COL25A1
		HBZ	CYP1A2	COL2A1
		HMOX1	EPHB3	COL5A1
		HSP90B1	EPHB4	COL5A2
		HSPA5	F3	COL7A1
		JDP2	GAPDH	CSF1R
		MEFV	GATA1	CYP1A1
		METAP2	GPM6A	CYP1A2
		MMP13	GRB7	DNMT3B
		MMS22L	HMOX1	DOT1L
		MST1R	HSP90B1	EPHB3
		NR4A1	HSPA5	F3
		PLXNB1	IGSF8	FAT4
		POSTN	LAMB1	GATA1
		SLC25A23	LAMB3	HBZ
		THBS2	LRRC15	HMOX1
		TONSL	MCM2	HSD11B2
		WT1	MMP13	HSP90B1
			MNX1	HSPA5
			MST1R	KLF1
			NR4A1	MBTD1
			PER1	MCM10

PLCD1	MCM2
PLXNB1	MCM3AP
POSTN	METAP2
PTX3	MFN1
SATB2	MNX1
SEMA5A	MRC1
SRPX2	MST1R
SUZ12	MSTN
TGFBI	PER2
THBS2	PLCD1
TNC	POSTN
UNC5C	PTX3
WASF1	RPL24
WNK1	SALL3
WT1	SATB2
	SEMA5A
	SIK3
	SLC14A1
	SLC4A1
	SUZ12
	TGFBI
	THBS2
	TRRAP
	WASF1
	WT1
