# Supplementary material for: Waveband specific transcriptional control of select genetic pathways in vertebrate skin (Xiphophorus maculatus)
Source: BMC Genomics. 2018 May 10;19:355. doi: 10.1186/s12864-018-4735-5 (PMC5946439; doi:10.1186/s12864-018-4735-5)
Supplement: Supplementary file 3 — Table S3. A complete list of all NanoString targets and probe sequences used to verify the RNA-Seq data for each waveband exposure. (ZIP 242 kb) [file 12864_2018_4735_MOESM3_ESM.zip › TableS3i_520-530nm.pdf]

Function	cell proliferat	cell viability	necrosis	apoptosis	organismal death
z-score	-2.24	-2.02	2.12	2.00	3.02
number of ge	47	53	94	106	100
molecules	ADGRL2	ACER2	AATK	AATK	ABCA1
	ALOX15B	AGRN	ACER2	ABCA1	AGRN
	AREG	ANGPT2	ADAM8	ADAM8	ALOX12B
	ARHGAP32	ANXA5	ADGRL2	ADAMTS20	ALOXE3
	ARNTL	AQP3	AGRN	ADAMTSL4	ANGPT2
	ARNTL2	AREG	ANGPT2	ADGRL2	APBA1
	ATF3	ATF3	AREG	AGRN	AREG
	ATR	ATR	ATAD2	ALOX15B	ARNTL
	CBX7	BHLHE40	ATF3	ANGPT2	ATF3
	CEP192	CA2	ATR	ANXA5	ATR
	CLASP1	CERK	BHLHE40	AQP3	C4A/C4B
	CSF1R	CISH	CA3	AREG	CACNA1A
	CUL7	CLOCK	CACNA1A	ATAD2	CAPN1
	CUL9	COL17A1	CAPN1	ATF3	CDC45
	DNMT3B	CSF1R	CAV3	ATR	CERK
	DOT1L	CTSB	CDC45	BHLHE40	CHRNB2
	E2F1	CUL9	CERK	C6	CHTF18
	E2F2	E2F1	CERS5	CA3	COL11A1
	EGFR	EGFR	CISH	CACNA1A	COL19A1
	FANCA	EPHB3	CLASP1	CAPN1	COL7A1
	FASN	EPHB4	CLOCK	CAV3	CREB5
	FOSL1	FA2H	COL5A3	CDC45	CSF1R
	HMOX1	FANCA	CSF1R	CHTF18	CSRP3
	HSPA1A/HSP	FASN	CTSB	CLASP1	CTSB
	IRS1	FAT1	CTSD	COL5A3	CTSD
	JARID2	FBXO32	CTSV	CSF1R	CTSV
	JDP2	FOSL1	CUL7	CTSB	CUL7
	LRP6	HBB	CUL9	CTSD	CUL9
	MCM2	HMGA1	CYFIP2	CTSL	CYP26A1
	MLLT6	HMOX1	DHCR24	CTSV	CYP51A1
	MMS22L	HSP90B1	DKK3	CUL7	DDR1
	MTOR	HSPA1A/HSP	DNMT3B	CUL9	DNAJB9
	PBRM1	HSPA5	DSP	CYFIP2	DNMT3B
	PER1	IL6ST	E2F1	DDAH2	DOT1L
	PER3	INSIG1	E2F2	DDR1	DSP
	PIK3R2	JAG2	EEF1A1	DHCR24	E2F1
	PRKCB	MMS22L	EGFR	DKK3	E2F2
	PTCH1	MTOR	EPHB4	DNMT3B	ECE1
	PTPRB	PIK3R2	EYS	DOT1L	EGFR

PTX3	PLXNB1	F3	DSP	EPHB3
RHOU	PPFIA2	FANCA	E2F1	F3
SKI	PPFIA4	FASN	E2F2	FASN
THBS1	PRKCB	FBXO32	ECE1	FAT1
TIMP2	PRKDC	FOSL1	EEF1A1	FLCN
TNC	PTCH1	FSTL3	EGFR	FOSL1
TRRAP	SLX4	GLI2	EPHB4	GLI2
WT1	SVIL	GPX2	F3	GPX2
	THBS1	HMGA1	FANCA	HMGCR
	TIMP2	HMOX1	FASN	HMOX1
	TNFRSF1A	HSD11B2	FBXO32	HSD11B2
	TONSL	HSP90B1	FLCN	HSP90B1
	TP53BP1	HSPA1A/HSP	FOSL1	HSPA5
	WT1	HSPA5	FSTL3	IL6ST
		IL6ST	GLI2	INSIG1
		ILKAP	GPX2	ITPR3
		IRS1	HMGA1	JAG2
		ITPR3	HMGCR	KIF26A
		LGMN	HMOX1	KMT2C
		LRP6	HSD11B2	LIAS
		MCM10	HSP90B1	LMO7
		MCM2	HSPA1A/HSP	LRP4
		MEFV	HSPA5	LRP6
		MKL1	IL6ST	MAGI2
		MMS22L	ILKAP	MCM10
		MSTN	IRS1	MCM2
		MTOR	ITPR3	MCM3AP
		NACC2	JAG2	MKL1
		NCOA1	KLF11	MNX1
		NEO1	LGMN	MSTN
		NPTX1	LRP6	MTOR
		NR3C2	MCM10	MTSS1
		ODC1	MCM2	NALCN
		PAK6	MEFV	NCOA1
		PER1	MKL1	NR3C2
		PIK3R2	MNX1	PHF21A
		PLXNB1	MSTN	PRKDC
		PRKCB	MTOR	PTCH1
		PRKDC	NACC2	PTPRJ
		PTCH1	NCOA1	PTPRS
		4-Sep	NPTX1	PTX3
		SKI	NR3C2	RPL24

SLC3A2	ODC1	SALL3
SLC5A5	PAK6	SEMA5A
SMOX	PER1	SIK3
SVIL	PIK3R2	SKI
THBS1	PRKCB	SLC14A1
TIMP2	PRKDC	SLC3A2
TNC	PTCH1	SLC4A10
TNFRSF1A	4-Sep	SLX4
TNS2	SHC4	STXBP5
TP53BP1	SKI	SUZ12
WFS1	SLX4	THBS1
WNK3	SMOX	TIMP2
WT1	SUZ12	TNFRSF1A
	TAOK1	TP53BP1
	THBS1	TRRAP
	TIMP2	WNT9B
	TNC	WT1
	TNFRSF1A	ZMIZ1
	TNS2	ZNF385A
	TP53BP1	
	WFS1	
	WNK3	
	WT1	
	YBX2	
	ZNF385A	
