# Supplementary material for: Waveband specific transcriptional control of select genetic pathways in vertebrate skin (Xiphophorus maculatus)
Source: BMC Genomics. 2018 May 10;19:355. doi: 10.1186/s12864-018-4735-5 (PMC5946439; doi:10.1186/s12864-018-4735-5)
Supplement: Supplementary file 3 — Table S3. A complete list of all NanoString targets and probe sequences used to verify the RNA-Seq data for each waveband exposure. (ZIP 242 kb) [file 12864_2018_4735_MOESM3_ESM.zip › TableS3h_510-520nm.pdf]

Function	cell proliferat	cell viability	dna repair	cytoplasm or	cytoskeleton	cellular recor	microtubule	necrosis	apoptosis	organismal death
z-score	-2.99	-3.61	-2.06	-4.28	-4.41	-2.39	-4.11	2.29	2.63	6.40
number of ge	72	176	10	126	122	81	110	166	183	177
molecules	ADARB1	ADAMTS20	ATM	AATK	AATK	ABCA1	AATK	AATK	AATK	ABCA1
	ADGRL2	ADGRF5	BRCA2	ABCA1	ABR	ACTC1	ADGRF5	ACER2	ABCA1	ABR
	ALOX15B	AGRN	EGFR	ABR	ADGRF5	ADAMTS2	ADGRG6	ACO2	ACO2	ACTC1
	ARHGAP32	ALOX15B	EZH2	ADGRF5	ADGRG6	ADGRF5	AGRN	ACTC1	ACTC1	ADARB1
	ARNTL2	ALOXE3	PBRM1	ADGRG6	AGRN	ANKRD1	ALS2	ADARB1	ADAMTS20	ADGRF5
	ATF3	ANGPT2	PRKDC	AGRN	ALS2	ATF3	ANGPT2	ADGRL2	ADAMTSL4	ADGRG6
	ATM	AQP3	RIF1	ALS2	ANGPT2	ATM	AQP1	AGRN	ADGRL2	AGRN
	ATR	ARHGAP26	TLR2	ANGPT2	APBB1IP	BRCA2	ARHGAP32	ALS2	AGRN	AKAP6
	BRCA2	ARHGAP32	TP53BP1	APBB1IP	AQP1	CBL	ARHGAP4	ANGPT2	ALOX15B	ALOX12B
	CALD1	ARL11	TRRAP	AQP1	ARHGAP32	CDH6	ARHGEF9	ANKRD1	ALS2	ALOXE3
	CBL	ATF3		ARHGAP32	ARHGAP4	CITED1	ATF3	ARHGAP4	ANGPT2	ANGPT2
	CBX7	ATM		ARHGAP4	ARHGEF9	CITED2	AVIL	ARL11	ANKRD1	AQP1
	CEP192	BHLHE40		ARHGEF9	ATF3	COL11A1	BCAS3	ATAD2	ANXA5	ARHGAP35
	CEP250	BNIP3		ATF3	AVIL	COL5A1	CALD1	ATF3	AQP1	ATF3
	CITED2	BRCA2		AVIL	BCAS3	CSF1R	CAMSAP2	ATM	AQP3	ATM
	CLASP1	BRD8		BCAS3	CALD1	CYP51A1	CBL	ATR	ARHGAP35	ATR
	COL1A1	CA2		BNIP3	CAMSAP2	DCHS1	CC2D2A	BCAS2	ARHGAP4	BAHCC1
	CSF1R	CALD1		CALD1	CBL	DSP	CELSR2	BHLHE40	ARL11	BRCA2
	CUL7	CBL		CAMSAP2	CC2D2A	ECE1	CEP192	BNIP3	ARL6IP5	CACNA2D2
	CUL9	CD109		CBL	CELSR2	EGFR	CEP250	BRCA2	ATAD2	CAPN1
	DDIT4	CHRNB2		CC2D2A	CEP192	EPHA3	CHRNB2	C8orf4	ATF3	CBL
	DNMT3B	CITED1		CELSR2	CEP250	EPHB3	CLASP1	CA3	ATM	CCDC86
	DOT1L	CITED2		CEP192	CHRNB2	EPHB4	CLSTN1	CACNA2D2	ATR	CDC45
	E2F1	CLSTN1		CEP250	CLASP1	ERMP1	CNTNAP1	CAPN1	BCAS2	CERK
	EGFR	CNTFR		CHRNB2	CLSTN1	FANCA	CNTNAP2	CBL	BHLHE40	CHRNB2
	ELK1	CNTNAP2		CLASP1	CNTNAP1	FANCL	COL25A1	CCDC86	BNIP3	CHTF18
	EZH2	COL24A1		CLSTN1	CNTNAP2	FAT4	CSF1R	CD33	BRCA2	CITED1
	FANCA	COL25A1		CNTNAP1	COL25A1	FGFR1	CTSV	CDC45	C8orf4	CITED2
	FASN	CREB5		CNTNAP2	CSF1R	FGFR2	CUL7	CDH6	CA3	CNTFR
	FGFR1	CREG1		COL25A1	CTSV	FHOD3	CUL9	CERK	CACNA2D2	CNTNAP1
	FGFR2	CSF1R		CSF1R	CUL7	GADD45G	DAB2IP	CERS5	CAPN1	COL11A1
	FIGNL1	CTSB		CTSV	CUL9	GATA3	DAG1	CERS6	CBL	COL1A1
	FOSL1	CTSE		CUL7	DAB2IP	GLI2	DDR1	CITED1	CCDC86	COL25A1
	GADD45G	CTSV		CUL9	DAG1	GLI3	DKK3	CLASP1	CD33	COL4A2
	GATA3	CYB5D2		DAB2IP	DDR1	HEYL	DOCK4	CNTFR	CDC45	COL5A1
	HMOX1	CYP26B1		DAG1	DKK3	HMOX1	DOCK7	COL1A1	CERS6	COL5A2
	IRS1	CYTL1		DDR1	DOCK4	HP	DSP	COL25A1	CHTF18	COL7A1
	JARID2	DAB2IP		DKK3	DOCK7	HSPB8	EGFR	COL4A2	CITED1	CREB5
	JDP2	DAG1		DOCK4	DSP	HSPG2	EIF2AK4	COL5A3	CITED2	CREM
	LIG1	DDIT4		DOCK7	EGFR	IFT172	ELK1	CREM	CLASP1	CSF1R
	MCM2	DDR1		DSP	EIF2AK4	IRS1	EPHA3	CSF1R	CNTFR	CTSB
	MLLT6	DNMT3A		EGFR	ELK1	ITGA1	EPHB3	CTSB	COL1A1	CTSD
	MMS22L	DNMT3B		EIF2AK4	EPHA3	ITGA6	EPHB6	CTSD	COL25A1	CTSV
	MYOD1	DOCK4		ELK1	EPHB3	ITGA8	ESPN	CTSV	COL4A2	CUL7
	NME1	DOCK7		EPHA3	EPHB6	JARID2	FASN	CUL7	COL5A3	CUL9
	PBRM1	DOT1L		EPHB3	ESPN	LAMC1	FGFR1	CUL9	CREM	CYP24A1
	PER1	DSP		EPHB6	F3	LIG1	FGFR2	CYFIP2	CSF1R	CYP26B1
	PER3	E2F1		ESPN	FASN	LRP4	GATA3	DAB2IP	CTSB	CYP51A1
	PHC1	EGFR		F3	FAT1	LZTS2	HERC1	DAG1	CTSD	DAG1
	PIK3R2	EGLN3		FASN	FGFR1	MBTD1	IFT172	DDIT4	CTSE	DCHS1
	PKMYT1	EIF2AK4		FAT1	FGFR2	MMP14	ITGA1	DKK3	CTSL	DDR1
	PLA2G10	ELK1		FGFR1	GATA3	MNX1	ITGA6	DNMT3A	CTSV	DNAJB9
	POLA1	EPHB3		FGFR2	HERC1	MSTN	ITGA8	DNMT3B	CUL7	DNMT3A
	PPP1R9B	EPHB4		GATA3	IFT172	ODC1	KIF5B	DSP	CUL9	DNMT3B
	PRKCA	EVC2		HERC1	ITGA1	PBRM1	LAMA1	E2F1	CYFIP2	DOT1L
	PTCH1	EXT1		IFT172	ITGA6	PHC1	LAMB1	EGFR	CYP26B1	DSP
	PTPRF	EZH2		ITGA1	ITGA8	PLCB1	LAMC1	EGLN3	DAB2IP	E2F1
	PTX3	FASN		ITGA6	KIF5B	PRKCA	LDLR	EIF2AK4	DAG1	ECE1
	RBL1	FBXO32		ITGA8	LAMA1	PRKDC	LINGO1	ELK1	DDIT4	EGFR
	RHOU	FGFR1		KIF5B	LAMB1	PTCH1	LRP1	EPHB4	DDR1	EGLN3
	SEMA3F	FGFR2		LAMA1	LAMC1	PTPRS	LRP4	EPHB6	DKK3	EHHADH
	SESN1	FIGNL1		LAMB1	LDLR	RBL1	LTB4R	EPX	DNMT3A	EIF2AK4

SKI	FLCN	LAMC1	LIG1	RFX3	MAGI2	EZH2	DNMT3B	EPHA3
SOCS1	FOSL1	LDLR	LINGO1	RLTPR	MARK4	F3	DOT1L	EPHB3
TLR2	FSTL3	LIG1	LPIN1	S1PR4	MIA	FAM134B	DSP	EPX
TNC	GADD45G	LINGO1	LRP1	SIK3	MINK1	FANCA	E2F1	EVC2
TRRAP	GATA3	LPIN1	LRP4	SLC23A1	MNX1	FANCL	ECE1	EXT1
TSG101	GFI1B	LRP1	LTB4R	SLC9A4	MTSS1	FASN	EDA	EZH2
TTC28	GLI2	LRP4	MAGI2	SOCS1	MYO6	FBXO32	EGFR	F3
WT1	GLI3	LTB4R	MARK4	SOX18	NCAM1	FGFR1	EGLN3	FANCL
WWC1	GNA15	MAGI2	MAST1	SREBF1	NEO1	FGFR2	EIF2AK4	FASN
XBP1	GNAL	MARK4	MIA	TCAP	NME1	FOSL1	ELK1	FAT1
	HEYL	MAST1	MINK1	TCF7	PAK6	FSTL3	EPHB4	FAT4
	HMOX1	MIA	MNX1	TLR2	PIP5K1B	GADD45G	EPHB6	FGFR1
	HSPG2	MINK1	MTSS1	TYR	PLCG2	GATA3	EZH2	FGFR2
	IKZF4	MNX1	MYO6	WNT7B	PLXNB1	GFI1B	F3	FLCN
	IL21R	MTSS1	NCAM1	WT1	PLXNB2	GLI2	FAM134B	FOSL1
	INSIG1	MYO6	NECTIN2	XBP1	PPP1R9B	GLI3	FANCA	GATA3
	IRS1	NCAM1	NEO1	YBX2	PRKCA	GNAL	FASN	GFI1B
	ITGA6	NECTIN2	NME1	ZFPM2	PTK7	HEYL	FAT4	GLI2
	JAG2	NEO1	PAK6	ZMIZ1	PTPDC1	HIC1	FBXO32	GNAL
	JARID2	NME1	PIP5K1B		PTPRF	HMOX1	FGFR1	HEYL
	JDP2	PAK6	PLCG2		PVR	HSPB8	FGFR2	HIC1
	KIF5B	PIP5K1B	PLXNB1		RAPH1	IDO1	FIGNL1	HMOX1
	LAMC1	PLCG2	PLXNB2		RELN	IGFBP4	FLCN	HP
	LDLR	PLXNB1	PPP1R9B		RFX3	IL21R	FNDC1	HPR
	LGALS1	PLXNB2	PRKCA		RHOU	ILKAP	FOSL1	HSD17B1
	LINGO1	PPP1R9B	PRKDC		SALL3	IRS1	FSTL3	HSPB8
	LPIN1	PRKCA	PTK7		SDK2	ITGA1	GADD45G	HSPG2
	LRP1	PRKDC	PTPDC1		SEMA3E	ITGA6	GATA3	IDO1
	LRP4	PTK7	PTPRF		SEMA3F	ITPR3	GFI1B	IFT172
	LTBP4	PTPDC1	PVR		SEMA5A	LDLR	GLI2	INSIG1
	MAGI2	PTPRF	RAPH1		4-Sep	LGALS1	GLI3	ITGA6
	MBNL1	PTX3	RELN		SLC9A5	LIG1	GNAL	ITGA8
	MINK1	PVR	RFX3		SLIT1	LINGO1	HEYL	ITPR3
	MMP14	RAPH1	RHOU		SLIT3	LRP1	HIC1	JAG2
	MMP19	RELN	SALL3		SLITRK3	LTB4R2	HMOX1	KIF26A
	MNX1	RFX3	SDK2		SRGAP1	LZTS2	HSPB8	KIF5B
	MSTN	RHOU	SEMA3E		SRGAP2	MCM10	HSPG2	LAMA1
	MTF2	SALL3	SEMA3F		SRGAP3	MCM2	IDO1	LAMC1
	MYO6	SDK2	SEMA5A		SV2A	MEFV	IFI27	LDLR
	MYOD1	SEC61A1	4-Sep		TEKT3	MIA	IGFBP4	LIAS
	NCAM1	SEMA3E	SHROOM4		TENM1	MMP14	ILKAP	LIG1
	NCOA1	SEMA3F	SLC3A2		TLR7	MMS22L	IRS1	LRP1
	NME1	SEMA5A	SLC9A5		TNC	MPHOSPH9	ITGA1	LRP4
	NME2	4-Sep	SLIT1		TNIK	MPO	ITGA6	MAGI2
	NMRK2	SHROOM4	SLIT3		UNC119B	MSTN	ITPR3	MBNL1
	OCA2	SLC3A2	SLITRK3		WISP2	MYO6	JAG2	MBTD1
	ODC1	SLC9A5	SRGAP1		WNT7B	MYOD1	KLF11	MCM10
	OGN	SLIT1	SRGAP2		ZSWIM6	NACC2	LAMA1	MCM2
	PER3	SLIT3	SRGAP3			NCAM1	LDLR	MED13
	PHC1	SLITRK3	SV2A			NCEH1	LGALS1	MMP14
	PIK3R2	SRGAP1	TEKT3			NCOA1	LIG1	MNX1
	PLCB1	SRGAP2	TENM1			NECTIN2	LINGO1	MPO
	PLCG2	SRGAP3	TLR7			NEO1	LRP1	MSTN
	PLXNB2	SV2A	TNC			NME1	LTB4R2	MTF2
	POU2F2	TEKT3	TNIK			NSD2	LZTS2	MTSS1
	PPM1L	TENM1	UNC119B			ODC1	MCM10	MYOD1
	PRKCA	TLR7	WDR62			OSGIN1	MCM2	NALCN
	PRKDC	TNC	WISP2			PAK6	MEFV	NCAM1
	PTCH1	TNIK	WNT7B			PER1	MIA	NCOA1
	PTPRF	UNC119B	ZSWIM6			PIK3R2	MMP14	NRG2
	RAPH1	WDR62				PKMYT1	MNX1	PHC1
	RBL1	WISP2				PLCB1	MPO	PHEX
	RELN	WNT7B				PLCG2	MSTN	PHF21A
	RLTPR	ZSWIM6				PLXNB1	MYO6	PLA2G10
	RNH1					PPP1R9B	MYOD1	PLCB1

RORB	PRKCA	NACC2	PLCG2
S1PR4	PRKDC	NCAM1	PLXNB2
SALL3	PTCH1	NCEH1	POLG
SATB2	PTPRF	NCOA1	POU2F2
SCUBE3	PTPRR	NECTIN2	PRKCA
SDK2	RBL1	NME1	PRKDC
SEMA3E	S1PR4	NME2	PTCH1
SEMA3F	SEMA3F	NRG2	PTPRF
SEMA4C	4-Sep	NSD2	PTPRS
SEMA5A	SH3PXD2A	ODC1	PTX3
SH3PXD2A	SIGLEC5	OSGIN1	RBL1
SHC4	SIGLEC8	PAK6	RFX3
SIGLEC10	SIGLEC9	PDE3A	RGMA
SIGLEC8	SKI	PER1	RIF1
SIK3	SLC1A3	PIK3R2	RPL24
SKI	SLC25A38	PKMYT1	SALL3
SLC1A3	SLC3A2	PLCG2	SATB2
SLC25A38	SLC9A4	PPP1R9B	SEMA5A
SLC3A2	SLIT1	PRKCA	SFRP5
SLIT1	SLIT3	PRKDC	SGK223
SNX19	SMOX	PTCH1	SH3PXD2A
SOCS1	SOCS1	PTP4A2	SIK3
SPAG9	SPIN1	PTPRF	SKI
SPON1	SREBF1	RBL1	SLC1A3
SREBF1	ST14	RHO	SLC23A1
SRGAP1	STC1	S1PR4	SLC3A2
SRGAP2	SVIL	SEMA3F	SMARCC2
ST14	TAX1BP1	4-Sep	SOCS1
STC1	TCF7	SFRP5	SREBF1
SUZ12	TLR2	SHC4	SRGAP3
SV2A	TLR7	SIGLEC5	ST14
TDO2	TNC	SIGLEC8	SUZ12
TET1	TP53BP1	SIGLEC9	SV2A
THRAP3	TSG101	SKI	SYNJ1
TLR2	TTR	SLC1A3	TAX1BP1
TLR7	TYR	SLC25A38	TENM3
TMBIM1	WNK3	SLC9A4	TLR2
TMEM120A	WT1	SMOX	TLR7
TNC	XBP1	SOCS1	TNXB
TPM4		SREBF1	TP53BP1
TSG101		ST14	TRRAP
WDR7		STC1	TSG101
WISP2		SUZ12	UNC13A
WNT7B		TAX1BP1	UNC79
WT1		TLR2	UPF1
XBP1		TLR7	WNT7B
ZFPM2		TNC	WT1
ZNF521		TP53BP1	XBP1
ZNF536		TSG101	ZFPM2
		TTR	ZMIZ1
		UPF1	
		WNK3	
		WT1	
		XBP1	
		YBX2	
		ZFAND5	
