# Supplementary material for: Waveband specific transcriptional control of select genetic pathways in vertebrate skin (Xiphophorus maculatus)
Source: BMC Genomics. 2018 May 10;19:355. doi: 10.1186/s12864-018-4735-5 (PMC5946439; doi:10.1186/s12864-018-4735-5)
Supplement: Supplementary file 3 — Table S3. A complete list of all NanoString targets and probe sequences used to verify the RNA-Seq data for each waveband exposure. (ZIP 242 kb) [file 12864_2018_4735_MOESM3_ESM.zip › TableS3g_500-510nm.pdf]

**Function**	lipid oxidatio	fatty acid oxi	organismal death
**z-score**	3.27	3.01	2.12
**number of ge**	17	9	54
**molecules**	AGT	ACSBG1	AGRN
	ALOX12B	AGT	AGT
	ALOXE3	CERK	ALOX12B
	APC	CYP1A1	ALOXE3
	CERK	CYP1A2	APC
	CYP1A1	EXTL1	ATR
	CYP51A1	GCK	BIRC6
	DHCR24	HMOX1	CAPN1
	EBP	SREBF1	CERK
	FASN		COL11A1
	HBB		COL7A1
	HMGCR		CUL7
	HMOX1		CUL9
	INSIG1		CYP1A1
	LSS		CYP1A2
	MSTN		CYP51A1
	SREBF1		DOT1L
			EPHB3
			FASN
			FAT4
			GATA3
			GCK
			GLI2
			HMGCR
			HMOX1
			HP
			HPR
			HSPA5
			INSIG1
			ISG15
			KMT2C
			LIAS
			LRP6
			MCM3AP
			MED13
			MNX1
			MSTN
			NCOA1
			NUP98
			PER2
			PHF21A
			PIK3R4
			PTHLH
			PTPRF
			RPL24
			SEMA5A
			SIK3
			SLC4A1
			SREBF1
			SUZ12
			TRRAP
			TSC1
			UBR2
			WT1
