# Supplementary material for: Waveband specific transcriptional control of select genetic pathways in vertebrate skin (Xiphophorus maculatus)
Source: BMC Genomics. 2018 May 10;19:355. doi: 10.1186/s12864-018-4735-5 (PMC5946439; doi:10.1186/s12864-018-4735-5)
Supplement: Supplementary file 3 — Table S3. A complete list of all NanoString targets and probe sequences used to verify the RNA-Seq data for each waveband exposure. (ZIP 242 kb) [file 12864_2018_4735_MOESM3_ESM.zip › TableS3f_550-600nm.pdf]

Function	cell prolifera	cytoplasm or	cytoskeleton	microtubule	inflammatior	fatty acid oxi	organismal death
z-score	3.50	-2.23	-2.07	-2.55	-3.86	2.70	4.53
number of g	72	36	35	30	11	11	49
molecules	ADAMTS7	AGRN	AGRN	AGRN	ATRN	CIDEC	AGRN
	ALS2	ALS2	ALS2	ALS2	COL11A1	FASN	BIRC6
	AXIN2	ARHGAP32	ARHGAP32	ARHGAP32	COL1A1	GCG	BPTF
	BIRC6	BCAS3	BCAS3	BCAS3	COL1A2	HMGA1	CASZ1
	BPTF	CELSR2	CELSR2	CELSR2	DENND4B	INS	CD151
	CACNA1D	CNTNAP2	CNTNAP2	CNTNAP2	ENO2	NCOA1	CDON
	CASZ1	CUL7	CUL7	CUL7	EPHB3	PARD3	COL11A1
	CD151	DCLK2	DCLK2	DCLK2	LRBA	PTPRF	COL12A1
	CDON	DNM1	DNM1	DNM1	SLIT3	SST	COL1A1
	CELSR2	DOCK4	DOCK4	DOCK4	TAX1BP1	TG	COL4A1
	CLEC19A	EGR3	EGR3	EGR3	XIRP1	TGM1	COL5A1
	CNTNAP2	EPHB3	EPHB3	EPHB3			COL7A1
	COL11A1	FASN	FASN	FASN			CUL7
	COL12A1	FAT1	FAT1	FLNA			DNM1
	COL15A1	FLNA	FLNA	HERC1			DOT1L
	COL1A2	HERC1	HERC1	KLHL41			EGR3
	COL21A1	INS	INS	LAMB1			EPHB3
	COL24A1	KLHL41	KLHL41	MAGI2			FASN
	COL4A1	LAMB1	LAMB1	MKL1			FAT1
	COL5A3	MAGI2	MAGI2	NEO1			FLNA
	COL6A3	MKL1	MKL1	PARD3			GCG
	DENND4B	NEO1	NEO1	PLXNB1			GDF6
	DMXL2	PARD3	PARD3	PTPN23			HSPB8
	DNM1	PKD1	PKD1	PTPRF			HSPG2
	DOCK4	PLXNB1	PLXNB1	RELN			INS
	EGR3	PRKDC	PRKDC	RERE			KLHL40
	FASN	PTPN23	PTPN23	RRAD			LRP6
	FAT1	PTPRF	PTPRF	SLIT3			MAGI2
	FAT3	RELN	RELN	TNIK			MCM3AP
	FBRS	RERE	RERE	ZDHHC8			MKL1
	FBRSL1	RRAD	RRAD				MRC1
	GDF6	SEC16A	SLIT3				NCOA1
	GLI3	SLIT3	STK35				NDST3
	HERC2	STK35	TNIK				PARD3
	HMGA1	TNIK	ZDHHC8				PER2
	HSPG2	ZDHHC8					PHF21A
	JARID2						PKD1
	KLF11						POLR2A
	KLHL30						PRKDC
	KLHL41						PTPRF
	LRBA						PTPRS
	MAGI1						SMG1
	MAGI2						SUZ12
	MRC1						TAB2
	MURC						TAX1BP1
	NAV3						TENM3

NCOA7
NHSL1
PARD3
PCOLCE2
PHF21A
PKD1
PLXNB1
PRKDC
PRR12
PTPRF
PTPRS
RELN
SCN8A
SLIT3
STK35
SUZ12
SVEP1
SVIL
TAX1BP1
TNIK
TNRC18
TRRAP
TTC28
UNC79
VPS13B
ZNF668

TGM1
TRRAP
UNC79
