# Supplementary material for: Waveband specific transcriptional control of select genetic pathways in vertebrate skin (Xiphophorus maculatus)
Source: BMC Genomics. 2018 May 10;19:355. doi: 10.1186/s12864-018-4735-5 (PMC5946439; doi:10.1186/s12864-018-4735-5)
Supplement: Supplementary file 3 — Table S3. A complete list of all NanoString targets and probe sequences used to verify the RNA-Seq data for each waveband exposure. (ZIP 242 kb) [file 12864_2018_4735_MOESM3_ESM.zip › TableS3e_500-550nm.pdf]

**Function**	DNA repair	M phase	chromosoma	S phase	G2/M Phase	inflammatior	lipid oxidatio	fatty acid oxidation
**z-score**	-3.00	-2.17	-2.52	-2.03	-3.61	-2.14	3.95	2.11
**number of g(**	45	26	66	40	13	8	14	12
**molecules**	ACSL1	ARID1A	ABCB11	ABCB11	ALPK3	C4A/C4B	ABCB11	CETP
	ALPK3	CAMK1G	ARID1A	ABHD8	ARID1A	CALCA	ACSL1	DGKZ
	AMPD1	CAPN3	BHLHE40	ABRA	COL11A1	DGKZ	ITPR3	GAPDH
	ARID1A	CELSR2	BST1	AMPD1	DENND4B	FBXO32	MAPK8	GPD1
	ASB10	CHRNA4	CALCA	ARID1A	GLB1L3	GATA3	MTNR1B	GYS2
	BCAP29	CTNNA2	CAMK1G	BST1	GLTSCR1	HMOX1	MVP	NOX1
	BTBD11	DHH	CAPN3	CELSR2	KIF21A	MAPK8	PDK2	PFKFB1
	CHRNA4	FAM107A	CD151	CLK1	LARP4B	MTMR4	PGAM2	PFKM
	CORO1C	GAPDH	CELSR2	CTNNA2	MYH13		PTP4A3	PIK3C2B
	CTNNA2	KIRREL3	CHRNA4	DENND4B	MYOM1		PTPRF	PKM
	CYP1A2	LGI1	CHRNB1	DPP10	PSME4		RRAD	PPARGC1A
	CYP2W1	LMOD2	CREB5	ESYT2	STEAP4		SSTR2	PPP1R3A
	DNAJB5	MAPK8	CSRP3	FHL1	SVIL		STEAP4	
	DPP10	MYOC	CTNNA2	GAPDH			USP13	
	DUSP26	MYOM1	CYP1A2	GATA3				
	EEF2	NR4A3	DGKZ	KBTBD3				
	EPRS	NYAP2	DHH	KIF21A				
	EPX	OBSL1	DKK2	KIRREL3				
	GATA3	PTPRF	EEF2	KLHL30				
	HIPK3	RERE	EPX	LRBA				
	ITPR3	SLIT3	FAM107A	LRRC30				
	JARID2	SLITRK3	FAM134B	MAP3K20				
	KBTBD3	SP4	FBXO32	MYH13				
	KIF21A	TCAP	GAPDH	NOX1				
	KIRREL3	TNIK	GATA3	NRAP				
	LMOD2	XIRP1	HMOX1	OPCML				
	LRBA		HSP90AA1	PFAS				
	MYF6		HSPA4	PFKM				
	MYOM1		ITPR3	PIK3C2B				
	NETO1		KIRREL3	PKM				
	NRAP		KLHL40	PPP1R3A				
	NYAP2		LGI1	PRKDC				
	OBSL1		LMOD2	SIK3				
	PER3		MAPK8	SLITRK3				
	PKM		MYF6	SVIL				
	PPP1R3A		MYOC	SYNPO2L				
	PRKDC		MYOM1	TNNI1				
	PSME4		NOX1	TP53BP2				
	PTPRF		NYAP2	TRIM63				
	RHCG		OBSL1	XIRP2				
	SLITRK3		PFKFB1					
	SYNPO2L		PFKM					
	TENM1		PPARGC1A					
	WDR17		PPP1R3A					
	XIRP2		PRKDC					
			PROM1					
			PSME4					
			PTPRF					
			RRAD					
			RERE					
			SCN1B					
			SIK3					
			SLIT3					
			SLITRK3					
			SP4					
			TCAP					
			TENM1					
			TLX1					
			TNIK					
			TNNT1					
			TP53BP2					
			TRIM55					
			TRIM63					
			WDR17					
			XIRP1					
			ZBTB16					
