# Supplementary material for: Waveband specific transcriptional control of select genetic pathways in vertebrate skin (Xiphophorus maculatus)
Source: BMC Genomics. 2018 May 10;19:355. doi: 10.1186/s12864-018-4735-5 (PMC5946439; doi:10.1186/s12864-018-4735-5)
Supplement: Supplementary file 3 — Table S3. A complete list of all NanoString targets and probe sequences used to verify the RNA-Seq data for each waveband exposure. (ZIP 242 kb) [file 12864_2018_4735_MOESM3_ESM.zip › TableS3d_450-500nm.pdf]

**Function**	cell proliferat	cell viability	inflammatior	necrosis	apoptosis	organismal death
**z-score**	3.15	2.25	2.88	2.50	-2.04	-2.92
**number of ge**	40	54	19	37	34	32
**molecules**	ADAM8	ADAM8	ADAM8	ADAM8	ADAM8	ACSS1
	ANGPT2	ANGPT2	ANGPT2	ANGPT2	ANGPT2	ANGPT2
	ANKRD1	ANKRD1	C5AR1	ANKRD1	ANKRD1	ATP2A2
	ARHGEF4	ARG2	CEBPB	ARG2	ARG2	C5AR1
	ATP2A2	ATP2A2	CSF3	ATP2A2	BCL6B	CA4
	BCL6B	BCL6B	GGT1	BCL6B	C5AR1	CEBPB
	C5AR1	C5AR1	HP	C5AR1	CA4	CREB5
	CCDC169	CA4	IL1B	CA4	CEBPB	CSF3
	CEBPB	CEBPB	KDR	CEBPB	CSF3	CYP24A1
	CH25H	CSF3	LDLR	CSF3	GCK	F8
	CREB5	CYP24A1	LTB4R	DENND4A	GGT1	GCK
	CSF3	DENND4A	MMP9	GCK	GNAT1	GGT1
	CYP24A1	DNAH7	PTAFR	GGT1	HSH2D	HP
	DENND4A	F13B	PTX3	HCAR1	HSP90B1	HSD17B1
	DNAH7	F8	RIPK3	HSH2D	HYOU1	HSP90B1
	F13B	GCK	TGM2	HSP90B1	IGFBP6	HYOU1
	F8	GGT1	TLR2	HYOU1	IL1B	IL1B
	GCK	GNAT1	TLR5	IGFBP6	KDR	KDR
	GGT1	HCAR1	TNFRSF9	IL1B	LDLR	LDLR
	GNAT1	HP		KDR	MME	MME
	HP	HSD17B1		LDLR	MMP9	MMP9
	HSD17B1	HSH2D		MME	NACC2	PFKFB3
	HSP90B1	HSP90B1		MMP9	PTAFR	PTAFR
	HYOU1	HYOU1		NACC2	RGS5	PTX3
	IGFBP6	IGFBP6		PFKFB3	RHO	RIPK3
	IL1B	IL1B		PTAFR	RIPK3	SALL1
	IL1R2	KDR		RGS5	S1PR4	SLC11A2
	KDR	LDLR		RIPK3	SALL1	TGM2
	LDLR	MME		S1PR4	SOAT1	TIMP2
	LPAR5	MMP9		SALL1	TGM2	TLR2
	MME	NACC2		SLC11A2	TIMP2	TLR5
	MMP9	OCSTAMP		SOAT1	TLR2	TNFRSF9
	SLC11A2	PFKFB3		TGM2	TNFAIP2	
	SOAT1	PTAFR		TIMP2	TNFRSF9	
	TGM2	RGS5		TLR2		
	TIMP2	RHO		TNFRSF9		
	TLR2	RIPK3		TYRP1		
	TLR5	S1PR4				
	TNFRSF9	SALL1				
	TYRP1	SLC11A2				

SOAT1
SPSB4
SQLE
STEAP4
TGM2
THRSP
TIMP2
TLR2
TNFAIP2
TNFRSF9
TYRP1
VEPH1
VWA5A
XIRP2
