# Supplementary material for: Waveband specific transcriptional control of select genetic pathways in vertebrate skin (Xiphophorus maculatus)
Source: BMC Genomics. 2018 May 10;19:355. doi: 10.1186/s12864-018-4735-5 (PMC5946439; doi:10.1186/s12864-018-4735-5)
Supplement: Supplementary file 3 — Table S3. A complete list of all NanoString targets and probe sequences used to verify the RNA-Seq data for each waveband exposure. (ZIP 242 kb) [file 12864_2018_4735_MOESM3_ESM.zip › TableS3c_400-450nm.pdf]

| Function | z-score | number of ge | molecules | | | | |
|---|---|---|---|---|---|---|---|
| cell proliferation | 2.00 | 5 | HMOX1 | KCNJ12 | NR4A3 | PPARGC1A | YBX2 |
| cell viability | 2.00 | 5 | HMOX1 | KCNJ12 | NR4A3 | PPARGC1A | YBX2 |
