# Supplementary material for: Waveband specific transcriptional control of select genetic pathways in vertebrate skin (Xiphophorus maculatus)
Source: BMC Genomics. 2018 May 10;19:355. doi: 10.1186/s12864-018-4735-5 (PMC5946439; doi:10.1186/s12864-018-4735-5)
Supplement: Supplementary file 3 — Table S3. A complete list of all NanoString targets and probe sequences used to verify the RNA-Seq data for each waveband exposure. (ZIP 242 kb) [file 12864_2018_4735_MOESM3_ESM.zip › TableS3b_350-400nm.pdf]

**Function**	cell viability	dna repair	check point c	inflammatior	necrosis	apoptosis	organismal death
**z-score**	-2.02	-2.11	-2.58	-2.35	-2.78	2.05	-2.67
**number of ge**	22	16	31	24	33	16	37
**molecules**	ANXA1	ARNTL	ALPK3	ANXA1	ANXA1	ANXA1	ANXA1
	ATM	BMPR1B	ANXA1	ARNTL	ATM	ATM	ARNTL
	BMPR1B	CTBP2	ARNTL	ATM	BMPR1B	BMPR1B	ATM
	CTBP2	ETV7	ATM	ATP1B2	CDC34	CDC34	BMPR1B
	CXCL12	EZR	BMPR1B	CLIC3	CTBP2	CXCL12	CDC34
	CYP1A1	HOXC13	CAMK1G	CXCL12	CXCL12	EZR	CTBP2
	ELOVL7	IRF1	CTBP2	DENND3	DNAJB1	GALNT3	CXCL12
	EMP1	NR4A3	CXCL12	EZR	DPM3	IRF1	DNAJB1
	ETV7	PAX6	EMP1	ITPR1	DSG2	ITPR1	DPM3
	EZR	PER2	ETV7	MAP2K6	EMP1	MAP2K6	DSG2
	FOXQ1	PPARGC1A	EVPL	MYO5B	EZR	NCEH1	EMP1
	GNA15	SAMD11	EZR	NCEH1	GALNT3	NR4A3	EZR
	IDH2	SOX1	GNA15	NIPA2	GALNT5	PDGFC	GALNT3
	IRF1	SOX2	HUNK	NPPC	GNPNAT1	PPARGC1A	GALNT5
	ITPR1	TMEM173	IDH2	NR4A3	IDH2	STAP2	GNPNAT1
	LRRN1	WNT5A	IRF1	OSBPL2	IRF1	WNT5A	HUNK
	MAP2K6		ITPR1	PAX6	ITPR1		IDH2
	MYH14		MAP2K6	PDYN	MAP2K6		IRF1
	PDGFC		MYO5B	PER2	MLKL		ITPR1
	SOX2		NPPC	PPARGC1A	NCEH1		MAP2K6
	STAP2		NR4A3	RANGAP1	NR4A3		MLKL
	WNT5A		PAX6	RSAD2	PAX6		NCEH1
			PPARGC1A	SEC23B	PDGFC		NR4A3
			PRSS12	SLC9A3R2	PLEKHF1		PAX6
			RSAD2		PPARGC1A		PDGFC
			SCEL		RAB25		PDYN
			SOX1		SIGIRR		PLEKHF1
			SOX2		SLC9A3R2		PPARGC1A
			SOX3		SOX2		RAB25
			TGM1		STAP2		SEC23B
			WNT5A		TGM1		SIGIRR
					TMEM173		SLC9A3R2
					WNT5A		SOX2
							STAP2
							TGM1
							TMEM173
							WNT5A
