# Supplementary material for: Waveband specific transcriptional control of select genetic pathways in vertebrate skin (Xiphophorus maculatus)
Source: BMC Genomics. 2018 May 10;19:355. doi: 10.1186/s12864-018-4735-5 (PMC5946439; doi:10.1186/s12864-018-4735-5)
Supplement: Supplementary file 3 — Table S3. A complete list of all NanoString targets and probe sequences used to verify the RNA-Seq data for each waveband exposure. (ZIP 242 kb) [file 12864_2018_4735_MOESM3_ESM.zip › TableS3a_FL.pdf]

| Function | cell proliferat | cell viability | dna repair | cytoplasm or | cytoskeleton | cellular recor | microtubule | dna replicatio | M phase | chromosoma | check point c | S phase | G2/M phase | inflammatior | lipid oxidatio | fatty acid oxi | necrosis | apoptosis | organismal death |
|---|---|---|---|---|---|---|---|---|---|---|---|---|---|---|---|---|---|---|---|
| z-score | -5.98 | -5.08 | -3.34 | -3.25 | -3.25 | -3.06 | -2.83 | -2.66 | -2.52 | -2.49 | -2.29 | -2.06 | -2.00 | 2.02 | 2.11 | 2.32 | 4.06 | 4.15 | 7.87 |
| number of g | 176 | 78 | 45 | 82 | 73 | 16 | 59 | 38 | 47 | 17 | 22 | 16 | 26 | 8 | 26 | 14 | 108 | 135 | 128 |
| molecules | ABCC5 | AGTPBP1 | ASCC3 | AGTPBP1 | AGTPBP1 | ATM | AGTPBP1 | ATM | ANLN | AURKA | ATM | ATM | ATM | DHFR | ACRC | AMT | AIMP1 | ADRB2 | ADRB2 |
| | ADRB2 | AIMP1 | ATM | ALS2 | ALS2 | BRCA1 | ALS2 | BRCA1 | ATM | CCNA2 | BRCA1 | BHLHE40 | AURKA | FBXO32 | ADM2 | ASNS | ALS2 | AIMP1 | AGL |
| | AIMP1 | ALS2 | BRCA1 | ASPM | ASPM | BRCA2 | AURKA | BRCA2 | AURKA | CENPE | BUB1 | CCNA2 | BRCA1 | POLE | BCAS3 | BHMT | ASNS | ALS2 | ARNTL |
| | ALS2 | ANLN | BRCA2 | AURKA | AURKA | EXO1 | BCAS3 | BRIP1 | AURKB | DLGAP5 | BUB1B | CDK1 | CCNA2 | POLE2 | BUB1B | GGT5 | ATAD2 | ASNS | ATM |
| | ARNTL | ASCC3 | BRIP1 | AURKB | AURKB | FANCD2 | BRCA1 | CCNA2 | BRCA2 | KIF14 | CCNB1 | E2F1 | CCNB1 | RRM2 | CEP192 | HGD | ATM | ATAD2 | AURKA |
| | ASCC3 | ASNS | CDK1 | BCAS3 | BCAS3 | MCM8 | CDC20 | CDC6 | BUB1B | KIF18A | CCNB2 | E2F8 | CDC25B | SLC2A4 | DHFR | HPD | AURKA | ATM | AURKB |
| | ASNS | ATM | E2F1 | BORA | BORA | MMS22L | CDC25B | CDK1 | CCNB1 | KIF22 | CDC20 | FOXM1 | CDK1 | TOP2A | E2F1 | MTR | AURKB | AURKA | BARD1 |
| | ASPM | AURKA | EXO1 | BRCA1 | BRCA1 | PALB2 | CDK1 | CDT1 | CDC20 | KIF2C | CDC6 | HMOX1 | CENPJ | TYMS | EPHB6 | MYC | BARD1 | AURKB | BRCA1 |
| | ATAD2 | AURKB | FANCD2 | CCNB1 | CCNB1 | PARPBP | CELSR2 | CKS2 | CDC25B | KIFC1 | CKS2 | MCM10 | CIT | | FOSL1 | OAT | BHLHE40 | BARD1 | BRCA2 |
| | ATM | BARD1 | FANCI | CDC20 | CDC20 | RAD51 | CENPE | E2F1 | CDC6 | MIS12 | E2F1 | MCM3 | CLSPN | | GSTT1 | PAH | BRCA1 | BHLHE40 | BRIP1 |
| | AURKA | BHLHE40 | FANCL | CDC25B | CDC25B | RAD52 | CENPF | FOXM1 | CDK1 | NCAPD2 | EXO1 | MYBL2 | DTL | | HPX | PHGDH | BRCA2 | BRCA1 | BUB1 |
| | AURKB | BRCA1 | FIGNL1 | CDK1 | CDK1 | RAD54B | CENPJ | HMGA1 | CDT1 | NCAPG | FANCD2 | MYC | FANCD2 | | HSP90AA1 | SHMT1 | BUB1 | BRCA2 | BUB1B |
| | BARD1 | BRCA2 | HMGA1 | CELSR2 | CELSR2 | RECQL4 | CEP192 | LIG1 | CENPE | NCAPG2 | KNTC1 | PLK1 | FOXM1 | | KCNK2 | SHMT2 | BUB1B | BUB1 | CCNA2 |
| | BHLHE40 | BRIP1 | HMOX1 | CENPE | CENPE | RFC3 | CEP70 | MCM2 | CENPJ | PLK1 | MAD2L1 | POLA1 | HMGA1 | | KIF20B | SMS | CABLES1 | CABLES1 | CCNB2 |
| | BHMT | BUB1B | KPNA2 | CENPF | CENPF | RPA2 | CIT | MCM3 | CEP55 | SGO1 | MAD2L1BP | RPA1 | MAD2L1 | | KIF23 | | CASC5 | CASC5 | CCNF |
| | BRCA1 | CCNA2 | LIG1 | CENPJ | CENPJ | TONSL | CKAP5 | MCM8 | CIT | SMC4 | MDC1 | TYMS | MAP2K6 | | MEGF6 | | CCNB1 | CCNA2 | CDC20 |
| | BRCA2 | CCNB1 | MSH6 | CEP192 | CEP192 | | CYR61 | MYC | CKS2 | TTK | MTBP | | MASTL | | MKI67 | | CDC20 | CCNB1 | CDC45 |
| | BRIP1 | CDK1 | MUTYH | CEP70 | CEP70 | | DACT1 | NASP | DIAPH3 | | MYC | | MCM10 | | ODC1 | | CDC6 | CDC20 | CDK1 |
| | BUB1 | CENPA | NEIL3 | CIT | CIT | | DDR1 | NPM1 | ECT2 | | NDC80 | | MDC1 | | PLK1 | | CDK1 | CDC25B | CENPE |
| | BUB1B | CKAP5 | NFE2L1 | CKAP5 | CKAP5 | | DIAPH3 | NR4A3 | FBXO5 | | PLK1 | | MELK | | PRC1 | | CDT1 | CDC45 | CENPJ |
| | CABLES1 | CLOCK | NPM1 | CYR61 | CYR61 | | DLGAP5 | ORC1 | INCENP | | PTTG1 | | MYBL2 | | PRKCB | | CENPE | CDC6 | CENPU |
| | CCNA2 | CYR61 | PALB2 | DACT1 | DACT1 | | ECT2 | ORC2 | KIF14 | | ZWILCH | | MYC | | RRM2 | | CENPI | CDK1 | CHAF1A |
| | CCNB1 | DCK | PCNA | DDR1 | DDR1 | | EGR3 | ORC3 | KIF20A | | | | NPM1 | | TICRR | | CKAP5 | CENPA | CHTF18 |
| | CCNB2 | DNMT1 | POLA1 | DIAPH3 | DIAPH3 | | EPHB6 | ORC5 | KIF20B | | | | PLK1 | | TOP2A | | CLSPN | CENPE | CIT |
| | CCNF | E2F1 | POLD1 | DLGAP5 | DLGAP5 | | FASN | PCNA | KIF23 | | | | PTTG1 | | TYMS | | CLYBL | CENPF | CKAP5 |
| | CDC20 | EGR3 | POLE | ECT2 | ECT2 | | FBXO5 | PLK1 | KIFC1 | | | | RPA1 | | UBE2C | | CYR61 | CENPJ | CKS2 |
| | CDC25B | FANCD2 | POLE2 | EGR3 | EGR3 | | FOXM1 | POLA1 | L3MBTL1 | | | | | | | | DCK | CHTF18 | COL11A1 |
| | CDC45 | FASN | POLG | EPHB6 | EPHB6 | | HAP1 | POLA2 | MAD2L1 | | | | | | | | DCTD | CIT | CPT1A |
| | CDC6 | FBXO32 | POLQ | ESPL1 | ESPL1 | | HAUS8 | POLD1 | MASTL | | | | | | | | DHCR24 | CKAP5 | CYR61 |
| | CDCA7 | FBXO5 | PRKDC | FASN | FASN | | HSP90AA1 | POLE | MCM4 | | | | | | | | DHFR | CTNNBL1 | DACT1 |
| | CDCA8 | FKBP5 | RAD51 | FBXO5 | FBXO5 | | KALRN | POLE2 | MCM8 | | | | | | | | DNMT1 | CYR61 | DDR1 |
| | CDK1 | FOSL1 | RAD52 | FHL3 | FHL3 | | KIF18A | POLG | MYH9 | | | | | | | | DTL | DCK | DIAPH3 |
| | CDT1 | FOXM1 | RAD54B | FOXM1 | FOXM1 | | KIF20B | RECQL4 | NCAPD2 | | | | | | | | DTYMK | DDR1 | DNMT1 |
| | CENPA | HAP1 | RAD54L | HAP1 | HAP1 | | KIF23 | RFC3 | NDC80 | | | | | | | | E2F1 | DEPTOR | DNMT3B |
| | CENPF | HMGA1 | RECQL4 | HAUS1 | HAUS8 | | KIF2C | RNASEH2A | NPM1 | | | | | | | | EGR3 | DHCR24 | DTL |
| | CENPJ | HMOX1 | RNASEH2A | HAUS2 | HSP90AA1 | | KIFC1 | RPA1 | NUF2 | | | | | | | | EPHB6 | DHFR | E2F1 |
| | CKS2 | KIF18A | RPA1 | HAUS3 | KALRN | | KLF5 | RPA2 | NUMA1 | | | | | | | | EYS | DNMT1 | E2F8 |
| | CLOCK | KIFC1 | RPA2 | HAUS5 | KIF18A | | MAD2L1 | TIMELESS | NUSAP1 | | | | | | | | FANCL | DNMT3B | ECT2 |
| | CLSPN | KLF5 | SUV39H1 | HAUS7 | KIF20B | | MID1IP1 | | PLK1 | | | | | | | | FASN | DTYMK | EGR3 |
| | CLYBL | KLF6 | TOP2A | HAUS8 | KIF23 | | MTBP | | PRC1 | | | | | | | | FBXO32 | E2F1 | EHHADH |
| | CYR61 | LIG1 | TRIP13 | HSP90AA1 | KIF2C | | MYC | | PTTG1 | | | | | | | | FBXO5 | E2F8 | ESPL1 |
| | DCK | MAD2L1 | TYMS | KALRN | KIFC1 | | MYH9 | | RACGAP1 | | | | | | | | FKBP5 | ECT2 | EXO1 |
| | DDR1 | MAP2K6 | UBE2T | KIF18A | KLF5 | | NDC80 | | RAD51 | | | | | | | | FOSL1 | EGR3 | FANCD2 |
| | DEPTOR | METAP2 | UNG | KIF20B | KNSTRN | | NET1 | | SPDL1 | | | | | | | | GMDS | EPHB6 | FANCL |
| | DHCR24 | MMS22L | XRCC4 | KIF23 | LIG1 | | NFIX | | TOP2A | | | | | | | | HAP1 | ESPL1 | FASN |
| | DHFR | MYBL2 | | KIF2C | MAD2L1 | | NUMA1 | | TRIP13 | | | | | | | | HAUS8 | EXO1 | FBXO5 |
| | DLGAP5 | MYC | | KIFC1 | MAP2K6 | | NUSAP1 | | UBE2C | | | | | | | | HCAR1 | FASN | FOSL1 |
| | DNMT1 | NDC80 | | KLF5 | MID1IP1 | | PHGDH | | | | | | | | | | HMGA1 | FBXO32 | FOXM1 |
| | DNMT3B | NFIL3 | | KNSTRN | MTBP | | PIBF1 | | | | | | | | | | HMMR | FBXO5 | G2E3 |
| | DTL | NR4A3 | | LIG1 | MYC | | PLA2G10 | | | | | | | | | | HMOX1 | FIGNL1 | GGT5 |
| | DTYMK | NUF2 | | MAD2L1 | MYH9 | | PLK4 | | | | | | | | | | INCENP | FKBP5 | GINS4 |
| | E2F1 | PALB2 | | MAP2K6 | NDC80 | | PRC1 | | | | | | | | | | KIF14 | FOSL1 | HAP1 |
| | E2F8 | PBK | | MID1IP1 | NET1 | | PRKCA | | | | | | | | | | KLF5 | G2E3 | HELLS |
| | EBI3 | PCNA | | MTBP | NFIX | | PRKCB | | | | | | | | | | KLF6 | GMDS | HMOX1 |
| | ECT2 | PLK1 | | MTFP1 | NUMA1 | | RACGAP1 | | | | | | | | | | KPNA2 | HAP1 | HSD17B4 |
| | EGR3 | PRKCA | | MTFR2 | NUSAP1 | | STMN1 | | | | | | | | | | LIG1 | HAUS1 | HSP90AA1 |
| | EMP3 | PRKCB | | MYC | PHGDH | | TACC3 | | | | | | | | | | MAD2L1 | HELLS | IL12B |
| | EPHB6 | PRKDC | | MYH9 | PIBF1 | | TNIK | | | | | | | | | | MAN2C1 | HMGA1 | INCENP |
| | ESPL1 | RAD51 | | NDC80 | PLA2G10 | | TPX2 | | | | | | | | | | MAP2K6 | HMMR | KCNK2 |
| | EXOC4 | RAD52 | | NET1 | PLK4 | | | | | | | | | | | | MAT2A | HMOX1 | KIF22 |
| | FAM83D | RAD54B | | NFIX | PRC1 | | | | | | | | | | | | MCM10 | HSP90AA1 | KLF5 |
| | FANCD2 | RAD54L | | NUMA1 | PRKCA | | | | | | | | | | | | MDC1 | IL12B | KLF6 |
| | FANCL | RPA1 | | NUSAP1 | PRKCB | | | | | | | | | | | | MELK | KIF14 | LIG1 |
| | FASN | RRM2 | | PHGDH | PRKDC | | | | | | | | | | | | MMS22L | KIF18A | LPL |
| | FBXO32 | SHMT2 | | PIBF1 | RACGAP1 | | | | | | | | | | | | MSTN | KLF5 | MAD2L1 |
| | FIGNL1 | SKA1 | | PLA2G10 | SPAG5 | | | | | | | | | | | | MTFP1 | KLF6 | MASTL |
| | FKBP5 | SLC2A4 | | PLK1 | STK35 | | | | | | | | | | | | MYBL2 | KPNA2 | MCM10 |
| | FOSL1 | STMN1 | | PLK4 | STMN1 | | | | | | | | | | | | MYC | LIG1 | MCM2 |
| | FOXM1 | SUV39H1 | | PRC1 | TACC3 | | | | | | | | | | | | NASP | MAD2L1 | METAP2 |
| | FUT9 | TGM2 | | PRKCA | TGM2 | | | | | | | | | | | | NDC80 | MAN2C1 | MIS18A |
| | GGT5 | TONSL | | PRKCB | TNIK | | | | | | | | | | | | NFE2L1 | MAP2K6 | MKI67 |
| | HAP1 | TSC22D3 | | PRKDC | TPX2 | | | | | | | | | | | | NFIL3 | MAT2A | MSH6 |
| | HCAR1 | TTK | | RAB30 | TTK | | | | | | | | | | | | NFIX | MCM10 | MSTN |
| | HELLS | TYMS | | RACGAP1 | | | | | | | | | | | | | NIF3L1 | MCM2 | MTF2 |
| | HMGA1 | UHRF1 | | SPAG5 | | | | | | | | | | | | | NPM1 | MCM8 | MTR |
| | HMMR | UNG | | STK35 | | | | | | | | | | | | | NUF2 | MDC1 | MUTYH |
| | HMOX1 | WWOX | | STMN1 | | | | | | | | | | | | | ODC1 | MELK | MYBL2 |
| | HP1BP3 | XRCC4 | | TACC3 | | | | | | | | | | | | | PBK | MIS18A | MYC |
| | HPX | | | TGM2 | | | | | | | | | | | | | PCNA | MKI67 | MYH9 |
| | HSP90AA1 | | | TNIK | | | | | | | | | | | | | PER1 | MSH6 | NASP |
| | IL12B | | | TPX2 | | | | | | | | | | | | | PHGDH | MSTN | NCAPG2 |
| | KALRN | | | TTK | | | | | | | | | | | | | PKMYT1 | MTFP1 | NCAPH |
| | KCNK2 | | | | | | | | | | | | | | | | PLK1 | MUTYH | NCAPH2 |
| | KIF14 | | | | | | | | | | | | | | | | PLK4 | MYBL2 | NDC80 |
| | KIF15 | | | | | | | | | | | | | | | | PRKCA | MYC | NFE2L1 |
| | KIF18A | | | | | | | | | | | | | | | | PRKCB | NASP | NFIL3 |
| | KIF20A | | | | | | | | | | | | | | | | PRKDC | NCAPG2 | NFIX |
| | KIF20B | | | | | | | | | | | | | | | | PTTG1 | NCAPH2 | NPM1 |
| | KIF23 | | | | | | | | | | | | | | | | RACGAP1 | NDC80 | NR4A3 |
| | KIF2C | | | | | | | | | | | | | | | | RAD51 | NET1 | NUMA1 |
| | KLF5 | | | | | | | | | | | | | | | | RNASEH2A | NFE2L1 | NUSAP1 |
| | KLF6 | | | | | | | | | | | | | | | | RPA1 | NFIL3 | OAT |
| | KPNA2 | | | | | | | | | | | | | | | | RPA2 | NPM1 | PALB2 |
| | LIG1 | | | | | | | | | | | | | | | | RRM2 | NR4A3 | PCNA |
| | MAD2L1 | | | | | | | | | | | | | | | | SLC29A2 | NUF2 | PER2 |
| | MAP2K6 | | | | | | | | | | | | | | | | SPAG5 | NUMA1 | PHGDH |
| | MCM10 | | | | | | | | | | | | | | | | STMN1 | NUSAP1 | PLA2G10 |
| | MCM2 | | | | | | | | | | | | | | | | TACC3 | ODC1 | PLK4 |
| | MCM3 | | | | | | | | | | | | | | | | TGM2 | ORC2 | POLD1 |
| | MCM4 | | | | | | | | | | | | | | | | TOP2A | PALB2 | POLG |
| | MCM5 | | | | | | | | | | | | | | | | TPX2 | PBK | PRKCA |
| | MCM8 | | | | | | | | | | | | | | | | TRAIP | PCNA | PRKDC |
| | MELK | | | | | | | | | | | | | | | | TSC22D3 | PER1 | PTTG1 |
| | METAP2 | | | | | | | | | | | | | | | | TTK | PHGDH | RAD51 |
| | MKI67 | | | | | | | | | | | | | | | | TYMS | PKMYT1 | RAD52 |
| | MSTN | | | | | | | | | | | | | | | | UBE2C | PLK1 | RAD54B |
| | MTBP | | | | | | | | | | | | | | | | UCP2 | PLK4 | RAD54L |
| | MTF2 | | | | | | | | | | | | | | | | WWOX | PRKCA | RBL1 |
| | MYBL2 | | | | | | | | | | | | | | | | | PRKCB | RECQL4 |
| | MYC | | | | | | | | | | | | | | | | | PRKDC | SHMT2 |
| | MYH9 | | | | | | | | | | | | | | | | | PTGER1 | SLC2A4 |
| | NASP | | | | | | | | | | | | | | | | | PTTG1 | SLC4A1 |

| | | |
|---|---|---|
| NCAPG | RACGAP1 | SMARCAL1 |
| NEIL3 | RAD51 | SMC2 |
| NET1 | RBL1 | TACC3 |
| NFIL3 | RGS5 | TGM2 |
| NFIX | RPA1 | TIMELESS |
| NPM1 | RPA2 | TKT |
| NR4A3 | RRM2 | TNNT2 |
| NUMA1 | SPAG5 | TOP2A |
| NUPR2 | STMN1 | TRAIP |
| ODC1 | TACC3 | TRIM63 |
| ORC1 | TGM2 | TXNRD2 |
| ORC5 | TNNT2 | UCP2 |
| PBK | TOP2A | UHRF1 |
| PCNA | TPX2 | UNG |
| PER1 | TRAIP | WWOX |
| PLA2G10 | TSC22D3 | XRCC4 |
| PLK1 | TTK | |
| PLK4 | TYMS | |
| POLA1 | UCP2 | |
| POLG | UNG | |
| PRC1 | WWOX | |
| PRKCA | XRCC4 | |
| PRKCB | YBX2 | |
| PRKDC | | |
| PTGER1 | | |
| PTTG1 | | |
| RAB30 | | |
| RACGAP1 | | |
| RAD51 | | |
| RAD52 | | |
| RBL1 | | |
| RECQL4 | | |
| RFC3 | | |
| RGS5 | | |
| RIDA | | |
| RPA1 | | |
| RRM2 | | |
| RTKN2 | | |
| SHMT1 | | |
| SHMT2 | | |
| SLC29A2 | | |
| SLC2A4 | | |
| SLC4A1 | | |
| SMARCAL1 | | |
| STMN1 | | |
| SUV39H1 | | |
| TACC3 | | |
| TGM2 | | |
| TIMELESS | | |
| TNIK | | |
| TOP2A | | |
| TPX2 | | |
| TRAIP | | |
| TSC22D3 | | |
| TTK | | |
| TXNRD2 | | |
| TYMS | | |
| UBE2C | | |
| UCP2 | | |
| UHRF1 | | |
| UPP1 | | |
| WNK2 | | |
| WWOX | | |
| XRCC4 | | |
