# Supplementary material for: Waveband specific transcriptional control of select genetic pathways in vertebrate skin (Xiphophorus maculatus)
Source: BMC Genomics. 2018 May 10;19:355. doi: 10.1186/s12864-018-4735-5 (PMC5946439; doi:10.1186/s12864-018-4735-5)
Supplement: Supplementary file 4 — Table S4a–k. A list of all differentially modulated genes used by IPA enrichment software to predict the direction of change for each functional class represented in Fig. 4. Table a is FL, tables b–e are the 50 nm wavebands and tables g–k are the 10 nm wavebands. (ZIP 262 kb) [file 12864_2018_4735_MOESM4_ESM.zip › TableS4k_540-550nm.pdf]

**Function**	ingestion	organismal death
**z-score**	-2.342	2.646
**number of ge**	6	38
**molecules**	ATM	AEBP1
	GHR	AGRN
	MSTN	ALOX12B
	PER1	ALOXE3
	SLC14A1	ATM
	SLC14A2	ATR
		CDC45
		CDON
		COL10A1
		COL11A1
		COL1A1
		COL2A1
		COL5A1
		COL5A2
		COL7A1
		CSF1R
		CXCL12
		CYP1A1
		CYP1A2
		DOT1L
		GATA3
		GHR
		GPHN
		IGFBP5
		LPL
		MCM10
		MCM2
		MNX1
		MSTN
		POSTN
		RBL1
		RPL24
		SALL3
		SEMA5A
		SIK3
		SLC14A1
		SUZ12
		TRRAP
