# Supplementary material for: Waveband specific transcriptional control of select genetic pathways in vertebrate skin (Xiphophorus maculatus)
Source: BMC Genomics. 2018 May 10;19:355. doi: 10.1186/s12864-018-4735-5 (PMC5946439; doi:10.1186/s12864-018-4735-5)
Supplement: Supplementary file 4 — Table S4a–k. A list of all differentially modulated genes used by IPA enrichment software to predict the direction of change for each functional class represented in Fig. 4. Table a is FL, tables b–e are the 50 nm wavebands and tables g–k are the 10 nm wavebands. (ZIP 262 kb) [file 12864_2018_4735_MOESM4_ESM.zip › TableS4j_530-540nm.pdf]

**Function**	benign neopl	differentiatio	quantity of b	quantity of c	migration of	fibrosis	adhesion of c	organismal death
**z-score**	2.172	-2.802	2.317	2.172	-2.484	2.34	-2.573	3.35
**number of ge**	42	63	27	12	49	18	8	64
**molecules**	AEBP1	ADAM8	ADAM8	ATM	ADAM8	ADORA1	COL1A1	ADORA1
	AGRN	ADORA1	ANPEP	CAPN1	ADORA1	COL1A1	COL2A1	AEBP1
	ANXA5	AGRN	ATM	COL1A1	ALOX15B	COL1A2	COL7A1	AGRN
	ATM	ALAS2	C6	DNMT3B	ANPEP	CSF1R	POSTN	ALAS2
	ATR	ALOX15B	CERK	FSTL3	ANXA5	CYP1A2	TGFBI	ALOX12B
	ATRN	ALOXE3	COL10A1	GATA1	AQP3	F3	THBS2	ALOXE3
	CA1	ANPEP	CSF1R	HSD11B2	ATM	FSTL3	TNC	ATM
	CA2	AQP3	CTSE	PER1	C6	HBB	TNMD	ATR
	COL10A1	ATM	CYP1A1	PER2	CELSR2	HMOX1		CAPN1
	COL11A1	BRD8	F3	POSTN	COL11A1	HPX		CDC45
	COL11A2	CA2	GATA1	SIK3	COL18A1	LRRC15		CDON
	COL15A1	CDON	HBB	WT1	COL1A1	MMP13		CERK
	COL16A1	CHRNB2	HBZ		COL7A1	MSTN		CHRNB2
	COL18A1	CNTFR	HMOX1		CSF1R	POSTN		CNTFR
	COL1A1	COL11A2	HPX		CTSE	PTX3		COL10A1
	COL1A2	COL18A1	HSP90B1		CYP1A1	SLC4A1		COL11A1
	COL21A1	COL25A1	JARID2		CYP1A2	THBS2		COL1A1
	COL22A1	COL2A1	MBTD1		EPHB3	WT1		COL25A1
	COL25A1	CSF1R	MGAT5B		EPHB4			COL2A1
	COL27A1	CTSE	MMP13		F3			COL5A1
	COL2A1	CYB5D2	NR4A1		GATA1			COL5A2
	COL4A6	CYTL1	PER2		GPM6A			COL7A1
	COL5A1	DNMT3B	PLCD1		GRB7			CSF1R
	COL5A2	DOT1L	SLC14A1		HMOX1			CYP1A1
	COL5A3	EPHB3	SLC4A1		HSP90B1			CYP1A2
	COL6A3	EPHB4	TGFBI		HSPA5			DNMT3B
	COL7A1	EXTL1	THBS2		IGSF8			DOT1L
	COX6A2	FSTL3			LAMB1			EPHB3
	CSF1R	GAPDH			LAMB3			F3
	DNMT3B	GATA1			LRRC15			FAT4
	ENTPD5	GPM6A			MCM2			GATA1
	EPHB3	HMOX1			MMP13			HBZ
	FKBP10	HPX			MNX1			HMOX1
	GPM6A	HSP90B1			MST1R			HSD11B2
	HMOX1	HSPA5			NR4A1			HSP90B1
	HSD11B2	JARID2			PER1			HSPA5
	LAMB3	JDP2			PLCD1			KLF1
	MST1R	KLF1			PLXNB1			MBTD1
	NR4A1	LAMB3			POSTN			MCM10
	RORB	MFN1			PTX3			MCM2
	THBS2	MGAT5B			SATB2			MCM3AP
	TNC	MMP13			SEMA5A			METAP2
		MNX1			SRPX2			MFN1
		MST1R			SUZ12			MNX1
		MSTN			TGFBI			MRC1
		NR4A1			THBS2			MST1R
		OCSTAMP			TNC			MSTN
		OGN			UNC5C			PER2
		PANX3			WASF1			PLCD1
		PER3						POSTN
		PLCD1						PTX3
		POSTN						RPL24
		RORB						SALL3
		SALL3						SATB2
		SATB2						SEMA5A
		SEMA5A						SIK3
		SIK3						SLC14A1
		SRPX2						SLC4A1
		SUZ12						SUZ12
		THBS2						TGFBI
		TMBIM1						THBS2
		TNC						TRRAP
		WT1						WASF1
								WT1
