# Supplementary material for: Waveband specific transcriptional control of select genetic pathways in vertebrate skin (Xiphophorus maculatus)
Source: BMC Genomics. 2018 May 10;19:355. doi: 10.1186/s12864-018-4735-5 (PMC5946439; doi:10.1186/s12864-018-4735-5)
Supplement: Supplementary file 4 — Table S4a–k. A list of all differentially modulated genes used by IPA enrichment software to predict the direction of change for each functional class represented in Fig. 4. Table a is FL, tables b–e are the 50 nm wavebands and tables g–k are the 10 nm wavebands. (ZIP 262 kb) [file 12864_2018_4735_MOESM4_ESM.zip › TableS4i_520-530nm.pdf]

Function	quantity of c	cell proliferat	benign neopl	cell viability	differentiatio	quantity of b	quantity of c	vasculogenes	hypertrophy	fibrosis	necrosis	apoptosis	stress respon	cell death	organismal death
z-score	-2.274	-2.24	-2.144	-2.02	-2.401	-2.255	-2.056	-2.012	2.5	2.208	2.12	2.00	-2.709	2.004	3.02
number of g	74	47	53	53	100	41	15	36	20	25	94	106	9	125	100
molecules	ABCA1	ADGRL2	AGRN	ACER2	ADAM8	ABCA1	ANGPT2	ANGPT2	ABCA1	ANGPT2	AATK	AATK	ANGPT2	AATK	ABCA1
	ADAM8	ALOX15B	ANGPT2	AGRN	ADAMTS20	ADAM8	ARNTL	ARNTL	ANGPT2	ATF3	ACER2	ABCA1	CTSB	ABCA1	AGRN
	AGRN	AREG	ANXA5	ANGPT2	AGRN	ARNTL	CAPN1	ATF3	ATF3	CAV3	ADAM8	ADAM8	CTSD	ABCC5	ALOX12B
	ALOX15B	ARHGAP32	ATR	ANXA5	ALOX15B	ATF3	CTSV	C6	CA2	CISH	ADGRL2	ADAMTS20	DNAJB9	ACER2	ALOXE3
	ANGPT2	ARNTL	ATRN	AQP3	ALOXE3	BHLHE40	DNMT3B	CSF1R	CAV3	CSF1R	AGRN	ADAMTSL4	HMOX1	ADAM8	ANGPT2
	ARNTL	ARNTL2	C4A/C4B	AREG	ANGPT2	C4A/C4B	EGFR	CTSB	CSRP3	CSRP3	ANGPT2	ADGRL2	HSP90B1	ADAMTS20	APBA1
	ARNTL2	ATF3	CA1	ATF3	AQP3	C6	FSTL3	CUL7	CTSB	CTSB	AREG	AGRN	HSPA5	ADAMTSL4	AREG
	ATF3	ATR	CA2	ATR	AREG	CACNA1A	GLI2	CYP51A1	CTSD	DSP	ATAD2	ALOX15B	MTOR	ADGRL2	ARNTL
	BHLHE40	CBX7	CACNA1A	BHLHE40	ARHGAP26	CERK	HSD11B2	DDR1	ECE1	EGFR	ATF3	ANGPT2	NR3C2	AGRN	ATF3
	C4A/C4B	CEP192	CBX7	CA2	ARHGAP32	CISH	IL6ST	E2F1	EGFR	F3	ATR	ANXA5		ALOX15B	ATR
	C6	CLASP1	COL11A1	CERK	ARNTL	CSF1R	IRS1	ECE1	FBXO32	FSTL3	BHLHE40	AQP3		ANGPT2	C4A/C4B
	CACNA1A	CSF1R	COL16A1	CISH	ATF3	CTSD	JAG2	EGFR	FSTL3	HBB	CA3	AREG		ANXA5	CACNA1A
	CAPN1	CUL7	COL17A1	CLOCK	BHLHE40	CTSV	PER1	EPHB3	HMGA1	HMOX1	CACNA1A	ATAD2		AQP3	CAPN1
	CERK	CUL9	COL19A1	COL17A1	CA2	CYP27A1	SIK3	EPHB4	HMGCR	IL6ST	CAPN1	ATF3		AREG	CDC45
	CHRNB2	DNMT3B	COL21A1	CSF1R	CACNA1A	DDR1	WT1	F3	HMOX1	LGMN	CAV3	ATR		ARNTL	CERK
	CISH	DOT1L	COL24A1	CTSB	CAV3	DKK3		HMOX1	IL6ST	LRRC15	CDC45	BHLHE40		ATAD2	CHRNB2
	COL5A3	E2F1	COL27A1	CUL9	CD109	E2F1		IL6ST	MKL1	MSTN	CERK	C6		ATF3	CHTF18
	CSF1R	E2F2	COL4A6	E2F1	CHRNB2	E2F2		IRS1	MTOR	MTSS1	CERS5	CA3		ATR	COL11A1
	CTSB	EGFR	COL5A3	EGFR	CISH	F3		LUZP1	NR3C2	NR3C2	CISH	CACNA1A		BHLHE40	COL19A1
	CTSD	FANCA	COL7A1	EPHB3	CNTN4	FANCA		MTOR	PRKCB	PRKCB	CLASP1	CAPN1		C4A/C4B	COL7A1
	CTSV	FASN	CSF1R	EPHB4	COL24A1	HBB		NCOA1		PTX3	CLOCK	CAV3		C6	CREB5
	CUL7	FOSL1	CTSB	FA2H	CREB5	HMOX1		ODC1		THBS1	COL5A3	CDC45		CA3	CSF1R
	CYP27A1	HMOX1	CUL9	FANCA	CREG1	HSP90B1		PLXNB1		TIMP2	CSF1R	CHTF18		CACNA1A	CSRP3
	DDR1	HSPA1A/HSP	DNMT3B	FASN	CSF1R	IL6ST		PRKDC		TNFRSF1A	CTSB	CLASP1		CAPN1	CTSB
	DKK3	IRS1	DPT	FAT1	CSRP3	JAG2		PTPRB		WT1	CTSD	COL5A3		CAV3	CTSD
	DNMT3B	JARID2	E2F1	FBXO32	CTSB	JARID2		PTPRJ			CTSV	CSF1R		CBX7	CTSV
	DOT1L	JDP2	E2F2	FOSL1	CTSV	LGMN		PTX3			CUL7	CTSB		CDC45	CUL7
	DSP	LRP6	EGFR	HBB	CYB5D2	LRP6		ROBO4			CUL9	CTSD		CERK	CUL9
	E2F1	MCM2	EPHB3	HMGA1	CYP26A1	MKL1		SEMA5A			CYFIP2	CTSL		CERS5	CYP26A1
	E2F2	MLLT6	FKBP10	HMOX1	CYP27A1	MMP19		SMOC2			DHCR24	CTSV		CHTF18	CYP51A1
	EGFR	MMS22L	GLI2	HSP90B1	DDR1	NR3C2		THBS1			DKK3	CUL7		CISH	DDR1
	F3	MTOR	HMGA1	HSPA1A/HSP	DNMT3B	PRKCB		TIMP2			DNMT3B	CUL9		CLASP1	DNAJB9
	FANCA	PBRM1	HMGCR	HSPA5	DOCK4	PRKDC		TNC			DSP	CYFIP2		CLOCK	DNMT3B
	FSTL3	PER1	HMOX1	IL6ST	DOT1L	PTPRJ		WNK1			E2F1	DDAH2		COL5A3	DOT1L
	GLI2	PER3	HSD11B2	INSIG1	DSP	SLC14A1		WT1			E2F2	DDR1		CSF1R	DSP
	HBB	PIK3R2	IL6ST	JAG2	E2F1	STEAP4		ZMIZ1			EEF1A1	DHCR24		CTSB	E2F1
	HMGA1	PRKCB	JAG2	MMS22L	E2F2	THBS1					EGFR	DKK3		CTSD	E2F2
	HMOX1	PTCH1	KMT2C	MTOR	EBP	TIMP2					EPHB4	DNMT3B		CTSL	ECE1
	HSD11B2	PTPRB	MTOR	PIK3R2	EGFR	TNFRSF1A					EYS	DOT1L		CTSV	EGFR
	HSP90B1	PTX3	NR3C2	PLXNB1	EPHB3	TP53BP1					F3	DSP		CUL7	EPHB3
	IL6ST	RHOU	ODC1	PPFIA2	EPHB4	ZNF385A					FANCA	E2F1		CUL9	F3
	IRS1	SKI	OLFM1	PPFIA4	FASN						FASN	E2F2		CYFIP2	FASN
	JAG2	THBS1	PBRM1	PRKCB	FBXO32						FBXO32	ECE1		DDAH2	FAT1
	JARID2	TIMP2	PRKCB	PRKDC	FLCN						FOSL1	EEF1A1		DDR1	FLCN
	LGMN	TNC	PTCH1	PTCH1	FOSL1						FSTL3	EGFR		DHCR24	FOSL1
	LRP4	TRRAP	RELN	SLX4	FSTL3						GLI2	EPHB4		DKK3	GLI2
	LRP6	WT1	RORB	SVIL	GLI2						GPX2	F3		DNMT3B	GPX2

MKL1	SLC5A5	THBS1	HMGA1	HMGA1	FANCA	DOT1L	HMGCR
MMP19	THBS1	TIMP2	HMOX1	HMOX1	FASN	DSP	HMOX1
MNX1	TNC	TNFRSF1A	HSP90B1	HSD11B2	FBXO32	E2F1	HSD11B2
MSTN	TNFRSF1A	TONSL	HSPA1A/HSPA1B	HSP90B1	FLCN	E2F2	HSP90B1
MTOR	UGP2	TP53BP1	HSPA5	HSPA1A/HSP	FOSL1	ECE1	HSPA5
NEO1	WISP2	WT1	IL6ST	HSPA5	FSTL3	EEF1A1	IL6ST
NR3C2			INSIG1	IL6ST	GLI2	EGFR	INSIG1
PER1			IRS1	ILKAP	GPX2	EPHB4	ITPR3
PRKCB			JAG2	IRS1	HMGA1	EYS	JAG2
PRKDC			JARID2	ITPR3	HMGCR	F3	KIF26A
PTPRJ			JDP2	LGMN	HMOX1	FANCA	KMT2C
PTPRS			LRP4	LRP6	HSD11B2	FASN	LIAS
RELN			LRP6	MCM10	HSP90B1	FBXO32	LMO7
SIK3			LTBP4	MCM2	HSPA1A/HSPA1B	FLCN	LRP4
SKI			MAGI2	MEFV	HSPA5	FOSL1	LRP6
SLC14A1			METRN	MKL1	IL6ST	FSTL3	MAGI2
SLC17A8			MKL1	MMS22L	ILKAP	GLI2	MCM10
STEAP4			MMP19	MSTN	IRS1	GPX2	MCM2
THBS1			MNX1	MTOR	ITPR3	GRB7	MCM3AP
TIMP2			MSTN	NACC2	JAG2	HBB	MKL1
TNC			MTOR	NCOA1	KLF11	HMGA1	MNX1
TNFRSF1A			NCOA1	NEO1	LGMN	HMGCR	MSTN
TP53BP1			NMRK2	NPTX1	LRP6	HMOX1	MTOR
WFS1			NR3C2	NR3C2	MCM10	HSD11B2	MTSS1
WT1			OCSTAMP	ODC1	MCM2	HSP90B1	NALCN
YBX2			ODC1	PAK6	MEFV	HSPA1A/HSP	NCOA1
ZNF385A			PER3	PER1	MKL1	HSPA5	NR3C2
			PIK3R2	PIK3R2	MNX1	IL6ST	PHF21A
			PRKCB	PLXNB1	MSTN	ILKAP	PRKDC
			PRKDC	PRKCB	MTOR	IRS1	PTCH1
			PTCH1	PRKDC	NACC2	ITPR3	PTPRJ
			PTPRJ	PTCH1	NCOA1	JAG2	PTPRS
			RELN	4-Sep	NPTX1	KLF11	PTX3
			RLTPR	SKI	NR3C2	LGMN	RPL24
			RORB	SLC3A2	ODC1	LRP6	SALL3
			SALL3	SLC5A5	PAK6	MCM10	SEMA5A
			SEMA5A	SMOX	PER1	MCM2	SIK3
			SHC4	SVIL	PIK3R2	MEFV	SKI
			SIK3	THBS1	PRKCB	MKL1	SLC14A1
			SKI	TIMP2	PRKDC	MMS22L	SLC3A2
			SLC3A2	TNC	PTCH1	MNX1	SLC4A10
			SNX19	TNFRSF1A	4-Sep	MSTN	SLX4
			SRGAP2	TNS2	SHC4	MTOR	STXBP5
			SUZ12	TP53BP1	SKI	NACC2	SUZ12
			THBS1	WFS1	SLX4	NCOA1	THBS1
			TIMP2	WNK3	SMOX	NEO1	TIMP2
			TMBIM1	WT1	SUZ12	NPTX1	TNFRSF1A
			TNC		TAOK1	NR3C2	TP53BP1
			TNFRSF1A		THBS1	ODC1	TRRAP
			WFIKKN2		TIMP2	OLFM1	WNT9B

WISP2	TNC	PAK6	WT1
WT1	TNFRSF1A	PER1	ZMIZ1
ZNF385A	TNS2	PIK3R2	ZNF385A
	TP53BP1	PLXNB1	
	WFS1	PRKCB	
	WNK3	PRKDC	
	WT1	PTCH1	
	YBX2	4-Sep	
	ZNF385A	SHC4	
		SKI	
		SLC3A2	
		SLC5A5	
		SLX4	
		SMOX	
		SUZ12	
		SVIL	
		TAOK1	
		THBS1	
		TIMP2	
		TNC	
		TNFRSF1A	
		TNS2	
		TP53BP1	
		WFS1	
		WNK3	
		WT1	
		YBX2	
		ZNF385A	
