# Supplementary material for: Waveband specific transcriptional control of select genetic pathways in vertebrate skin (Xiphophorus maculatus)
Source: BMC Genomics. 2018 May 10;19:355. doi: 10.1186/s12864-018-4735-5 (PMC5946439; doi:10.1186/s12864-018-4735-5)
Supplement: Supplementary file 4 — Table S4a–k. A list of all differentially modulated genes used by IPA enrichment software to predict the direction of change for each functional class represented in Fig. 4. Table a is FL, tables b–e are the 50 nm wavebands and tables g–k are the 10 nm wavebands. (ZIP 262 kb) [file 12864_2018_4735_MOESM4_ESM.zip › TableS4h_510-520nm.pdf]

Function	quantity of c	cell proliferat	transcription	benign neopl	cell viability	differentiatio	development	development	migration of	migration of	necrosis	apoptosis	cell death	organismal death
z-score	-2.519	-2.99	-2.044	2.511	-3.61	-3.612	-2.389	-2.515	-2.239	-2.586	2.29	2.63	2.376	6.40
number of ge	114	72	128	81	176	176	81	43	14	135	166	183	214	177
molecules	ABCA1	ADARB1	AEBP2	ACTC1	ADAMTS20	ADAMTS20	ABCA1	ATF3	ANGPT2	ABCA1	AATK	AATK	AATK	ABCA1
	ADGRF5	ADGRL2	AGRN	ADARB1	ADGRF5	ADGRF5	ACTC1	ATM	ATF3	ABR	ACER2	ABCA1	ABCA1	ABR
	AGRN	ALOX15B	ALOX12B	AGRN	AGRN	AGRN	ADAMTS2	BRCA2	CBL	ADARB1	ACO2	ACO2	ABCC5	ACTC1
	ALOX15B	ARHGAP32	ANKRD1	ANGPT2	ALOX15B	ALOX15B	ADGRF5	CDH6	CTSV	ADGRF5	ACTC1	ACTC1	ACER2	ADARB1
	ALS2	ARNTL2	ARHGAP35	ANXA5	ALOXE3	ALOXE3	ANKRD1	CITED1	DDR1	ADGRG3	ADARB1	ADAMTS20	ACO2	ADGRF5
	ANGPT2	ATF3	ARNTL2	ARHGAP35	ANGPT2	ANGPT2	ATF3	CITED2	F3	ALOX15B	ADGRL2	ADAMTSL4	ACTC1	ADGRG6
	APOA4	ATM	ATAD2	ATM	AQP3	AQP3	ATM	CSF1R	FGFR1	ANGPT2	AGRN	ADGRL2	ADAMTS20	AGRN
	AQP1	ATR	ATF3	ATR	ARHGAP26	ARHGAP26	BRCA2	DCHS1	HMOX1	ANXA5	ALS2	AGRN	ADAMTSL4	AKAP6
	ARNTL2	BRCA2	ATM	ATRN	ARHGAP32	ARHGAP32	CARMIL2	ERMP1	IGFBP4	AQP1	ANGPT2	ALOX15B	ADARB1	ALOX12B
	ATF3	CALD1	AUTS2	BRCA2	ARL11	ARL11	CBL	FANCA	LDLR	AQP3	ANKRD1	ALS2	ADGRL2	ALOXE3
	ATM	CBL	AVPR2	CA1	ATF3	ATF3	CDH6	FANCL	NEO1	ARHGAP35	ARHGAP4	ANGPT2	AGRN	ANGPT2
	BHLHE40	CBX7	BCAS3	CA2	ATM	ATM	CITED1	FAT4	PTPRF	ARHGAP4	ARL11	ANKRD1	ALOX15B	AQP1
	BRCA2	CEP192	BHLHE40	CALB2	BHLHE40	BHLHE40	CITED2	FGFR1	TLR2	ASAP2	ATAD2	ANXA5	ALS2	ARHGAP35
	CACNA2D2	CEP250	BRCA2	CBL	BNIP3	BNIP3	COL11A1	FGFR2	WISP2	ATF3	ATF3	AQP1	ANGPT2	ATF3
	CAPN1	CITED2	BRD8	CBX7	BRCA2	BRCA2	COL5A1	GADD45G		ATM	ATM	AQP3	ANKRD1	ATM
	CBL	CLASP1	C1QTNF1	COL11A1	BRD8	BRD8	CSF1R	GATA3		BCAS3	ATR	ARHGAP35	ANXA5	ATR
	CERK	COL1A1	CBL	COL14A1	CA2	CA2	CYP51A1	GLI2		BHLHE40	BCAS2	ARHGAP4	AQP1	BAHCC1
	CHRNB2	CSF1R	CBX7	COL15A1	CALD1	CALD1	DCHS1	GLI3		BRCA2	BHLHE40	ARL11	AQP3	BRCA2
	CITED2	CUL7	CELSR2	COL16A1	CBL	CARMIL2	DSP	HP		CBL	BNIP3	ARL6IP5	ARHGAP35	CACNA2D2
	CLSTN1	CUL9	CIART	COL17A1	CD109	CBL	ECE1	IRS1		CELSR2	BRCA2	ATAD2	ARHGAP4	CAPN1
	CNTFR	DDIT4	CITED1	COL1A1	CHRNB2	CD109	EGFR	ITGA1		CITED2	C8orf4	ATF3	ARL11	CBL
	CNTN5	DNMT3B	CITED2	COL1A2	CITED1	CHRNB2	EPHA3	ITGA8		CNTNAP2	CA3	ATM	ARL6IP5	CCDC86
	CNTNAP2	DOT1L	COL1A1	COL21A1	CITED2	CITED1	EPHB3	JARID2		COL11A1	CACNA2D2	ATR	ATAD2	CDC45
	COL1A1	E2F1	COL4A2	COL22A1	CLSTN1	CITED2	EPHB4	LAMC1		COL17A1	CAPN1	BCAS2	ATF3	CERK
	COL5A3	EGFR	CREB5	COL24A1	CNTFR	CLSTN1	ERMP1	LIG1		COL1A1	CBL	BHLHE40	ATM	CHRNB2
	CREM	ELK1	CREG1	COL25A1	CNTNAP2	CNTFR	FANCA	LRP4		COL4A2	CCDC86	BNIP3	ATR	CHTF18
	CSF1R	EZH2	CREM	COL27A1	COL24A1	CNTNAP2	FANCL	LZTS2		COL7A1	CD33	BRCA2	BCAS2	CITED1
	CTSB	FANCA	CYP24A1	COL4A2	COL25A1	COL24A1	FAT4	MBTD1		CSF1R	CDC45	C8orf4	BHLHE40	CITED2
	CTSD	FASN	CYP26B1	COL4A6	CREB5	COL25A1	FGFR1	MMP14		CTSB	CDH6	CA3	BNIP3	CNTFR
	CTSE	FGFR1	CYTL1	COL5A1	CREG1	CREB5	FGFR2	MNX1		CTSE	CERK	CACNA2D2	BRCA2	CNTNAP1
	CTSV	FGFR2	DAB2IP	COL5A2	CSF1R	CREG1	FHOD3	MSTN		CTSL	CERS5	CAPN1	C8orf4	COL11A1
	CUL7	FIGNL1	DBP	COL5A3	CTSB	CSF1R	GADD45G	ODC1		CTSV	CERS6	CBL	CA3	COL1A1
	CYP26B1	FOSL1	DKK3	COL7A1	CTSE	CTSB	GATA3	PRKDC		CYP26B1	CITED1	CCDC86	CACNA2D2	COL25A1
	DDR1	GADD45G	DNMT3A	COL8A1	CTSV	CTSE	GLI2	PTCH1		DAG1	CLASP1	CD33	CAPN1	COL4A2
	DKK3	GATA3	DNMT3B	COL9A3	CYB5D2	CTSV	GLI3	RFX3		DDIT4	CNTFR	CDC45	CBL	COL5A1
	DNMT3B	HMOX1	DOT1L	CSF1R	CYP26B1	CYB5D2	HEYL	SIK3		DDR1	COL1A1	CERS6	CBX7	COL5A2
	DOT1L	IRS1	E2F1	CTSB	CYTL1	CYP26B1	HMOX1	SLC9A4		DOCK10	COL25A1	CHTF18	CCDC86	COL7A1
	DSP	JARID2	EGFR	CUL9	DAB2IP	CYTL1	HP	SOCS1		DOCK4	COL4A2	CITED1	CD33	CREB5
	E2F1	JDP2	EIF2AK4	DNMT3A	DAG1	DAB2IP	HSPB8	SREBF1		DPT	COL5A3	CITED2	CDC45	CREM
	EDA	LIG1	ELK1	DNMT3B	DDIT4	DAG1	HSPG2	WNT7B		EGFR	CREM	CLASP1	CDH6	CSF1R
	EGFR	MCM2	EZH2	DPT	DDR1	DDIT4	IFT172	WT1		EGLN3	CSF1R	CNTFR	CERK	CTSB
	EIF2AK4	MLLT6	FGFR1	E2F1	DNMT3A	DDR1	IRS1	XBP1		ELK1	CTSB	COL1A1	CERS5	CTSD
	F3	MMS22L	FGFR2	EGFR	DNMT3B	DNMT3A	ITGA1	YBX2		EPHB3	CTSD	COL25A1	CERS6	CTSV
	FANCA	MYOD1	FLCN	EPHA3	DOCK4	DNMT3B	ITGA6			EPHB4	CTSV	COL4A2	CHTF18	CUL7
	FANCL	NME1	FOSL1	EPHB3	DOCK7	DOCK4	ITGA8			EZH2	CUL7	COL5A3	CITED1	CUL9
	FGFR1	PBRM1	FSTL3	EPHB6	DOT1L	DOCK7	JARID2			F3	CUL9	CREM	CITED2	CYP24A1
	FGFR2	PER1	GADD45G	EXT1	DSP	DOT1L	LAMC1			FASN	CYFIP2	CSF1R	CLASP1	CYP26B1
	FSTL3	PER3	GATA3	EZH2	E2F1	DSP	LIG1			FAT1	DAB2IP	CTSB	CLNK	CYP51A1
	GADD45G	PHC1	GFI1B	FGFR1	EGFR	E2F1	LRP4			FAT3	DAG1	CTSD	CNTFR	DAG1
	GATA3	PIK3R2	GLI2	FGFR2	EGLN3	EGFR	LZTS2			FGFR1	DDIT4	CTSE	COL1A1	DCHS1
	GLI2	PKMYT1	GLI3	FKBP10	EIF2AK4	EGLN3	MBTD1			FGFR2	DKK3	CTSL	COL25A1	DDR1
	HEYL	PLA2G10	HEYL	GATA3	ELK1	EIF2AK4	MMP14			FLCN	DNMT3A	CTSV	COL4A2	DNAJB9
	HMOX1	POLA1	HIC1	GLI2	EPHB3	ELK1	MNX1			FOSL1	DNMT3B	CUL7	COL5A3	DNMT3A
	HSPB8	PPP1R9B	IFI27	HMOX1	EPHB4	EPHB3	MSTN			GATA3	DSP	CUL9	CREM	DNMT3B
	HSPG2	PRKCA	IFT172	HP	EVC2	EPHB4	ODC1			GLI2	E2F1	CYFIP2	CSF1R	DOT1L
	IDO1	PTCH1	IKZF4	IFI44L	EXT1	EVC2	PBRM1			GLI3	EGFR	CYP26B1	CTSB	DSP
	IL21R	PTPRF	ITGA6	IGFBP4	EZH2	EXT1	PHC1			GRB7	EGLN3	DAB2IP	CTSD	E2F1
	IRS1	PTX3	JAG2	ITGA6	FASN	EZH2	PLCB1			HIC1	EIF2AK4	DAG1	CTSE	ECE1
	ITGA1	RBL1	JARID2	JAG2	FBXO32	FASN	PRKCA			HMOX1	ELK1	DDIT4	CTSL	EGFR
	ITGA6	RHOU	JDP2	LGALS1	FGFR1	FBXO32	PRKDC			HP	EPHB4	DDR1	CTSV	EGLN3
	JAG2	SEMA3F	KLF11	LIG1	FGFR2	FGFR1	PTCH1			IDO1	EPHB6	DKK3	CUL7	EHHADH
	JARID2	SESN1	LDLR	MIA	FIGNL1	FGFR2	PTPRS			IGFBP4	EPX	DNMT3A	CUL9	EIF2AK4
	LAMC1	SKI	LGALS1	MMP14	FLCN	FIGNL1	RBL1			IL21R	EZH2	DNMT3B	CYFIP2	EPHA3
	LDLR	SOCS1	LPIN1	MXRA5	FOSL1	FLCN	RFX3			IRS1	F3	DOT1L	CYP26B1	EPHB3
	LGALS1	TLR2	LRP1	NCAM1	FSTL3	FOSL1	S1PR4			ITGA1	FAM134B	DSP	DAB2IP	EPX
	LIG1	TNC	MAGI2	ODC1	GADD45G	FSTL3	SIK3			ITGA6	FANCA	E2F1	DAG1	EVC2
	LRP4	TRRAP	MED13	PBRM1	GATA3	GADD45G	SLC23A1			ITGA8	FANCL	ECE1	DDIT4	EXT1
	LTB4R	TSG101	MLLT6	PLXNB2	GFI1B	GATA3	SLC9A4			LAMA1	FASN	EDA	DDR1	EZH2
	MBTD1	TTC28	MMS19	PRKCA	GLI2	GFI1B	SOCS1			LAMB1	FBXO32	EGFR	DKK3	F3
	MMP14	WT1	MNX1	PTCH1	GLI3	GLI2	SOX18			LAMC1	FGFR1	EGLN3	DNMT3A	FANCL
	MMP19	WWC1	MSTN	RELN	GNA15	GLI3	SREBF1			LDLR	FGFR2	EIF2AK4	DNMT3B	FASN
	MNX1	XBP1	MTF2	RORB	GNAL	GNA15	TCAP			LGALS1	FOSL1	ELK1	DOT1L	FAT1
	MPO		MYO6	SLC24A3	HEYL	GNAL	TCF7			LRP1	FSTL3	EPHB4	DSP	FAT4
	MSTN		MYOD1	SLIT3	HMOX1	HEYL	TLR2			LTB4R	GADD45G	EPHB6	E2F1	FGFR1
	MYOD1		NACC2	TLR2	HSPG2	HMOX1	TYR			LTB4R2	GATA3	EZH2	ECE1	FGFR2
	NCAM1		NCAM1	TNC	IKZF4	HSPG2	WNT7B			MAGI2	GFI1B	F3	EDA	FLCN
	NEO1		NCOA1	TNNI2	IL21R	IKZF4	WT1			MCM2	GLI2	FAM134B	EGFR	FOSL1
	NLRC5		NEO1	TYR	INSIG1	IL21R	XBP1			MCM3	GLI3	FANCA	EGLN3	GATA3
	NRG2		NLRC5	UGP2	IRS1	INSIG1	YBX2			MIA	GNAL	FASN	EIF2AK4	GFI1B
	PER1		NME1	WISP2	ITGA6	IRS1	ZFPM2			MINK1	HEYL	FAT4	ELK1	GLI2
	PHC1		NME2	ZFPM2	JAG2	ITGA6	ZMIZ1			MMP14	HIC1	FBXO32	EPHB4	GNAL
	PLA2G10		NOSTRIN		JARID2	JAG2				MMP19	HMOX1	FGFR1	EPHB6	HEYL
	PLCG2		NR1D2		JDP2	JARID2				MNX1	HSPB8	FGFR2	EPX	HIC1
	POU2F2		NSD2		KIF5B	JDP2				MPO	IDO1	FIGNL1	EZH2	HMOX1

PRKDC	PAK6	LAMC1	KIF5B	NCAM1	IGFBP4	FLCN	F3	HP
PTPRF	PBRM1	LDLR	LAMC1	NEO1	IL21R	FNDC1	FANCA	HPR
PTPRS	PER1	LGALS1	LDLR	NME1	ILKAP	FOSL1	FANCL	HSD17B1
RBL1	PER3	LINGO1	LGALS1	NME2	IRS1	FSTL3	FASN	HSPB8
RELN	PHF21A	LPIN1	LINGO1	NSD2	ITGA1	GADD45G	FAT4	HSPG2
RHO	PIK3R2	LRP1	LPIN1	OSGIN1	ITGA6	GATA3	FBXO32	IDO1
SIGLEC1	PLCB1	LRP4	LRP1	PAK6	ITPR3	GFI1B	FGFR1	IFT172
SIGLEC10	POLA1	LTBP4	LRP4	PER1	LDLR	GLI2	FGFR2	INSIG1
SIGLEC8	POU2F2	MAGI2	LTBP4	PIK3R2	LGALS1	GLI3	FIGNL1	ITGA6
SIK3	PRKCA	MBNL1	MAGI2	PLA2G10	LIG1	GNAL	FLCN	ITGA8
SKI	PRKDC	MINK1	MBNL1	PLCG2	LINGO1	HEYL	FNDC1	ITPR3
SLC17A8	PTCH1	MMP14	MINK1	PLXNA2	LRP1	HIC1	FOSL1	JAG2
SLC1A3	RAD54L2	MMP19	MMP14	PLXNB1	LTB4R2	HMOX1	FSTL3	KIF26A
SLC9A4	RBL1	MNX1	MMP19	PLXNB2	LZTS2	HSPB8	GADD45G	KIF5B
SOCS1	RFX3	MSTN	MNX1	PRKCA	MCM10	HSPG2	GATA3	LAMA1
SOX13	RORB	MTF2	MSTN	PTP4A2	MCM2	IDO1	GFI1B	LAMC1
SREBF1	SATB2	MYO6	MTF2	PTPRF	MEFV	IFI27	GLI2	LDLR
ST14	SCAF8	MYOD1	MYO6	PTX3	MIA	IGFBP4	GLI3	LIAS
TET1	SEC61A1	NCAM1	MYOD1	PVR	MMP14	ILKAP	GNAL	LIG1
TLR2	SKI	NCOA1	NCAM1	RELN	MMS22L	IRS1	GRB7	LRP1
TNC	SMARCC2	NME1	NCOA1	RHO	MPHOSPH9	ITGA1	HEYL	LRP4
TP53BP1	SOCS1	NME2	NME1	RHOU	MPO	ITGA6	HIC1	MAGI2
TRPM5	SOX18	NMRK2	NME2	RNH1	MSTN	ITPR3	HMOX1	MBNL1
UNC13A	SPIN1	OCA2	NMRK2	S1PR4	MYO6	JAG2	HP	MBTD1
UPF1	SREBF1	ODC1	OCA2	SATB2	MYOD1	KLF11	HSPB8	MCM10
WNT7B	SUZ12	OGN	ODC1	SEMA3E	NACC2	LAMA1	HSPG2	MCM2
WT1	TCF7	PER3	OGN	SEMA3F	NCAM1	LDLR	IDO1	MED13
XBP1	TENM1	PHC1	PER3	SEMA4C	NCEH1	LGALS1	IFI27	MMP14
YBX2	TET1	PIK3R2	PHC1	SEMA5A	NCOA1	LIG1	IGFBP4	MNX1
ZNF521	THRAP3	PLCB1	PIK3R2	4-Sep	NECTIN2	LINGO1	IL21R	MPO
	TLR2	PLCG2	PLCB1	SFRP5	NEO1	LRP1	ILKAP	MSTN
	TNC	PLXNB2	PLCG2	SHC4	NME1	LTB4R2	IRS1	MTF2
	TNNI2	POU2F2	PLXNB2	SIGLEC8	NSD2	LZTS2	ITGA1	MTSS1
	TP53BP1	PPM1L	POU2F2	SIGLEC9	ODC1	MCM10	ITGA6	MYOD1
	TRRAP	PRKCA	PPM1L	SLC1A3	OSGIN1	MCM2	ITPR3	NALCN
	TSG101	PRKDC	PRKCA	SLC3A2	PAK6	MEFV	JAG2	NCAM1
	WNT7B	PTCH1	PRKDC	SLIT1	PER1	MIA	KLF11	NCOA1
	WT1	PTPRF	PTCH1	SLIT3	PIK3R2	MMP14	LAMA1	NRG2
	WWC1	RAPH1	PTPRF	SOCS1	PKMYT1	MNX1	LDLR	PHC1
	XBP1	RBL1	RAPH1	SPAG9	PLCB1	MPO	LGALS1	PHEX
	YBX2	RELN	RBL1	SREBF1	PLCG2	MSTN	LIG1	PHF21A
	ZFPM2	RLTPR	RELN	SRGAP1	PLXNB1	MYO6	LINGO1	PLA2G10
	ZMIZ1	RNH1	RNH1	STC1	PPP1R9B	MYOD1	LRP1	PLCB1
	ZNF536	RORB	RORB	SUZ12	PRKCA	NACC2	LTB4R2	PLCG2
		S1PR4	S1PR4	TAX1BP1	PRKDC	NCAM1	LZTS2	PLXNB2
		SALL3	SALL3	TLR2	PTCH1	NCEH1	MCM10	POLG
		SATB2	SATB2	TLR7	PTPRF	NCOA1	MCM2	POU2F2
		SCUBE3	SCUBE3	TNC	PTPRR	NECTIN2	MEFV	PRKCA
		SDK2	SDK2	WISP2	RBL1	NME1	MIA	PRKDC
		SEMA3E	SEMA3E	WWC1	S1PR4	NME2	MMP14	PTCH1
		SEMA3F	SEMA3F	ZFAND5	SEMA3F	NRG2	MMS22L	PTPRF
		SEMA4C	SEMA4C		4-Sep	NSD2	MNX1	PTPRS
		SEMA5A	SEMA5A		SH3PXD2A	ODC1	MPHOSPH9	PTX3
		SH3PXD2A	SH3PXD2A		SIGLEC5	OSGIN1	MPO	RBL1
		SHC4	SHC4		SIGLEC8	PAK6	MSTN	RFX3
		SIGLEC10	SIGLEC10		SIGLEC9	PDE3A	MYO6	RGMA
		SIGLEC8	SIGLEC8		SKI	PER1	MYOD1	RIF1
		SIK3	SIK3		SLC1A3	PIK3R2	NACC2	RPL24
		SKI	SKI		SLC25A38	PKMYT1	NCAM1	SALL3
		SLC1A3	SLC1A3		SLC3A2	PLCG2	NCEH1	SATB2
		SLC25A38	SLC25A38		SLC9A4	PPP1R9B	NCOA1	SEMA5A
		SLC3A2	SLC3A2		SLIT1	PRKCA	NECTIN2	SFRP5
		SLIT1	SLIT1		SLIT3	PRKDC	NEO1	SGK223
		SNX19	SNX19		SMOX	PTCH1	NME1	SH3PXD2A
		SOCS1	SOCS1		SOCS1	PTP4A2	NME2	SIK3
		SPAG9	SPAG9		SPIN1	PTPRF	NRG2	SKI
		SPON1	SPON1		SREBF1	RBL1	NSD2	SLC1A3
		SREBF1	SREBF1		ST14	RHO	ODC1	SLC23A1
		SRGAP1	SRGAP1		STC1	S1PR4	OSGIN1	SLC3A2
		SRGAP2	SRGAP2		SVIL	SEMA3F	PAK6	SMARCC2
		ST14	ST14		TAX1BP1	4-Sep	PDE3A	SOCS1
		STC1	STC1		TCF7	SFRP5	PER1	SREBF1
		SUZ12	SUZ12		TLR2	SHC4	PIK3R2	SRGAP3
		SV2A	SV2A		TLR7	SIGLEC5	PKMYT1	ST14
		TDO2	TDO2		TNC	SIGLEC8	PLA2G10	SUZ12
		TET1	TET1		TP53BP1	SIGLEC9	PLCB1	SV2A
		THRAP3	THRAP3		TSG101	SKI	PLCG2	SYNJ1
		TLR2	TLR2		TTR	SLC1A3	PLXNB1	TAX1BP1
		TLR7	TLR7		TYR	SLC25A38	PPP1R9B	TENM3
		TMBIM1	TMBIM1		WNK3	SLC9A4	PRKCA	TLR2
		TMEM120A	TMEM120A		WT1	SMOX	PRKDC	TLR7
		TNC	TNC		XBP1	SOCS1	PTCH1	TNXB
		TPM4	TPM4			SREBF1	PTP4A2	TP53BP1
		TSG101	TSG101			ST14	PTPRF	TRRAP
		WDR7	WDR7			STC1	PTPRR	TSG101
		WISP2	WISP2			SUZ12	PVR	UNC13A
		WNT7B	WNT7B			TAX1BP1	RBL1	UNC79

WT1	WT1	TLR2	RETREG1	UPF1
XBP1	XBP1	TLR7	RHO	WNT7B
ZFPM2	ZFPM2	TNC	S1PR4	WT1
ZNF521	ZNF521	TP53BP1	SEMA3F	XBP1
ZNF536	ZNF536	TSG101	4-Sep	ZFPM2
		TTR	SFRP5	ZMIZ1
		UPF1	SH3PXD2A	
		WNK3	SHC4	
		WT1	SIGLEC5	
		XBP1	SIGLEC7	
		YBX2	SIGLEC8	
		ZFAND5	SIGLEC9	
			SKI	
			SLC1A3	
			SLC25A38	
			SLC3A2	
			SLC9A4	
			SLIT1	
			SLIT3	
			SMOX	
			SOCS1	
			SPIN1	
			SREBF1	
			ST14	
			STC1	
			SUZ12	
			SVIL	
			TAX1BP1	
			TCF7	
			TLR2	
			TLR7	
			TNC	
			TP53BP1	
			TSG101	
			TTR	
			TYR	
			UPF1	
			WNK3	
			WT1	
			XBP1	
			YBX2	
			ZFAND5	
			ZFPM2	
