# Supplementary material for: Waveband specific transcriptional control of select genetic pathways in vertebrate skin (Xiphophorus maculatus)
Source: BMC Genomics. 2018 May 10;19:355. doi: 10.1186/s12864-018-4735-5 (PMC5946439; doi:10.1186/s12864-018-4735-5)
Supplement: Supplementary file 4 — Table S4a–k. A list of all differentially modulated genes used by IPA enrichment software to predict the direction of change for each functional class represented in Fig. 4. Table a is FL, tables b–e are the 50 nm wavebands and tables g–k are the 10 nm wavebands. (ZIP 262 kb) [file 12864_2018_4735_MOESM4_ESM.zip › TableS4g_500-510nm.pdf]

**Function**	benign neopl	differentiatio	differentiatio	differentiatio	organismal death
**z-score**	2.227	-2.142	2.089	2.173	2.12
**number of ge**	23	51	9	20	54
**molecules**	AGRN	ADAMTS20	COL11A1	AGT	AGRN
	ANXA5	AGRN	CYP1A1	ALOXE3	AGT
	APC	AGT	CYP1A2	APC	ALOX12B
	ATR	ALOX15B	DHCR24	EPHB4	ALOXE3
	ATRN	ALOXE3	GLI2	FASN	APC
	COL11A1	APC	JDP2	FSTL3	ATR
	COL18A1	ARHGAP32	MST1	GATA3	BIRC6
	COL4A6	COL18A1	PTHLH	GLI2	CAPN1
	COL7A1	CYB5D2	WT1	HMOX1	CERK
	CUL9	DDIT4		INSIG1	COL11A1
	EPHB3	DOT1L		ISG15	COL7A1
	FAM57A	EBP		JDP2	CUL7
	FKBP10	EPHB3		LAMB3	CUL9
	GATA3	EPHB4		LRP6	CYP1A1
	GLI2	EXTL1		MSTN	CYP1A2
	HMGCR	FASN		PTHLH	CYP51A1
	HMOX1	FSTL3		SIK3	DOT1L
	HP	GATA3		SREBF1	EPHB3
	KMT2C	GLI2		TNC	FASN
	LAMB3	GON4L		WT1	FAT4
	TG	HMOX1			GATA3
	TNC	HSPA5			GCK
	TSC1	INSIG1			GLI2
		ISG15			HMGCR
		JARID2			HMOX1
		JDP2			HP
		KALRN			HPR
		LAMB3			HSPA5
		LRP6			INSIG1
		MNX1			ISG15
		MST1			KMT2C
		MSTN			LIAS
		NCOA1			LRP6
		NMRK2			MCM3AP
		NUP98			MED13
		PER3			MNX1
		PLXNA4			MSTN
		PTHLH			NCOA1
		PTPRF			NUP98

RRAD
SEMA5A
SHC4
SIK3
SREBF1
SUZ12
TG
TMBIM1
TNC
TSC1
UBR2
WT1

PER2
PHF21A
PIK3R4
PTHLH
PTPRF
RPL24
SEMA5A
SIK3
SLC4A1
SREBF1
SUZ12
TRRAP
TSC1
UBR2
WT1
