# Supplementary material for: Waveband specific transcriptional control of select genetic pathways in vertebrate skin (Xiphophorus maculatus)
Source: BMC Genomics. 2018 May 10;19:355. doi: 10.1186/s12864-018-4735-5 (PMC5946439; doi:10.1186/s12864-018-4735-5)
Supplement: Supplementary file 4 — Table S4a–k. A list of all differentially modulated genes used by IPA enrichment software to predict the direction of change for each functional class represented in Fig. 4. Table a is FL, tables b–e are the 50 nm wavebands and tables g–k are the 10 nm wavebands. (ZIP 262 kb) [file 12864_2018_4735_MOESM4_ESM.zip › TableS4f_550-600nm.pdf]

Function	quantity of c	cell proliferat	generation o	differentiatio	angiogensis	vasculogenes	migration of	inflammatior	organismal death
z-score	-2.653	3.50	-2.325	-2.501	-2.049	-2.354	-2.633	-3.86	4.53
number of ge	36	72	44	44	27	22	45	11	49
molecules	AGRN	ADAMTS7	ADAMTS2	ADAMTS20	ABRA	ABRA	ADAMTS7	ATRN	AGRN
	ALS2	ALS2	ADAMTS7	ADAMTS7	ADAMTS2	ADAMTS2	BCAS3	COL11A1	BIRC6
	ARNTL2	AXIN2	AGRN	AGRN	BIRC6	CD151	CD151	COL1A1	BPTF
	BIRC6	BIRC6	ALS2	ARHGAP32	CD151	COL15A1	CELSR2	COL1A2	CASZ1
	CACNA1D	BPTF	ARHGAP32	AXIN2	COL15A1	COL1A1	CNTNAP2	DENND4B	CD151
	CD151	CACNA1D	AXIN2	CACNA1D	COL1A1	COL4A1	COL11A1	ENO2	CDON
	CNTNAP2	CASZ1	CACNA1D	CASZ1	COL1A2	CUL7	COL17A1	EPHB3	COL11A1
	COL1A1	CD151	CD151	CDON	COL4A1	EGR3	COL1A1	LRBA	COL12A1
	COL5A3	CDON	CELSR2	CNTNAP2	COL5A1	EPHB3	COL4A1	SLIT3	COL1A1
	CUL7	CELSR2	CNTNAP2	COL24A1	CUL7	EPHB4	COL7A1	TAX1BP1	COL4A1
	DNM1	CLEC19A	COL11A1	COL4A1	EGR3	FLNA	DOCK4	XIRP1	COL5A1
	DOT1L	CNTNAP2	COL1A1	DOCK4	EPHB3	GLI3	EPHB3		COL7A1
	EGR3	COL11A1	COL4A1	DOT1L	EPHB4	HSPG2	EPHB4		CUL7
	FLNA	COL12A1	CUL7	EGR3	FLNA	INS	FASN		DNM1
	GCG	COL15A1	EGR3	EPHB3	GLI3	NCOA1	FAT1		DOT1L
	GDF6	COL1A2	EPHB3	EPHB4	HSPG2	PLXNB1	FAT3		EGR3
	HMGA1	COL21A1	EPHB4	FASN	INS	PRKDC	FLNA		EPHB3
	HSPB8	COL24A1	FLNA	GCG	NCOA1	PTPRB	GLI3		FASN
	HSPG2	COL4A1	GCG	GDF6	PKD1	SLIT3	INS		FAT1
	INS	COL5A3	GLI3	GLI3	PLXNB1	SST	LAMB1		FLNA
	JARID2	COL6A3	HERC1	HMGA1	PRKDC	TAB2	LAMB3		GCG
	JMJD1C	DENND4B	HERC2	HSPG2	PTPRB	WNK1	LRP6		GDF6
	KLF13	DMXL2	HMGA1	INS	RAMP1		MAGI1		HSPB8
	LRP6	DNM1	HSPG2	JARID2	SLIT3		MAGI2		HSPG2
	MKL1	DOCK4	JMJD1C	JMJD1C	SST		MKL1		INS
	NDST3	EGR3	LAMB1	KLF13	TAB2		NDST3		KLHL40
	NEO1	FASN	LRP6	KLHL41	WNK1		NEO1		LRP6
	PER2	FAT1	MAGI2	LAMB3			PCOLCE2		MAGI2
	PKD1	FAT3	MKL1	LRP6			PKD1		MCM3AP
	PRKDC	FBRS	NCOA1	MAGI2			PLXNA1		MKL1
	PTPRF	FBRSL1	PARD3	MKL1			PLXNB1		MRC1
	PTPRS	GDF6	PKD1	NCOA1			PTPRF		NCOA1
	RELN	GLI3	PLXNB1	PKD1			RAMP1		NDST3
	RERE	HERC2	PRKDC	PRKDC			RELN		PARD3
	SST	HMGA1	PTPRF	PRRC2C			RERE		PER2
	TG	HSPG2	RELN	PTPRF			SEMA4C		PHF21A
		JARID2	RERE	RELN			SHC4		PKD1
		KLF11	SLIT3	RRAD			SLIT3		POLR2A
		KLHL30	SST	SEMA4C			SST		PRKDC
		KLHL41	TAB2	SHC4			STK35		PTPRF
		LRBA	TCAP	SUZ12			SUZ12		PTPRS
		MAGI1	TNIK	TG			TAB2		SMG1
		MAGI2	WNK1	TGM1			TAX1BP1		SUZ12
		MRC1	ZDHHC8	ZDHHC8			TG		TAB2
		MURC					ZNF703		TAX1BP1
		NAV3							TENM3
		NCOA7							TGM1
		NHSL1							TRRAP
		PARD3							UNC79
		PCOLCE2							
		PHF21A							
		PKD1							
		PLXNB1							
		PRKDC							
		PRR12							
		PTPRF							
		PTPRS							

RELN
SCN8A
SLIT3
STK35
SUZ12
SVEP1
SVIL
TAX1BP1
TNIK
TNRC18
TRRAP
TTC28
UNC79
VPS13B
ZNF668
