# Supplementary material for: Waveband specific transcriptional control of select genetic pathways in vertebrate skin (Xiphophorus maculatus)
Source: BMC Genomics. 2018 May 10;19:355. doi: 10.1186/s12864-018-4735-5 (PMC5946439; doi:10.1186/s12864-018-4735-5)
Supplement: Supplementary file 4 — Table S4a–k. A list of all differentially modulated genes used by IPA enrichment software to predict the direction of change for each functional class represented in Fig. 4. Table a is FL, tables b–e are the 50 nm wavebands and tables g–k are the 10 nm wavebands. (ZIP 262 kb) [file 12864_2018_4735_MOESM4_ESM.zip › TableS4e_500-550nm.pdf]

**Function**	transcription	developmen	hypertrophy	fibrosis	inflammation
**z-score**	2.744	-2	-2.368	-2.664	-2.14
**number of ge**	33	30	17	8	8
**molecules**	ABRA	ALPK3	ANKRD1	ACSL1	C4A/C4B
	ANKRD1	ANKRD1	CSRP3	CSRP3	CALCA
	ARID1A	ARID1A	FBXO32	HMOX1	DGKZ
	BHLHE40	CAPN3	HMOX1	HSPB8	FBXO32
	CALCA	COL11A1	HSPB8	MAPK8	GATA3
	CALCOCO1	CSRP3	JARID2	PFKFB1	HMOX1
	CAPN3	CYP1A2	MAPK8	RERE	MAPK8
	CELSR2	DHRS3	MYOM1	XIRP1	MTMR4
	CREB5	FHL1	NR4A3		
	CSRP3	GATA3	PFKFB1		
	DNAJB5	HMOX1	PFKM		
	DUSP22	HSPB11	PPARGC1A		
	DUSP26	HSPB8	RRAD		
	GATA3	JARID2	TCAP		
	HELT	LMOD2	TRIM55		
	HIPK3	MAPK8	TRIM63		
	HIVEP1	MYF6	XIRP1		
	JARID2	PKM			
	MAPK8	PPARGC1A			
	MED13	PRKDC			
	MYF6	PTPRF			
	NR4A3	SIK3			
	PER3	TCAP			
	POLR2A	TLX1			
	PPARGC1A	TNNI1			
	PRKDC	TP53BP2			
	SP4	TRIM55			
	TAF3	TRIM63			
	TLX1	XIRP1			
	USP13	XIRP2			
	VGLL2				
	ZBTB16				
	ZNF703				
