# Supplementary material for: Waveband specific transcriptional control of select genetic pathways in vertebrate skin (Xiphophorus maculatus)
Source: BMC Genomics. 2018 May 10;19:355. doi: 10.1186/s12864-018-4735-5 (PMC5946439; doi:10.1186/s12864-018-4735-5)
Supplement: Supplementary file 4 — Table S4a–k. A list of all differentially modulated genes used by IPA enrichment software to predict the direction of change for each functional class represented in Fig. 4. Table a is FL, tables b–e are the 50 nm wavebands and tables g–k are the 10 nm wavebands. (ZIP 262 kb) [file 12864_2018_4735_MOESM4_ESM.zip › TableS4d_450-500nm.pdf]

**Function**	quantity of c	cell proliferat	generation o	cell viability	differentiatic	angiogensis	vasculogenes	development	development	migration of	migration of	adhesion of c	inflammatior	necrosis	apoptosis	stress respor	cell death	organismal death
**z-score**	2.198	3.15	2.26	2.25	3.022	2.85	2.853	2.38	2.461	2.406	2.699	2.41	2.88	2.50	-2.04	2	2.563	-2.92
**number of g**	27	40	21	54	24	19	14	17	10	6	25	5	19	37	34	6	41	32
**molecules**	ADAM8	ADAM8	ANGPT2	ADAM8	ADAM8	ADAM8	ANGPT2	ANKRD1	CEBPB	ANGPT2	ADAM8	ANGPT2	ADAM8	ADAM8	ADAM8	ANGPT2	ADAM8	ACSS1
	ANGPT2	ANGPT2	C5AR1	ANGPT2	ANGPT2	ANGPT2	BCL6B	ATP2A2	CSF3	IL1B	ANGPT2	IL1B	ANGPT2	ANGPT2	ANGPT2	ATP2A2	ANGPT2	ANGPT2
	BCL6B	ANKRD1	CEBPB	ANKRD1	ATP2A2	ARHGEF4	C5AR1	CEBPB	GGT1	KDR	ARHGEF4	LDLR	C5AR1	ANKRD1	ANKRD1	CEBPB	ANKRD1	ATP2A2
	C5AR1	ARHGEF4	CSF3	ARG2	C5AR1	BCL6B	CSF3	CSF3	HP	LDLR	C5AR1	MME	CEBPB	ARG2	ARG2	HSP90B1	ARG2	C5AR1
	CEBPB	ATP2A2	GGT1	ATP2A2	CEBPB	C5AR1	IL1B	GGT1	IL1B	MMP9	CEBPB	TIMP2	CSF3	ATP2A2	BCL6B	IL1B	ATP2A2	CA4
	CSF3	BCL6B	GNAT1	BCL6B	CREB5	CSF3	KDR	HP	KDR	TLR2	CSF3		GGT1	BCL6B	C5AR1	MMP9	BCL6B	CEBPB
	GGT1	C5AR1	HSP90B1	C5AR1	CSF3	HP	LTB4R	IL1B	MMP9		HP		HP	C5AR1	CA4		C5AR1	CREB5
	GNAT1	CCDC169	IL1B	CA4	HSP90B1	IL1B	MMP9	KDR	RIPK3		HSP90B1		IL1B	CA4	CEBPB		CA4	CSF3
	HSP90B1	CEBPB	KDR	CEBPB	IGFBP6	KDR	PFKFB3	MMP9	SALL1		HYOU1		KDR	CEBPB	CSF3		CEBPB	CYP24A1
	IGFBP6	CH25H	LDLR	CSF3	IL1B	LTB4R	PTAFR	RIPK3	TIMP2		IGFBP6		LDLR	CSF3	GCK		CSF3	F8
	IL1B	CREB5	MMP9	CYP24A1	KDR	MMP9	PTX3	S1PR4			IL1B		LTB4R	DENND4A	GGT1		DENND4A	GCK
	KDR	CSF3	PFKFB3	DENND4A	LDLR	PFKFB3	RGS5	SALL1			KDR		MMP9	GCK	GNAT1		GCK	GGT1
	LDLR	CYP24A1	RGS5	DNAH7	MME	PTAFR	TGM2	TGM2			LDLR		PTAFR	GGT1	HSH2D		GGT1	HP
	LTB4R	DENND4A	RHO	F13B	MMP9	PTX3	TIMP2	THRSP			LTB4R		PTX3	HCAR1	HSP90B1		GNAT1	HSD17B1
	MME	DNAH7	RIPK3	F8	OCSTAMP	RGS5		TIMP2			MME		RIPK3	HSH2D	HYOU1		HCAR1	HSP90B1
	MMP9	F13B	SLC11A2	GCK	RIPK3	S1PR4		TLR2			MMP9		TGM2	HSP90B1	IGFBP6		HP	HYOU1
	PTAFR	F8	SOAT1	GGT1	S1PR4	TGM2		XIRP2			PTAFR		TLR2	HYOU1	IL1B		HSH2D	IL1B
	RHO	GCK	TIMP2	GNAT1	SALL1	TIMP2					PTX3		TLR5	IGFBP6	KDR		HSP90B1	KDR
	RIPK3	GGT1	TLR2	HCAR1	TGM2	TLR2					RHO		TNFRSF9	IL1B	LDLR		HYOU1	LDLR
	SLC11A2	GNAT1	TLR5	HP	TIMP2						S1PR4			KDR	MME		IGFBP6	MME
	SOAT1	HP	TNFRSF9	HSD17B1	TLR2						TGM2			LDLR	MMP9		IL1B	MMP9
	STEAP4	HSD17B1		HSH2D	TLR5						TIMP2			MME	NACC2		KDR	PFKFB3
	TGM2	HSP90B1		HSP90B1	TNFRSF9						TLR2			MMP9	PTAFR		LDLR	PTAFR
	TIMP2	HYOU1		HYOU1	TYRP1						TLR5			NACC2	RGS5		MME	PTX3
	TLR2	IGFBP6		IGFBP6							TNFRSF9			PFKFB3	RHO		MMP9	RIPK3
	TLR5	IL1B		IL1B										PTAFR	RIPK3		NACC2	SALL1
	TNFRSF9	IL1R2		KDR										RGS5	S1PR4		PFKFB3	SLC11A2
		KDR		LDLR										RIPK3	SALL1		PTAFR	TGM2
		LDLR		MME										S1PR4	SOAT1		RGS5	TIMP2
		LPAR5		MMP9										SALL1	TGM2		RHO	TLR2
		MME		NACC2										SLC11A2	TIMP2		RIPK3	TLR5
		MMP9		OCSTAMP										SOAT1	TLR2		S1PR4	TNFRSF9
		SLC11A2		PFKFB3										TGM2	TNFAIP2		SALL1	
		SOAT1		PTAFR										TIMP2	TNFRSF9		SLC11A2	
		TGM2		RGS5										TLR2			SOAT1	
		TIMP2		RHO										TNFRSF9			TGM2	
		TLR2		RIPK3										TYRP1			TIMP2	
		TLR5		S1PR4													TLR2	
		TNFRSF9		SALL1													TNFAIP2	
		TYRP1		SLC11A2													TNFRSF9	
				SOAT1													TYRP1	
				SPSB4														
				SQLE														
				STEAP4														
				TGM2														
				THRSP														
				TIMP2														
				TLR2														
				TNFAIP2														
				TNFRSF9														
				TYRP1														
				VEPH1														
				VWA5A														
				XIRP2														
