# Supplementary material for: Waveband specific transcriptional control of select genetic pathways in vertebrate skin (Xiphophorus maculatus)
Source: BMC Genomics. 2018 May 10;19:355. doi: 10.1186/s12864-018-4735-5 (PMC5946439; doi:10.1186/s12864-018-4735-5)
Supplement: Supplementary file 4 — Table S4a–k. A list of all differentially modulated genes used by IPA enrichment software to predict the direction of change for each functional class represented in Fig. 4. Table a is FL, tables b–e are the 50 nm wavebands and tables g–k are the 10 nm wavebands. (ZIP 262 kb) [file 12864_2018_4735_MOESM4_ESM.zip › TableS4c_400-450nm.pdf]

**Function**	cell viability	cell death
**z-score**	2.00	2.12
**number of ge**	5	5
**molecules**	HMOX1	HMOX1
	KCNJ12	KCNJ12
	NR4A3	NR4A3
	PPARGC1A	PPARGC1A
	YBX2	YBX2
