# Supplementary material for: Waveband specific transcriptional control of select genetic pathways in vertebrate skin (Xiphophorus maculatus)
Source: BMC Genomics. 2018 May 10;19:355. doi: 10.1186/s12864-018-4735-5 (PMC5946439; doi:10.1186/s12864-018-4735-5)
Supplement: Supplementary file 4 — Table S4a–k. A list of all differentially modulated genes used by IPA enrichment software to predict the direction of change for each functional class represented in Fig. 4. Table a is FL, tables b–e are the 50 nm wavebands and tables g–k are the 10 nm wavebands. (ZIP 262 kb) [file 12864_2018_4735_MOESM4_ESM.zip › TableS4b_350-400nm.pdf]

Function	cell viability	ingestion	differentiatio	differentiatic	inflammatior	necrosis	apoptosis	cell death	organismal death
z-score	-2.02	2.387	-2.402	-2.213	-2.35	-2.78	2.05	-2.501	-2.67
number of ge	22	8	13	8	24	33	16	8	37
molecules	ANXA1	ARNTL	ATM	ARNTL	ANXA1	ANXA1	ANXA1	ATM	ANXA1
	ATM	ATM	BMPR1B	BMPR1B	ARNTL	ATM	ATM	CA3	ARNTL
	BMPR1B	ATP1B2	CA3	CLEC3B	ATM	BMPR1B	BMPR1B	HMOX1	ATM
	CTBP2	HOMER2	CTBP2	CYP1A1	ATP1B2	CDC34	CDC34	ITPR1	BMPR1B
	CXCL12	NPPC	FAAH	CYP1A2	CLIC3	CTBP2	CXCL12	MLKL	CDC34
	CYP1A1	NR4A3	GALNT3	LPIN1	CXCL12	CXCL12	EZR	RAB25	CTBP2
	ELOVL7	PER1	GNPNAT1	PER1	DENND3	DNAJB1	GALNT3	SLC9A3R2	CXCL12
	EMP1	PER2	HMOX1	PPARGC1A	EZR	DPM3	IRF1	TGM1	DNAJB1
	ETV7		ITPR1		ITPR1	DSG2	ITPR1		DPM3
	EZR		MLKL		MAP2K6	EMP1	MAP2K6		DSG2
	FOXQ1		RAB25		MYO5B	EZR	NCEH1		EMP1
	GNA15		SLC9A3R2		NCEH1	GALNT3	NR4A3		EZR
	IDH2		TGM1		NIPA2	GALNT5	PDGFC		GALNT3
	IRF1				NPPC	GNPNAT1	PPARGC1A		GALNT5
	ITPR1				NR4A3	IDH2	STAP2		GNPNAT1
	LRRN1				OSBPL2	IRF1	WNT5A		HUNK
	MAP2K6				PAX6	ITPR1			IDH2
	MYH14				PDYN	MAP2K6			IRF1
	PDGFC				PER2	MLKL			ITPR1
	SOX2				PPARGC1A	NCEH1			MAP2K6
	STAP2				RANGAP1	NR4A3			MLKL
	WNT5A				RSAD2	PAX6			NCEH1
					SEC23B	PDGFC			NR4A3
					SLC9A3R2	PLEKHF1			PAX6
						PPARGC1A			PDGFC
						RAB25			PDYN
						SIGIRR			PLEKHF1
						SLC9A3R2			PPARGC1A
						SOX2			RAB25
						STAP2			SEC23B
						TGM1			SIGIRR
						TMEM173			SLC9A3R2
						WNT5A			SOX2
									STAP2
									TGM1
									TMEM173
									WNT5A
