# Supplementary material for: Waveband specific transcriptional control of select genetic pathways in vertebrate skin (Xiphophorus maculatus)
Source: BMC Genomics. 2018 May 10;19:355. doi: 10.1186/s12864-018-4735-5 (PMC5946439; doi:10.1186/s12864-018-4735-5)
Supplement: Supplementary file 4 — Table S4a–k. A list of all differentially modulated genes used by IPA enrichment software to predict the direction of change for each functional class represented in Fig. 4. Table a is FL, tables b–e are the 50 nm wavebands and tables g–k are the 10 nm wavebands. (ZIP 262 kb) [file 12864_2018_4735_MOESM4_ESM.zip › TableS4a_FL.pdf]

**Function**	cell proliferat	cell viability	inflammatior	necrosis	apoptosis	cell death	organismal death
**z-score**	-5.98	-5.08	2.02	4.06	4.15	5.03	7.87
**number of g**	176	78	8	108	135	172	128
**molecules**	ABCC5	AGTPBP1	DHFR	AIMP1	ADRB2	ABCC5	ADRB2
	ADRB2	AIMP1	FBXO32	ALS2	AIMP1	ADRB2	AGL
	AIMP1	ALS2	POLE	ASNS	ALS2	AGTPBP1	ARNTL
	ALS2	ANLN	POLE2	ATAD2	ASNS	AIMP1	ATM
	ARNTL	ASCC3	RRM2	ATM	ATAD2	ALS2	AURKA
	ASCC3	ASNS	SLC2A4	AURKA	ATM	ARNTL	AURKB
	ASNS	ATM	TOP2A	AURKB	AURKA	ASNS	BARD1
	ASPM	AURKA	TYMS	BARD1	AURKB	ATAD2	BRCA1
	ATAD2	AURKB		BHLHE40	BARD1	ATM	BRCA2
	ATM	BARD1		BRCA1	BHLHE40	AURKA	BRIP1
	AURKA	BHLHE40		BRCA2	BRCA1	AURKB	BUB1
	AURKB	BRCA1		BUB1	BRCA2	BARD1	BUB1B
	BARD1	BRCA2		BUB1B	BUB1	BHLHE40	CCNA2
	BHLHE40	BRIP1		CABLES1	CABLES1	BRCA1	CCNB2
	BHMT	BUB1B		CASC5	CASC5	BRCA2	CCNF
	BRCA1	CCNA2		CCNB1	CCNA2	BUB1	CDC20
	BRCA2	CCNB1		CDC20	CCNB1	BUB1B	CDC45
	BRIP1	CDK1		CDC6	CDC20	C7	CDK1
	BUB1	CENPA		CDK1	CDC25B	CABLES1	CENPE
	BUB1B	CKAP5		CDT1	CDC45	CASC5	CENPJ
	CABLES1	CLOCK		CENPE	CDC6	CCNA2	CENPU
	CCNA2	CYR61		CENPI	CDK1	CCNB1	CHAF1A
	CCNB1	DCK		CKAP5	CENPA	CDC20	CHTF18
	CCNB2	DNMT1		CLSPN	CENPE	CDC25B	CIT
	CCNF	E2F1		CLYBL	CENPF	CDC45	CKAP5
	CDC20	EGR3		CYR61	CENPJ	CDC6	CKS2
	CDC25B	FANCD2		DCK	CHTF18	CDK1	COL11A1
	CDC45	FASN		DCTD	CIT	CDT1	CPT1A
	CDC6	FBXO32		DHCR24	CKAP5	CENPA	CYR61
	CDCA7	FBXO5		DHFR	CTNNBL1	CENPE	DACT1
	CDCA8	FKBP5		DNMT1	CYR61	CENPF	DDR1
	CDK1	FOSL1		DTL	DCK	CENPI	DIAPH3
	CDT1	FOXM1		DTYMK	DDR1	CENPJ	DNMT1
	CENPA	HAP1		E2F1	DEPTOR	CHTF18	DNMT3B
	CENPF	HMGA1		EGR3	DHCR24	CIT	DTL
	CENPJ	HMOX1		EPHB6	DHFR	CKAP5	E2F1
	CKS2	KIF18A		EYS	DNMT1	CLOCK	E2F8
	CLOCK	KIFC1		FANCL	DNMT3B	CLSPN	ECT2
	CLSPN	KLF5		FASN	DTYMK	CLYBL	EGR3
	CLYBL	KLF6		FBXO32	E2F1	CTNNBL1	EHHADH
	CYR61	LIG1		FBXO5	E2F8	CYR61	ESPL1
	DCK	MAD2L1		FKBP5	ECT2	DCK	EXO1
	DDR1	MAP2K6		FOSL1	EGR3	DCTD	FANCD2
	DEPTOR	METAP2		GMDS	EPHB6	DDR1	FANCL
	DHCR24	MMS22L		HAP1	ESPL1	DEPTOR	FASN
	DHFR	MYBL2		HAUS8	EXO1	DHCR24	FBXO5

DLGAP5	MYC	HCAR1	FASN	DHFR	FOSL1
DNMT1	NDC80	HMGA1	FBXO32	DNMT1	FOXM1
DNMT3B	NFIL3	HMMR	FBXO5	DNMT3B	G2E3
DTL	NR4A3	HMOX1	FIGNL1	DTL	GGT5
DTYMK	NUF2	INCENP	FKBP5	DTYMK	GINS4
E2F1	PALB2	KIF14	FOSL1	E2F1	HAP1
E2F8	PBK	KLF5	G2E3	E2F8	HELLS
EBI3	PCNA	KLF6	GMDS	ECT2	HMOX1
ECT2	PLK1	KPNA2	HAP1	EGR3	HSD17B4
EGR3	PRKCA	LIG1	HAUS1	EMP3	HSP90AA1
EMP3	PRKCB	MAD2L1	HELLS	EPHB6	IL12B
EPHB6	PRKDC	MAN2C1	HMGA1	ESPL1	INCENP
ESPL1	RAD51	MAP2K6	HMMR	EXO1	KCNK2
EXOC4	RAD52	MAT2A	HMOX1	EYS	KIF22
FAM83D	RAD54B	MCM10	HSP90AA1	FAAH	KLF5
FANCD2	RAD54L	MDC1	IL12B	FANCD2	KLF6
FANCL	RPA1	MELK	KIF14	FANCL	LIG1
FASN	RRM2	MMS22L	KIF18A	FASN	LPL
FBXO32	SHMT2	MSTN	KLF5	FBXO32	MAD2L1
FIGNL1	SKA1	MTFP1	KLF6	FBXO5	MASTL
FKBP5	SLC2A4	MYBL2	KPNA2	FIGNL1	MCM10
FOSL1	STMN1	MYC	LIG1	FKBP5	MCM2
FOXM1	SUV39H1	NASP	MAD2L1	FOSL1	METAP2
FUT9	TGM2	NDC80	MAN2C1	FOXM1	MIS18A
GGT5	TONSL	NFE2L1	MAP2K6	G2E3	MKI67
HAP1	TSC22D3	NFIL3	MAT2A	GMDS	MSH6
HCAR1	TTK	NFIX	MCM10	HAP1	MSTN
HELLS	TYMS	NIF3L1	MCM2	HAUS1	MTF2
HMGA1	UHRF1	NPM1	MCM8	HAUS8	MTR
HMMR	UNG	NUF2	MDC1	HCAR1	MUTYH
HMOX1	WWOX	ODC1	MELK	HELLS	MYBL2
HP1BP3	XRCC4	PBK	MIS18A	HMGA1	MYC
HPX		PCNA	MKI67	HMMR	MYH9
HSP90AA1		PER1	MSH6	HMOX1	NASP
IL12B		PHGDH	MSTN	HSD17B4	NCAPG2
KALRN		PKMYT1	MTFP1	HSP90AA1	NCAPH
KCNK2		PLK1	MUTYH	IL12B	NCAPH2
KIF14		PLK4	MYBL2	INCENP	NDC80
KIF15		PRKCA	MYC	KCNK2	NFE2L1
KIF18A		PRKCB	NASP	KIF14	NFIL3
KIF20A		PRKDC	NCAPG2	KIF18A	NFIX
KIF20B		PTTG1	NCAPH2	KLF5	NPM1
KIF23		RACGAP1	NDC80	KLF6	NR4A3
KIF2C		RAD51	NET1	KPNA2	NUMA1
KLF5		RNASEH2A	NFE2L1	LIG1	NUSAP1
KLF6		RPA1	NFIL3	MAD2L1	OAT
KPNA2		RPA2	NPM1	MAN2C1	PALB2
LIG1		RRM2	NR4A3	MAP2K6	PCNA
MAD2L1		SLC29A2	NUF2	MAT2A	PER2

MAP2K6	SPAG5	NUMA1	MCM10	PHGDH
MCM10	STMN1	NUSAP1	MCM2	PLA2G10
MCM2	TACC3	ODC1	MCM8	PLK4
MCM3	TGM2	ORC2	MDC1	POLD1
MCM4	TOP2A	PALB2	MELK	POLG
MCM5	TPX2	PBK	METAP2	PRKCA
MCM8	TRAIP	PCNA	MIS18A	PRKDC
MELK	TSC22D3	PER1	MKI67	PTTG1
METAP2	TTK	PHGDH	MMS22L	RAD51
MKI67	TYMS	PKMYT1	MSH6	RAD52
MSTN	UBE2C	PLK1	MSTN	RAD54B
MTBP	UCP2	PLK4	MTBP	RAD54L
MTF2	WWOX	PRKCA	MTFP1	RBL1
MYBL2		PRKCB	MUTYH	RECQL4
MYC		PRKDC	MYBL2	SHMT2
MYH9		PTGER1	MYC	SLC2A4
NASP		PTTG1	MYH9	SLC4A1
NCAPG		RACGAP1	NASP	SMARCAL1
NEIL3		RAD51	NCAPG2	SMC2
NET1		RBL1	NCAPH2	TACC3
NFIL3		RGS5	NDC80	TGM2
NFIX		RPA1	NET1	TIMELESS
NPM1		RPA2	NFE2L1	TKT
NR4A3		RRM2	NFIL3	TNNT2
NUMA1		SPAG5	NFIX	TOP2A
NUPR2		STMN1	NIF3L1	TRAIP
ODC1		TACC3	NPM1	TRIM63
ORC1		TGM2	NR4A3	TXNRD2
ORC5		TNNT2	NUF2	UCP2
PBK		TOP2A	NUMA1	UHRF1
PCNA		TPX2	NUSAP1	UNG
PER1		TRAIP	ODC1	WWOX
PLA2G10		TSC22D3	ORC2	XRCC4
PLK1		TTK	PALB2	
PLK4		TYMS	PBK	
POLA1		UCP2	PCNA	
POLG		UNG	PER1	
PRC1		WWOX	PHGDH	
PRKCA		XRCC4	PKMYT1	
PRKCB		YBX2	PLA2G10	
PRKDC			PLK1	
PTGER1			PLK4	
PTTG1			PRKCA	
RAB30			PRKCB	
RACGAP1			PRKDC	
RAD51			PTGER1	
RAD52			PTTG1	
RBL1			RACGAP1	
RECQL4			RAD51	

RFC3
RGS5
RIDA
RPA1
RRM2
RTKN2
SHMT1
SHMT2
SLC29A2
SLC2A4
SLC4A1
SMARCAL1
STMN1
SUV39H1
TACC3
TGM2
TIMELESS
TNIK
TOP2A
TPX2
TRAIP
TSC22D3
TTK
TXNRD2
TYMS
UBE2C
UCP2
UHRF1
UPP1
WNK2
WWOX
XRCC4

RBL1
RGS5
RNASEH2A
RPA1
RPA2
RRM2
SLC29A2
SLC2A4
SLC4A1
SPAG5
SPTB
STMN1
TACC3
TGM2
TNNT2
TOP2A
TPX2
TRAIP
TSC22D3
TTK
TXNRD2
TYMS
UBE2C
UCP2
UNG
WWOX
XRCC4
YBX2
