# Supplementary material for: Waveband specific transcriptional control of select genetic pathways in vertebrate skin (Xiphophorus maculatus)
Source: BMC Genomics. 2018 May 10;19:355. doi: 10.1186/s12864-018-4735-5 (PMC5946439; doi:10.1186/s12864-018-4735-5)
Supplement: Supplementary file 5 — Table S5a–k. A list of all differentially modulated genes used by IPA enrichment software to predict the direction of change for each functional class represented in Fig. 6. Table a is FL, tables b–e are the 50 nm wavebands and tables g–k are the 10 nm wavebands. (ZIP 77 kb) [file 12864_2018_4735_MOESM5_ESM.zip › TableS5d_High-Low.pdf]

Upstream I	z-score	Genes in dataset												
BMP2	-2.66	BGLAP	BHLHE40	BMP4	COL10A1	CYP19A1	DKK1	F3	KLF9	POSTN	SOX9	TGM2	TMEM119	TNFRSF11
CDKN2A	-2.69	CCL19	CDKN2A	EEF1A1	FBL	HUNK	MCM4	MCM7	NPM1	PDGFA	PTX3	XCR1		
CST5	-3.32	ANXA6	BRIX1	EBNA1BP2	EVPL	GAR1	NIFK	NOP2	PITRM1	PPL	RRS1	TOMM22		
CTGF	-2.96	COL10A1	EGLN3	HIF1A	IL17A									
CTNNB1	-2.05	ANXA1	APOD	BHLHE40	BMP4	BRMS1L	C6	CAPN1	CDKN2A	COL27A1	COL4A6	CRABP2	CYP19A1	CYP24A1
GH1	-2.21	ANGPTL4	APOE	BMP4	CYB5B	CYP27B1	IGFBP1	IRF1	SMARCA4					
ID2	-2.24	BCL3	CDKN2A	FOXO3	HIF1A	MAP2K6	RAPGEF4	ZBTB16						
ID3	-2.80	BCL3	CDKN2A	ELOVL6	FOXO3	HIF1A	MAP2K6	PREP	RAPGEF4	ZBTB16				
IL1	-2.05	APOE	BHLHE40	CSF1R	CSF3	CTSL	CYP1A1	ELF3	F3	GNRHR	HIF1A	HMOX1	IGFBP1	IL17A
IL12 (compl	-2.03	BCL3	CCL19	IL12B	IL17A	IRF1	ISG15	MAP3K8	PRDM1	PRF1				
IL15	-2.61	ANXA1	CCL19	CDK2AP1	CSF3	GLDC	HMCN1	HSPE1	IL12B	IL17A	IRF1	NCAM1	NR3C2	OGN
IL2	-2.24	BHLHE40	CSF1R	CSF3	CYCS	CYP1A1	E2F4	FOXO3	GDF15	HSP90B1	HSPA5	HSPD1	IL12B	IL17A
KMT2D	-2.43	DKK1	IRF1	NAALADL2	PTGR1	PTGR2	SLC35G2							
OSM	-2.11	ABCC4	ANXA1	ASNS	BHLHE40	BLMH	CSF3	CTSL	CYP19A1	EVPL	F3	HIF1A	HMOX1	HSPA5
OTX2	-2.71	BMP4	CDH4	DKK1	NCAM1	PRDM1	TNC							
RORA	-2.52	APOE	ASNS	CAV3	CYP19A1	ELOVL6	FDFT1	GOT1	IGFBP1	IL17A	PCK1	SLC25A24		
TET2	-2.00	BCL3	BHLHE40	ITGA2	ZBTB16									
TGFB3	-2.55	BHLHE40	CDKN2A	COL11A1	CYP19A1	HTRA1	THBS1	TNC	TNFRSF11B					
Tlr	-2.15	IL12B	IRF1	IRF7	WNT5A	ZC3H12A								
TP73	-2.25	ATG4C	BHLHE40	COL5A2	FOXO3	MMP19	NCAM1	PPL	SERPINA1	SYNE2	THBS1	VMP1		
Ubiquitin	-2.22	HSP90B1	HSPA4	HSPA5	HSPD1	NOS1								
WNT3A	-2.89	ARHGAP18	BGLAP	BMP4	DKK1	FMOD	IL12B	IRS2	NCAM1	OGN	PDGFA	SOX9	THBS1	TNC
FOXO3	-2.46	CAD	FOXO3	GRB14	IGFBP1	IL17A	IRS2	MXI1	PRDM1	SLC1A4				
ERBB3	-2.31	F3	FBLN2	HIF1A	HMOX1	MXI1	PIM2	THBS1	TNC	TNXB	USP14			
Tgf beta	-2.28	ANGPTL4	BGLAP	BHLHE40	CDKN2A	CIITA	COL10A1	CSF1R	FBLN2	HMOX1	HSPG2	IL12B	MMP11	PDGFA
ARNTL	-2.04	CLOCK	ELOVL6	NPY	PER1	PER3								
EPAS1	2.12	ANGPTL4	BHLHE40	CKMT1A/C	EGLN3	FBLN2	HIF1A	HMGCS1	HSPA4	HSPA5	IRS2	KDR	MANF	MB
AHR	2.41	ABCC4	ADAMTS2	CDKN2A	COL11A1	COL14A1	COL27A1	COL5A2	COLQ	CYP19A1	CYP1A1	FBLN2	FOSL1	GOT1
SPDEF	2.63	COL4A6	COL5A2	COL6A2	HIF1A	TNC	WNT5A							
ABCA1	2.17	ANXA1	APOE	CSF3	FDFT1	HSPA5	IL12B							
XBP1	2.18	ATP2A2	HMOX1	HSP90B1	HSPA5	HYOU1	PRDM1	SEC23B	SEC24D	SERPINA1				
E2F1	2.20	BCS1L	BGLAP	BMP4	CDKN2A	COL10A1	CRABP2	CYP27B1	DGKI	E2F4	EIF3I	EYA2	FOXO3	HMGA1
MED1	2.20	ACP6	BCL3	BHLHE40	CYP1A1	CYP24A1	IGFBP1	MB	PRDM1	6-Sep	SMARCA4	THBS1		
F2R	2.21	F3	HMOX1	IL17A	KDR	TGM2	THBS1							
MAPK3	2.22	BMP4	CYP24A1	FOSL1	GNRHR	THBS1								
BMP6	2.24	ARHGAP18	BMP4	CDKN2A	ERRFI1	FOSL1	HMOX1	SCEL	SMARCA1					
MMP3	2.24	DDX39A	EFTUD2	SF3B4	SRSF3	SYNCRIP								
FOXO1	2.25	ANGPTL4	BGLAP	CYCS	ELOVL6	HMOX1	HSPA5	IGFBP1	IL17A	IRS2	MB	MXI1	NPY	PCK1
ATF4	2.29	ASNS	BGLAP	CDKN2A	CHAC1	GDF15	HSP90B1	HSPA5	KLF9	MARS	NARS	PCK1	PSAT1	PTX3
ERK	2.33	BGLAP	CYP1A1	E2F4	FOSL1	FOXO3	GDF15	GOT1	HAS1	HMGA1	HMOX1	IGFBP1	IL12B	IL17A
NFE2L2	2.35	ABCC4	BGLAP	CDC34	FKBP5	FOXO3	GNA14	HMOX1	HSP90B1	LMNA	NARS	NFE2L1	PREP	PSAT1
F2	2.50	ANGPTL4	BCL3	CAD	EDNRB	ESM1	F3	FOSL1	HIF1A	HMGA1	HMOX1	KDR	PDGFA	THBS1
EIF4E	2.80	CDC34	CDKN2A	FOSL1	HIF1A	HMOX1	POLR3D	SNRPA	ZPR1					
Cg	2.90	BHLHE40	COL11A1	CYP19A1	EIF4E	ESM1	GNRHR	HIF1A	HMGA1	IRS2	KNOP1	MMP19	NR3C2	PFKFB3
MYC	4.30	ABCE1	ANXA6	ASNS	ATAD3B	C1QBP	CAD	CDC34	CDKN2A	CLEC3B	CLUH	COL14A1	COL5A2	CRABP2

ZBTB16														
DIXDC1	DKK1	EYA2	FBLN2	FOSL1	FOXB1	FSTL3	HSPE1	HTRA1	LMNA	NCAM1	NOX1	OGN	PDAP1	PRDM1
IRF1	MAP2K6	NOX1	PDGFA	SOX9	TGM2	TNC	TNFRSF11	WNT5A						
PRF1														
IRF1	ITGA2	MAP2K1	MAP2K6	NIFK	NOP2	PDE4A	PIM2	PRDM1	PRF1	PTHLH	TNFRSF11	UCK2	UMPS	
IRF1	IRF7	MCM7	PDAP1	PDGFA	PFKFB2	PFKFB3	PRDM1	PREP	SERPINA1	SLC7A8	TDO2	TECR	TNC	TNFRSF11
TNFRSF11	TNXB	WNT5A												
POSTN	PTHLH	TNFRSF11B												
PFKFB3	SOX9	TNFRSF11B												
HIF1A	HSPA5	HUNK	IL17A	IRF1	PCK1	SLC1A4	TDO2	THBS1						
HSP90B1	HSPD1	HSPE1	IRS2	KDR	MCM4	MCM7	NCOA3	PPM1G	PSAT1	RBBP4	THBS1	UCK2	UMPS	
PRDM1	PRF1	TDO2	THBS1											
TNFRSF11B														
PRDM1	RRS1	TGM2	THBS1											
PSMD11	RRS1	SLC1A4	SLC7A8	SYT5	USP14									
UCK2														
PTX3	SCUBE1	SMARCA1												
CYCS	DKK1	EFTUD2	EIF4E	EIF4G1	EVPL	F3	FBL	FBLN2	FCGRT	FKBP5	FMOD	FOSL1	GOT1	HIF1A

SERPINA1 SOX11 SOX9 TGM2 TNC TNFRSF11B

WNT5A

HMGA1 HMOX1 HSPD1 HSPE1 IL12B IRF7 KDR LRRN1 MCM7 MYBBP1A NARS NCAM1 NOP58 NPM1 NPY

PARP1 PCK1 PFKFB3 POLR3D PPID PPL PRDM1 PREP PRMT1 PSAT1 RBBP4 RCC1 RRS1 RUVBL2 SCEL

SERPINA1 SFXN1 SOX9 THBS1 TIMM23 TNC UBA2 WNT5A
