# Supplementary material for: Waveband specific transcriptional control of select genetic pathways in vertebrate skin (Xiphophorus maculatus)
Source: BMC Genomics. 2018 May 10;19:355. doi: 10.1186/s12864-018-4735-5 (PMC5946439; doi:10.1186/s12864-018-4735-5)
Supplement: Supplementary file 5 — Table S5a–k. A list of all differentially modulated genes used by IPA enrichment software to predict the direction of change for each functional class represented in Fig. 6. Table a is FL, tables b–e are the 50 nm wavebands and tables g–k are the 10 nm wavebands. (ZIP 77 kb) [file 12864_2018_4735_MOESM5_ESM.zip › TableS5c_High.pdf]

Regulator	z-score	Genes in dataset														
AHR	2.377	ADAMTS2	COL11A1	COL14A1	COL16A1	COL1A1	COL1A2	COL27A1	COL4A2	COL5A1	COL5A2	COL9A3	CTSD	DMXL2	E2F1	FGFR2
APOE	-2.807	ABCA1	COL1A1	CTSB	CTSD	CTSV	F3	HMOX1	HSPG2	LDLR	LRP1	PTX3				
ARNTL	-2.222	DBP	FASN	PER1	PER3	SREBF1										
ASCL1	-2.433	E2F1	EPHB3	GADD45G	PLXNA2	RELN	RORB	SALL3								
ATF4	2.617	ATF3	CHAC1	DDIT4	FASN	PRKDC	PTX3	SLC3A2	SREBF1							
BMP2	-2.201	BHLHE40	COL1A1	COL1A2	EVC2	F3	FGFR1	FGFR2	PLXNA2							
CCND1	-2.567	AQP3	ASAP2	ATAD2	CDC45	COL1A1	COL27A1	COL5A2	E2F1	EGFR	FGFR1	HSPB8	MAGI2	MCM10	MCM4	RBL1
CLDN7	2.236	F3	FOSL1	IFI44	ITPR3	PKMYT1										
CR1L	2.646	COL15A1	COL1A1	COL1A2	COL4A2	COL5A1	COL8A1	TNC								
CSF2	-2.953	ABCA1	ATM	CD33	CHTF18	COL8A1	CSF1R	F3	FANCA	FANCL	FIGNL1	FOSL1	IDO1	MCM3	MCM5	MCM6
CTGF	-2.586	COL1A1	COL4A2	COL8A1	EGLN3	ITGA1	MMP14	WNK1								
CTNNB1	-2.598	ACTC1	ADGRG2	AGRN	ATM	BHLHE40	CA3	CAPN1	CNTFR	COL1A1	COL27A1	COL4A2	COL4A6	CYFIP2	CYP24A1	CYP51A1
E2f	-3.403	CDC45	E2F1	ITGA6	LIG1	MCM10	MCM2	MCM3	MCM4	MCM5	MCM6	ORC1	POLA1	RBL1		
E2F1	-2.691	ANGPT2	ARHGAP4	ATAD2	ATM	BNIP3	CA2	CALD1	CBX7	CDC45	CITED2	CTSB	E2F1	EZH2	FGFR1	FGFR2
E2F6	2.219	CDC45	E2F1	LIG1	MCM2	MCM3	MCM5									
EIF4E	1.944	ATR	BNIP3	EGFR	FOSL1	HMOX1	IGFBP4	INSIG1	MIA	ODC1	SVIL	TP53BP1	UPP1			
EPAS1	2.077	ANGPT2	BHLHE40	BNIP3	CITED2	CYP51A1	EGFR	EGLN3	FASN	GYS2	LDLR	MMP14	PRKCA	SREBF1	UGP2	WISP2
ERBB3	-2.353	COL1A1	EGFR	F3	HMOX1	HP	TNC	TNXB								
ESR1	-2.907	ABCC5	ACTC1	ADGRG6	AQP1	AQP3	ARHGEF9	ATF3	ATP2A3	BCAS3	BRCA2	C8orf4	CA2	CALD1	CBL	CELSR2
FAS	2.646	CLASP1	COL15A1	COL1A1	COL1A2	COL4A2	COL5A1	COL8A1	CTSL	F3	GADD45G	HPR	LDLR	PRKDC	PTP4A2	PTX3
FOXO3	2.255	BNIP3	CTSV	DDIT4	FASN	FBXO32	FGFR2	GABARAPL	MSTN	MYOD1	SESN1					
HDAC2	2.828	COL11A1	COL1A2	DPT	F3	GABARAPL	LIG1	MCM10	MCM3	MCM5	TNNI2					
HNF1B	2.121	BHLHE40	CA2	CITED2	COL5A1	COL5A2	EXT1	LAMB1	TTR	UPP1						
HSF1	-2.229	DHX34	FASN	HMOX1	JARID2	LDLR	PDZD2	TTR								
IGF1R	-2.025	ANKRD1	ATM	COL1A1	COL4A2	FASN	IGFBP4	IRS1	MYOD1	NME1	RPL24	SREBF1				
MKNK1	-2.646	AGRN	ANXA5	LAMC1	MYO6	PLXNB2	RNH1	TP53BP1								
MTM1	-2.213	BNIP3	CTSV	FBXO32	GABARAPL	ZFAND5										
MYOC	-2.219	CA2	DAG1	DDIT4	FBXO32	JDP2	LAMB1	LAMC1	STC1	UPP1						
NUPR1	2.173	ABCC5	AGRN	ALOXE3	ATF3	ATR	BNIP3	CACNA2D2	CERK	CITED2	COL1A2	CREB5	DNMT3B	ERMP1	FAM114A1	ITPR3
OTX2	-2.188	COL8A1	CPXM2	MYOD1	NCAM1	OCA2	RHO	SAG	TNC	TTR	TYR					
PDX1	2.121	ATF3	ATP2A3	COL1A1	COL25A1	CREM	F3	FGFR1	INSIG1	PTPRF	SLIT3					
RABL6	-2.121	ABAT	EZH2	FRMD4A	HMOX1	MCM10	MCM2	MCM5	POLA1							
Rb	2.177	E2F1	EZH2	FGFR2	MCM3	MCM5	NEO1	RBL1								
RB1	2.455	ANGPT2	ATAD2	BNIP3	CITED2	CNOT6L	E2F1	EZH2	F3	FANCA	FANCL	FOSL1	LIG1	MCM10	MCM2	MCM3
RBL1	2.789	E2F1	F3	MCM10	MCM2	MCM3	MCM5	MCM6	ORC1	RBL1						
RORA	-2.186	ABCA1	AKR1D1	APOA4	FASN	NR1D2	RHBG	SEMA3F	SREBF1							
SP1	-2.38	ABCA1	ALOX15B	ATF3	ATM	ATP2A3	CITED2	COL1A1	COL1A2	COL7A1	COL8A1	CREM	CTSD	CYP51A1	DNMT3A	E2F1
SPDEF	2.714	COL16A1	COL1A1	COL4A2	COL4A6	COL5A1	COL5A2	DKK3	EGFR	FGFR1	ITGA6	LAMC1	PRKCA	PTPRF	TNC	
SRF	2.377	ACTC1	ANKRD1	CALD1	ELK1	EPX	FOSL1	GADD45G	HSPG2	ITGA1	ITGA6	LDLR	LRP1	LRP4	MSTN	MYOD1
TBX2	-2.887	ATF3	BHLHE40	DBP	E2F1	EZH2	LIG1	MCM2	MCM4	MCM5	MCM6	PKMYT1	RBL1			
Tgf beta	-2.069	ABCA1	BHLHE40	COL1A1	COL1A2	COL7A1	CSF1R	GATA3	GLI2	HMOX1	HSPG2	IDO1	TLR2			
TGFB3	-2.392	BHLHE40	CDH6	COL11A1	COL1A1	COL1A2	COL5A1	SH3PXD2A	TNC							
TNFSF13	2.236	MCM2	MCM4	MCM5	MCM6	XBP1										
TP73	-2.593	ATF3	ATM	BHLHE40	CNTFR	COL5A2	CTSD	DBP	DDIT4	EDA	EGFR	FASN	HSPB8	IGFBP4	JAG2	LIG1
WNT3A	-2	COL1A1	DDIT4	FHOD3	GADD45G	GFI1B	IRS1	MMP14	NCAM1	OGN	RHOU	SLC7A2	TNC	TNXB	TYR	WISP2
ZNF217	2	CITED1	COL8A1	CREB5	DPP6	SHC4	SPAG9									

FOSL1	HP	ITGA6	PLXNA2	POLA1	RBL1	SEMA3F	SLC22A7									
SRGAP2																
MMP14	MPO	ODC1	POLE	SOCS1	TLR2	TPM4	UPP1	XBP1								
EGFR	EPHB3	EPHB4	FBRS	FOSL1	FSTL3	GADD45G	GATA3	GLI2	IDO1	IRS1	ITGA1	ITGA6	LAMB1	ME1	MLLT6	MMP14
IRS1	JAG2	MCM10	MCM2	MCM3	MCM4	MCM5	MCM6	ORC1	PHC1	POLA1	PRKDC	PRPSAP1	RBL1	SLC3A2		
CEP250	COL16A1	COL5A2	COL8A1	CTSB	CTSD	CYFIP2	CYP26B1	DDIT4	DOCK4	E2F1	EGFR	FASN	FGFR1	FGFR2	FLCN	FOSL1
SIGLEC7	TLR2	TNC	TP53BP1	TPM4	WNK1											
MCM10	MMS22L	NR1D2	PER3	PFKFB4	PRAG1	PRR12	SIPA1L2	SLF1	SPIN4	SRGAP2	TP53BP1	UPP1	WNK1	XBP1	ZSWIM6	
MCM4	MCM5	MCM6	ORC1	PTX3	RBL1	SUZ12										
EDA	EGFR	F3	FASN	FGFR1	FOSL1	HMOX1	HSD17B1	IGFBP4	IL21R	LAMA1	LDLR	LTB4R	MMP14	PRKCA	RBL1	SOCS1
RNH1	SKI	SVIL	TCAP	TCF7	TNC	WT1										
MMP14	MMP19	NCAM1	PIEZO2	PRKDC	RELN	TTR	WISP2									
WNT7B	XBP1															

MYOD1	NCAM1	OGN	PDE1C	PTCH1	SEC61A1	SEMA5A	SESN1	SMS	SUZ12	TCF7	TNC	TNIK	WNT7B	WT1		
GATA3	GLI2	IFI27	IFI44	IFI44L	IGFBP4	IRS1	ITGA6	KIF5B	LAMB1	LDLR	LGALS1	LTB4R	LTBP4	MPHOSPH	MYO6	NCOA1
SREBF1	TLR2	TNC														

NLGN4X NME1 NR1D2 NUP210 PAK6 PLCB1 PLXNA2 POLA1 PPP1R9B PPP6R2 PTCH1 PTP4A2 PTX3 PVR RBL1 SEMA3F SESN1

SLC3A2	SLC7A2	SLC7A8	SLC9A2	SLC9A5	SPAG9	TAX1BP1	TP53BP1	TTR	WISP2	WNK1	XBP1
