# Supplementary material for: Waveband specific transcriptional control of select genetic pathways in vertebrate skin (Xiphophorus maculatus)
Source: BMC Genomics. 2018 May 10;19:355. doi: 10.1186/s12864-018-4735-5 (PMC5946439; doi:10.1186/s12864-018-4735-5)
Supplement: Supplementary file 5 — Table S5a–k. A list of all differentially modulated genes used by IPA enrichment software to predict the direction of change for each functional class represented in Fig. 6. Table a is FL, tables b–e are the 50 nm wavebands and tables g–k are the 10 nm wavebands. (ZIP 77 kb) [file 12864_2018_4735_MOESM5_ESM.zip › TableS5b_Low-High.pdf]

Upstream Regulator	z-score	Genes in dataset								
GATA3	-2.126	EVPL	GATA3	HMGA1	HSPG2	SOX9				
TGFB1	-2.627	EDNRB	GAD1	GATA3	HMGA1	HSPG2	SOX9	TNFRSF11B		
CTNNB1	-2.348	EYA2	FOSL1	GAD1	GATA3	SOX9	TNFRSF11B			
IL1	-2.594	CYP1A1	ODC1	SOX9	TNFRSF11	MMP19				
TNF	-2.456	CYP1A1	EDNRB	FOSL1	HSPG2	ODC1	SOX9	TNFRSF11B		
IL1B	2.452	CYP1A1	FOSL1	GAD1	GATA3	HMGA1	HSPG2	ODC1	SOX9	TNFRSF11B
IFNG	2.617	EDNRB	GAD1	GATA3	HMGA1	HSPG2	ODC1	TNFRSF11B		
NFkB (complex)	2.747	EDNRB	GAD1	GATA3	ODC1	TNFRSF11B				
ERK1/2	2.205	FOSL1	GAD1	HMGA1	SOX9	GATA3				
MYC	2.622	EVPL	FOSL1	HMGA1	ODC1	SCEL	SOX9			
ERK	2.982	CYP1A1	FOSL1	HMGA1	ODC1	GATA3				
CYP1A1	6.497	CYP1A1	ODC1	SEP2	SOX9	EDNRB				
