# Supplementary material for: Waveband specific transcriptional control of select genetic pathways in vertebrate skin (Xiphophorus maculatus)
Source: BMC Genomics. 2018 May 10;19:355. doi: 10.1186/s12864-018-4735-5 (PMC5946439; doi:10.1186/s12864-018-4735-5)
Supplement: Supplementary file 5 — Table S5a–k. A list of all differentially modulated genes used by IPA enrichment software to predict the direction of change for each functional class represented in Fig. 6. Table a is FL, tables b–e are the 50 nm wavebands and tables g–k are the 10 nm wavebands. (ZIP 77 kb) [file 12864_2018_4735_MOESM5_ESM.zip › TableS5a_Low.pdf]

Upstream Regulator	z-score	Genes in dataset						
ARNTL	2.60	ARNTL	CLOCK	PER1	PER2	PER3		
BRCA1	2.07	ATM	CYP1A1	EZR	HMOX1	LPIN1		
CLOCK	2.89	BHLHE40	CA1	CLOCK	LPIN1	PER1	PER2	PER3
EGF	2.34	ATM	CYP1A1	EZR	HMOX1	NR4A3	PER1	TGM1
HGF	2.00	ARNTL	ATM	HMOX1	ITPR1	NR4A3		
HIF1A	2.00	ANKZF1	BHLHE40	CXCL12	HMOX1	ITPR1	NR4A3	
IL1	-2.41	BHLHE40	CYP1A1	CYP1A2	HMOX1	PPARGC1A		
P38 MAPK	2.19	CXCL12	CYP1A1	HMOX1	NR4A3	PPARGC1A		
Pkc(s)	2.39	CYP1A1	HMOX1	NR4A3	PER1	PER2	TGM1	
PPARG	2.00	CA2	HMOX1	MPO	MYH14	PPARGC1A		
PPARGC1A	4.23	CYP1A1	CYP1A2	LPIN1	MYH14	PPARGC1A		
TNFSF11	2.11	CA2	CLOCK	CYP1A1	CYP1A2	HMOX1		
Vegf	2.63	ARNTL	CA2	CXCL12	HMOX1	ITPR1	NR4A3	
