# Supplementary material for: Waveband specific transcriptional control of select genetic pathways in vertebrate skin (Xiphophorus maculatus)
Source: BMC Genomics. 2018 May 10;19:355. doi: 10.1186/s12864-018-4735-5 (PMC5946439; doi:10.1186/s12864-018-4735-5)
Supplement: Supplementary file 6 — Table S6a–d. A list of all differentially modulated genes used by IPA enrichment software to predict the direction of change for each upstream regulator represented in Fig. 7. Table a is the list for the low waveband exposure, table b is for the low-high exposure, table c is the high exposure and table d is the high-low exposure. (PDF 28 kb) [file 12864_2018_4735_MOESM6_ESM.pdf]

Ensembl ID	HUGO ID	Probe Sequence
ENSXMAT00000000218	RAVER1	CAGCACAAGGAGTATCGATGCTAGGGGATCTTCCTAAAGAGATGAACCTTCCTCAGAGCGCCTTTCTCAATGCCAATGTTTATCCTTCAGCGAGCAGCAG
ENSXMAT00000000471	ADRB2B	GTCTGGGTGTCAGACAAAAAATCAGCTGTTGAATGCTTATACAGTGAGACCTGCTGTGAATTCAACACCAGTGCAACATATGCAGTCTGTTCCTCAATTG
ENSXMAT00000000868	MTCL1	GGTGGAAAACCGCAGCCTGCGAGCGGAGAACGAAGACATTCGCAGTCAGTATGAAAGAGATTGCTTTGGGAGGGAACCCTTCGCCAGCATGCCCTCGTCT
ENSXMAT00000001067	CREB5	TGTCCTGCTCCTCTGGCGCCTCTCAATCTGGATCAGTGATTACCCAGGCTCCCTCCACCTTAACACATTCAGGGCCGACTCCTGGGGCGCTGTCCTGCCT
ENSXMAT00000001085	CDKN1A	CGCTGAAGAGGAAACAGACGAATATTACAGATTTCTATCAGGCCAAGAGACGAGTTGTGTGGAAGCCGAGGAAATCCGGGGAGTGATTTTCACAGAAACG
ENSXMAT00000001311	PFKFB1	CCAGAGAAGAAAAAGACCTACAACTGTGGATAGTTCCTGTCTAATCTGTGTGGCTGACGTTTCTGATTCTGTTGAACGCATCACAAACAGGTGGAGGTTA
ENSXMAT00000001424	CAMK1G	TACTCCGTCAGCTCTCAGATCCAGAAGAACTTCGCCCAGTCCAAGTGGAAGCAAGCCTTCAACGCCGCTGTAGCCATCAATCACATGAAGAAGCTGCAGC
ENSXMAT00000001625	HSPA4	GCTGACTTTTTACCGGAAAGAGCCATTCATGCTGGAAGCCTATTACAATAATCTGAAGGAGCTAAACTACCCTAACGCAACAATAGGTCAGTTTCTGATC
ENSXMAT00000001952	PRKDC	ATGGACCTACTACTCGCCGTCCTTGACTGCTACAACACCTTTATCGACTTGCACATCATCAAGCCTCAGCACATTCTCAGCTCAAAAGACAAATCTAGCT
ENSXMAT00000001996	PLCH2	CAAAACAGACTGTCACCAAGCAGAGCATCTGATCCCGTCTCAGTTAAACTCAAACGACCACTTCTCGTAGAAACCATCAGGTGCTTTTCCACAGCAGGAA
ENSXMAT00000002257	BHLHE40	CAGAGTGGCATGCAAATCGAGCAACCCACTGTTAGCCAGGAGAAGTCAGAGGAGATGTTTCGCTCTGGTTTCCACATGTGTGCCAAGGAGATTCCTCAGT
ENSXMAT00000002359	NR1D4	CAACGCCAACTACTCCTTCTCCTCCAGTCAGAACCAGTGTCCAGTTAGTGGCAGTTTATCGTCTCAGACCTTCACAGCCAGTCATGGTGTCATGCCAGAC
ENSXMAT00000002554	NOS2	CTGTATTCAGGAGAGATTCACCTCACAGTGGCTGTGGTCTCTTATCGTACCAGAGGTGGTCAAGGACCTATCCATCACGGAGTATGTTCATCGTGGCTCA
ENSXMAT00000002625	WNT5A	TGTGAAGTGCAGGGTGTGCACCACAGTTGTAGACCAATTCGTCTGCAAGTGATGAAAGACCCGTTGCCTTGCGGATGATGGTTGTACATCTGTGTGAATT
ENSXMAT00000002809	TSHB	GCTGCACCTATGATAAGGTGGAATATCGCACAGTCATTCTGCCTGGCTGTGCTATCGACTCCAACCCTGCATATACCTACCCAGTAGCTATCAGCTGCCA
ENSXMAT00000002966	YWHAQ	GTTCACTCCCTGCCAGCTCTGATCCCAGTGATGCGTTCACTGTCGTCTGTTTAGGGTGAACTTATTGATGCCGATTAGTTTGTCAGTGGAGAGAAGCCAT
ENSXMAT00000003105	GADD45A	ATTTCCAGTAAAACGCGGCGCTGGGAGGAAGCAGGGCATGTTTTTATGACGCCTTGTTTTGAGGCCGTTGCCTCAGAGCGCTTGGCGCTTTACCAGGTAT
ENSXMAT00000003254	RORA	TTGTTTAAGAAGCAGCGCTGAAATGAAAACTCACAGCAACTTTCTGGGAGGTTTCCGTCGGCATCGTGATGCAATCGGCGCCTCTTTAAGGACAATCGT
ENSXMAT00000003526	MAP2K1	AAAATCCCAGAGGAGATTCTTGGCAAAGTCAGCATTGCTGTCATCAAAGGTCTTTCCTACCTGCGTGAGAAGCACAAGATCATGCATAGAGATGTCAAGC
ENSXMAT00000003968	PIF1	TCTTCACTGGAAGTGCTGGTACGGGGAAATCCTTCCTGCTGAAGAGAATCCTGGGATCTCTCCCACCGAAGAGCACCTTTGCTACGGCCAGCACAGGAGT
ENSXMAT00000004084	DSCAML1	CCTGCCAAAATCTTGTCATTTGGAGGTACGGTAACCACTCCTTGGATGAAGGAGGTGCGTCTGCCTTGTAGTTCCGTAGGTGAACCAACCACCTACAATCA
ENSXMAT00000004168	MYL2B	GGTGCTGACCCAGAGGAGACCATCCTGAATGCTTTCAAAGTCTTTGACCCTGAGGGAACAGGAACACTAAAGAAAGATTACGTGACGGAGATGTTGACGA
ENSXMAT00000004591	HMOX1	GACCGATCGTGCATCTCAACTTTTCCATAACTTTGCTGGTGGCGAGTTGGAAATCTCGTTAATGCATGCGCTTTGGACTCCGGCTGACCTGCTTGGTGAA
ENSXMAT00000004603	DCT	GTCAAGATTCTCCAGATGGGGAGTGGTGTGCAACAGCTTGGATGATTATAACCGTCTGGTGACTCTTTGCAATGGCACGAATGAGGGTTTTATTCAGAGA
ENSXMAT00000004686	LPIN1	CGTGTCCAAAAGTACACATTTCCGAGCGATCCCTCATCAGACACCTTCAGACCAATGTGCGCCAAGCTCTAAAACACAAGCTCCCTCTTTGCTGATTGAG
ENSXMAT00000004757	CCNF	AGCTCAGGATACTCATCTGTCCAGAGTGTTAGCCCATCATCTACATGCTCCTCGTCCTCCCTCGTGACCCACACGTTCAGGACCTTTACCACATCACTTG
ENSXMAT00000004776	G2E3	TACAACGAAATATTCCCGTCTTACTGTCAGGATCACAGTCCAACCCAGTCTCTCTGCGTAGACTCAGACTTCAGTCTTCCTCAGTCCTGCTCAATATGTT
ENSXMAT00000005334	CDCA7	CTAAGACTGTAAGCTATAACTGAGCAAAAAATTGCCCAGTACACCATCCGCAGTCTTACCTGTGATGAAATGCAATAGTCCTGTGATGTTAGCATTGCCT
ENSXMAT00000005357	PRKCB	AAGCCGTCAGACTCGTCCTCGTCCTCGGTTTGTAAGTACGACAGCAACGGCAATCAGGACCGTGTCAAACTCGCAGACTTCAACTTTATCATGGTTCTGG
ENSXMAT00000006647	PRKCA	CTCAGTGGAGGATGATGGAACGACGAACTGAGGAAGAAATTTGAGGAAGCCAAGTTAGGCCCGGGAAAGAAGATGATCTCAGAGACAGATGACGGCCGC
ENSXMAT00000007109	SYBU	TCGCATCGAATTCGGTTGGGAAGAAAGTTTCTTCGCGCTGCCGTGGCCCTCACATCAGAACGCTCCCGGTTTTCAAACCAGCCGGGGTTCCGTCTTTGAA
ENSXMAT00000007381	DNAH7	GATCCATCATCGACGTCAATCTGCCCAAGTTCCTGGCCCAAGATCTGCAGCTTTTTGAGGGTATCACCTCCGATCTCTTCCCGGGTGTGATGCTCCCGGA
ENSXMAT00000008349	HSP90AA1	GAGAAAGAGAAACAACATCAAGCTTTATGTCCGGAGGGTCTTCATCATGGACAACTGTGAGGAGCTGATACCAGAGTATCTCAATTTCATCAAGGGTGTT
ENSXMAT00000008371	KPNA2	AGGTGTTGGCCGACGCGTGCTGGGCCGTTTCCTACCTTACGGACGGACCCAACGACCGCATTGACGTGGTGGTCAAAACCGGCGTGATTCCTCGCCTGGT
ENSXMAT00000008587	PLCB1	CCTACACTGGCTTCACTGAGGATAGCAGTGTTTGAAGAAGGAGGAAAGTTTATTGGCCACCGCATCATGCCTGTTTCAGCCATCCGTCCAGGTTATCGAT
ENSXMAT00000008665	ENOX1	GAGATCATCGCCATCCGCAAGAGCAAGAAGAACTTCTGCCACATCCGTTTCAGCGAGGAGTTCATGATTGACAAGGCCATATATCTGTCAGGATATCGGA
ENSXMAT00000008690	KLHL38	TTTTGAGACACTTATTGGTTGGATCCGTCATGATCCCGTCTCCAGGCGGGGGACCATAAGCAGCCTTTTCAAAAAGGTCCGTCTTCGACATTTACACCCT
ENSXMAT00000008787	ADAMTS2	ATATGGATGGTGATTCGAACATTCAGGACAACATGCTGGTAGAGGACAGTGCCTATGAATGGGCTCCGAAGAGATGGTCTTATTGTTCCAAAGCTTGTGG
ENSXMAT00000009048	APEX2	TTGCGGACCACCGGCTGGCCCAGGAGCAGTTTTCGGGGGCAGACATTATGCCGGAGGTGGAGGGCTCCGATCACTGTCCCGTTTGGGCGCACCTGAAATG
ENSXMAT00000009163	TGM8	TGGTCAAGCGTGATGGTTCCATGGTAAACATTGAAACGGACTCTATCAAAATCGGGCAAAACATTTCAACCAAGTCAGTCGGCACCAACGACAGGATGAA
ENSXMAT00000009327	CRY2A	CCTGCAGCTGTCTCTTACATCACCTCTGAAACTCTTTACCTCCACCTCTCCCACCTACACTCACCATTCACCAGCTTTGAATGCCTCAGCAGTCTGTTTA
ENSXMAT00000009703	PPAR	CTGCAAGGGTTTCTTTCGGCGGACCATCCGTCTGAAGCTGGAGTACGACAGATGTAAGCGTCGCTGCAAGATCCAAAAAAAGAACCGCAATAAGTGCCAA
ENSXMAT00000010231	GADD45B	CTTCACGCTGCTGCAGTCCTTCTGCTGCGACAGCGGCATCAACATCCTGCGGGTCTCTGGGGTTCAGAGGTTGTGGCAGCTTCTGGGCGGAGTGGACGCC
ENSXMAT00000010831	RAB3B	CTGCAGACCGTACAGATGAGTAGCACTTAGTGGCTGCTGTCCTTTCTGTGTCCGCACCTCCAAGGTTTTTGTATTTCCACTGAACCGCTGGAACGTCCTG
ENSXMAT00000010964	CHK1	CTGTGACGGCATGGCACTTGTCTTCAAACTCACTTGCACCCAACAGGTCACTGTGAGCACTCTGGACAAACGTAACAACAAGCTAATCTTCAAGGTCTTT
ENSXMAT00000011193	NUMA1	AATCAATCTCTCCAGTCTGCAACAGACCAAGTCTTGGCTAAAGAAAACCTGCTAGCTCAGAAGGACACTGAAATTTCCCAGCAAAACGATTCACTTCAAA
ENSXMAT00000011812	FOXN1	CCACCTGTTTACAGGGTTGGACTGCCGGGAGAGTACAATGTTGACCTGAGGGGAATGCATGAGTTACTTCAAGATGGAGACACCAGCTATGACATAGACA
ENSXMAT00000011885	SPC25	TTTAAAAGGAAGGCCAAAGAAATGTGGACTAACTGTATGCTAATGATCTCAAATAGTGATGCTGGCCTTAAGAAGTAATTGATACTCTGGTCTGTAACCG
ENSXMAT00000012579	IRS2	ACAATGGCTACATGCCTATGTGGTGCGGACACAAGATGTCAGTGGAAAGCAGTGATGGAAAGCCAAGCAATGGAGAGTACCTGTACATGTCTCCCGTTGA
ENSXMAT00000013490	GNA15	CACTTCACTTGTGCCACAGACACCAACAACATCCGCAAGGTTTTCACCGACATCAGAGACACGGTCCTGCTCAAGTCGCTAAGGGACTACGGAGTCCTCT
ENSXMAT00000013492	ITPR3	TGGAGATGAAGGAGATCTACACGTCCAACCACATCTGGAAGCTGTTTGAGGATTTCACCGTGGACATGGCGCGGGTCTGCAACAAGCGCGAGAAACGACT
ENSXMAT00000014150	ECT2	GTGACCGCGGCGAACACGTCACACTCTTCCTCTTTAACGACTGTCTGGAGATTGCCAGGAAGCGTCACAAAGTGATCAATACATTTAAAAGTCCAGTGGG
ENSXMAT00000014172	SNCA	GACCCAAACATTCCATGTCGCTCTGCCAAGTCCCGTCGTGCCTCGTCTCTGGTCCGTCTTGGAGTCTGTGCTTGGCGTCTGTGCGTTTCTGTGCGTGCCG
ENSXMAT00000014274	WT1	TGGCATCTCAGCTGGAATGCGTCACATGGAACCAAATGAACTCACTGACATCAACAATGAAGGGCAGTCATGCGATCAGTTATGACAGCGACCCTCCAGC
ENSXMAT00000014334	CCNB2	GGCGATCCTGGTGGACTGGTTGATCCAGGTCCACTCCCGCTTCCAGCTGCTGCAGGAGACGCTGTATCTCACCGTGGCCGTTCTGGACCGCTTCCTGCAG
ENSXMAT00000014517	ARHGAP19	GCTCCCTCCAGCCGCCAGTAAGAAGCATCCTCGTTCCCGCTCCTTTGGTGGCTTTATCAAGCGAAAAGCTCGAGGCGAGCAGATGAGAGAGAGGCACATC
ENSXMAT00000014787	CDK1	AAGGGACCTATGGGGTGGTGTATAAGGGCAGACATAAGTCCACAGGACAAGTTGTGGCTATGAAGAAAATCCGTCTGGAGAGCGAGGAGGAGGGAGTTCC
ENSXMAT00000014944	PCNA	ATTGAGATGAATGAGCCGGTCCAACTGATCTTTGCCCTGAATTACCTGAACTTCTTCACAAAGGCCACACCGCTGTCCAAAACCGTCACCCTCAGTATGT
ENSXMAT00000015684	SLC2A4RG	ACCAGGGCCCATCTTTTACAAATGCGTAACGGACGGAGGCTCGACGGCGGCGGTTAAAAACGAGCGAACTTCGTCCGTCGGTAGCGGGCACTCACCGGGC
ENSXMAT00000015815	YBX2	GTTCATCAGACTGCCATCAAGAAGAACAATCCTAGAAAGTTTCTCCGAAGTGTTGGAGATGGGGAAGTCGTAGAGTTTGACGTAATTGAAGCAACTAAAG
ENSXMAT00000016088	ELOVL6L	GGTCCGGGGGCCAGAAAACTATGAAGAAACGTGTGGAACATTTCAGAACCACCGACCGGACCTCCAGTTTTATCTGAGCAAGTCGTTCATGTTCAGCGCG
ENSXMAT00000016248	PER2	TATACCCTTTAATTGGACATCAAGCACCACCGGTGCCAGTGTATCAGGTAGATTCTGGGGGTGTCCCAATGCAAGCGCAGCCATTTGATCAGACTGCTTT
ENSXMAT00000016798	MAP2K6	TGATGGATCTGGACATCTCCATGAGGACAGTGGACTGTTTTTACACCGTCACTTTCTATGGAGCGTTGTTCAGAGAGGGTGATGTGTGGATCTGCATGGA
ENSXMAT00000016834	CYP1	AGCTTAGTAAACCTCGCAGAAGATTTTGTCCAGGTGGTAGGCAGTGGCAACCCTGCAGACTTCATCCCTGCTATGCAGTATCTTCCCAACAAATCAATGA
ENSXMAT00000016886	KIFC1	GAGTCTCATACGGGTAAAACGGCCGACACACAGAAGAATTATCACTTCAGTTTCGACCGGGTGTTCGGGCCTCTGGCTTCACAGCAGGAGATCTTTGATG
ENSXMAT00000017010	CLOCK	CCCAGCAGGGCCAGACTCAGACCATCAGCATTTCTCAGCAGCAGGAGCAGCAGCAGCAGATTCAGGTGCAGAACCAGGTTTCCGCGCTGCAGGCAGGTCA
ENSXMAT00000017117	WDR76	ATCCCTACGCCTTCAGAATAAACCAGCAGAGAAGTTGACTCTTCCCAAAGAACCTACTGATTTGCTTCACCCTGATCTGGTATTGTATGTAAAGACCCGT
ENSXMAT00000017220	BMPR1B	CACTGACTACCATGAAAACGGATCCTTGTATGACTACCTGAAATCAACCACCCTGGACAATAAAGCCATGCTGCGTTTGGCCTATTCATCTGTTTCGGGA
ENSXMAT00000017412	PARPBP	AAACAACAAAAGAGCTAAAAGAGAGGATTGTCCAGCTTCACCAGACCCAGAAACAAGCTGCTAATGTGGATGGGACGGGTATAAGTCCTGCTAGGCCAAA
ENSXMAT00000017587	MAD2L1	TGAGATCCGCTCTGTCATCAGGCAGATAACAGCCACGGTTACCTTCCTCCCTCTGCTGGAGACGCCGTGTGCCTTTGATCTCCTGGTCTACACTGATAAA
ENSXMAT00000017652	HUNK	GAGAATGTCAAGATGAGATTCGAAATGGATCCTCCTGTGTCCAAGACACACAGTGAACTTGGGACGTCTTGTAATACTGGCCTTGATGACTAATGCACAC
ENSXMAT00000017917	ENSXMAG00000017815	GGACCAGAGGCCGTATCAAAGAAGTTGTCCACATATCCTGGTTATAATGAAACCATTGATCCCAGCATTGCCAACGTGTTTGCCACAGCTGCCTACCGAT
ENSXMAT00000018069	PPP1R27	AGGTCCCTTGAGAATCACAGTCGTCCGAAAGCCGCTGCAAGTTCTTCGGCGGTGAACGGAGAAAGCCGCATCAGTCCAGGTTCCTGGTTTTTGAGCTGCC
ENSXMAT00000018361	FGGY	AAAGCCGCTCACTTTTTTGACCTGCCGGACTTCCTGTCCTGGAAAGCTACAGGCTCCACAACGAGGTCTTTATGTACTCTAGTGTGTAAGTGGACATACT
ENSXMAT00000018403	HP	CAGAGAAGTCGTCTGCTCTGCGCTCTGATGTAATGGCTAAAATGTACCGATAGCAGCCACAGAGCTTCAGGCTCTCTTGTTTTGATAGATGTTTCGCGAG
ENSXMAT00000018595	ATP2A1	GACTGGCACCCTCACAACCAACCAGATGTGTGTGACCAAGATGTTTGTTATCAAGAACGTTGATGGGGACCATGTTGACCTTGATGCCTTTGACATCTCT
ENSXMAT00000019435	XPC	TCCACTCCAGAGAAACCTGGCTGAAGGAAGCTAGAACTGTTCGACTTGGAGAGGAGCCGTACAAGATGGTGAAAGGCTTCTCCAACCGCTCCAGAAAGGC
ENSXMAT00000019667	SIX3	GCTTCCTCTGGTCCTTGCCGGTGGCTCCGGGAGCGTGCGAGGCGATCAACAAGCACGAGTCCATCCTGCGCGCCCGCGCCGTGGTGGCGTTCCACACGGG

ENSXMAT00000020266	SI_CH211-19901.2	CTGATGAAGCAGTGGCCTATGGTGCAGCAGTCCAGGCTGCTATCCTCATGGGTGACACCTCAGAAAATGTCCAAGATTTACTGCTCCTGGATGTGGCACC
ENSXMAT00000000552	FAM102BB	GTGATGAAGCATTAGCGCTGCGATGACAGGATGACCTTGATCACACTTTAAACAAATGGTTTGCGTTGGTTTCTCAATTCCCACAGCAAACACAACGCCC
ENSXMAT00000000653	TPM4	ATCCTGAATGACAGACTCAAGGAGGCGGAAACCCGTGCAGAATTTGCAGAAAGGACGGTGTCAAAGCTTGAAAAGACCATAGACGACCTAGAAGAGAACC
ENSXMAT00000003489	SORT1	GCCATTTCTTCCCTGCGGGGCGTCTACTTGACCAGCATACTCACAGAAGACGGTAACATAGAGACAGTGATCACGTATGACCAAGGAGCCAAGTGGCAGC
ENSXMAT00000004652	WDR37	GAACGGGAGTTTGAGAATCTGTACATTGAAAACCTTGAATTGCGCCGGGAAATCGACTCTCTCAACGAGCGTCTGACTGGAGACGGACAGGCCATCGAGG
ENSXMAT00000007048	LCP2A	CACCGGCGGACGCATCGGAACCGCCAAGATCCAAACCTCCGGTTCTTCCTCCTCCCACCTCCTCCGTCAGCAGGAGTAACTCCTCTGCCAGAGTAGCGCC
ENSXMAT00000009755	FLT4	ACGCAACGCCAGCGTTTCCGTCATCTATAAGTGCTCTGCTGAAAACAAAGTGGGCAAAGATGAGTTCCCTATCTACTTTTATGTGACCACCATTCCTGAA
ENSXMAT00000010762	INTS9	TCTTTACAGAGCCAGATTTCTCCTACCTGGATGCCCTGGCTCCGTACCAGCCGCTAGCCATGAAATGTGTTTACTGTCCTATTGACACCAGGCTCAACTT
ENSXMAT00000012666	SI DKEY-1C7.1	CAGTGATCACCTCTAGCAACACACAAAATGGACGAATCAAACTCTCTGATGACAGGAAGTCAAGAATATTCACAGTGACCATTTCCAGTCTGACCCTGAA
ENSXMAT00000016883	PCP4	GACAAGGATCCGGGCCTGCTGCTGGCAACAACAAGACCTCTGGGGGAAAAGGTCCAGAAGAGTCCAAGGGAGAGATCCCAGAGGATTTTGACATTGATAT
ENSXMAT00000019159	TMEM204	GCTGCTGCTGTTACTACTGATGTGTTTTACCAGCTGACAAAACGTAGTTAATAATGTTAGAGATAAAGTTTGGAGCAGACATGATGGTAGTGGTGGGCAC

*Housekeeping genes used for data normalization are highlighted in yellow.
